# The bZIP protein from *Tamarix hispida*, *ThbZIP1*, is ACGT elements binding factor that enhances abiotic stress signaling in transgenic Arabidopsis

**DOI:** 10.1186/1471-2229-13-151

**Published:** 2013-10-04

**Authors:** Xiaoyu Ji, Guifeng Liu, Yujia Liu, Lei Zheng, Xianguang Nie, Yucheng Wang

**Affiliations:** 1 State Key Laboratory of Tree Genetics and Breeding (Northeast Forestry University), 26 Hexing Road, 150040 Harbin, China

**Keywords:** Abiotic stress, bZIP transcription factor, *Arabidopsis thaliana*, Gene expression regulation, *Tamarix hispida*, Yeast one-hybrid

## Abstract

**Background:**

*Tamarix spp.* are woody halophyte, which are very tolerant to abiotic stresses such as salinity and drought, but little is known about their specific stress response systems. Basic leucine zipper proteins (bZIPs) play important roles in the ability of plants to withstand adverse environmental conditions. However, their exact roles in abiotic stress tolerance are still not fully known. In the current study, we functionally characterized a *bZIP* gene (*ThbZIP1*) from *Tamarix hispida* in response to abiotic stresses.

**Results:**

We addressed the regulatory network of *ThbZIP1* in three levels, i.e. its upstream regulators, the cis-acting elements recognized by *ThbZIP1*, and its downstream target genes. Two MYCs were found to bind to E-box, in the promoter of *ThbZIP1* to activate its expression. Expression of *ThbZIP1* is induced by ABA, salt, drought, methyl viologen and cold. ThbZIP1 can specifically bind to ACGT elements, with the highest binding affinity to the C-box, followed by the G-box and lastly the A-box. Compared with wild-type (Col-0) Arabidopsis, transgenic plants expressing *ThbZIP1* had an increased tolerance to drought and salt, but had an increased sensitivity to ABA during seed germination and root growth; meanwhile, ROS level, cell death and water loss rate in transgenic plants were significantly reduced. Microarray analyses showed that many ROS scavenging genes were up-regulated by *ThbZIP1* under salt stress conditions.

**Conclusions:**

Based on these data, we suggest that ThbZIP1 confers abiotic stress tolerance through activating stress tolerance genes to modulate ROS scavenging ability and other physiological changes involved in stress tolerance, and plays an important role in the ABA-mediated stress response of *T. hispida*.

## Background

Basic leucine zipper proteins (bZIPs) are a large family of transcription factors (TFs) in plants that contain a characteristic and highly conserved basic domain with two structural features: a basic domain responsible for sequence-specific DNA binding and an adjacent heptad leucine repeat domain referred to as a leucine zipper dimerization motif [1,2]. The bZIPs are involved in diverse physiological processes such as seed maturation and germination, plant senescence [2], photomorphogenesis and light signaling [3], and also play important roles in the ability of plants to withstand adverse environmental conditions. Transformed plants that overexpress *bZIP* genes have improved tolerance to stresses of salt [4,5], water deficits [6,7], freezing [1,8], methyl viologen-oxidative [1], heat shock [9], hypersensitivity to ABA [10], and pathogen infections [2]. In addition, the *bZIP* gene is also found to reprogram amino acid metabolism during low energy stress [11].

The *bZIP* genes can regulate the expression of genes involved in stress tolerance. For example, a *bZIP* gene from *Poncirus trifoliata* can upregulate the genes involved in stress tolerance, including *LEA*, *CDPK* and *DREB*[6]. Overexpression of a tomato *bZIP* gene can regulate stress-related genes, such as *AtRD29A*, *AtCOR47* and the SlCI7-like dehydrin [12]. The Arabidopsis *bZIP28* can form a transcriptional complex that upregulates the expression of ER stress-induced genes [13]. The investigation of the target genes regulated by bZIPs on a genome scale may provide information regarding the gene expression regulation network mediated by this TF. Some studies have investigated the target genes regulated by bZIPs, but only a few target genes were investigated, and the study of target genes regulated by bZIP on a genome-wide scale is needed.

*Tamarix hispida* Willd. is a woody halophyte that can grow well in drought prone soils and salinity soil. Previously, we had cloned a *bZIP* gene (*ThbZIP1*) from *T. hispida*, and the transgenic tobacco overexpression of *ThbZIP1* showed improved salt tolerance [14]. In the current study, we further showed that the *ThbZIP1* gene is transcriptionally regulated by *MYC* genes. ThbZIP1 specifically binds to ACGT elements, including C-box, G-box and A-box, and can upregulate serial stress related genes. Moreover, overexpression of *ThbZIP1* led to a reduction in the cellular levels of ROS, cell death and water loss rate under salt, drought and ABA treatment conditions. These results suggest that *ThbZIP1* plays a pivotal role in the fine-tuning ABA signaling and controls ROS accumulation.

## Results

### Cloning the promoter of *ThbZIP1* and analysis of its activity

The cloned promoter of *ThbZIP1* is 1,571 bp in length. To analyze promoter activity of the promoter of *ThbZIP1*, Arabidopsis plants harboring the *ProThbZIP1::GUS* transgene were analyzed using GUS staining (Additional file 1: Figure S1A). The results showed that GUS activity can be detected in all tissues of Arabidopsis, including seeds, pistils, anthers and stamens, in addition to whole seedlings at different developmental stages (Additional file 1: Figure S1B), which indicates that it has high promoter activity in different plant tissues.

### The expression of *ThbZIP1* is activated by ThMYC6

**Figure 1:**
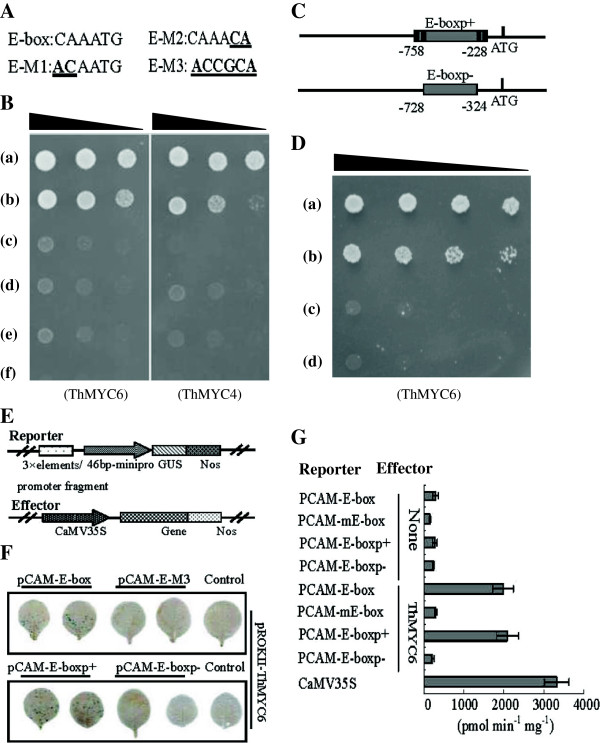
**Analyses of the upstream regulator of ThbZIP1. A**: E-box and its mutated sequences. **B**: The bindings of *ThMYC6* / *ThMYC4* to E-box and the mutated E-box sequences. a: pHIS2-p53 + pGAD-Rec2-p53 (positive control); b: pHIS2-E-box + pGAD-Rec2-*ThMYC6*/*ThMYC4*; c: pHIS2-E-M1 + pGAD-Rec2-*ThMYC6*/*ThMYC4*; d: pHIS2-E-M2 + pGAD-Rec2-*ThMYC6*/*ThMYC4*; e: pHIS2-E-M3 + pGAD-Rec2-*ThMYC6*/*ThMYC4*; f: pHIS2-p53 + pGAD-Rec2-*ThMYC6* /*ThMYC4* (negative control). pGAD-Rec2-53: p53 as a fusion with the GAL4 AD in pGAD-Rec. pHIS2-E-box, -E-M1, -E-M2, -E-M3: three tandem copies of E-box, its mutants E-M1, E-M2 and E-M3 (shown in Figure 1A) respectively inserted into pHIS2. pGAD-Rec2-*ThMYC4*, -*ThMYC6*: *ThMYC4* and *ThMYC6* as a fusion with the GAL4 AD in pGADT7-Rec2. The triangle indicates serial dilutions (1/1, 1/10, 1/100). **C**: The promoter fragments of *ThbZIP1* with or without the cis-elements used in analyses of the yeast one-hybrid and coexpression of reporter and effector vectors. The black lines indicate the E-box, and the grey lines show the promoter fragments. **D**: The bindings of TFs to promoter fragments with or without the E-box motifs using yeast one-hybrid analysis (shown in Figure 1C). a: pHIS2- p53 + pGADT7-Rec2-p53(positive control); b: pHIS2-ProE(+) + pGADT7-Rec2-*ThMYC6/ThMYC4*; c: pHIS2-ProE(-) + pGADT7-Rec2-*ThMYC6/ThMYC4*; d: pHIS2-p53 + pGADT7-Rec2-*ThMYC6/ThMYC4* (negative control). pHIS2-ProE(+), -ProE(-): pHIS2 harboring the promoter fragments containing E-box, or without E-box motifs. **E**: Diagrams of the reporter and effector vectors. **F**: The coexpression of reporter and effector vectors in tobacco leaves. pCAM-E-box, -E-M3: three tandem copies of E-box, or its mutant E-M3 were respectively fused to a minimal promoter to drive GUS. -E-boxp+, -E-boxp: one copy of promoter fragment containing E-box, or without E-box was respectively fused to a minimal promoter for driving GUS. pROKII-*ThMYC6*: the ORF of *ThMYC6* was under the control of CaMV 35S promoter. **G**: The GUS activity assay of the coexpression of reporter and effector plasmid. Error bars indicate SE.

The cis-elements in the promoter of *ThbZIP1* were predicted, and diverse cis-elements were found, such as E-box, ABRE, DOFCOREZM, MYBCORE and W-box (Additional file 2: Figure S2). Additionally there were seven E-box (“CANNTG”) motifs in this promoter (Additional file 2: Figure S2), indicating that E-box motif is important for regulating the expression of *ThbZIP1*. Therefore, yeast one-hybrid analysis was performed to investigate the TFs that can bind to E-box motif. Two *MYC* genes (*ThMYC4* and *ThMYC6*) were found to bind to the E-box motif (Figure 1B). In addition, ThMYC6 bound more strongly to E-box than ThMYC4, which was used for further study. To further understand the specificity of these interactions, the E-box motif was mutated (Figure 1A) and the interaction between the TFs and the mutated motifs were investigated using yeast one-hybrid analysis. The results showed that ThMYC6 failed to interact with these mutated motifs (Figure 1B), which indicates the specificity of its binding to E-box motif.

To further determine if *ThMYC6* can activate the expression of *ThbZIP1* by binding to E-box motifs in the promoter, the pHIS2 vectors that contained the *ThbZIP1* promoter fragments with E-box motifs (pHIS2-ProE(+)) and without E-box motif (pHIS2-ProE(-)) (Figure 1C) interacted with the pGADT7 constructs that harboring *ThMYC6*. The results indicated that *ThMYC6* can interact with the promoter fragment containing the E-box motifs, but failed to interact with the promoter fragment that lacked the E-box motifs (Figure 1D).

To further confirm the interaction of *ThMYC6* with E-box, the effector construct (pROKII-*ThMYC6*) was co-transformed into tobacco leaves together with its corresponding reporter plasmids (pROKII-*ThMYC6* cotransformed with pCAM-E-box, pCAM-E-M3, pCAM-E-boxp + or pCAM-E-boxp-, respectively). Histochemical staining and a GUS activity assay showed that the *GUS* gene was activated in the tobacco cells when the co-transformation of pROKII-*ThMYC6* with pCAM-E-box or pCAM-E-boxp + were performed. However, the co-transformation of pROKII-*ThMYC6* with pCAM-E-boxp-, and the negative controls all failed in GUS activation (Figure 1F, G). These data clearly indicated that ThMYC6 can activate gene expression through its interaction with the *ThbZIP1* promoter *via* binding to the E-box motifs.

### The expression of *ThbZIP1* is induced by abiotic stresses and ABA

**Figure 2:**
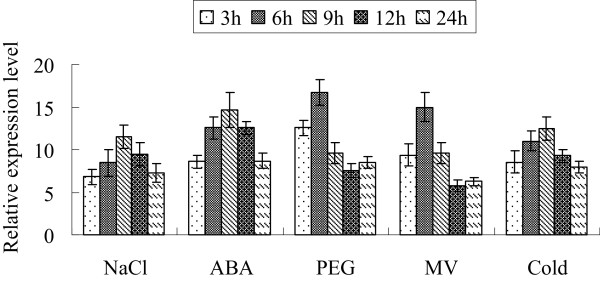
**The expression patterns of *ThbZIP1 *in response to abiotic stresses and ABA.** The expression patterns of *ThbZIP1* in response to salt, osmotic, oxidative, and cold stress and ABA treatment. The error bars were obtained from multiple replicates of the real-time PCR.

Previously, we had studied the expression of *ThMYC6*, and the results showed that it can be highly induced by salt, osmotic and ABA treatments [15]. In the present study, real-time RT-PCR results showed that *ThbZIP1* is highly induced by treatments with NaCl, PEG6000, ABA, methyl viologen (MV) or cold (Figure 2). These results suggested that both *ThMYC6* and *ThbZIP1* respond to abiotic stresses and are involved in the ABA signaling pathway.

### The motif sequences recognized by ThbZIP1

**Figure 3:**
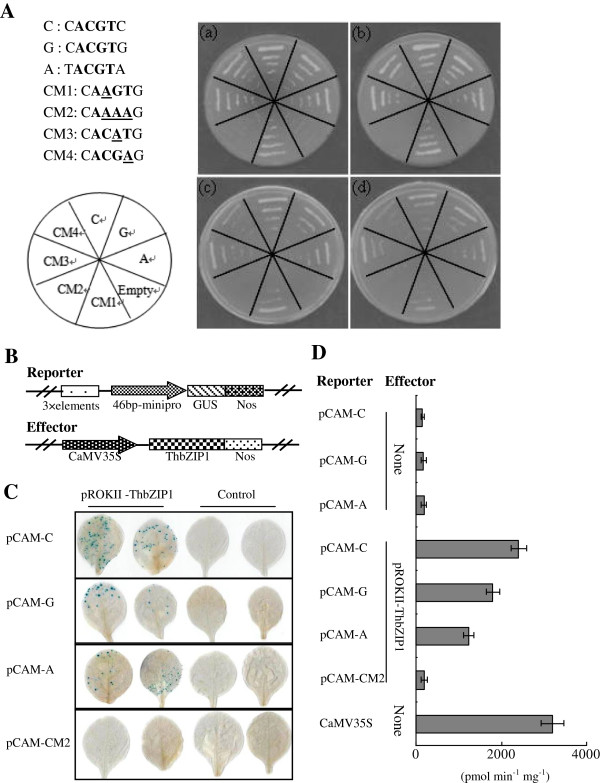
**Analyses of the bindings of ThbZIP1 to C-, G- and A-box. A**: (a-d): Analysis of the bindings of *ThbZIP1* to C-, G-, A-box and their mutated sequences by yeast one-hybrid analysis. The yeast cells were grown on different intensities of selective dropout medias: SD/- Trp-Leu/-His (TDO) + 3-AT (3-AT concentrations, a: 30 mM, b: 40 mM, c: 50 mM, d: 60 mM). **B**: A diagram of the reporter and effector vectors. **C**: The coexpression of reporter and effector vector in tobacco leaves. pCAM-C, -G -A, -CM2: three tandem of C-, G-, A-box, or their mutant CM2 was respectively fused to a minimal promoter (-46 to +1) to drive GUS. pROKII-ThbZIP1: the ORF of *ThbZIP1* was under the control of CaMV 35S promoter. **D**: GUS activity assay of the coexpression of reporter and effector plasmid. CaMV35S: The transformation of pCAMBIA1301 alone (positive control). The transformation of the reporter plasmids alone were used as negative controls. All assays were repeated three times and error bars indicate SE.

The ThbZIP1 can bind to C-, G- and A-box sequences, but binds more strongly to C-box, followed by G-box and lastly A-box motifs (Figure 3A). Among the four mutants of ACGT elements, ThbZIP1 fails to bind to the CM2 mutant, but is able to bind to the CM1, CM3 and CM4 to some extent. Furthermore, ThbZIP1 is able to bind to CM3 more strongly than CM1 and CM4 (Figure 3A). To confirm these interactions, the effector construct (pROKII–*ThbZIP1*) and reporter plasmids (pCAM-C-box, -G-box or -A-box) were coexpressed in the tobacco leaves. Consistent with the yeast one-hybrid analyses, GUS staining and activity measurements both showed that ThbZIP1 can bind more strongly to C-box sequences, followed by G-box and A-box (Figure 3C, D).

### Constitutive expression of *ThbZIP1* enhances salt tolerance

**Figure 4:**
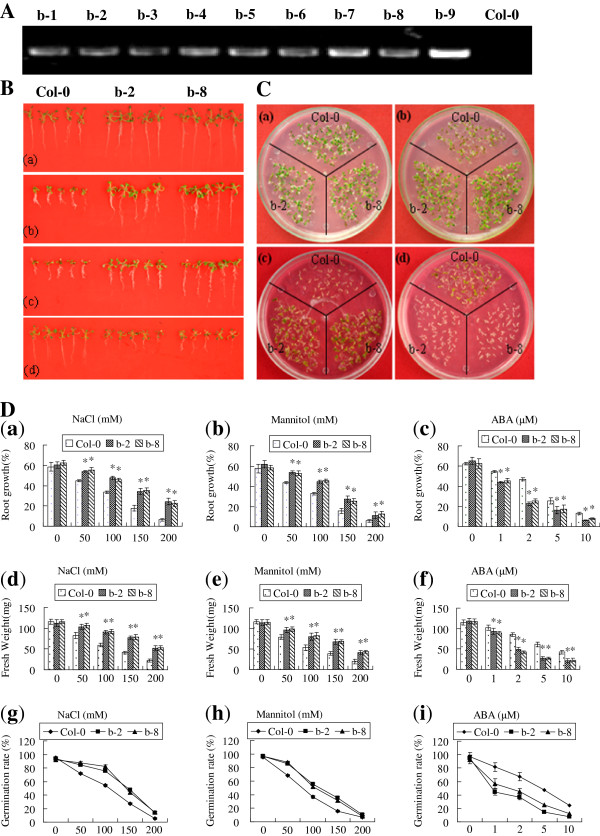
**Comparisons of germination rate and growth between ThbZIP1 transformed and Col-0 plants. A**: RT-PCR detected the expression of exogenous *ThbZIP1* in transgenic plants. **B**: The growth of *ThbZIP1* transformed and Col-0 plants under salt, drought, ABA and normal growth conditions; a, b, c, plants under the conditions of normal growth (a), salt (b), drought (c), and ABA (d). **C**: A comparison of the seed germination rates between *ThbZIP1* transformed and Col-0 plants under normal growth condition (a), salt (b), drought (c) and ABA (d) stress conditions; **D**: An analysis of root growth (a, b, c), fresh weight (d, e, f) and germination rate (g, h, i) of *ThbZIP1* transformed and Col-0 plants under salt, drought and ABA stress conditions. Data are means ± SD from three independent experiments. * Significant (t test, P < 0.05) difference compared with Col-0 plants.

Nine independent T_3_ homozygous lines overexpressing *ThbZIP1* were generated, and RT-PCR confirmed that the exogenous *ThbZIP1* was expressed in the transgenic plants (Figure 4A). Two independent *ThbZIP1* transgenic lines (b-2, b-8) were selected for further study. Under normal growth conditions, there was no difference in growth between Col-0 and transgenic plants (Figure 4B, C, D). However, the transgenic lines showed significant improved root growth and fresh weight under drought or salt stress as compared with wild-type (Col-0) plants. In addition, the *ThbZIP1* transgenic plants exhibited an increase in seed germination under NaCl or Mannitol stress in comparison with Col-0 plants (Figure 4C, D). These results suggest that *ThbZIP1* conferred an enhanced salt and drought tolerance to *ThbZIP1* transformed plants.

In the absence of exogenously applied ABA, the percentage of successful germinations of transgenic seeds and plant growth were similar to those of Col-0 seeds. However, the germination of *ThbZIP1* transgenic seeds was more sensitive to exogenously applied ABA as compared to Col-0 (Figure 4C, D). There were no obvious differences in the above ground organs between the *ThbZIP1*-transformed plants and Col-0, but the root length of the *ThbZIP1* transgenic plants was shorter in response to exogenously applied ABA (Figure 4B, D).

### The target genes regulated by ThbZIP1

The expression profile changes between Col-0 and *ThbZIP1* overexpressed plants were compared under normal growth and salt stress conditions using Agilent Arabidopsis Oligo microarray. A total of 241 and 322 genes were upregulated and downregulated in *ThbZIP1* overexpressed plants, respectively, under normal growth condition (Additional file 3: Table S1). Under salt stress conditions, 1,204 and 1,228 genes were significantly upregulated and downregulated in *ThbZIP1*-transformed plants, respectively (Additional file 4: Table S2).

Twenty-four significantly differentially expressed genes (12 genes in normal condition, and 12 genes in the NaCl response) identified by microarray were randomly selected for real-time RT-PCR analyses. There were high correlation coefficients between the real-time PCR and microarray data (R^2^ = 0.9817, P < 0.05 under normal growth conditions; R^2^ = 0.9764, P < 0.05 under salt stress conditions) (Additional file 5: Figure S3), which validates the reliability of the microarray results.

Gene Ontology (GO) analysis was conducted of the pathways significantly (P < 0.05) enriched in the differentially regulated genes (Additional file 6: Table S3). Under salt stress conditions, the upregulated genes were significantly enriched in 17 subgroups, and the downregulated genes were significantly enriched in 16 subgroups. Among these enriched subgroups, seven subgroups were enriched for both up- and down-regulated genes, such as binding, metabolic process, cellular process and response to stimulus, indicating that these pathways were highly altered in response to salt stress. The subgroup of catalytic activity pathways were only enriched by the upregulated genes under salt stress, indicating that this pathway may play a positive role in ThbZIP1-modulated salt stress response.

To study whether the genes can be directly regulated by *ThbZIP1 via* binding to the C-, G- or A-box in their promoters, we randomly selected the genes upregulated by *ThbZIP1* and screened their promoter regions for searching C-, G- or A-box motifs. The results showed that all of these genes have at least one C-box, G-box or A-box and most of them have two or more C-, G- or A-box motifs in their promoter regions (Additional file 7: Table S4). This result suggested that *ThbZIP1* can directly regulate the expression of genes *via* binding to the C-, G- or A-box motifs present in their promoters.

### Physiological roles mediated by of ThbZIP1

**Figure 5:**
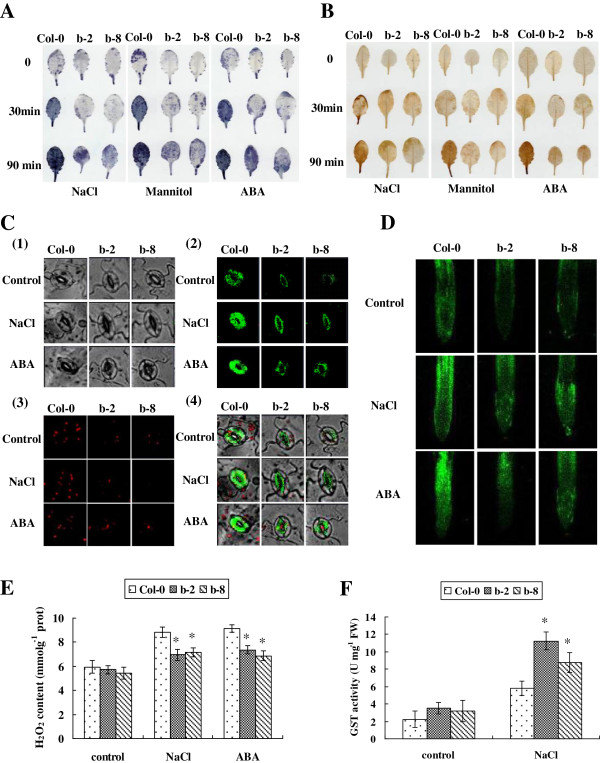
**The constitutive expression of *ThbZIP1 *decreases ROS levels and enhanced GST activity. A, B**: The leaves from Col-0 and transgenic plants were pretreated with NaCl, mannitol, or ABA, and were stained with NBT to visualize O^2-^**(A)**, or were stained with DAB to visualize H_2_O_2_**(B)**. **C**: Analyses of ROS production in intact guard cells of transgenic and Col-0 plants indicated by H_2_DCF-DA. Epidermal peels were loaded with H_2_DCF-DA after the incubation with 10 μM ABA or 150 mM NaCl for 2 h. (1) peel cells imaged under bright field; (2) ROS in guard cells were detected by H_2_DCF-DA (ROS shown as green fluorescence); (3) Chloroplast in the leaves that shown as red fluorescence; (4) merge of bright field and fluorescence (chloroplast shown as red fluorescence). **D**: Detection of ROS accumulation in root tips of transgenic and Col-0 plants using H_2_DCF-DA staining method. **E**: Measurement of H_2_O_2_ level in transgenic and Col-0 plants. **F**: Comparison of GST activity between Col-0 and ThbZIP1-transformed plants. All experiments were repeated three times. Data are means ± SD from three independent experiments. * Significant (t test, P < 0.05) difference compared with Col-0 plants.

The cellular levels of O^2-^ and H_2_O_2_, the two prominent ROS species, were compared between transgenic and Col-0 plants by NBT and DAB *in situ* staining, respectively. The steady state levels of both H_2_O_2_ and O^2-^ were displayed as deep brown and dark blue products, respectively, and were highly reduced in leaves of *ThbZIP1* transgenic plants compared with Col-0 plants under salt, drought and ABA stress conditions (Figure 5A, B). Consistent with DAB staining, H_2_O_2_ content measurement also showed that there was no difference in H_2_O_2_ content between transgenic and Col-0 plants under normal growth condition, but H_2_O_2_ content in the transgenic plants was significantly decreased compared with that in Col-0 plants under salt and ABA treatment (Figure 5E).

The cellular level of ROS in guard cells and root tips were examined using H_2_DCF-DA fluorescence staining. ROS levels in the guard cells and the root tips of the transgenic plants were notably lower than those in Col-0 plants (Figure 5C, D). These observations indicated that *ThbZIP1* expression enables plants cells to decrease ROS amounts to alleviate the damage caused by stress.

Under salt stress conditions, 7 unique GST genes were highly upregulated by overexpression of *ThbZIP1*, and no GSTs were downregulated (Additional file 4: Table S2); therefore, we further studied the GST activities in response to salt stress. Consistent with the gene expression profiles of GSTs, the transgenic and Col-0 plants showed similar GST activities prior to stress, but the transgenic lines showed significantly higher GST activities than Col-0 plants did under salt stress conditions (Figure 5F). Taken together, these results suggested that GST genes play important roles in *ThbZIP1* mediated elimination of cellular ROS generated by salt stress.

**Figure 6:**
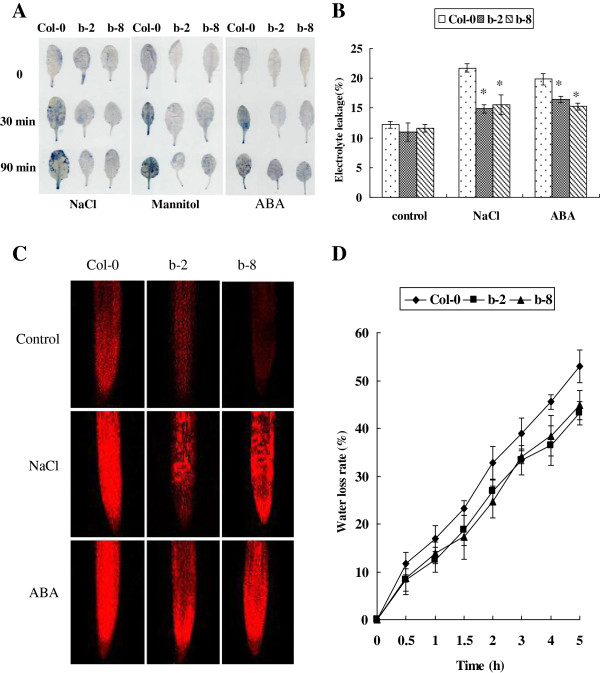
**The constitutive expression of *ThbZIP1 *decreases cell death and water loss rate under stress conditions. A**: Cell death staining with Evans blue before (Control) and after the treatment of NaCl, mannitol, or ABA. **B**: Comparison of electrolyte leakage between Col-0 and *ThbZIP1* transformed plants. **C**: Assay of cell death between transgenic and Col-0 plants using propidium iodide (PI) fluorescence staining. **D**: Transpirational water loss rate analyses in Col-0 and ThbZIP1 transformed plants. All experiments were repeated three times. Data are means ± SD from three independent experiments. * Significant (t test, P < 0.05) difference compared with Col-0 plants.

Evans blue staining showed that the cell death in *ThbZIP1* transgenic plants were notably reduced after salt, drought and ABA treatments compared with the Col-0 plants (Figure 6A). The levels of cell death in root tips were examined using propidium iodide (PI) fluorescence staining. As shown in Figure 6C, under normal, ABA and salt stress conditions, there were notably fewer dead cells in the transgenic plants than in Col-0 plants. Moreover, electrolyte leakage assay also showed that after salt and ABA treatments electrolyte leakage in transgenic lines was significantly lower than in Col-0 plants (Figure 6B). This result was consistent with the results of Evans blue and PI staining, which indicated that the overexpression of *ThbZIP1* could protect the cells from death under stress conditions.

In addition, the water loss rate assay showed that the leaves of *ThbZIP1*-expressing transgenic plants exhibited delayed water loss rate relative to Col-0 plant leaves under dehydration conditions (Figure 6D), which demonstrated that the capacity to conserve water is enhanced by the expression of *ThbZIP1*.

## Discussion

Despite the fact that transformation of *bZIP* genes leads to improved abiotic stress tolerance, the molecular mechanisms of the tolerance and sensitivity remain largely unknown and were explored here. The microarray results showed that compared under normal growth condition, the number of differentially expressed genes increased by nearly 5-fold under salt stress conditions (Additional file 4: Table S2). This indicated that the expression of this TF alone is not sufficient to activate many stress-related genes, but needs to be combined with stress signals. Previous studies also supported this point; for example, a bZIP protein, SlAREB, alone cannot activate the expression of *AtRD29A* in Arabidopsis until ABA is supplemented [12]. Also, the expression of succinate dehydrogenase 2–3 and *LEA* can only be regulated by ABI3 in Arabidopsis in the presence of ABA [16].

Modulation of ROS levels is critical for the tolerance of abiotic stresses, given that ROS levels can reflect the damage of cellular components and act as signaling molecules at low concentrations [9]. Plants possess a complex antioxidant system to detoxify stress-induced ROS, which includes several enzymes to scavenge ROS and protect the cells against oxidative stress. The microarray results showed that compared with in Col-0 plants, the ROS scavenging genes in transgenic plants found in higher levels included *GST*, *POD*, *SOD*, L-ascorbate peroxidase (*APX*), thylakoidal ascorbate peroxidase and glutathione peroxidase (Additional file 4: Table S2). GSTs are involved in plant cell stress signaling and provide protection by detoxifying endogenous plant toxins under stress conditions [17]. This report has demonstrated that; (1) constitutive expression of *ThbZIP1* leads to 7 unique *GSTs* that were highly upregulated under salt stress conditions, and no *GSTs* were downregulated (Additional file 4: Table S2); correspondingly, GST activity in transgenic plants was highly improved (Figure 5E); (2) ROS levels were greatly decreased in *ThbZIP1* overexpressed plants compared with Col-0 under salt stress conditions (Figure 5A-D); (3) overexpression of *ThbZIP1* conferred tolerance to salt stresses (Figure 4); (4) GSTs can efficiently detoxify ROS induced in plants by various stress stimulus. These results combined strongly indicate that GSTs play an important role in *ThbZIP1* mediated ROS scavenging to enhance plant tolerance. In addition, our previous studies showed that ThbZIP1 can improve the activities of POD and SOD, and decreased MDA levels under salt stress conditions [14]. These results together suggested that ROS scavenging capacity is enhanced in the plants that overexpress *ThbZIP1* under stress conditions. This conclusion was also further supported by the histochemical assay described here. DAB and NBT staining, H_2_O_2_ measurement and ROS detection in guard cells and root tips using H_2_DCF-DA all clearly demonstrated that the *ThbZIP1* transgenic lines accumulated remarkably less ROS compared with Col-0 under stress conditions (Figure 5A-E). Furthermore, Evens blue and PI staining and electrolyte leakage assay all showed a decrease in cell death rates in *ThbZIP1* transgenic plants under stress conditions compared with Col-0 plants (Figure 6A-C). These combined results indicate conclusively that *ThbZIP1* plays a critical role in ROS scavenging, which prevents the oxidative damage of cells and decreases cell death.

What deserves to be mentioned is that, although ThbZIP1-transformed Arabidopsis plants showed improved ROS scavenging and decreased membrane damage under ABA treatment, still they are sensitive to ABA. Obviously, ABA may cause damage to the plants overexpressing *ThbZIP1* through other pathways rather than ROS injury and membrane damage.

## Conclusion

**Figure 7:**
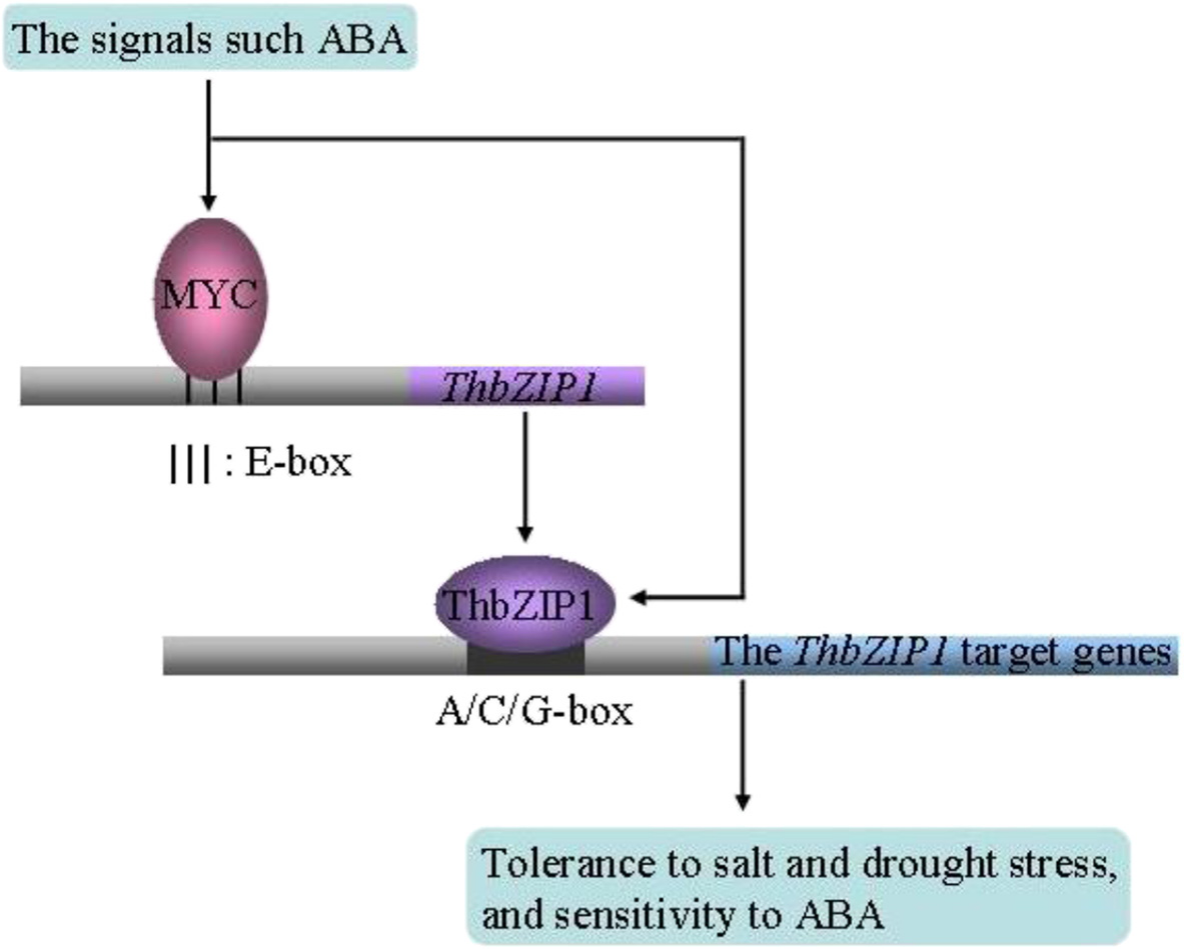
**Model of the regulatory network of ThbZIP1 involved in abiotic stress responses.** Stress signals such as ABA are produced in plants when exposed to abiotic stressors, and the expression of *MYC* is induced by stress signals; then the MYC proteins bind to the E-box motif in the promoter of *ThbZIP1* to activate the expression of *ThbZIP1*. The ThbZIP1 in turn binds to the A-, C- or G-box in the promoter of the genes to alter their expression, resulting that abiotic stress tolerances are improved in plants.

Based on all the experimental data presented here, a model is proposed to explain the ThbZIP1-mediated salt tolerance (Figure 7). Stress signals such as ABA are generated during abiotic stresses, which activate the expression of the transcription factors including MYC; then, the MYC proteins bind to E-box motifs in the *ThbZIP1* promoter to induce its expression. Combined with the stress signals, the activated ThbZIP1 in turn binds to the A-, C- or G-box motifs in the promoters of genes to activate their expression, leading to the improvement of abiotic stress tolerance in plants. Therefore, *ThbZIP1* is a factor in modulating abiotic stress responses through an ABA-dependent signaling pathway.

## Methods

### Plant materials and growth conditions

Seedlings of *T. hispida* were grown in a greenhouse under controlled conditions of 70% relative humidity, light/dark cycles of 14/10 h, and were maintained at 24°C. To induce abiotic stresses, the seedlings were at 4°C or watered on their roots with a solution of 0.4 M NaCl, 20% (w/v) PEG6000, 100 μM ABA or 50 μM MV for 3, 6, 9, 12 and 24 h; the seedlings watered with water were harvested at the corresponding time points as controls.

### Plant transformation

The ORF of *ThbZIP1* was inserted into pROKII driven by CaMV35S promoter, designated as pROKII-ThbZIP1 (see Additional file 8: Table S5 for primers used). The pROKII-ThbZIP1 was transformed into Arabidopsis (ecotype Columbia) using the floral dip method.

### Cloning and activity analysis of the ThbZIP1 promoter

Based on the sequence of *ThbZIP1* (GenBank number: FJ752700), the promoter of *ThbZIP1* (1,571 bp in length) was cloned using a Genome Walking Kit (Takara, Dalian, China). The potential cis-regulatory elements in the promoter were predicted using the software PLACE [18]. The 35S promoter in pCAMBIA1301 was replaced with the *ThbZIP1* promoter that fused with the 5′ UTR of ThbZIP1 to drive the *β-glucuronidase* (*GUS*) gene (*ProThbZIP1::GUS*; Additional file 1: Figure S1A). The *ProThbZIP1::GUS* construct was transferred into Arabidopsis plants by the floral dip method. The T_3_ seedlings were used for promoter activity analysis.

### Analysis of the upstream regulators of ThbZIP1

Previously, eight transcriptomes from roots of *T. hispida* treated with NaHCO3 for 0, 12, 24 and 48 h (two biological replicates were set at each time point) were built [19]. In total, 47,324 unigenes were generated after these transcriptomes *de novo* assembly using SOAPdenovo. The TFs from different families were identified, PCR amplified and cloned into pGADT7-Rec2 (Clontech, Palo Alto, CA, USA) to form a cDNA library (designed as TFs library) for yeast one-hybrid assay. There are seven E-box motifs (“CANNTG”) in the promoter of *ThbZIP1*. Three tandem copies of E-box motif were cloned into a pHIS2 vector (designed as pHIS2-E-box; see Additional file 8: Table S6 for primers used), and were screened with a TFs library for yeast one-hybrid assay (Clontech, Palo Alto, CA, USA). The interactions of pHIS2-p53 (three tandem copies of the cis-acting DNA consensus sequence in pHIS2, which is recognized by p53) with the tested TFs were used as negative controls.

Two *MYC* (*ThMYC4* and *ThMYC6* GenBank number: JN166788 and JN166790) were identified to bind to the E-box motif, of which *ThMYC6* that bound with greater strength to the E-box was used for further study. The E-box core motif of “CANNTG” was mutated to “*AC*AATG”, “CAAA*CA*”, and “*ACCGCA*” (designed as pHIS2-E-M1, -E-M2, -E-M3, respectively; see Additional file 8: Table S6 for primers used). The interactions of *ThMYC6* with E-box motif and its corresponding mutated motifs were studied using the yeast one-hybrid analysis.

To determine if *ThMYC6* is able to activate the expression of *ThbZIP1* by interacting with the E-box motifs, the pHIS2 constructs that harbored the promoter fragments of *ThbZIP1* which contained E-boxes (pHIS2-ProE(+)), or without E-boxes (pHIS2-ProE(-)) were respectively generated as reporter vectors (see Additional file 8: Table S6 for primers used). The interactions of these constructs with *ThMYC6* were studied using yeast one-hybrid analysis.

To further verify these interactions, the three tandem copies of the E-box and its mutant E-M3 (*ACCGCA*) were respectively fused to the minimal 35S promoter (-46 to + 1) to drive GUS, and designed as pCAM-E-box and pCAM-E-M3 (see Additional file 8: Table S7 for primers used). The promoter fragments of *ThbZIP1*, which contained E-box motifs (named as pCAM-E-boxp+), and lacked E-box motifs (pCAM-E-boxp-) (Figure 1C) were respectively fused to the minimal 35S promoter to drive GUS as reporter vectors (see Additional file 8: Table S7 for primers used). The effector vector was constructed by cloning the full ORF of *ThMYC6* into pROKII driven by the 35S promoter (named as pROKII-*ThMYC6*). Both of the reporter vectors and their corresponding effector vectors were co-transformed into tobacco leaves using the particle bombardment. The transformation of pCAMBIA1301 alone (CaMV35S) was used as positive control. The transformation of the reporter plasmids alone or effector plasmids alone was used as negative controls. All assays were repeated three times. GUS histochemical staining assay was performed as described by Jefferson [20], and the GUS stained leaves were scanned by using scanner (D4800,UNISPLENDOUR, China). GUS activity levels were determined according to Jefferson [21].

### Real-time PCR analysis of gene expression

The real-time RT-PCR was performed using *α-tubulin* (XM_002301092) and *actin 3* (XM_002308329) as internal controls (see Additional file 8: Table S8 for primers used). PCR was performed on a MJ Research OpticonTM^2^ instrument with the following conditions: 94°C for 30 s, 45 cycles of 94°C for 12 s, 58°C for 30 s, 72°C for 40 s, and 80°C for 1 s for a plate reading. The relative expression levels of the products were calculated according to the 2^-ΔΔCt^ method [22]. Relative gene expression levels were calculated as the transcription level under stress treatment divided by the transcription level of the controls (i.e., samples from plants grown under normal conditions and harvested at the same treatment time points).

### Assay of ThbZIP1 bindings to C-box, G-box and A-box motifs

Three tandem copies of C-box, G-box and A-box, together with their mutants, CM1: CAAGTG, CM2: CAAAAG, CM3: CACATG and CM4: CACGAG, were cloned into a pHIS2 vector (see Additional file 8: Table S6 for primers used), respectively. Yeast one-hybrid screening analysis was performed to study their interactions with *ThbZIP1*. The yeast cells were grown on selective dropout media: SD/- Trp-Leu/-His (TDO) + 3-AT (3-AT concentration from 30 to 60 mM). Three tandem copies of the C-box, G-box and A-box and the mutant sequence CM2 (CAAAAG) were fused to the minimal 35S promoter (-46 to +1) for driving GUS (constructs containing C-box, G-box, A-box and mutant sequence CM2 named as pCAM-C, pCAM-G, pCAM-A, and pCAM-CM2, respectively). The effector vector was constructed by cloning the ORF of *ThbZIP1* into pROKII driven by the 35S promoter (pROKII-*ThbZIP1*) (see Additional file 8: Table S7 for primers used). Both the reporter and effector vectors were co-transformed into tobacco leaves using particle bombardment. GUS staining and GUS activity assay were determined as above.

### Analysis of ABA, salt and drought stress tolerances

The T_3_ generation of *ThbZIP1* transgenic plants were used in ABA, salt and drought stress tolerance tests. The seeds were sown on MS medium for three days and germinated seeds were transferred into a 1/2 MS medium plus 0, 1, 2, 5, and 10 μM ABA; or 0, 50, 100, 150 and 200 mM NaCl; or 0, 50, 100, 150 and 200 mM Mannitol for two weeks, respectively. The root length and fresh weight were measured. The seeds were sown on a 1/2 MS medium plus 0, 1, 2, 5, or 10 μM ABA, or 0, 50, 100, 150 or 200 mM NaCl, or 0, 50, 100, 150 or 200 mM mannitol for one week, and the germination rates of each transgenic plant or Col-0 plant were measured.

### Microarray experiments and data analysis

The four-week-old seedlings of Col-0 and *ThbZIP1* transgenic plants without treatment or subjected to 150 mM NaCl for 3 h were used for the microarray analyses and three independent biological replications were performed. The Agilent Arabidopsis Oligo microarrays were employed. A Welch’s t-test was used for the parametric test, and the Benjamini and Hochberg false discovery rate for multiple testing corrections was used with a P-value of < 0.05 to filter and identify reliable genes. All genes that were considered to show significant expression level differences by these tests were then filtered by a fold change >2.0. For verification of microarray results, 24 differentially expressed genes identified by microarray were randomly selected for real-time RT-PCR analyses.

### Searching for ThbZIP1-binding sequences in gene promoters

The genes up-regulated by *ThbZIP1* under normal growth or salt stress conditions were randomly selected for ThbZIP1-binding sequences in their promoter regions. The promoter sequences (from -1 to -900) of these genes were derived from TAIR database (http://www.arabidopsis.org/). For identification of ThbZIP1-binding motifs, the sequences of C-, G- and A-box were searched in the promoter regions of these genes.

### Detection of ROS and cell death

Arabidopsis leaves from the two transgenic lines and Col-0 subjected to the ABA, NaCl or Mannitol treatments were infiltrated with 3, 30-diaminobenzidine (DAB) solutions or nitroblue tetrazolium (NBT) following the procedures described by Zhang *et al.*[23]. Cell death was examined by Evans blue staining as described by Kim *et al.*[24]. ROS production in intact guard cells and root tips were detected using 2, 7-dichlorofluorescin diacetate (H_2_DCF-DA) as described by Pei *et al.*[25]*.* H_2_O_2_ levels and GST activity were measured according to Thordal-Christensen *et al.*[26] and Terada *et al.*[27]. Five-day-old seedlings were transferred into MS medium or MS medium with l25 mM NaCl, 10 μM ABA and placed vertically. After stress for 24 h, at least 9 seedlings of each line were incubated with 1 mg/mL PI (Invitrogen) for 20 min. The root tips of stained seedlings were visualized by LSM710 microscope (Zeiss, Jena, Germany) with excitation at 488 nm and emission at 516 nm, respectively.

### Measurement of electrolyte leakage and water loss rates

Electrolyte leakage was measured according to Liu *et al.*[13]. For water loss rates measurements, leaves were detached and weighed immediately (fresh weight, FW), and were then left on the laboratory bench (humidity, 45–50%, 20–22°C) and weighed at designated time intervals (desiccated weights). Leaves were finally oven-dried for 24 h at 80°C to a constant dry weight (DW). Water contents (WC) were measured according to the formula: WC (%) = (desiccated weight – DW)/(FW – DW) × 100.

### Statistical analyses

Statistical analyses were carried out using SPSS 16.0 (SPSSInc, Chicago, III, USA) software. Data were compared using Student’s t-test. Differences were considered to be significant if P < 0.05. ** represented 0.001 < P < 0.01 and * represented 0.01 < P < 0.05.

## Authors’ contributions

YW and GL conceived the project and designed experiments. XJ, YL, LZ, and XN participated in the experiments. XJ performed data analysis. XJ, and YC Wang drafted the manuscript. YW provided reagents and analysis tools. All authors read and approved the final manuscript.

## Supplementary Material

Additional file 1: Figure S1 Promoter activity assay of the ThbZIP1 promoter. A: Schematic map of the *ThbZIP1* promoter inserted into pCAMBIA1301 vector. B: Test of the *ThbZIP1* promoter activity in *Arabidopsis* plants. Seeds (a), three-day-old seedlings (b), five-day-old seedlings (c), one-week-old seedlings (d), ten-day-old seedlings (e), leaf (f), root (g), flower (h), stamen (i), pistil (j, k). Click here for file

Additional file 2: Figure S2 The promoter sequence of ThbZIP1 and analysis of the important cis-elements within the promoter region. The *cis*-elements predicted by PLACE software are shown in different colors. Click here for file

Additional file 3: Table S1 The significantly differentially regulated genes (p < 0.05; ratio > 2 or <0.5) in ThbZIP1 transformed plants relative to Col-0 under normal growth condition. Click here for file

Additional file 4: Table S2 The significantly differentially regulated genes (p < 0.05; ratio > 2 or <0.5) in ThbZIP1 transformed plants relative to Col-0 under salt stress conditions. Click here for file

Additional file 5: Figure S3 Comparison of the results of microarray and real-time RT-PCR. The significantly differentially regulated genes detected by microarray were randomly selected for real-time RT-PCR analysis. Correlation analysis of the results between real-time RT-PCR and cDNA microarray were calculated (P < 0.05). Click here for file

Additional file 6: Table S3 Go analysis of the genes in response to NaCl treatment. Click here for file

Additional file 7: Table S4 The significantly upregulated genes in transgenic plants overexpression of ThbZIP1. Click here for file

Additional file 8 **Primers used in the study. Table S5.** Primer sequences used in construction of the overexpression vectors of ThbZIP1 and ThbZIP1 fused with GFP. **Table S6.** The primer sequences used in the yeast one-hybrid analyses. **Table S7.** The primers used for construction of the reporter vectors. **Table S8.** The primer sequences used in real-time RT-PCR. Click here for file
