# Supplementary material for: The bZIP protein from Tamarix hispida, ThbZIP1, is ACGT elements binding factor that enhances abiotic stress signaling in transgenic Arabidopsis
Source: BMC Plant Biol. 2013 Oct 4;13:151. doi: 10.1186/1471-2229-13-151 (PMC3852707; doi:10.1186/1471-2229-13-151)
Supplement: Additional file 1: Figure S1 — Promoter activity assay of the ThbZIP1 promoter. A: Schematic map of the ThbZIP1 promoter inserted into pCAMBIA1301 vector. B: Test of the ThbZIP1 promoter activity in Arabidopsis plants. Seeds (a), three-day-old seedlings (b), five-day-old seedlings (c), one-week-old seedlings (d), ten-day-old seedlings (e), leaf (f), root (g), flower (h), stamen (i), pistil (j, k). [file 1471-2229-13-151-S1.doc]

**Additional file 1: Figure S1**

**
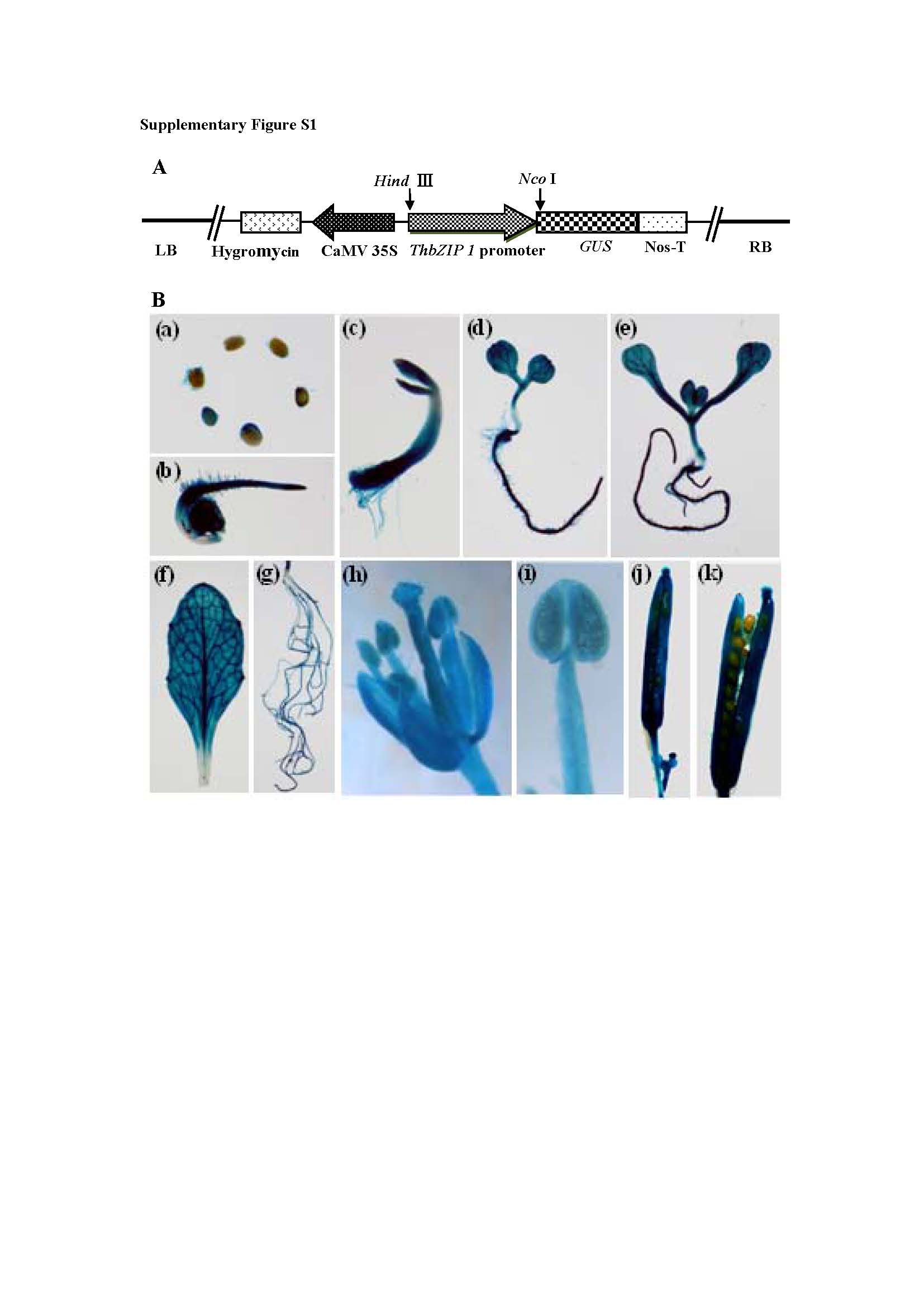
**

**Fig. S1** Promoter activity assay of *ThbZIP1*. A: Schematic map of the *ThbZIP1* promoter inserted into pCAMBIA1301 vector. B: Test of the *ThbZIP1* promoter activity in Arabidopsis plants. Seeds (a), three-day-old seedlings (b), five-day-old seedlings (c), one-week-old seedlings (d), ten-day-old seedlings (e), leave (f), roots (g), flower (h), stamen (i), pistil (j, k).
