# Supplementary material for: The bZIP protein from Tamarix hispida, ThbZIP1, is ACGT elements binding factor that enhances abiotic stress signaling in transgenic Arabidopsis
Source: BMC Plant Biol. 2013 Oct 4;13:151. doi: 10.1186/1471-2229-13-151 (PMC3852707; doi:10.1186/1471-2229-13-151)
Supplement: Additional file 2: Figure S2 — The promoter sequence of ThbZIP1 and analysis of the important cis-elements within the promoter region. The cis-elements predicted by PLACE software are shown in different colors. [file 1471-2229-13-151-S2.doc]

**Additional file 2: Figure S2**


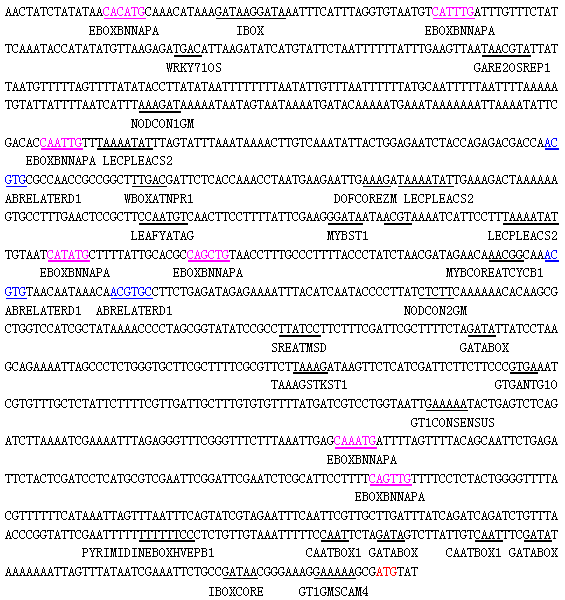


**Fig. S2** The promoter sequence of *ThbZIP1* and analysis of the important cis-elements within the promoter. The *cis*-elements predicted by PLACE software are shown in different colors.
