# Supplementary material for: The bZIP protein from Tamarix hispida, ThbZIP1, is ACGT elements binding factor that enhances abiotic stress signaling in transgenic Arabidopsis
Source: BMC Plant Biol. 2013 Oct 4;13:151. doi: 10.1186/1471-2229-13-151 (PMC3852707; doi:10.1186/1471-2229-13-151)
Supplement: Additional file 3: Table S1 — The significantly differentially regulated genes (p < 0.05; ratio > 2 or <0.5) in ThbZIP1 transformed plants relative to Col-0 under normal growth condition. [file 1471-2229-13-151-S3.doc]

**Additional file 3:** **Table S1 Differential expression genes in the transgenic *ThbZIP1* *Arabidopsis* compared with the Col-0.**

| **ProbeId** | **pvalues** | **foldchange** | **Locustag** | **Description** |
| --- | --- | --- | --- | --- |
| A_84_P803590 | 0.0283 | 25.0335 | AT4G37420 | hypothetical protein |
| A_84_P19584 | 0.0227 | 14.0955 | AT4G15975 | zinc finger (C3HC4-type RING finger) family protein |
| A_84_P18952 | 0.0248 | 13.3273 | AT1G09460 | protein coding |
| A_84_P22673 | 0.0446 | 11.9872 | AT2G40435 | transcription regulator |
| A_84_P17388 | 0.0395 | 11.8949 | AT3G06500 | protein coding |
| A_84_P20669 | 0.0274 | 11.4959 | AT5G49448 | CPuORF4 (Conserved peptide upstream open reading frame 4) |
| A_84_P555310 | 0.0314 | 11.3847 | AT2G36650 | hypothetical protein |
| A_84_P15151 | 0.0287 | 10.138 | AT1G24130 | protein coding |
| A_84_P11606 | 0.0273 | 9.3311 | AT2G29480 | ATGSTU2 (GLUTATHIONE S-TRANSFERASE 20); glutathione transferase |
| A_84_P803062 | 0.046 | 9.3083 | AT3G30775 | protein coding |
| A_84_P83299 | 0.0325 | 8.5474 | AT2G45330 | EMB1067 (EMBRYO DEFECTIVE 1067); tRNA 2'-phosphotransferase |
| A_84_P21800 | 0.028 | 7.9757 | AT1G70290 | protein coding |
| A_84_P14435 | 0.0159 | 7.0226 | AT2G26710 | BAS1/CYP734A1 (PHYB ACTIVATION TAGGED SUPPRESSOR 1); oxygen binding / steroid hydroxylase |
| A_84_P18113 | 0.0073 | 6.0443 | AT1G76640 | protein coding |
| A_84_P610481 | 0.0388 | 5.9947 | AT2G34600 | JAZ7/TIFY5B (JASMONATE-ZIM-DOMAIN PROTEIN 7) |
| A_84_P18998 | 0.0317 | 5.9133 | AT1G72900 | protein coding |
| A_84_P704033 | 0.0316 | 5.8438 | AT1G11185 | misc_RNA |
| A_84_P278280 | 0.0348 | 5.6107 | AT2G39570 | ACT domain-containing protein |
| A_84_P809927 | 0.0052 | 5.5172 | AT5G07440 | GDH2 (GLUTAMATE DEHYDROGENASE 2); oxidoreductase |
| A_84_P12314 | 0.0155 | 5.5097 | AT1G23200 | protein coding |
| A_84_P17690 | 0.0421 | 5.467 | AT4G15330 | CYP705A1 (cytochrome P450, family 705, subfamily A, polypeptide 1); oxygen binding |
| A_84_P12178 | 0.0474 | 5.3812 | AT1G56120 | protein coding |
| A_84_P223589 | 0.0317 | 5.2907 | AT4G16000 | hypothetical protein |
| A_84_P14985 | 0.0473 | 5.2392 | AT5G47220 | ATERF-2/ATERF2/ERF2 (ETHYLENE RESPONSE FACTOR 2); DNA binding / transcription activator/ transcripti |
| A_84_P16900 | 0.0171 | 5.0984 | AT5G53820 | hypothetical protein |
| A_84_P23977 | 0.0301 | 4.9673 | AT3G09010 | protein coding |
| A_84_P18315 | 0.0271 | 4.9554 | AT3G09940 | protein coding |
| A_84_P563122 | 0.0047 | 4.921 | AT1G61160 | protein coding |
| A_84_P816700 | 0.0434 | 4.6459 | AT1G17745 | protein coding |
| A_84_P11903 | 0.0338 | 4.6055 | AT1G06160 | protein coding |
| A_84_P12126 | 0.0181 | 4.5677 | AT5G40540 | protein kinase, putative |
| A_84_P811911 | 0.0449 | 4.5265 | AT5G66400 | RAB18 (RESPONSIVE TO ABA 18) |
| A_84_P11580 | 0.0182 | 4.4323 | AT2G47870 | glutaredoxin family protein |
| A_84_P15515 | 0.0326 | 4.3304 | AT3G19390 | protein coding |
| A_84_P594753 | 0.0324 | 4.252 | AT4G22230 | hypothetical protein |
| A_84_P818265 | 0.0325 | 4.1533 | AT4G32480 | hypothetical protein |
| A_84_P848773 | 0.0388 | 4.0695 | AT4G15393 | CYP702A5 (cytochrome P450, family 702, subfamily A, polypeptide 5); heme binding / iron ion binding |
| A_84_P815732 | 0.022 | 4.0455 | AT1G08980 | protein coding |
| A_84_P132245 | 0.0133 | 3.9487 | AT5G50940 | pseudo |
| A_84_P10206 | 0.03 | 3.8264 | AT5G25160 | ZFP3 (ZINC FINGER PROTEIN 3); nucleic acid binding / transcription factor/ zinc ion binding |
| A_84_P235233 | 0.0457 | 3.7461 | AT4G36610 | hydrolase, alpha/beta fold family protein |
| A_84_P544827 | 0.0035 | 3.7383 | AT3G25760 | protein coding |
| A_84_P768715 | 0.0162 | 3.7163 | AT5G15265 | hypothetical protein |
| A_84_P279270 | 0.0038 | 3.6355 | AT4G03500 | ankyrin repeat family protein |
| A_84_P814134 | 0.0484 | 3.6314 | AT1G19660 | protein coding |
| A_84_P23975 | 0.0488 | 3.6235 | AT3G01080 | protein coding |
| A_84_P92139 | 0.0301 | 3.6151 | AT4G37700 | hypothetical protein |
| A_84_P18158 | 0.0161 | 3.6117 | AT1G57850 | protein coding |
| A_84_P212878 | 0.0254 | 3.5669 | AT2G45930 | hypothetical protein |
| A_84_P10038 | 0.0498 | 3.5407 | AT4G20050 | QRT3 (QUARTET 3) |
| A_84_P71564 | 0.0184 | 3.5125 | AT2G19800 | MIOX2 (MYO-INOSITOL OXYGENASE 2) |
| A_84_P757973 | 0.0015 | 3.504 | AT2G44578 | protein binding / zinc ion binding |
| A_84_P565178 | 0.0141 | 3.4921 | AT3G25882 | protein coding |
| A_84_P11239 | 0.0285 | 3.4779 | AT5G56840 | DNA-binding family protein |
| A_84_P11659 | 0.0497 | 3.4517 | AT2G39400 | hydrolase, alpha/beta fold family protein |
| A_84_P14348 | 0.0066 | 3.4415 | AT1G13700 | protein coding |
| A_84_P22951 | 0.0042 | 3.4213 | AT2G27010 | CYP705A9 (cytochrome P450, family 705, subfamily A, polypeptide 9); oxygen binding |
| A_84_P18388 | 0.0126 | 3.3916 | AT3G26330 | protein coding |
| A_84_P215028 | 0.0114 | 3.3871 | AT3G25770 | protein coding |
| A_84_P169523 | 0.0472 | 3.3833 | AT5G03020 | kelch repeat-containing F-box family protein |
| A_84_P849345 | 0.0371 | 3.3526 | AT3G24740 | protein coding |
| A_84_P198764 | 0.0377 | 3.3171 | AT3G49790 | protein coding |
| A_84_P849451 | 0.0403 | 3.2889 | AT3G26910 | protein coding |
| A_84_P241449 | 0.0375 | 3.2554 | AT1G28190 | protein coding |
| A_84_P740321 | 0.0218 | 3.2058 | AT3G44550 | protein coding |
| A_84_P92059 | 0.0439 | 3.1873 | AT5G67480 | BT4 (BTB AND TAZ DOMAIN PROTEIN 4); protein binding / transcription regulator |
| A_84_P804662 | 0.0271 | 3.1764 | AT4G27780 | ACBP2 (ACYL-COA BINDING PROTEIN ACBP 2) |
| A_84_P225559 | 0.0336 | 3.146 | AT5G64810 | WRKY51 (WRKY DNA-binding protein 51); transcription factor |
| A_84_P23166 | 0.0388 | 3.1108 | AT3G50700 | protein coding |
| A_84_P12352 | 0.0285 | 3.1027 | AT1G02190 | protein coding |
| A_84_P267120 | 0.0099 | 3.102 | AT1G74950 | protein coding |
| A_84_P558681 | 7.00E-04 | 3.0806 | AT1G53903 | protein coding |
| A_84_P13096 | 0.0204 | 3.047 | AT5G47370 | HAT2; transcription factor |
| A_84_P210768 | 0.0207 | 3.0347 | AT5G19230 | hypothetical protein |
| A_84_P182664 | 0.0089 | 3.0315 | AT2G02710 | PAC motif-containing protein |
| A_84_P274410 | 0.047 | 3.0211 | AT1G68330 | protein coding |
| A_84_P811894 | 0.0477 | 3.0159 | AT4G08950 | phosphate-responsive protein, putative (EXO) |
| A_84_P17694 | 0.0179 | 3.0026 | AT4G16520 | ATG8F (AUTOPHAGY 8F); microtubule binding |
| A_84_P561083 | 0.0343 | 2.9854 | AT5G42930 | triacylglycerol lipase |
| A_84_P818520 | 0.0404 | 2.9705 | AT5G06370 | NC domain-containing protein |
| A_84_P761749 | 0.0015 | 2.9535 | AT3G22275 | protein coding |
| A_84_P523657 | 0.0036 | 2.9413 | AT4G12545 | protease inhibitor/seed storage/lipid transfer protein (LTP) family protein |
| A_84_P14226 | 0.0437 | 2.928 | AT1G10070 | protein coding |
| A_84_P764106 | 0.0273 | 2.9186 | AT4G27740 | hypothetical protein |
| A_84_P825052 | 0.0451 | 2.913 | AT1G53170 | protein coding |
| A_84_P19313 | 0.0148 | 2.9129 | AT3G26160 | protein coding |
| A_84_P788668 | 0.0463 | 2.9068 | AT2G04690 | cellular repressor of E1A-stimulated genes (CREG) family |
| A_84_P815139 | 0.0038 | 2.8916 | AT5G61210 | SNAP33 (synaptosomal-associated protein 33); SNAP receptor |
| A_84_P14585 | 0.0291 | 2.8782 | AT3G16800 | protein coding |
| A_84_P17984 | 0.0341 | 2.8525 | AT1G60730 | protein coding |
| A_84_P159575 | 0.0011 | 2.8488 | AT4G15765 | monooxygenase family protein |
| A_84_P724318 | 0.0086 | 2.8374 | AT5G06865 | miscRNA |
| A_84_P169283 | 0.0079 | 2.8241 | AT5G06570 | hydrolase |
| A_84_P600236 | 0.0122 | 2.7879 | AT5G22545 | hypothetical protein |
| A_84_P13746 | 0.042 | 2.7821 | AT3G59350 | protein coding |
| A_84_P752539 | 0.0125 | 2.7688 | AT1G08920 | protein coding |
| A_84_P13573 | 0.0308 | 2.7664 | AT3G07650 | protein coding |
| A_84_P11360 | 0.0486 | 2.762 | AT1G52040 | protein coding |
| A_84_P164883 | 0.0353 | 2.7615 | AT5G42900 | hypothetical protein |
| A_84_P823526 | 0.0308 | 2.7402 | AT5G04250 | OTU-like cysteine protease family protein |
| A_84_P15229 | 0.0033 | 2.7254 | AT1G79650 | protein coding |
| A_84_P175641 | 0.0204 | 2.719 | AT3G19660 | protein coding |
| A_84_P11410 | 0.0308 | 2.7137 | AT1G04370 | protein coding |
| A_84_P579358 | 0.0436 | 2.7084 | AT3G01516 | protein coding |
| A_84_P11822 | 0.0239 | 2.7038 | AT3G51450 | protein coding |
| A_84_P531612 | 0.0022 | 2.7026 | AT4G04745 | hypothetical protein |
| A_84_P20203 | 0.0374 | 2.6961 | AT3G01770 | protein coding |
| A_84_P821845 | 0.0117 | 2.6909 | AT3G15510 | protein coding |
| A_84_P17642 | 0.0452 | 2.6846 | AT4G29270 | acid phosphatase class B family protein |
| A_84_P507030 | 0.0485 | 2.6728 | AT5G49525 | hypothetical protein |
| A_84_P14678 | 0.0488 | 2.6614 | AT3G56000 | protein coding |
| A_84_P23069 | 0.0486 | 2.6588 | AT3G17690 | protein coding |
| A_84_P52340 | 0.029 | 2.651 | AT3G26840 | protein coding |
| A_84_P853555 | 0.0253 | 2.6236 | AT1G18020 | protein coding |
| A_84_P594594 | 0.0052 | 2.6174 | AT3G22415 | protein coding |
| A_84_P10178 | 0.0086 | 2.6092 | AT5G12050 | hypothetical protein |
| A_84_P21194 | 0.0478 | 2.6085 | AT3G23240 | protein coding |
| A_84_P22095 | 0.0347 | 2.6056 | AT1G01240 | protein coding |
| A_84_P18708 | 0.0355 | 2.601 | AT5G23050 | acyl-activating enzyme 17 (AAE17) |
| A_84_P10737 | 0.0372 | 2.5838 | AT3G11410 | protein coding |
| A_84_P23445 | 0.0411 | 2.5816 | AT5G25140 | CYP71B13 (cytochrome P450, family 71, subfamily B, polypeptide 13); oxygen binding |
| A_84_P854997 | 0.047 | 2.5815 | AT1G01180 | protein coding |
| A_84_P764120 | 0.0209 | 2.5795 | AT4G21830 | methionine sulfoxide reductase domain-containing protein / SeIR domain-containing protein |
| A_84_P67354 | 0.0484 | 2.5616 | AT5G63580 | flavonol synthase, putative |
| A_84_P18949 | 0.0476 | 2.5544 | AT1G04610 | protein coding |
| A_84_P858196 | 0.0118 | 2.5536 | AT1G72820 | protein coding |
| A_84_P60310 | 0.0246 | 2.5446 | AT5G05850 | leucine-rich repeat family protein |
| A_84_P10861 | 0.0432 | 2.542 | AT3G47790 | protein coding |
| A_84_P825243 | 0.0367 | 2.5341 | AT2G38820 | hypothetical protein |
| A_84_P312153 | 0.0425 | 2.5228 | AT3G11430 | protein coding |
| A_84_P240045 | 0.0271 | 2.5189 | AT5G22270 | hypothetical protein |
| A_84_P54250 | 0.0402 | 2.5057 | AT1G19960 | protein coding |
| A_84_P11789 | 0.0345 | 2.5024 | AT3G43250 | protein coding |
| A_84_P297634 | 0.0433 | 2.4991 | AT5G15500 | ankyrin repeat family protein |
| A_84_P790017 | 0.0069 | 2.4981 | AT2G46320 | mitochondrial substrate carrier family protein |
| A_84_P562314 | 0.001 | 2.4922 | AT2G04080 | MATE efflux family protein |
| A_84_P15710 | 0.0426 | 2.477 | AT4G20110 | vacuolar sorting receptor, putative |
| A_84_P837016 | 0.0094 | 2.4766 | AT1G04310 | protein coding |
| A_84_P604788 | 0.0057 | 2.4694 | AT5G59510 | DVL18/RTFL5 (ROTUNDIFOLIA LIKE 5) |
| A_84_P286390 | 0.0309 | 2.4676 | AT3G25780 | protein coding |
| A_84_P848467 | 0.0498 | 2.4601 | AT1G36070 | protein coding |
| A_84_P13850 | 0.0175 | 2.4515 | AT4G27260 | GH3.5/WES1; indole-3-acetic acid amido synthetase |
| A_84_P573310 | 0.0305 | 2.4451 | AT4G01330 | protein kinase family protein |
| A_84_P12544 | 0.0448 | 2.4278 | AT2G26750 | epoxide hydrolase, putative |
| A_84_P274610 | 0.0378 | 2.4178 | AT2G39000 | GCN5-related N-acetyltransferase (GNAT) family protein |
| A_84_P858843 | 0.047 | 2.4164 | AT1G12440 | protein coding |
| A_84_P18072 | 0.0481 | 2.4141 | AT1G72520 | protein coding |
| A_84_P21894 | 0.028 | 2.4129 | AT1G68740 | protein coding |
| A_84_P17216 | 0.0267 | 2.4113 | AT1G30220 | protein coding |
| A_84_P197444 | 0.0171 | 2.4095 | AT3G22160 | protein coding |
| A_84_P13549 | 0.0037 | 2.4047 | AT2G39420 | esterase/lipase/thioesterase family protein |
| A_84_P185254 | 0.0367 | 2.3997 | AT5G45310 | hypothetical protein |
| A_84_P17629 | 0.0163 | 2.3975 | AT4G26400 | zinc finger (C3HC4-type RING finger) family protein |
| A_84_P854273 | 0.0257 | 2.3928 | AT3G51370 | protein coding |
| A_84_P512586 | 0.0205 | 2.3834 | AT1G53785 | protein coding |
| A_84_P15371 | 0.0191 | 2.3794 | AT2G23290 | AtMYB70 (myb domain protein 70); DNA binding / transcription factor |
| A_84_P20213 | 0.0483 | 2.3781 | AT3G04000 | protein coding |
| A_84_P10447 | 0.0449 | 2.3727 | AT1G77210 | protein coding |
| A_84_P837674 | 0.0045 | 2.372 | AT5G05160 | leucine-rich repeat transmembrane protein kinase, putative |
| A_84_P800117 | 0.0402 | 2.3718 | AT4G06536 | hypothetical protein |
| A_84_P821393 | 0.001 | 2.3456 | AT1G17160 | protein coding |
| A_84_P823935 | 0.0065 | 2.3446 | AT4G10000 | hypothetical protein |
| A_84_P521598 | 0.0334 | 2.3363 | AT2G22760 | basic helix-loop-helix (bHLH) family protein |
| A_84_P860746 | 0.0357 | 2.3294 | AT3G47510 | protein coding |
| A_84_P756184 | 0.002 | 2.329 | AT2G04066 | MATE efflux protein-related |
| A_84_P855337 | 0.0082 | 2.3132 | AT2G18280 | AtTLP2 (TUBBY LIKE PROTEIN 2); phosphoric diester hydrolase/ transcription factor |
| A_84_P838159 | 0.0476 | 2.3121 | AT3G22560 | protein coding |
| A_84_P287970 | 0.0483 | 2.3084 | AT5G65690 | PCK2/PEPCK (PHOSPHOENOLPYRUVATE CARBOXYKINASE 2); ATP binding / phosphoenolpyruvate carboxykinase (A |
| A_84_P11073 | 0.0203 | 2.3051 | AT1G19300 | protein coding |
| A_84_P598498 | 0.0286 | 2.3009 | AT5G41120 | esterase/lipase/thioesterase family protein |
| A_84_P201098 | 0.0039 | 2.2989 | AT4G31820 | ENP (ENHANCER OF PINOID); signal transducer |
| A_84_P166223 | 0.0442 | 2.2939 | AT2G35230 | VQ motif-containing protein |
| A_84_P22682 | 0.0119 | 2.2671 | AT3G06483 | protein coding |
| A_84_P786570 | 0.0428 | 2.2661 | AT2G22860 | ATPSK2 (PHYTOSULFOKINE 2 PRECURSOR); growth factor |
| A_84_P816158 | 0.0064 | 2.2514 | AT1G18030 | protein coding |
| A_84_P544235 | 0.0426 | 2.244 | AT5G28237 | tryptophan synthase, beta subunit, putative |
| A_84_P283660 | 0.0472 | 2.2405 | AT2G42040 | hypothetical protein |
| A_84_P224949 | 0.0409 | 2.217 | AT3G52105 | protein coding |
| A_84_P10028 | 0.0184 | 2.2144 | AT1G17530 | protein coding |
| A_84_P94069 | 0.0079 | 2.2136 | AT5G53160 | hypothetical protein |
| A_84_P762636 | 0.0219 | 2.2095 | AT3G22961 | protein coding |
| A_84_P812141 | 0.0065 | 2.2078 | AT3G16400 | protein coding |
| A_84_P772475 | 0.0442 | 2.2047 | AT1G66760 | protein coding |
| A_84_P853045 | 0.0361 | 2.2035 | AT4G20380 | LSD1 (LESION SIMULATING DISEASE) |
| A_84_P12533 | 0.0385 | 2.2025 | AT1G11180 | protein coding |
| A_84_P856876 | 0.0494 | 2.1992 | AT1G73330 | protein coding |
| A_84_P122532 | 0.0299 | 2.1984 | AT5G67210 | hypothetical protein |
| A_84_P11888 | 0.0273 | 2.1929 | AT4G03030 | kelch repeat-containing F-box family protein |
| A_84_P23814 | 0.044 | 2.1919 | AT1G20030 | protein coding |
| A_84_P280890 | 0.0285 | 2.1899 | AT3G10320 | protein coding |
| A_84_P869814 | 0.0295 | 2.1805 | AT4G05330 | AGD13 (ARF-GAP DOMAIN 13); ARF GTPase activator/ zinc ion binding |
| A_84_P591730 | 0.0317 | 2.1788 | AT2G06255 | ELF4-L3 (ELF4-LIKE 3) |
| A_84_P587454 | 0.0374 | 2.1725 | AT3G19850 | protein coding |
| A_84_P711353 | 0.02 | 2.1643 | AT2G04560 | transferase, transferring glycosyl groups |
| A_84_P753864 | 0.0419 | 2.1639 | AT1G67365 | misc_RNA |
| A_84_P20256 | 0.0474 | 2.156 | AT3G12820 | protein coding |
| A_84_P595864 | 0.0317 | 2.1528 | AT5G54730 | AtATG18f (Arabidopsis thaliana homolog of yeast autophagy 18 (ATG18) f) |
| A_84_P83309 | 0.0499 | 2.1463 | AT3G07000 | protein coding |
| A_84_P254110 | 0.0227 | 2.1446 | AT3G20300 | protein coding |
| A_84_P19135 | 0.0253 | 2.1334 | AT2G28630 | beta-ketoacyl-CoA synthase family protein |
| A_84_P842737 | 0.0376 | 2.1282 | AT3G29330 | protein coding |
| A_84_P11796 | 0.0352 | 2.1259 | AT3G45590 | protein coding |
| A_84_P796867 | 0.0162 | 2.1178 | AT1G21510 | protein coding |
| A_84_P830682 | 0.0396 | 2.1137 | AT1G07745 | protein coding |
| A_84_P599444 | 0.0173 | 2.1024 | AT5G57565 | protein kinase family protein |
| A_84_P21062 | 0.0143 | 2.1004 | AT2G29400 | TOPP1 (TYPE ONE PROTEIN PHOSPHATASE 1); protein serine/threonine phosphatase |
| A_84_P206058 | 0.0438 | 2.0932 | AT4G37290 | hypothetical protein |
| A_84_P848148 | 0.0312 | 2.0912 | AT1G22710 | protein coding |
| A_84_P750661 | 0.0329 | 2.0904 | AT1G07170 | protein coding |
| A_84_P822194 | 0.0257 | 2.0892 | AT1G55510 | protein coding |
| A_84_P869124 | 0.0053 | 2.0829 | AT3G48530 | protein coding |
| A_84_P18557 | 0.0361 | 2.0827 | AT4G22470 | protease inhibitor/seed storage/lipid transfer protein (LTP) family protein |
| A_84_P244235 | 0.003 | 2.0825 | AT3G20810 | protein coding |
| A_84_P23859 | 0.0197 | 2.0799 | AT2G30250 | WRKY25 (WRKY DNA-binding protein 25); transcription factor |
| A_84_P10155 | 0.037 | 2.0797 | AT5G05750 | DNAJ heat shock N-terminal domain-containing protein |
| A_84_P836480 | 0.0166 | 2.0707 | AT1G09280 | protein coding |
| A_84_P849029 | 0.0208 | 2.0694 | AT1G01780 | protein coding |
| A_84_P21629 | 0.0376 | 2.068 | AT5G53550 | YSL3 (YELLOW STRIPE LIKE 3); oligopeptide transporter |
| A_84_P791189 | 0.0453 | 2.0629 | AT3G27906 | protein coding |
| A_84_P101436 | 0.0049 | 2.061 | AT5G54067 | hypothetical protein |
| A_84_P17442 | 0.027 | 2.0588 | AT3G26320 | protein coding |
| A_84_P291394 | 0.0463 | 2.0586 | AT2G41370 | BOP2 (BLADE ON PETIOLE2); protein binding |
| A_84_P598951 | 0.0192 | 2.0578 | AT2G40520 | nucleotidyltransferase family protein |
| A_84_P18215 | 0.0238 | 2.0572 | AT2G32290 | BAM6/BMY5 (BETA-AMYLASE 6); beta-amylase |
| A_84_P22141 | 0.0349 | 2.0555 | AT3G16910 | protein coding |
| A_84_P792605 | 0.0448 | 2.0495 | AT3G61310 | protein coding |
| A_84_P11227 | 0.0065 | 2.0479 | AT5G53400 | nuclear movement family protein |
| A_84_P221816 | 0.0291 | 2.0424 | AT2G21860 | violaxanthin de-epoxidase-related |
| A_84_P823879 | 0.0423 | 2.0371 | AT5G47240 | ATNUDT8 (Arabidopsis thaliana Nudix hydrolase homolog 8); hydrolase |
| A_84_P23014 | 0.0079 | 2.0357 | AT2G44581 | protein binding / zinc ion binding |
| A_84_P21763 | 0.0377 | 2.0356 | AT1G61580 | protein coding |
| A_84_P581411 | 0.0359 | 2.0294 | AT4G32295 | hypothetical protein |
| A_84_P88039 | 0.0433 | 2.0294 | AT5G15910 | dehydrogenase-related |
| A_84_P18471 | 0.0309 | 2.0277 | AT3G58660 | protein coding |
| A_84_P245905 | 0.0221 | 2.0158 | AT3G13230 | protein coding |
| A_84_P808808 | 0.0322 | 2.0154 | AT2G24270 | ALDH11A3 (Aldehyde dehydrogenase 11A3); 3-chloroallyl aldehyde dehydrogenase/ glyceraldehyde-3-phosp |
| A_84_P20851 | 0.0345 | 2.0086 | AT1G75640 | protein coding |
| A_84_P10352 | 0.0179 | 2.0048 | AT1G27910 | protein coding |
| A_84_P206238 | 0.0035 | 2.0027 | AT5G22060 | ATJ2 (Arabidopsis thaliana DnaJ homologue 2) |
| A_84_P21705 | 0.034 | 2.0022 | AT5G28520 | hypothetical protein |
| A_84_P784673 | 0.029 | 2.002 | AT5G57620 | MYB36 (myb domain protein 36); DNA binding / transcription factor |
| A_84_P291034 | 0.0035 | 2.0015 | AT5G09500 | 40S ribosomal protein S15 (RPS15C) |
| A_84_P758710 | 0.0252 | 0.4995 | AT2G15050 | LTP; lipid binding |
| A_84_P22067 | 0.0323 | 0.4994 | AT2G01210 | leucine-rich repeat transmembrane protein kinase, putative |
| A_84_P825582 | 0.0323 | 0.4989 | AT2G41040 | methyltransferase-related |
| A_84_P14218 | 0.0138 | 0.4981 | AT1G65610 | protein coding |
| A_84_P869400 | 0.022 | 0.4975 | AT3G29035 | protein coding |
| A_84_P20798 | 0.0179 | 0.4965 | AT1G01610 | protein coding |
| A_84_P575735 | 0.0125 | 0.4965 | AT2G34350 | nodulin-related |
| A_84_P19158 | 0.0461 | 0.496 | AT2G41290 | strictosidine synthase family protein |
| A_84_P10924 | 0.0334 | 0.4943 | AT3G62740 | protein coding |
| A_84_P835475 | 0.01 | 0.4942 | AT4G17750 | HSF1 (ARABIDOPSIS HEAT SHOCK FACTOR 1); DNA binding / transcription factor |
| A_84_P21609 | 0.0115 | 0.4928 | AT1G56170 | protein coding |
| A_84_P11543 | 0.0206 | 0.4927 | AT1G27880 | protein coding |
| A_84_P21370 | 0.0146 | 0.4927 | AT4G12320 | CYP706A6 (cytochrome P450, family 706, subfamily A, polypeptide 6); oxygen binding |
| A_84_P14983 | 0.0186 | 0.4919 | AT5G46740 | UBP21 (UBIQUITIN-SPECIFIC PROTEASE 21); ubiquitin-specific protease |
| A_84_P20212 | 0.0347 | 0.4918 | AT3G04710 | protein coding |
| A_84_P204458 | 0.0189 | 0.4912 | AT2G16070 | PDV2 (PLASTID DIVISION2) |
| A_84_P12222 | 0.0121 | 0.4881 | AT5G65860 | ankyrin repeat family protein |
| A_84_P12357 | 0.0411 | 0.4879 | AT1G10480 | protein coding |
| A_84_P14811 | 0.0393 | 0.4874 | AT4G30680 | MA3 domain-containing protein |
| A_84_P10998 | 0.0067 | 0.487 | AT4G23570 | SGT1A (Suppressor of G2 (Two) 1A); protein binding |
| A_84_P543741 | 0.0446 | 0.487 | AT2G18220 | hypothetical protein |
| A_84_P850500 | 0.0451 | 0.4861 | AT1G78580 | protein coding |
| A_84_P834927 | 0.0454 | 0.4856 | AT1G63850 | protein coding |
| A_84_P68964 | 0.0251 | 0.4847 | AT5G62700 | TUB3 (TUBULIN BETA-3); structural molecule |
| A_84_P805556 | 0.0099 | 0.4844 | AT3G44110 | protein coding |
| A_84_P504684 | 0.0185 | 0.4843 | AT1G78610 | protein coding |
| A_84_P223109 | 0.0192 | 0.4839 | AT2G46420 | hypothetical protein |
| A_84_P753050 | 0.015 | 0.4824 | AT1G18835 | protein coding |
| A_84_P16879 | 0.0463 | 0.4822 | AT5G47800 | phototropic-responsive NPH3 family protein |
| A_84_P288654 | 0.0333 | 0.482 | AT1G22530 | protein coding |
| A_84_P21809 | 0.0146 | 0.4815 | AT1G60030 | protein coding |
| A_84_P10225 | 0.0456 | 0.481 | AT5G37960 | oxidoreductase-related |
| A_84_P808919 | 0.0301 | 0.4785 | AT2G45960 | PIP1B (plasma membrane intrinsic protein 1;2) |
| A_84_P854985 | 0.0383 | 0.4771 | AT4G13840 | transferase family protein |
| A_84_P558846 | 0.0233 | 0.4768 | AT2G35130 | pentatricopeptide (PPR) repeat-containing protein |
| A_84_P805060 | 0.0273 | 0.4766 | AT5G61020 | ECT3 (evolutionary conserved C-terminal 3) |
| A_84_P20265 | 0.0347 | 0.4751 | AT3G15090 | protein coding |
| A_84_P13004 | 0.0292 | 0.4741 | AT5G09730 | BXL3 (BETA-XYLOSIDASE 3); hydrolase, hydrolyzing O-glycosyl compounds |
| A_84_P195914 | 0.0402 | 0.4736 | AT3G47030 | protein coding |
| A_84_P843476 | 0.0305 | 0.4721 | AT2G20190 | ATCLASP/CLASP; binding |
| A_84_P16735 | 0.0085 | 0.4709 | AT4G39070 | zinc finger (B-box type) family protein |
| A_84_P715005 | 0.0486 | 0.4675 | AT5G52060 | ATBAG1 (ARABIDOPSIS THALIANA BCL-2-ASSOCIATED ATHANOGENE 1); protein binding |
| A_84_P16575 | 0.0456 | 0.4674 | AT3G57490 | protein coding |
| A_84_P846184 | 0.0457 | 0.4671 | AT1G11580 | protein coding |
| A_84_P10347 | 0.0055 | 0.4669 | AT5G17600 | zinc finger (C3HC4-type RING finger) family protein |
| A_84_P12750 | 0.0464 | 0.4665 | AT3G47430 | protein coding |
| A_84_P606611 | 0.0258 | 0.4659 | AT2G43500 | RWP-RK domain-containing protein |
| A_84_P15752 | 0.0074 | 0.4649 | AT4G29690 | type I phosphodiesterase/nucleotide pyrophosphatase family protein |
| A_84_P12089 | 0.0167 | 0.4628 | AT5G22980 | SCPL47 (serine carboxypeptidase-like 47); serine carboxypeptidase |
| A_84_P18118 | 0.0036 | 0.4618 | AT1G56430 | protein coding |
| A_84_P20938 | 0.0112 | 0.4583 | AT1G69880 | protein coding |
| A_84_P860623 | 0.013 | 0.4571 | AT5G17310 | UTP--glucose-1-phosphate uridylyltransferase, putative / UDP-glucose pyrophosphorylase, putative / U |
| A_84_P576295 | 0.0123 | 0.457 | AT1G03106 | protein coding |
| A_84_P14723 | 0.0392 | 0.4564 | AT4G03070 | AOP1 (2-oxoglutarate?dependent dioxygenase 1.1); oxidoreductase, acting on paired donors, with incor |
| A_84_P18240 | 0.0013 | 0.4552 | AT2G19310 | hypothetical protein |
| A_84_P22409 | 0.0119 | 0.4539 | AT4G38710 | glycine-rich protein |
| A_84_P826191 | 0.0336 | 0.453 | AT5G44800 | CHR4/MI-2-LIKE (chromatin remodeling 4); ATP binding / DNA binding / chromatin binding / helicase |
| A_84_P834657 | 0.0301 | 0.4528 | AT1G05830 | protein coding |
| A_84_P175924 | 0.0404 | 0.4506 | AT2G44745 | WRKY family transcription factor |
| A_84_P13530 | 0.0408 | 0.4457 | AT2G24190 | short-chain dehydrogenase/reductase (SDR) family protein |
| A_84_P16133 | 0.0155 | 0.4436 | AT1G79310 | protein coding |
| A_84_P815408 | 0.022 | 0.4432 | AT2G04030 | CR88 (EMBRYO DEFECTIVE 1956); ATP binding |
| A_84_P10969 | 0.0317 | 0.4422 | AT4G12390 | PME1; pectinesterase inhibitor |
| A_84_P21844 | 0.0061 | 0.4415 | AT1G11520 | protein coding |
| A_84_P534067 | 0.0255 | 0.4405 | AT2G38640 | hypothetical protein |
| A_84_P216708 | 0.0281 | 0.4401 | AT2G42975 | hypothetical protein |
| A_84_P22433 | 0.0411 | 0.44 | AT5G01260 | glycoside hydrolase starch-binding domain-containing protein |
| A_84_P11942 | 0.0428 | 0.4388 | AT4G23320 | protein kinase family protein |
| A_84_P126931 | 0.022 | 0.4372 | AT2G01505 | CLE16 (CLAVATA3/ESR-RELATED 16); receptor binding |
| A_84_P802894 | 0.0414 | 0.4368 | AT2G26080 | ATGLDP2 (ARABIDOPSIS THALIANA GLYCINE DECARBOXYLASE P-PROTEIN 2); glycine dehydrogenase (decarboxyla |
| A_84_P258870 | 0.0226 | 0.4346 | AT1G72130 | protein coding |
| A_84_P20772 | 0.0382 | 0.4343 | AT5G04370 | NAMT1 |
| A_84_P787041 | 0.0209 | 0.4341 | AT5G66820 | hypothetical protein |
| A_84_P14746 | 0.0102 | 0.4341 | AT4G11660 | AT-HSFB2B (Arabidopsis thaliana heat shock transcription factor B2B); transcription factor |
| A_84_P248025 | 0.0495 | 0.4283 | AT2G42800 | leucine-rich repeat family protein |
| A_84_P113792 | 0.0462 | 0.4258 | AT3G03770 | protein coding |
| A_84_P857535 | 0.0043 | 0.4254 | AT3G44326 | protein coding |
| A_84_P204138 | 0.0164 | 0.4253 | AT1G66080 | protein coding |
| A_84_P12309 | 0.0251 | 0.4242 | AT1G67360 | protein coding |
| A_84_P249845 | 0.0044 | 0.4234 | AT3G46870 | protein coding |
| A_84_P519178 | 0.0453 | 0.4216 | AT3G63530 | protein coding |
| A_84_P808508 | 0.0441 | 0.4207 | AT1G29970 | protein coding |
| A_84_P162193 | 0.03 | 0.4204 | AT5G62070 | IQD23 (IQ-domain 23); calmodulin binding |
| A_84_P790566 | 0.0386 | 0.4202 | AT1G69900 | protein coding |
| A_84_P722142 | 0.0099 | 0.4195 | AT2G43375 | misc_RNA |
| A_84_P15742 | 0.0382 | 0.4182 | AT4G27450 | hypothetical protein |
| A_84_P20361 | 0.0443 | 0.4179 | AT3G57920 | protein coding |
| A_84_P541541 | 0.0065 | 0.4173 | AT5G20640 | hypothetical protein |
| A_84_P588557 | 0.0453 | 0.416 | AT4G36830 | GNS1/SUR4 membrane family protein |
| A_84_P808697 | 0.0323 | 0.4152 | AT2G05100 | LHCB2.1 (Photosystem II light harvesting complex gene 2.1); chlorophyll binding |
| A_84_P22113 | 0.0445 | 0.4148 | AT3G02870 | protein coding |
| A_84_P22953 | 0.0225 | 0.4148 | AT2G25140 | CLPB-M/CLPB4/HSP98.7 (HEAT SHOCK PROTEIN 98.7); ATP binding / ATPase |
| A_84_P544270 | 0.0282 | 0.4116 | AT5G44780 | hypothetical protein |
| A_84_P19603 | 0.0371 | 0.4109 | AT1G62740 | protein coding |
| A_84_P12256 | 0.0032 | 0.4103 | AT1G56710 | protein coding |
| A_84_P803542 | 0.0148 | 0.4098 | AT4G32208 | hypothetical protein |
| A_84_P15842 | 0.0209 | 0.4094 | AT5G10220 | ANN6 (ANNEXIN ARABIDOPSIS 6); calcium ion binding / calcium-dependent phospholipid binding |
| A_84_P581126 | 0.0185 | 0.4091 | AT2G46670 | pseudo-response regulator, putative / timing of CAB expression 1-like protein, putative |
| A_84_P15382 | 0.0411 | 0.409 | AT2G30770 | CYP71A13 (CYTOCHROME P450, FAMILY 71, SUBFAMILY A, POLYPEPTIDE 13); indoleacetaldoxime dehydratase/ |
| A_84_P17413 | 0.0148 | 0.4076 | AT3G25230 | protein coding |
| A_84_P15703 | 0.0318 | 0.4063 | AT4G18340 | glycosyl hydrolase family 17 protein |
| A_84_P13191 | 0.0396 | 0.405 | AT5G21100 | L-ascorbate oxidase, putative |
| A_84_P500425 | 0.0345 | 0.4035 | AT3G14205 | protein coding |
| A_84_P10688 | 0.0031 | 0.4024 | AT1G30070 | protein coding |
| A_84_P12525 | 0.0387 | 0.4018 | AT2G47880 | glutaredoxin family protein |
| A_84_P861010 | 0.0416 | 0.4008 | AT3G14172 | protein coding |
| A_84_P126821 | 0.0094 | 0.4003 | AT2G46790 | APRR9 (PSEUDO-RESPONSE REGULATOR 9); transcription regulator |
| A_84_P849700 | 0.0128 | 0.3998 | AT2G41650 | hypothetical protein |
| A_84_P16561 | 0.0419 | 0.3983 | AT3G54220 | protein coding |
| A_84_P15570 | 0.031 | 0.397 | AT3G43800 | protein coding |
| A_84_P20316 | 0.0456 | 0.3969 | AT1G48500 | protein coding |
| A_84_P762102 | 0.0011 | 0.3945 | AT3G49115 | protein coding |
| A_84_P11504 | 0.0065 | 0.3937 | AT1G36240 | protein coding |
| A_84_P831839 | 0.0235 | 0.3934 | AT1G28350 | protein coding |
| A_84_P142169 | 0.0496 | 0.3923 | AT4G00180 | YAB3 (YABBY3) |
| A_84_P141179 | 0.0209 | 0.3908 | AT5G04840 | bZIP protein |
| A_84_P19384 | 0.0259 | 0.3896 | AT3G51080 | protein coding |
| A_84_P839691 | 0.0224 | 0.3886 | AT1G30473 | protein coding |
| A_84_P523896 | 0.0251 | 0.3881 | AT5G50335 | hypothetical protein |
| A_84_P14882 | 0.0243 | 0.3879 | AT1G06620 | protein coding |
| A_84_P766447 | 0.0025 | 0.3877 | AT5G02500 | HSC70-1 (heat shock cognate 70 kDa protein 1); ATP binding |
| A_84_P17584 | 0.0433 | 0.3871 | AT1G17380 | protein coding |
| A_84_P833705 | 0.046 | 0.3869 | AT5G19310 | homeotic gene regulator, putative |
| A_84_P587219 | 0.0103 | 0.3868 | AT1G69526 | protein coding |
| A_84_P22402 | 0.0272 | 0.3852 | AT4G36220 | FAH1 (FERULATE-5-HYDROXYLASE 1); ferulate 5-hydroxylase |
| A_84_P787143 | 0.02 | 0.3842 | AT5G49560 | hypothetical protein |
| A_84_P11445 | 0.0463 | 0.3828 | AT1G08260 | protein coding |
| A_84_P565120 | 0.0356 | 0.3811 | AT3G08505 | protein coding |
| A_84_P12962 | 0.0194 | 0.3764 | AT4G15280 | UDP-glucoronosyl/UDP-glucosyl transferase family protein |
| A_84_P20989 | 0.0056 | 0.3763 | AT1G53930 | protein coding |
| A_84_P10109 | 0.0164 | 0.3751 | AT4G36240 | zinc finger (GATA type) family protein |
| A_84_P503380 | 0.0207 | 0.3751 | AT5G08600 | U3 ribonucleoprotein (Utp) family protein |
| A_84_P16958 | 0.0346 | 0.3736 | AT5G15450 | APG6/CLPB-P/CLPB3 (ALBINO AND PALE GREEN 6); ATP binding / ATPase |
| A_84_P767112 | 0.0208 | 0.3735 | AT5G09590 | mtHSC70-2 (HEAT SHOCK PROTEIN 70); ATP binding / unfolded protein binding |
| A_84_P765138 | 0.0107 | 0.3731 | AT4G31398 | misc_RNA |
| A_84_P18951 | 0.0217 | 0.3725 | AT1G05170 | protein coding |
| A_84_P856766 | 0.0251 | 0.3719 | AT3G09950 | protein coding |
| A_84_P165263 | 0.0313 | 0.3702 | AT1G44318 | protein coding |
| A_84_P850398 | 0.0249 | 0.3686 | AT4G00360 | CYP86A2 (ABERRANT INDUCTION OF TYPE THREE GENES 1); oxygen binding |
| A_84_P199584 | 0.0054 | 0.3683 | AT3G14200 | protein coding |
| A_84_P12141 | 0.0231 | 0.3669 | AT1G66230 | protein coding |
| A_84_P856120 | 0.026 | 0.3656 | AT3G28550 | protein coding |
| A_84_P17083 | 0.0275 | 0.3649 | AT1G10540 | protein coding |
| A_84_P279650 | 0.0483 | 0.3641 | AT1G19490 | protein coding |
| A_84_P737916 | 0.0367 | 0.3631 | AT2G01818 | zinc ion binding |
| A_84_P19080 | 0.0175 | 0.363 | AT1G19700 | protein coding |
| A_84_P10477 | 0.0486 | 0.3626 | AT1G13080 | protein coding |
| A_84_P180334 | 0.0356 | 0.3622 | AT2G43660 | glycosyl hydrolase family protein 17 |
| A_84_P757480 | 0.0387 | 0.36 | AT2G30432 | TCL1 (TRICHOMELESS1); DNA binding |
| A_84_P12228 | 0.0314 | 0.3596 | AT5G67280 | RLK (RECEPTOR-LIKE KINASE); ATP binding / kinase/ protein serine/threonine kinase |
| A_84_P10418 | 0.0276 | 0.3594 | AT1G51440 | protein coding |
| A_84_P16668 | 0.0039 | 0.3591 | AT4G23200 | protein kinase family protein |
| A_84_P16852 | 0.0276 | 0.3587 | AT5G40390 | SIP1 (SEED IMBIBITION 1-LIKE); galactinol-sucrose galactosyltransferase/ hydrolase, hydrolyzing O-gl |
| A_84_P585407 | 0.0295 | 0.3583 | AT1G58520 | protein coding |
| A_84_P10183 | 0.0278 | 0.3558 | AT5G14130 | peroxidase, putative |
| A_84_P844786 | 0.0116 | 0.3557 | AT2G16890 | UDP-glucoronosyl/UDP-glucosyl transferase family protein |
| A_84_P18880 | 0.012 | 0.3544 | AT1G35250 | protein coding |
| A_84_P22704 | 0.0039 | 0.3533 | AT1G62450 | protein coding |
| A_84_P16780 | 0.0256 | 0.3527 | AT5G07500 | PEI1; nucleic acid binding / transcription factor |
| A_84_P15207 | 0.0273 | 0.3524 | AT1G14120 | protein coding |
| A_84_P793674 | 0.0231 | 0.3513 | AT5G01990 | auxin efflux carrier family protein |
| A_84_P21492 | 0.0473 | 0.3497 | AT5G02370 | kinesin motor protein-related |
| A_84_P14635 | 0.0061 | 0.3496 | AT1G02460 | protein coding |
| A_84_P20015 | 0.0094 | 0.3493 | AT1G33430 | protein coding |
| A_84_P789714 | 0.0322 | 0.349 | AT2G32240 | hypothetical protein |
| A_84_P20743 | 0.0468 | 0.3461 | AT5G15740 | hypothetical protein |
| A_84_P762988 | 0.0297 | 0.3449 | AT4G36950 | MAPKKK21; ATP binding / protein kinase |
| A_84_P835353 | 0.0285 | 0.3447 | AT3G28345 | protein coding |
| A_84_P13831 | 0.0201 | 0.343 | AT4G23150 | protein kinase family protein |
| A_84_P217598 | 0.027 | 0.3389 | AT4G36570 | myb family transcription factor |
| A_84_P18793 | 0.0292 | 0.335 | AT5G54080 | HGO (HOMOGENTISATE 1,2-DIOXYGENASE); homogentisate 1,2-dioxygenase |
| A_84_P828124 | 0.0321 | 0.333 | AT1G72360 | protein coding |
| A_84_P16630 | 0.0259 | 0.3315 | AT4G09750 | short-chain dehydrogenase/reductase (SDR) family protein |
| A_84_P22386 | 0.0105 | 0.3314 | AT4G32540 | YUC (YUCCA); FAD binding / NADP binding / flavin-containing monooxygenase/ monooxygenase/ oxidoreduc |
| A_84_P767270 | 0.0358 | 0.3287 | AT5G20240 | PI (PISTILLATA); DNA binding / transcription factor |
| A_84_P214628 | 0.0018 | 0.3264 | AT5G52900 | hypothetical protein |
| A_84_P18342 | 0.0073 | 0.3253 | AT3G16050 | protein coding |
| A_84_P22643 | 0.002 | 0.3251 | AT5G20150 | SPX (SYG1/Pho81/XPR1) domain-containing protein |
| A_84_P763975 | 0.0062 | 0.3226 | AT4G20240 | (cytochrome P450, family 71, subfamily A, polypeptide 27); oxygen binding |
| A_84_P12763 | 0.0067 | 0.3207 | AT3G50560 | protein coding |
| A_84_P11731 | 0.0088 | 0.3179 | AT3G24500 | protein coding |
| A_84_P12513 | 0.0244 | 0.3176 | AT2G22240 | inositol-3-phosphate synthase isozyme 2 / myo-inositol-1-phosphate synthase 2 / MI-1-P synthase 2 / |
| A_84_P23395 | 0.0072 | 0.3176 | AT5G06000 | EIF3G2 (eukaryotic translation initiation factor 3G2); RNA binding / translation initiation factor |
| A_84_P18710 | 0.034 | 0.3163 | AT5G23660 | MTN3 (ARABIDOPSIS HOMOLOG OF MEDICAGO TRUNCATULA MTN3) |
| A_84_P15038 | 0.0424 | 0.3088 | AT5G61350 | protein kinase family protein |
| A_84_P16663 | 0.0418 | 0.3062 | AT4G21820 | calmodulin-binding family protein |
| A_84_P162443 | 0.0246 | 0.3058 | AT5G64510 | hypothetical protein |
| A_84_P15821 | 0.0419 | 0.3049 | AT5G03790 | ATHB51/LMI1 (LATE MERISTEM IDENTITY1); DNA binding / sequence-specific DNA binding / transcription f |
| A_84_P74014 | 0.0338 | 0.3042 | AT3G11340 | protein coding |
| A_84_P827965 | 0.0292 | 0.3041 | AT3G26932 | protein coding |
| A_84_P101246 | 0.0162 | 0.3012 | AT5G22530 | hypothetical protein |
| A_84_P215208 | 0.0356 | 0.3001 | AT3G57980 | protein coding |
| A_84_P21218 | 0.0166 | 0.2993 | AT1G12960 | protein coding |
| A_84_P506004 | 0.0269 | 0.2963 | AT5G01790 | hypothetical protein |
| A_84_P567922 | 0.0343 | 0.2934 | AT3G59250 | protein coding |
| A_84_P769417 | 0.0185 | 0.2924 | AT5G41761 | hypothetical protein |
| A_84_P20653 | 0.0396 | 0.2922 | AT5G45220 | Toll-Interleukin-Resistance (TIR) domain-containing protein |
| A_84_P837561 | 0.0467 | 0.2915 | AT5G06790 | hypothetical protein |
| A_84_P14905 | 0.0195 | 0.2872 | AT1G18350 | protein coding |
| A_84_P16844 | 0.0474 | 0.2871 | AT5G38250 | serine/threonine protein kinase, putative |
| A_84_P17365 | 0.0143 | 0.2853 | AT3G08970 | protein coding |
| A_84_P581112 | 0.0437 | 0.2853 | AT2G42150 | DNA-binding bromodomain-containing protein |
| A_84_P135105 | 0.0296 | 0.2848 | AT3G17640 | protein coding |
| A_84_P17757 | 0.0492 | 0.2836 | AT5G18670 | BMY3 (BETA-AMYLASE 9); beta-amylase |
| A_84_P23757 | 0.028 | 0.2824 | AT1G76420 | protein coding |
| A_84_P11916 | 0.0454 | 0.2823 | AT4G12870 | gamma interferon responsive lysosomal thiol reductase family protein / GILT family protein |
| A_84_P18401 | 0.006 | 0.282 | AT3G12580 | protein coding |
| A_84_P832786 | 0.0199 | 0.2797 | AT5G23480 | hypothetical protein |
| A_84_P223359 | 0.0457 | 0.2785 | AT1G54000 | protein coding |
| A_84_P20876 | 0.0205 | 0.275 | AT1G64780 | protein coding |
| A_84_P17111 | 0.0225 | 0.2735 | AT1G02820 | protein coding |
| A_84_P21423 | 0.0438 | 0.2734 | AT4G28680 | tyrosine decarboxylase, putative |
| A_84_P606765 | 0.0361 | 0.2734 | AT1G56555 | protein coding |
| A_84_P21474 | 0.035 | 0.2716 | AT4G15380 | CYP705A4 (cytochrome P450, family 705, subfamily A, polypeptide 4); oxygen binding |
| A_84_P10627 | 0.0061 | 0.2697 | AT2G20880 | AP2 domain-containing transcription factor, putative |
| A_84_P15946 | 0.0037 | 0.2696 | AT5G51440 | 23.5 kDa mitochondrial small heat shock protein (HSP23.5-M) |
| A_84_P14320 | 0.002 | 0.2695 | AT1G74310 | protein coding |
| A_84_P21169 | 0.0074 | 0.2681 | AT3G04280 | protein coding |
| A_84_P752907 | 0.0282 | 0.2678 | AT1G13605 | protein coding |
| A_84_P17828 | 0.0024 | 0.2671 | AT5G48570 | peptidyl-prolyl cis-trans isomerase, putative / FK506-binding protein, putative |
| A_84_P558814 | 9.00E-04 | 0.2649 | AT2G25780 | hypothetical protein |
| A_84_P23712 | 0.0296 | 0.2646 | AT1G59860 | protein coding |
| A_84_P264360 | 0.0081 | 0.2636 | AT2G05350 | hypothetical protein |
| A_84_P566698 | 0.0114 | 0.2635 | AT1G68210 | protein coding |
| A_84_P15440 | 0.0138 | 0.2556 | AT1G07400 | protein coding |
| A_84_P19231 | 0.0013 | 0.2554 | AT2G26150 | ATHSFA2 (Arabidopsis thaliana heat shock transcription factor A2) |
| A_84_P193944 | 0.0334 | 0.251 | AT5G66650 | hypothetical protein |
| A_84_P16777 | 0.0383 | 0.2506 | AT5G07010 | sulfotransferase family protein |
| A_84_P81409 | 0.0415 | 0.2488 | AT3G15630 | protein coding |
| A_84_P296824 | 0.0179 | 0.2487 | AT3G15450 | protein coding |
| A_84_P23679 | 0.0427 | 0.2486 | AT1G80920 | protein coding |
| A_84_P813591 | 0.0447 | 0.2455 | AT1G77120 | protein coding |
| A_84_P12642 | 0.0465 | 0.2454 | AT3G10020 | protein coding |
| A_84_P22055 | 0.0395 | 0.2432 | AT2G38380 | peroxidase 22 (PER22) (P22) (PRXEA) / basic peroxidase E |
| A_84_P20792 | 0.0451 | 0.2416 | AT1G15670 | protein coding |
| A_84_P10198 | 3.00E-04 | 0.2368 | AT5G22920 | zinc finger (C3HC4-type RING finger) family protein |
| A_84_P290004 | 0.0028 | 0.2368 | AT4G21850 | methionine sulfoxide reductase domain-containing protein / SeIR domain-containing protein |
| A_84_P188574 | 0.0207 | 0.2365 | AT5G02020 | hypothetical protein |
| A_84_P13976 | 0.0436 | 0.2341 | AT5G19120 | aspartic-type endopeptidase/ pepsin A |
| A_84_P53790 | 0.0086 | 0.234 | AT1G49500 | protein coding |
| A_84_P11129 | 0.0295 | 0.2339 | AT5G14180 | MPL1 (MYZUS PERSICAE-INDUCED LIPASE 1); catalytic |
| A_84_P809661 | 0.038 | 0.2283 | AT3G54020 | protein coding |
| A_84_P808837 | 0.0289 | 0.228 | AT2G23320 | WRKY15 (WRKY DNA-binding protein 15); transcription factor |
| A_84_P199564 | 0.0098 | 0.2276 | AT2G42890 | AML2; RNA binding |
| A_84_P178544 | 0.0492 | 0.2242 | AT1G56220 | protein coding |
| A_84_P541229 | 0.0496 | 0.2239 | AT3G13062 | protein coding |
| A_84_P22932 | 0.0159 | 0.2209 | AT2G22770 | NAI1; DNA binding / transcription factor |
| A_84_P13173 | 0.0211 | 0.2195 | AT5G67300 | ATMYB44/ATMYBR1/MYBR1 (MYB DOMAIN PROTEIN 44); DNA binding / transcription factor |
| A_84_P18467 | 0.033 | 0.2188 | AT3G57520 | protein coding |
| A_84_P17919 | 0.0185 | 0.2187 | AT5G21170 | 5'-AMP-activated protein kinase beta-2 subunit, putative |
| A_84_P56760 | 0.0301 | 0.21 | AT3G19680 | protein coding |
| A_84_P18716 | 0.027 | 0.2092 | AT5G25350 | EBF2 (EIN3-BINDING F BOX PROTEIN 2) |
| A_84_P300030 | 0.0108 | 0.2081 | AT5G08790 | ATAF2 (Arabidopsis NAC domain containing protein 81) |
| A_84_P19988 | 0.0406 | 0.2062 | AT1G12780 | protein coding |
| A_84_P255510 | 0.018 | 0.2032 | AT1G72450 | protein coding |
| A_84_P810188 | 0.0414 | 0.1997 | AT5G03240 | UBQ3 (POLYUBIQUITIN 3); protein binding |
| A_84_P798820 | 0.0324 | 0.1996 | AT2G41430 | ERD15 (EARLY RESPONSIVE TO DEHYDRATION 15) |
| A_84_P516094 | 0.0142 | 0.1993 | AT1G26665 | protein coding |
| A_84_P277980 | 0.0386 | 0.1964 | AT3G08840 | protein coding |
| A_84_P305320 | 0.0411 | 0.1957 | AT5G22310 | hypothetical protein |
| A_84_P798326 | 0.032 | 0.1939 | AT5G50670 | squamosa promoter-binding protein, putative |
| A_84_P502963 | 0.0104 | 0.1939 | AT2G22790 | hypothetical protein |
| A_84_P786235 | 0.0255 | 0.1926 | AT5G26260 | meprin and TRAF homology domain-containing protein / MATH domain-containing protein |
| A_84_P21976 | 0.0289 | 0.1911 | AT2G33150 | KAT2/PED1 (PEROXISOME DEFECTIVE 1); acetyl-CoA C-acyltransferase |
| A_84_P10170 | 0.0128 | 0.1899 | AT5G10140 | FLC (FLOWERING LOCUS C); transcription factor |
| A_84_P15965 | 0.0069 | 0.187 | AT5G56980 | hypothetical protein |
| A_84_P20120 | 8.00E-04 | 0.1859 | AT2G27690 | CYP94C1 (cytochrome P450, family 94, subfamily C, polypeptide 1); oxygen binding |
| A_84_P808523 | 0.0146 | 0.1797 | AT5G63190 | MA3 domain-containing protein |
| A_84_P12423 | 0.0483 | 0.177 | AT1G26670 | protein coding |
| A_84_P825252 | 0.0115 | 0.1759 | AT4G19390 | hypothetical protein |
| A_84_P802051 | 0.0343 | 0.1713 | AT3G16740 | protein coding |
| A_84_P17923 | 2.00E-04 | 0.1712 | AT5G28770 | BZO2H3 (ARABIDOPSIS THALIANA BASIC LEUCINE ZIPPER 63); DNA binding / transcription factor |
| A_84_P15340 | 0.013 | 0.1701 | AT2G06050 | OPR3 (OPDA-REDUCTASE 3); 12-oxophytodienoate reductase |
| A_84_P23703 | 0.0242 | 0.1699 | AT1G04400 | protein coding |
| A_84_P17101 | 0.0067 | 0.1696 | AT1G15350 | protein coding |
| A_84_P855512 | 0.0347 | 0.1678 | AT1G68190 | protein coding |
| A_84_P16244 | 0.0186 | 0.1645 | AT1G25550 | protein coding |
| A_84_P20749 | 0.047 | 0.1592 | AT5G20860 | pectinesterase family protein |
| A_84_P23377 | 0.0202 | 0.1551 | AT4G37180 | myb family transcription factor |
| A_84_P23294 | 7.00E-04 | 0.1538 | AT4G24230 | ACBP3 (ACYL-COA-BINDING DOMAIN 3); acyl-CoA binding |
| A_84_P17332 | 0.0146 | 0.151 | AT2G01850 | EXGT-A3 (endo-xyloglucan transferase A3); hydrolase, acting on glycosyl bonds / xyloglucan:xylogluco |
| A_84_P11702 | 0.02 | 0.1481 | AT1G53580 | protein coding |
| A_84_P229849 | 0.0432 | 0.1463 | AT5G60360 | AALP (ARABIDOPSIS ALEURAIN-LIKE PROTEASE); cysteine-type peptidase |
| A_84_P17092 | 2.00E-04 | 0.1458 | AT1G64720 | protein coding |
| A_84_P11914 | 1.00E-04 | 0.1454 | AT4G12400 | stress-inducible protein, putative |
| A_84_P13841 | 0.0492 | 0.144 | AT4G25380 | zinc finger (AN1-like) family protein |
| A_84_P12181 | 0.0195 | 0.1356 | AT5G56000 | heat shock protein 81-4 (HSP81-4) |
| A_84_P22012 | 0.0316 | 0.1325 | AT2G31180 | AtMYB14/Myb14at (myb domain protein 14); DNA binding / transcription factor |
| A_84_P239935 | 0.0294 | 0.1213 | AT1G01320 | protein coding |
| A_84_P11296 | 0.0266 | 0.1193 | AT5G20220 | zinc knuckle (CCHC-type) family protein |
| A_84_P217518 | 0.0197 | 0.1163 | AT1G79270 | protein coding |
| A_84_P13399 | 0.0431 | 0.1163 | AT1G17840 | protein coding |
| A_84_P170943 | 0.0421 | 0.113 | AT4G28100 | hypothetical protein |
| A_84_P11471 | 0.0155 | 0.1099 | AT1G72050 | protein coding |
| A_84_P561767 | 0.0055 | 0.1071 | AT4G14240 | hypothetical protein |
| A_84_P19485 | 0.0323 | 0.1058 | AT4G17880 | basic helix-loop-helix (bHLH) family protein |
| A_84_P12759 | 0.0216 | 0.105 | AT3G49670 | protein coding |
| A_84_P11920 | 0.0485 | 0.1043 | AT4G13860 | glycine-rich RNA-binding protein, putative |
| A_84_P137629 | 0.0079 | 0.1039 | AT5G64170 | dentin sialophosphoprotein-related |
| A_84_P67034 | 0.0325 | 0.0977 | AT1G55910 | protein coding |
| A_84_P243515 | 0.0069 | 0.0973 | AT4G27940 | mitochondrial substrate carrier family protein |
| A_84_P512133 | 0.0111 | 0.0952 | AT4G15810 | chloroplast outer membrane protein, putative |
| A_84_P300490 | 0.0114 | 0.0878 | AT4G26850 | VTC2 (VITAMIN C DEFECTIVE 2) |
| A_84_P175871 | 0.0364 | 0.087 | AT4G28290 | hypothetical protein |
| A_84_P287150 | 0.0107 | 0.0855 | AT5G56010 | HSP81-3 (Heat shock protein 81-3); ATP binding |
| A_84_P591145 | 0.0457 | 0.0853 | AT4G05631 | hypothetical protein |
| A_84_P21617 | 0.0311 | 0.0827 | AT5G49910 | cpHSC70-2 (HEAT SHOCK PROTEIN 70-7); ATP binding / unfolded protein binding |
| A_84_P283680 | 1.00E-04 | 0.0815 | AT3G07090 | protein coding |
| A_84_P757095 | 0.0487 | 0.0703 | AT2G12462 | hypothetical protein |
| A_84_P222439 | 0.0319 | 0.0658 | AT3G52370 | protein coding |
| A_84_P118252 | 0.0412 | 0.0649 | AT2G36630 | hypothetical protein |
| A_84_P22292 | 0.0162 | 0.0623 | AT4G04020 | FIB (FIBRILLIN); structural molecule |
| A_84_P271340 | 0.0081 | 0.0551 | AT3G09350 | protein coding |
| A_84_P18353 | 0.0233 | 0.0544 | AT3G19450 | protein coding |
| A_84_P268780 | 0.0347 | 0.0531 | AT1G45010 | protein coding |
| A_84_P22572 | 0.0096 | 0.0531 | AT5G52640 | HSP81-1 (HEAT SHOCK PROTEIN 81-1); ATP binding / unfolded protein binding |
| A_84_P505907 | 0.0041 | 0.0412 | AT4G12735 | hypothetical protein |
| A_84_P168163 | 0.0029 | 0.0405 | AT5G56030 | HSP81-2 (EARLY-RESPONSIVE TO DEHYDRATION 8); ATP binding |
| A_84_P16418 | 0.0026 | 0.0371 | AT3G09440 | protein coding |
| A_84_P106882 | 0.025 | 0.037 | AT1G78170 | protein coding |
| A_84_P14417 | 4.00E-04 | 0.0114 | AT2G20560 | DNAJ heat shock family protein |
