# Supplementary material for: The bZIP protein from Tamarix hispida, ThbZIP1, is ACGT elements binding factor that enhances abiotic stress signaling in transgenic Arabidopsis
Source: BMC Plant Biol. 2013 Oct 4;13:151. doi: 10.1186/1471-2229-13-151 (PMC3852707; doi:10.1186/1471-2229-13-151)
Supplement: Additional file 4: Table S2 — The significantly differentially regulated genes (p < 0.05; ratio > 2 or <0.5) in ThbZIP1 transformed plants relative to Col-0 under salt stress conditions. [file 1471-2229-13-151-S4.doc]

**Additional file 4: Table S2 Differential expression genes in the transgenic *ThbZIP1* *Arabidopsis* compared with the Col-0 in salt stress.**

| **ProbeId** | **pvalues** | **foldchange** | **Locustag** | **Description** |
| --- | --- | --- | --- | --- |
| A_84_P764941 | 0.012 | 448.0043 | AT4G22485 | hypothetical protein |
| A_84_P761298 | 0.0132 | 418.9891 | AT3G05727 | protein coding |
| A_84_P764950 | 0.0099 | 396.5156 | AT4G22517 | hypothetical protein |
| A_84_P764933 | 0.0024 | 253.1166 | AT4G22513 | hypothetical protein |
| A_84_P22341 | 0.0179 | 85.9138 | AT4G22520 | protease inhibitor/seed storage/lipid transfer protein (LTP) family protein |
| A_84_P219958 | 0.004 | 82.5544 | AT5G42760 | hypothetical protein |
| A_84_P187174 | 0.0236 | 81.7964 | AT3G17520 | protein coding |
| A_84_P756427 | 4.00E-04 | 65.9315 | AT2G27420 | cysteine proteinase, putative |
| A_84_P16086 | 0.0164 | 64.6316 | AT1G52100 | protein coding |
| A_84_P758588 | 0.0419 | 59.7834 | AT2G30766 | hypothetical protein |
| A_84_P20449 | 0.0177 | 58.2316 | AT4G22490 | protease inhibitor/seed storage/lipid transfer protein (LTP) family protein |
| A_84_P549811 | 0.0021 | 42.2663 | AT1G47395 | protein coding |
| A_84_P12696 | 0.0033 | 36.0442 | AT1G26790 | protein coding |
| A_84_P22363 | 5.00E-04 | 35.9946 | AT4G27360 | dynein light chain, putative |
| A_84_P13057 | 0.0317 | 33.7641 | AT5G36910 | THI2.2 (THIONIN 2.2); toxin receptor binding |
| A_84_P22852 | 0.0072 | 28.5263 | AT1G01520 | protein coding |
| A_84_P21265 | 0.0042 | 25.628 | AT3G48700 | protein coding |
| A_84_P567338 | 0.0053 | 22.9485 | AT5G55570 | heat shock protein binding / unfolded protein binding |
| A_84_P796533 | 0.0126 | 21.7523 | AT3G46650 | protein coding |
| A_84_P19734 | 0.0024 | 21.5612 | AT5G52570 | BETA-OHASE 2 (BETA-CAROTENE HYDROXYLASE 2) |
| A_84_P14808 | 0.036 | 21.2226 | AT4G30140 | GDSL-motif lipase/hydrolase family protein |
| A_84_P62840 | 0.0115 | 20.5084 | AT4G14690 | ELIP2 (EARLY LIGHT-INDUCIBLE PROTEIN 2); chlorophyll binding |
| A_84_P603601 | 0.003 | 20.5061 | AT4G02850 | phenazine biosynthesis PhzC/PhzF family protein |
| A_84_P20292 | 8.00E-04 | 20.3485 | AT3G02380 | protein coding |
| A_84_P16823 | 1.00E-04 | 19.8187 | AT1G01060 | protein coding |
| A_84_P521926 | 0.0408 | 19.4122 | AT4G22510 | hypothetical protein |
| A_84_P601919 | 0.0026 | 17.1202 | AT4G34550 | hypothetical protein |
| A_84_P551816 | 0.0112 | 15.6213 | AT3G03620 | protein coding |
| A_84_P22462 | 0.0041 | 15.3614 | AT5G09930 | ATGCN2 (Arabidopsis thaliana general control non-repressible 2) |
| A_84_P522715 | 0.0025 | 15.0215 | AT3G54830 | protein coding |
| A_84_P12926 | 0.0059 | 14.953 | AT4G31870 | ATGPX7 (GLUTATHIONE PEROXIDASE 7); glutathione peroxidase |
| A_84_P516715 | 0.011 | 14.5906 | AT5G15430 | calmodulin-binding protein-related |
| A_84_P12395 | 0.0012 | 14.514 | AT1G32900 | protein coding |
| A_84_P23101 | 0.0126 | 14.4316 | AT3G21890 | protein coding |
| A_84_P761316 | 0.0397 | 14.4074 | AT3G18217 | misc_RNA |
| A_84_P10810 | 0 | 14.0146 | AT3G27920 | protein coding |
| A_84_P11122 | 6.00E-04 | 13.4026 | AT5G11590 | TINY2 (TINY2); DNA binding / transcription factor |
| A_84_P21901 | 0.0417 | 13.1244 | AT1G06830 | protein coding |
| A_84_P21896 | 0.0268 | 13.0928 | AT1G72570 | protein coding |
| A_84_P23582 | 0.0086 | 12.9423 | AT5G15950 | adenosylmethionine decarboxylase family protein |
| A_84_P23950 | 0.027 | 11.9638 | AT1G07450 | protein coding |
| A_84_P17513 | 2.00E-04 | 11.5917 | AT3G55580 | protein coding |
| A_84_P14682 | 0.0295 | 11.5612 | AT3G57010 | protein coding |
| A_84_P22942 | 0.0447 | 11.3654 | AT2G41260 | M17 |
| A_84_P15740 | 1.00E-04 | 10.8112 | AT4G27030 | small conjugating protein ligase |
| A_84_P14787 | 0.04 | 10.5901 | AT4G25580 | stress-responsive protein-related |
| A_84_P21735 | 8.00E-04 | 10.4881 | AT1G12370 | protein coding |
| A_84_P117132 | 0.0012 | 10.4119 | AT5G03555 | permease, cytosine/purines, uracil, thiamine, allantoin family protein |
| A_84_P19241 | 2.00E-04 | 10.3311 | AT2G46830 | CCA1 (CIRCADIAN CLOCK ASSOCIATED 1); transcription factor |
| A_84_P22866 | 1.00E-04 | 10.3109 | AT1G20070 | protein coding |
| A_84_P15627 | 0.0089 | 10.2316 | AT3G57020 | protein coding |
| A_84_P20740 | 2.00E-04 | 10.1121 | AT5G14760 | AO (L-ASPARTATE OXIDASE); L-aspartate oxidase |
| A_84_P17111 | 0.0028 | 10.0961 | AT1G02820 | protein coding |
| A_84_P19626 | 0.0053 | 10.0281 | AT5G10250 | phototropic-responsive protein, putative |
| A_84_P11955 | 1.00E-04 | 9.9671 | AT4G26150 | CGA1 (CYTOKININ-RESPONSIVE GATA FACTOR 1); transcription factor |
| A_84_P19231 | 0.0041 | 9.891 | AT2G26150 | ATHSFA2 (Arabidopsis thaliana heat shock transcription factor A2) |
| A_84_P18868 | 0.0214 | 9.7675 | AT5G19730 | pectinesterase family protein |
| A_84_P23712 | 0.0113 | 9.698 | AT1G59860 | protein coding |
| A_84_P24070 | 0.0029 | 9.627 | AT3G27170 | protein coding |
| A_84_P169313 | 0.0165 | 9.4157 | AT2G36590 | ProT3 (PROLINE TRANSPORTER 3); amino acid transmembrane transporter |
| A_84_P19904 | 1.00E-04 | 9.4026 | AT1G10370 | protein coding |
| A_84_P12766 | 0.0208 | 9.299 | AT3G51240 | protein coding |
| A_84_P524838 | 0.0054 | 9.233 | AT5G66740 | hypothetical protein |
| A_84_P20842 | 0.0022 | 9.2083 | AT1G04650 | protein coding |
| A_84_P294704 | 0.0013 | 9.0484 | AT1G54010 | protein coding |
| A_84_P19702 | 0.0247 | 8.9815 | AT5G44110 | POP1 |
| A_84_P16314 | 0.0235 | 8.7481 | AT2G22590 | glycosyltransferase family protein |
| A_84_P759248 | 0 | 8.6873 | AT3G16175 | protein coding |
| A_84_P14520 | 0.0341 | 8.611 | AT3G01500 | protein coding |
| A_84_P13597 | 0.0086 | 8.5102 | AT3G09590 | protein coding |
| A_84_P23309 | 0.0054 | 8.4788 | AT4G27370 | VIIIB (Myosin-like protein VIIB); motor |
| A_84_P784192 | 0.038 | 8.3928 | AT5G60880 | hypothetical protein |
| A_84_P11901 | 0.0318 | 8.3813 | AT1G06100 | protein coding |
| A_84_P15283 | 0.0103 | 8.2939 | AT1G66100 | protein coding |
| A_84_P196024 | 0.0131 | 8.239 | AT4G34650 | SQS2 (SQUALENE SYNTHASE 2); farnesyl-diphosphate farnesyltransferase |
| A_84_P12249 | 0.0023 | 8.2179 | AT1G28010 | protein coding |
| A_84_P20747 | 0.0044 | 8.2086 | AT5G17050 | UDP-glucoronosyl/UDP-glucosyl transferase family protein |
| A_84_P15736 | 0.0293 | 8.1573 | AT1G21810 | protein coding |
| A_84_P17209 | 0.0354 | 8.1437 | AT1G31690 | protein coding |
| A_84_P183494 | 0.0015 | 7.9546 | AT4G24700 | hypothetical protein |
| A_84_P94599 | 9.00E-04 | 7.8651 | AT3G56290 | protein coding |
| A_84_P20876 | 0.0056 | 7.7217 | AT1G64780 | protein coding |
| A_84_P18353 | 0.0401 | 7.5934 | AT3G19450 | protein coding |
| A_84_P837561 | 0.0435 | 7.5719 | AT5G06790 | hypothetical protein |
| A_84_P11287 | 0.0035 | 7.5571 | AT5G15600 | SP1L4 (SPIRAL1-LIKE4) |
| A_84_P10101 | 0.0013 | 7.4402 | AT4G34610 | BLH6 (BELL1-LIKE HOMEODOMAIN 5); DNA binding / transcription factor |
| A_84_P17757 | 0.0043 | 7.4279 | AT5G18670 | BMY3 (BETA-AMYLASE 9); beta-amylase |
| A_84_P274380 | 0.005 | 7.3074 | AT2G25530 | AFG1-like ATPase family protein |
| A_84_P13983 | 3.00E-04 | 7.2804 | AT5G24120 | SIGE (RNA polymerase sigma subunit E); DNA binding / DNA-directed RNA polymerase/ sigma factor/ tran |
| A_84_P16506 | 0.0012 | 7.2642 | AT3G14770 | protein coding |
| A_84_P15049 | 0.0125 | 7.2007 | AT1G55180 | protein coding |
| A_84_P10257 | 0.0194 | 7.1933 | AT1G29430 | protein coding |
| A_84_P23539 | 0.0017 | 7.1298 | AT5G58770 | dehydrodolichyl diphosphate synthase, putative / DEDOL-PP synthase, putative |
| A_84_P565111 | 0.0078 | 7.0086 | AT3G05900 | protein coding |
| A_84_P18673 | 0.0134 | 7.0073 | AT5G07690 | MYB29 (myb domain protein 29); DNA binding / transcription factor |
| A_84_P14880 | 0.0456 | 6.85 | AT5G05580 | FAD8 (FATTY ACID DESATURASE 8); omega-3 fatty acid desaturase |
| A_84_P255500 | 0.0014 | 6.7283 | AT5G54130 | calcium-binding EF hand family protein |
| A_84_P19987 | 0.0013 | 6.6526 | AT1G52990 | protein coding |
| A_84_P809539 | 0.0209 | 6.6422 | AT2G34620 | mitochondrial transcription termination factor-related / mTERF-related |
| A_84_P20729 | 0.001 | 6.6322 | AT5G65010 | ASN2 (ASPARAGINE SYNTHETASE 2); asparagine synthase (glutamine-hydrolyzing) |
| A_84_P795986 | 0.0056 | 6.5886 | AT4G33560 | hypothetical protein |
| A_84_P575512 | 0.0038 | 6.5728 | AT1G26290 | protein coding |
| A_84_P282890 | 0.0043 | 6.566 | AT5G47610 | zinc finger (C3HC4-type RING finger) family protein |
| A_84_P19925 | 0.0054 | 6.5551 | AT1G03020 | protein coding |
| A_84_P16494 | 0.0051 | 6.5482 | AT3G21870 | protein coding |
| A_84_P19152 | 0.0334 | 6.5405 | AT2G22610 | kinesin motor protein-related |
| A_84_P785139 | 0.022 | 6.5266 | AT1G06360 | protein coding |
| A_84_P564785 | 0.0162 | 6.5166 | AT1G14250 | protein coding |
| A_84_P115132 | 0.0166 | 6.5058 | AT1G77580 | protein coding |
| A_84_P59780 | 2.00E-04 | 6.5012 | AT1G18810 | protein coding |
| A_84_P11530 | 0.011 | 6.4809 | AT1G78370 | protein coding |
| A_84_P793020 | 0.032 | 6.4731 | AT3G03450 | protein coding |
| A_84_P838708 | 0.0236 | 6.4651 | AT1G20870 | protein coding |
| A_84_P272230 | 0.0172 | 6.4159 | AT2G24540 | AFR (ATTENUATED FAR-RED RESPONSE) |
| A_84_P610895 | 0.0107 | 6.2806 | AT5G19970 | hypothetical protein |
| A_84_P567922 | 0.0412 | 6.2693 | AT3G59250 | protein coding |
| A_84_P16906 | 0.0058 | 6.2287 | AT5G55380 | membrane bound O-acyl transferase (MBOAT) family protein / wax synthase-related |
| A_84_P266650 | 7.00E-04 | 6.2171 | AT3G54510 | protein coding |
| A_84_P515014 | 0.0036 | 6.1933 | AT5G48920 | hydroxyproline-rich glycoprotein family protein |
| A_84_P22985 | 0.0412 | 6.1573 | AT2G36870 | xyloglucan:xyloglucosyl transferase, putative / xyloglucan endotransglycosylase, putative / endo-xyl |
| A_84_P18798 | 0.0207 | 6.0626 | AT5G55400 | fimbrin-like protein, putative |
| A_84_P575402 | 0.0038 | 5.9557 | AT1G03055 | protein coding |
| A_84_P22494 | 0.0033 | 5.9208 | AT5G23730 | nucleotide binding |
| A_84_P707913 | 0.0284 | 5.9176 | AT2G01918 | calcium ion binding |
| A_84_P14417 | 0.0029 | 5.8812 | AT2G20560 | DNAJ heat shock family protein |
| A_84_P547483 | 0.0179 | 5.8737 | AT3G27025 | protein coding |
| A_84_P18661 | 0.0057 | 5.8521 | AT5G04660 | CYP77A4 (cytochrome P450, family 77, subfamily A, polypeptide 4); oxygen binding |
| A_84_P10354 | 0.0319 | 5.8501 | AT1G27940 | protein coding |
| A_84_P601177 | 0.006 | 5.8451 | AT5G48690 | hypothetical protein |
| A_84_P11194 | 0.0117 | 5.7698 | AT5G44390 | FAD-binding domain-containing protein |
| A_84_P16792 | 3.00E-04 | 5.7621 | AT5G11260 | HY5 (ELONGATED HYPOCOTYL 5); DNA binding / transcription factor |
| A_84_P756973 | 0.0095 | 5.6963 | AT2G04032 | ZIP7 (ZINC TRANSPORTER 7 PRECURSOR); cation transmembrane transporter |
| A_84_P556074 | 0.0196 | 5.6394 | AT1G70985 | protein coding |
| A_84_P17183 | 3.00E-04 | 5.6232 | AT1G67070 | protein coding |
| A_84_P15074 | 0.001 | 5.6084 | AT5G20970 | heat shock family protein |
| A_84_P10432 | 6.00E-04 | 5.5971 | AT1G62180 | protein coding |
| A_84_P13818 | 0.0017 | 5.5894 | AT4G19820 | glycosyl hydrolase family 18 protein |
| A_84_P560489 | 0.0276 | 5.5604 | AT1G63300 | protein coding |
| A_84_P294864 | 0.0237 | 5.5496 | AT4G14150 | PAKRP1 (PHRAGMOPLAST-ASSOCIATED KINESIN-RELATED PROTEIN 1); microtubule motor/ plus-end-directed mic |
| A_84_P12493 | 0.0172 | 5.5303 | AT2G30540 | glutaredoxin family protein |
| A_84_P20887 | 0.001 | 5.53 | AT1G17050 | protein coding |
| A_84_P855921 | 0.0023 | 5.529 | AT1G50250 | protein coding |
| A_84_P513948 | 0.0342 | 5.5234 | AT4G27595 | protein transport protein-related |
| A_84_P591145 | 0.021 | 5.4339 | AT4G05631 | hypothetical protein |
| A_84_P700272 | 0.0039 | 5.4179 | AT1G56510 | protein coding |
| A_84_P581339 | 0.0013 | 5.3542 | AT4G12005 | hypothetical protein |
| A_84_P577248 | 0.022 | 5.3358 | AT1G10155 | protein coding |
| A_84_P130146 | 3.00E-04 | 5.3099 | AT2G34070 | hypothetical protein |
| A_84_P20204 | 7.00E-04 | 5.3075 | AT3G01840 | protein coding |
| A_84_P757095 | 0.0158 | 5.2782 | AT2G12462 | hypothetical protein |
| A_84_P262520 | 0.0159 | 5.2602 | AT5G55620 | hypothetical protein |
| A_84_P788429 | 0.0154 | 5.252 | AT3G12890 | protein coding |
| A_84_P826445 | 0.0261 | 5.2386 | AT4G12390 | PME1; pectinesterase inhibitor |
| A_84_P136665 | 0.0026 | 5.2375 | AT4G15430 | hypothetical protein |
| A_84_P199724 | 8.00E-04 | 5.2143 | AT5G43630 | zinc knuckle (CCHC-type) family protein |
| A_84_P21647 | 0.0111 | 5.1782 | AT5G58760 | DDB2 (DAMAGED DNA-BINDING 2); nucleotide binding |
| A_84_P16852 | 0.0012 | 5.137 | AT5G40390 | SIP1 (SEED IMBIBITION 1-LIKE); galactinol-sucrose galactosyltransferase/ hydrolase, hydrolyzing O-gl |
| A_84_P14917 | 3.00E-04 | 5.1285 | AT5G18240 | MYR1 (MYB-RELATED PROTEIN 1); transcription factor |
| A_84_P553165 | 0.0027 | 5.1033 | AT5G61270 | PIF7 (PHYTOCHROME-INTERACTING FACTOR7); DNA binding / transcription factor |
| A_84_P558950 | 0.0455 | 5.0957 | AT3G17130 | protein coding |
| A_84_P20679 | 0.0316 | 5.0805 | AT5G52280 | protein transport protein-related |
| A_84_P15993 | 0.0368 | 5.0514 | AT5G63450 | CYP94B1 (cytochrome P450, family 94, subfamily B, polypeptide 1); oxygen binding |
| A_84_P21743 | 0.0363 | 5.0103 | AT1G33811 | protein coding |
| A_84_P849175 | 0.0472 | 4.9925 | AT1G55490 | protein coding |
| A_84_P588433 | 0.0038 | 4.9467 | AT3G61750 | protein coding |
| A_84_P304480 | 0.0235 | 4.9235 | AT3G23510 | protein coding |
| A_84_P15038 | 0.031 | 4.9204 | AT5G61350 | protein kinase family protein |
| A_84_P12513 | 0.0024 | 4.902 | AT2G22240 | inositol-3-phosphate synthase isozyme 2 / myo-inositol-1-phosphate synthase 2 / MI-1-P synthase 2 / |
| A_84_P18032 | 0.0033 | 4.8987 | AT1G54050 | protein coding |
| A_84_P11949 | 0.0498 | 4.8908 | AT1G32470 | protein coding |
| A_84_P519046 | 0.001 | 4.8873 | AT3G09450 | protein coding |
| A_84_P15816 | 0.0063 | 4.8484 | AT5G02270 | ATNAP9 (Non-intrinsic ABC protein 9) |
| A_84_P13014 | 0.0358 | 4.836 | AT5G12110 | elongation factor 1B alpha-subunit 1 (eEF1Balpha1) |
| A_84_P20743 | 0.0061 | 4.8289 | AT5G15740 | hypothetical protein |
| A_84_P11914 | 0.045 | 4.8248 | AT4G12400 | stress-inducible protein, putative |
| A_84_P22004 | 0.0374 | 4.8235 | AT2G36790 | UGT73C6 (UDP-GLUCOSYL TRANSFERASE 73C6); UDP-glucosyltransferase/ UDP-glycosyltransferase/ transfera |
| A_84_P17768 | 0.0132 | 4.8021 | AT5G24850 | CRY3 (CRYPTOCHROME 3); DNA binding / DNA photolyase/ FMN binding |
| A_84_P525034 | 0.0083 | 4.7979 | AT1G62975 | protein coding |
| A_84_P140529 | 0.0047 | 4.7897 | AT3G15354 | protein coding |
| A_84_P22596 | 3.00E-04 | 4.7834 | AT5G59340 | WOX2 (WUSCHEL-related homeobox 2); transcription factor |
| A_84_P585407 | 0.0082 | 4.754 | AT1G58520 | protein coding |
| A_84_P20608 | 0.0018 | 4.7383 | AT5G25370 | PLDALPHA3 (PHOSPHLIPASE D ALPHA 3); phospholipase D |
| A_84_P769637 | 0.0081 | 4.7199 | AT5G15200 | 40S ribosomal protein S9 (RPS9B) |
| A_84_P291564 | 5.00E-04 | 4.7028 | AT1G59850 | protein coding |
| A_84_P515003 | 0.0332 | 4.7003 | AT5G45540 | hypothetical protein |
| A_84_P13435 | 7.00E-04 | 4.6988 | AT1G73370 | protein coding |
| A_84_P16114 | 0.0284 | 4.6861 | AT1G09350 | protein coding |
| A_84_P23387 | 7.00E-04 | 4.6847 | AT5G03260 | LAC11 (laccase 11); copper ion binding / oxidoreductase |
| A_84_P12560 | 0.0059 | 4.6843 | AT2G31380 | STH (salt tolerance homologue); transcription factor/ zinc ion binding |
| A_84_P11285 | 0.0243 | 4.6584 | AT5G14860 | transferase, transferring glycosyl groups |
| A_84_P243515 | 0.0021 | 4.6547 | AT4G27940 | mitochondrial substrate carrier family protein |
| A_84_P789435 | 0.0138 | 4.6454 | AT2G01505 | CLE16 (CLAVATA3/ESR-RELATED 16); receptor binding |
| A_84_P14540 | 0.0296 | 4.6324 | AT3G10840 | protein coding |
| A_84_P76184 | 0.0285 | 4.6211 | AT5G24110 | WRKY30 (WRKY DNA-binding protein 30); transcription factor |
| A_84_P18118 | 0.0254 | 4.6123 | AT1G56430 | protein coding |
| A_84_P21843 | 0.0065 | 4.6033 | AT1G22430 | protein coding |
| A_84_P753739 | 0.0309 | 4.5892 | AT1G51680 | protein coding |
| A_84_P19531 | 0.0197 | 4.5885 | AT4G28660 | PSB28 (PHOTOSYSTEM II REACTION CENTER PSB28 PROTEIN) |
| A_84_P13239 | 0.0135 | 4.5756 | AT1G50280 | protein coding |
| A_84_P521496 | 0.0227 | 4.5675 | AT1G65010 | protein coding |
| A_84_P14542 | 0.0069 | 4.5593 | AT3G09600 | protein coding |
| A_84_P12141 | 0.004 | 4.5393 | AT1G66230 | protein coding |
| A_84_P503380 | 0.0035 | 4.5366 | AT5G08600 | U3 ribonucleoprotein (Utp) family protein |
| A_84_P12515 | 0.0226 | 4.5283 | AT2G37080 | myosin heavy chain-related |
| A_84_P519713 | 0.0046 | 4.524 | AT1G66840 | protein coding |
| A_84_P18034 | 0.0013 | 4.5 | AT1G78510 | protein coding |
| A_84_P769126 | 0.0328 | 4.4965 | AT5G41071 | hypothetical protein |
| A_84_P18848 | 0.0349 | 4.4942 | AT5G14740 | CA2 (BETA CARBONIC ANHYDRASE 2); carbonate dehydratase/ zinc ion binding |
| A_84_P849911 | 0.0147 | 4.4866 | AT5G56850 | hypothetical protein |
| A_84_P23218 | 0.0155 | 4.4484 | AT3G62930 | protein coding |
| A_84_P15091 | 0.0061 | 4.4427 | AT1G56670 | protein coding |
| A_84_P106882 | 0.0409 | 4.4373 | AT1G78170 | protein coding |
| A_84_P13586 | 2.00E-04 | 4.425 | AT3G10680 | protein coding |
| A_84_P547385 | 0.0051 | 4.4197 | AT2G45403 | hypothetical protein |
| A_84_P51800 | 1.00E-04 | 4.4126 | AT3G23210 | protein coding |
| A_84_P214948 | 0.0245 | 4.3992 | AT5G08050 | hypothetical protein |
| A_84_P760202 | 0.0338 | 4.3935 | AT3G15520 | protein coding |
| A_84_P15002 | 0 | 4.3875 | AT5G51890 | peroxidase |
| A_84_P13432 | 6.00E-04 | 4.3812 | AT1G32100 | protein coding |
| A_84_P16418 | 0.0091 | 4.3506 | AT3G09440 | protein coding |
| A_84_P13039 | 0.0207 | 4.3504 | AT5G24420 | glucosamine/galactosamine-6-phosphate isomerase-related |
| A_84_P14140 | 2.00E-04 | 4.3349 | AT5G19850 | hydrolase, alpha/beta fold family protein |
| A_84_P10237 | 0.0397 | 4.3329 | AT5G41140 | hypothetical protein |
| A_84_P18474 | 0.0052 | 4.3289 | AT3G59400 | protein coding |
| A_84_P10931 | 0.0012 | 4.3003 | AT4G00050 | UNE10 (unfertilized embryo sac 10); DNA binding / transcription factor |
| A_84_P12117 | 0.0384 | 4.2933 | AT1G55960 | protein coding |
| A_84_P15783 | 0.0069 | 4.2812 | AT4G37760 | SQE3 (SQUALENE EPOXIDASE 3); oxidoreductase |
| A_84_P191914 | 7.00E-04 | 4.2796 | AT2G21320 | zinc finger (B-box type) family protein |
| A_84_P842891 | 0.0233 | 4.2737 | AT3G17360 | protein coding |
| A_84_P19884 | 0.0129 | 4.2712 | AT1G08810 | protein coding |
| A_84_P606233 | 0.0134 | 4.2633 | AT3G56260 | protein coding |
| A_84_P23174 | 0.0179 | 4.2468 | AT3G52720 | protein coding |
| A_84_P123192 | 0.0334 | 4.2434 | AT5G51720 | hypothetical protein |
| A_84_P22126 | 0.0031 | 4.2408 | AT3G23410 | protein coding |
| A_84_P18071 | 0.0041 | 4.2401 | AT1G10960 | protein coding |
| A_84_P545125 | 0.0075 | 4.2313 | AT5G28320 | hypothetical protein |
| A_84_P17028 | 0.0322 | 4.2277 | AT1G62560 | protein coding |
| A_84_P504728 | 0.0104 | 4.2239 | AT2G18120 | SRS4 (SHI-RELATED SEQUENCE 4) |
| A_84_P12008 | 0.0058 | 4.2213 | AT4G38960 | zinc finger (B-box type) family protein |
| A_84_P754189 | 0.0242 | 4.2177 | AT1G77138 | misc_RNA |
| A_84_P840256 | 0.0464 | 4.2109 | AT1G45010 | protein coding |
| A_84_P58590 | 0.0154 | 4.1985 | AT3G52740 | protein coding |
| A_84_P20023 | 8.00E-04 | 4.1955 | AT1G07180 | protein coding |
| A_84_P542288 | 0.0346 | 4.1928 | AT4G17470 | palmitoyl protein thioesterase family protein |
| A_84_P116422 | 2.00E-04 | 4.1812 | AT4G12700 | hypothetical protein |
| A_84_P17525 | 0.0498 | 4.1725 | AT3G58650 | protein coding |
| A_84_P167673 | 0.021 | 4.1584 | AT5G38430 | ribulose bisphosphate carboxylase small chain 1B / RuBisCO small subunit 1B (RBCS-1B) (ATS1B) |
| A_84_P266190 | 0.0163 | 4.1562 | AT1G26210 | protein coding |
| A_84_P20499 | 0.009 | 4.1538 | AT4G33810 | glycosyl hydrolase family 10 protein |
| A_84_P737847 | 0.0119 | 4.1427 | AT2G41312 | miscRNA |
| A_84_P309853 | 0.0278 | 4.1301 | AT5G44040 | hypothetical protein |
| A_84_P16960 | 0.0288 | 4.1301 | AT5G15850 | COL1 (CONSTANS-LIKE 1); transcription factor/ zinc ion binding |
| A_84_P11127 | 0.0029 | 4.1261 | AT5G13630 | GUN5 (GENOMES UNCOUPLED 5) |
| A_84_P228049 | 0.0251 | 4.1113 | AT5G63160 | BT1 (BTB and TAZ domain protein 1); protein binding / transcription regulator |
| A_84_P222439 | 0.044 | 4.1057 | AT3G52370 | protein coding |
| A_84_P307130 | 9.00E-04 | 4.0945 | AT1G44575 | protein coding |
| A_84_P75044 | 0.007 | 4.0686 | AT5G02830 | pentatricopeptide (PPR) repeat-containing protein |
| A_84_P16910 | 0.0034 | 4.0651 | AT5G56720 | malate dehydrogenase, cytosolic, putative |
| A_84_P861607 | 0.0467 | 4.0605 | AT3G15950 | protein coding |
| A_84_P11635 | 0.0151 | 4.056 | AT2G43910 | thiol methyltransferase, putative |
| A_84_P17365 | 0.0024 | 4.0511 | AT3G08970 | protein coding |
| A_84_P23664 | 0.0355 | 4.0475 | AT1G64670 | protein coding |
| A_84_P18462 | 0.0239 | 4.043 | AT3G56270 | protein coding |
| A_84_P102716 | 2.00E-04 | 4.0388 | AT4G18630 | hypothetical protein |
| A_84_P856526 | 0.0336 | 4.0259 | AT1G53310 | protein coding |
| A_84_P127131 | 0.0095 | 4.0139 | AT3G05750 | protein coding |
| A_84_P12603 | 7.00E-04 | 4.0005 | AT2G35660 | CTF2A; monooxygenase |
| A_84_P17828 | 0.0069 | 3.9911 | AT5G48570 | peptidyl-prolyl cis-trans isomerase, putative / FK506-binding protein, putative |
| A_84_P21280 | 0.0208 | 3.9872 | AT3G52130 | protein coding |
| A_84_P20553 | 6.00E-04 | 3.978 | AT5G04680 | hypothetical protein |
| A_84_P305070 | 0.0454 | 3.9685 | AT2G15020 | hypothetical protein |
| A_84_P739644 | 0.0387 | 3.9534 | AT1G70260 | protein coding |
| A_84_P761356 | 0.0062 | 3.9524 | AT3G61898 | protein coding |
| A_84_P825699 | 0.0093 | 3.9468 | AT5G35970 | DNA-binding protein, putative |
| A_84_P16450 | 0.0202 | 3.9404 | AT3G16000 | protein coding |
| A_84_P610114 | 0.0395 | 3.9338 | AT5G61865 | hypothetical protein |
| A_84_P23290 | 0.0241 | 3.9332 | AT4G23290 | protein kinase family protein |
| A_84_P16449 | 0.0054 | 3.8944 | AT3G22840 | protein coding |
| A_84_P205958 | 0.02 | 3.8862 | AT4G24930 | thylakoid lumenal 17.9 kDa protein, chloroplast |
| A_84_P18147 | 7.00E-04 | 3.8723 | AT1G78580 | protein coding |
| A_84_P12892 | 0.0282 | 3.8722 | AT4G24510 | CER2 (ECERIFERUM 2); transferase |
| A_84_P10483 | 0.0131 | 3.8656 | AT1G15260 | protein coding |
| A_84_P598113 | 0.0021 | 3.861 | AT3G66652 | protein coding |
| A_84_P754151 | 0.0309 | 3.8557 | AT1G26761 | protein coding |
| A_84_P23855 | 0.0206 | 3.855 | AT2G35370 | GDCH (Glycine decarboxylase complex H) |
| A_84_P239815 | 0.0492 | 3.85 | AT1G12020 | protein coding |
| A_84_P13806 | 0.0048 | 3.8382 | AT4G12890 | gamma interferon responsive lysosomal thiol reductase family protein / GILT family protein |
| A_84_P258870 | 0.0043 | 3.8362 | AT1G72130 | protein coding |
| A_84_P22977 | 0.0159 | 3.8332 | AT2G23000 | SCPL10 (serine carboxypeptidase-like 10); serine carboxypeptidase |
| A_84_P15712 | 0.0218 | 3.8322 | AT1G29170 | protein coding |
| A_84_P526889 | 0.0269 | 3.8293 | AT2G17300 | hypothetical protein |
| A_84_P21480 | 0.0024 | 3.8265 | AT4G17090 | CT-BMY (BETA-AMYLASE 3, BETA-AMYLASE 8); beta-amylase |
| A_84_P501388 | 0.037 | 3.824 | AT3G51220 | protein coding |
| A_84_P12011 | 0.0046 | 3.821 | AT4G39800 | MI-1-P SYNTHASE (Myo-inositol-1-phosphate synthase); inositol-3-phosphate synthase |
| A_84_P166063 | 0.0276 | 3.8194 | AT4G15620 | integral membrane family protein |
| A_84_P12484 | 0.0301 | 3.8132 | AT1G58170 | protein coding |
| A_84_P193384 | 0.0063 | 3.8102 | AT5G67385 | signal transducer |
| A_84_P22798 | 0.026 | 3.81 | AT1G78320 | protein coding |
| A_84_P107162 | 0.0034 | 3.7994 | AT5G62100 | ATBAG2 (ARABIDOPSIS THALIANA BCL-2-ASSOCIATED ATHANOGENE 2); protein binding |
| A_84_P24005 | 0.0162 | 3.7911 | AT3G02830 | protein coding |
| A_84_P203658 | 0.0426 | 3.7797 | AT2G44930 | hypothetical protein |
| A_84_P52840 | 0.0019 | 3.7724 | AT3G54500 | protein coding |
| A_84_P831380 | 0.0421 | 3.7648 | AT2G25480 | hypothetical protein |
| A_84_P613604 | 0.0154 | 3.7636 | AT5G42710 | hypothetical protein |
| A_84_P842382 | 0.0018 | 3.746 | AT5G37180 | SUS5; UDP-glycosyltransferase/ sucrose synthase |
| A_84_P10613 | 0.0419 | 3.7441 | AT2G42530 | COR15B |
| A_84_P272560 | 0.0076 | 3.7347 | AT3G12970 | protein coding |
| A_84_P21277 | 0.0406 | 3.7299 | AT3G51380 | protein coding |
| A_84_P16919 | 0.0273 | 3.7217 | AT5G59130 | subtilase family protein |
| A_84_P847870 | 0.0119 | 3.717 | AT3G22104 | protein coding |
| A_84_P118252 | 0.0035 | 3.7161 | AT2G36630 | hypothetical protein |
| A_84_P572140 | 0.0227 | 3.7157 | AT2G22140 | hypothetical protein |
| A_84_P19952 | 0.0166 | 3.708 | AT1G14840 | protein coding |
| A_84_P168163 | 0.0189 | 3.7068 | AT5G56030 | HSP81-2 (EARLY-RESPONSIVE TO DEHYDRATION 8); ATP binding |
| A_84_P18787 | 0.0227 | 3.7041 | AT5G52250 | transducin family protein / WD-40 repeat family protein |
| A_84_P21199 | 0.0042 | 3.7034 | AT3G18750 | protein coding |
| A_84_P24047 | 0.0417 | 3.6923 | AT3G21950 | protein coding |
| A_84_P23427 | 1.00E-04 | 3.6916 | AT5G16600 | MYB43 (myb domain protein 43); DNA binding / transcription factor |
| A_84_P562730 | 0.0124 | 3.6856 | AT4G38060 | hypothetical protein |
| A_84_P836393 | 0.0021 | 3.6685 | AT4G26190 | hypothetical protein |
| A_84_P14583 | 0.012 | 3.6682 | AT3G20270 | protein coding |
| A_84_P849408 | 0.0181 | 3.6653 | AT2G30520 | RPT2 (ROOT PHOTOTROPISM 2) |
| A_84_P12668 | 0.0144 | 3.6629 | AT3G22790 | protein coding |
| A_84_P279650 | 0.0041 | 3.6628 | AT1G19490 | protein coding |
| A_84_P200704 | 0.0074 | 3.6579 | AT5G06270 | hypothetical protein |
| A_84_P761076 | 0.0048 | 3.6576 | AT3G55646 | protein coding |
| A_84_P220408 | 0.0237 | 3.6572 | AT1G16750 | protein coding |
| A_84_P24095 | 0.0289 | 3.6536 | AT3G46670 | protein coding |
| A_84_P106106 | 0.0134 | 3.6449 | AT5G23820 | MD-2-related lipid recognition domain-containing protein / ML domain-containing protein |
| A_84_P16962 | 1.00E-04 | 3.6448 | AT5G16730 | hypothetical protein |
| A_84_P524320 | 0.0128 | 3.6442 | AT2G40475 | hypothetical protein |
| A_84_P10684 | 0.0364 | 3.6422 | AT2G23010 | SCPL9; serine carboxypeptidase |
| A_84_P21761 | 0.0022 | 3.6364 | AT1G64500 | protein coding |
| A_84_P20245 | 0.0372 | 3.62 | AT3G19480 | protein coding |
| A_84_P23399 | 0.0059 | 3.6082 | AT5G07080 | transferase family protein |
| A_84_P510106 | 0.0105 | 3.6077 | AT2G35850 | hypothetical protein |
| A_84_P16332 | 2.00E-04 | 3.606 | AT2G29450 | ATGSTU5 (Arabidopsis thaliana Glutathione S-transferase (class tau) 5); glutathione transferase |
| A_84_P172851 | 0.0171 | 3.5944 | AT3G24535 | protein coding |
| A_84_P813485 | 0.0365 | 3.5944 | AT3G62030 | protein coding |
| A_84_P12012 | 0.0306 | 3.5905 | AT4G40010 | SNRK2-7/SNRK2.7/SRK2F (SNF1-RELATED PROTEIN KINASE 2.7); kinase |
| A_84_P811094 | 0.013 | 3.5855 | AT1G56070 | protein coding |
| A_84_P13502 | 0.0211 | 3.5836 | AT2G27820 | PD1 (PREPHENATE DEHYDRATASE 1); arogenate dehydratase/ prephenate dehydratase |
| A_84_P802894 | 0.0096 | 3.5817 | AT2G26080 | ATGLDP2 (ARABIDOPSIS THALIANA GLYCINE DECARBOXYLASE P-PROTEIN 2); glycine dehydrogenase (decarboxyla |
| A_84_P163033 | 0.0498 | 3.577 | AT3G47830 | protein coding |
| A_84_P16958 | 6.00E-04 | 3.5743 | AT5G15450 | APG6/CLPB-P/CLPB3 (ALBINO AND PALE GREEN 6); ATP binding / ATPase |
| A_84_P15075 | 1.00E-04 | 3.5635 | AT5G20070 | ATNUDT19 (Arabidopsis thaliana Nudix hydrolase homolog 19); hydrolase |
| A_84_P23719 | 0.0347 | 3.5619 | AT1G16490 | protein coding |
| A_84_P755365 | 0.0122 | 3.552 | AT2G41310 | ATRR3 (RESPONSE REGULATOR 3); transcription regulator |
| A_84_P13308 | 0.0278 | 3.5382 | AT1G79410 | protein coding |
| A_84_P506967 | 0.0042 | 3.5382 | AT5G22240 | ATOFP10/OFP10 (Arabidopsis thaliana ovate family protein 10) |
| A_84_P175871 | 4.00E-04 | 3.5313 | AT4G28290 | hypothetical protein |
| A_84_P541401 | 0.0075 | 3.5265 | AT4G18425 | hypothetical protein |
| A_84_P788188 | 0.0272 | 3.5263 | AT2G32690 | pseudo |
| A_84_P24057 | 0.0257 | 3.5197 | AT3G19620 | protein coding |
| A_84_P126241 | 0.007 | 3.5152 | AT5G41900 | hydrolase, alpha/beta fold family protein |
| A_84_P14601 | 0.042 | 3.5151 | AT3G21090 | protein coding |
| A_84_P17103 | 0.0044 | 3.5107 | AT1G17100 | protein coding |
| A_84_P826388 | 0.0142 | 3.492 | AT3G30180 | protein coding |
| A_84_P13854 | 0.0035 | 3.4858 | AT4G28080 | binding |
| A_84_P12867 | 0.0044 | 3.4854 | AT4G18290 | KAT2 (K+ ATPase 2); cyclic nucleotide binding / inward rectifier potassium channel |
| A_84_P579438 | 0.0355 | 3.4798 | AT3G24630 | protein coding |
| A_84_P148418 | 0.0249 | 3.4778 | AT1G07610 | protein coding |
| A_84_P76024 | 5.00E-04 | 3.4776 | AT1G17360 | protein coding |
| A_84_P15609 | 0.0152 | 3.4707 | AT3G53170 | protein coding |
| A_84_P811658 | 0.049 | 3.4676 | AT2G36880 | MAT3 (METHIONINE ADENOSYLTRANSFERASE 3); methionine adenosyltransferase |
| A_84_P17242 | 0.0062 | 3.4569 | AT2G37180 | RD28 (plasma membrane intrinsic protein 2;3); water channel |
| A_84_P754720 | 0.0036 | 3.4565 | AT1G79529 | misc_RNA |
| A_84_P12984 | 0.0047 | 3.4444 | AT1G62750 | protein coding |
| A_84_P74474 | 0.0316 | 3.4364 | AT2G38320 | hypothetical protein |
| A_84_P13872 | 0.0098 | 3.4242 | AT4G32190 | centromeric protein-related |
| A_84_P18084 | 0.0142 | 3.4237 | AT1G77490 | protein coding |
| A_84_P784525 | 0.0066 | 3.4231 | AT4G17100 | pseudo |
| A_84_P237923 | 0.0425 | 3.4214 | AT1G68470 | protein coding |
| A_84_P20424 | 0.0304 | 3.4176 | AT4G12310 | CYP706A5 (cytochrome P450, family 706, subfamily A, polypeptide 5); oxygen binding |
| A_84_P11207 | 0.0318 | 3.4085 | AT1G29500 | protein coding |
| A_84_P20132 | 0.03 | 3.4076 | AT2G21210 | auxin-responsive protein, putative |
| A_84_P292134 | 0.0138 | 3.403 | AT4G23490 | fringe-related protein |
| A_84_P138919 | 0.0184 | 3.3953 | AT5G28400 | hypothetical protein |
| A_84_P800943 | 8.00E-04 | 3.3927 | AT4G15550 | IAGLU (INDOLE-3-ACETATE BETA-D-GLUCOSYLTRANSFERASE); UDP-glycosyltransferase/ transferase, transferr |
| A_84_P231779 | 0.0054 | 3.3893 | AT2G43445 | hypothetical protein |
| A_84_P15489 | 9.00E-04 | 3.3877 | AT1G53090 | protein coding |
| A_84_P123822 | 0.0036 | 3.3761 | AT2G39250 | SNZ (SCHNARCHZAPFEN); DNA binding / transcription factor |
| A_84_P595528 | 0.0291 | 3.3746 | AT3G48610 | protein coding |
| A_84_P217338 | 0.0083 | 3.3734 | AT5G52060 | ATBAG1 (ARABIDOPSIS THALIANA BCL-2-ASSOCIATED ATHANOGENE 1); protein binding |
| A_84_P10959 | 0.0015 | 3.3711 | AT4G09950 | avirulence-responsive family protein / avirulence induced gene (AIG1) family protein |
| A_84_P70894 | 0.0028 | 3.365 | AT5G01840 | ATOFP1/OFP1 (ARABIDOPSIS THALIANA OVATE FAMILY PROTEIN 1); protein binding / transcription repressor |
| A_84_P161343 | 0.0378 | 3.3585 | AT5G01370 | hypothetical protein |
| A_84_P18399 | 0.0182 | 3.3499 | AT3G11170 | protein coding |
| A_84_P22105 | 0.0053 | 3.348 | AT3G04030 | protein coding |
| A_84_P800976 | 0.0357 | 3.3475 | AT1G60950 | protein coding |
| A_84_P22382 | 0.0231 | 3.3404 | AT4G31590 | ATCSLC05 (Cellulose synthase-like C5); transferase, transferring glycosyl groups |
| A_84_P266080 | 1.00E-04 | 3.3395 | AT1G80810 | protein coding |
| A_84_P504309 | 0.0127 | 3.3314 | AT5G21430 | DNAJ heat shock N-terminal domain-containing protein |
| A_84_P300490 | 0.0094 | 3.3256 | AT4G26850 | VTC2 (VITAMIN C DEFECTIVE 2) |
| A_84_P20048 | 0.0162 | 3.3228 | AT1G14345 | protein coding |
| A_84_P16685 | 0.035 | 3.3138 | AT4G26790 | GDSL-motif lipase/hydrolase family protein |
| A_84_P10327 | 0.0103 | 3.3112 | AT5G64840 | ATGCN5 (Arabidopsis thaliana general control non-repressible 5) |
| A_84_P13533 | 0.0448 | 3.308 | AT1G67790 | protein coding |
| A_84_P10011 | 0.0037 | 3.3071 | AT4G09350 | DNAJ heat shock N-terminal domain-containing protein |
| A_84_P504436 | 6.00E-04 | 3.3028 | AT5G66600 | hypothetical protein |
| A_84_P149568 | 0.0214 | 3.2902 | AT2G44260 | hypothetical protein |
| A_84_P14677 | 0.007 | 3.2899 | AT3G55800 | protein coding |
| A_84_P83919 | 0.0079 | 3.2893 | AT2G21560 | hypothetical protein |
| A_84_P117012 | 0.0248 | 3.2796 | AT1G49010 | protein coding |
| A_84_P158525 | 0.0051 | 3.2739 | AT1G15940 | protein coding |
| A_84_P502404 | 0.0284 | 3.2707 | AT4G26950 | hypothetical protein |
| A_84_P16403 | 0.0016 | 3.2661 | AT2G27360 | lipase, putative |
| A_84_P15906 | 0.0448 | 3.2632 | AT5G40380 | protein kinase family protein |
| A_84_P23933 | 0.0448 | 3.2585 | AT2G37630 | AS1/ATMYB91/ATPHAN/MYB91 (ASYMMETRIC LEAVES 1, MYB DOMAIN PROTEIN 91); DNA binding / protein homodim |
| A_84_P17249 | 1.00E-04 | 3.2583 | AT2G47490 | mitochondrial substrate carrier family protein |
| A_84_P561605 | 0.0437 | 3.2525 | AT3G15115 | protein coding |
| A_84_P799986 | 0.0289 | 3.2514 | AT1G25450 | protein coding |
| A_84_P15827 | 0.0091 | 3.2476 | AT5G05860 | UGT76C2 (UDP-glucosyl transferase 76C2); UDP-glycosyltransferase/ transferase, transferring glycosyl |
| A_84_P120482 | 0.0067 | 3.246 | AT2G44230 | hypothetical protein |
| A_84_P11296 | 4.00E-04 | 3.2412 | AT5G20220 | zinc knuckle (CCHC-type) family protein |
| A_84_P84809 | 0.0076 | 3.2405 | AT4G17540 | hypothetical protein |
| A_84_P113562 | 2.00E-04 | 3.2303 | AT2G34460 | flavin reductase-related |
| A_84_P804142 | 0.0092 | 3.2298 | AT1G17840 | protein coding |
| A_84_P832655 | 0.0458 | 3.2289 | AT1G09470 | protein coding |
| A_84_P753015 | 0.0459 | 3.2274 | AT1G76952 | protein coding |
| A_84_P244675 | 0.0433 | 3.2271 | AT3G19310 | protein coding |
| A_84_P108002 | 0.0077 | 3.2224 | AT1G18060 | protein coding |
| A_84_P263940 | 0.0107 | 3.2196 | AT1G69325 | protein coding |
| A_84_P587219 | 0.0469 | 3.2193 | AT1G69526 | protein coding |
| A_84_P13655 | 0.0291 | 3.2159 | AT1G59540 | protein coding |
| A_84_P534015 | 0.0218 | 3.2126 | AT2G23360 | transport protein-related |
| A_84_P20858 | 0.022 | 3.2105 | AT1G60360 | protein coding |
| A_84_P124012 | 0.0309 | 3.2098 | AT5G26790 | hypothetical protein |
| A_84_P700711 | 2.00E-04 | 3.2093 | AT2G21300 | kinesin motor family protein |
| A_84_P18073 | 0.0044 | 3.2089 | AT1G55690 | protein coding |
| A_84_P126821 | 0.0066 | 3.2007 | AT2G46790 | APRR9 (PSEUDO-RESPONSE REGULATOR 9); transcription regulator |
| A_84_P143889 | 0.0022 | 3.1994 | AT2G36145 | hypothetical protein |
| A_84_P19357 | 0.0287 | 3.1973 | AT3G44990 | protein coding |
| A_84_P18684 | 0.0076 | 3.1932 | AT5G11320 | YUC4 (YUCCA4); monooxygenase |
| A_84_P22141 | 0.0106 | 3.189 | AT3G16910 | protein coding |
| A_84_P12483 | 0.019 | 3.1845 | AT1G31920 | protein coding |
| A_84_P12894 | 0.015 | 3.1812 | AT4G24960 | ATHVA22D (Arabidopsis thaliana HVA22 homologue D) |
| A_84_P857708 | 0.0363 | 3.175 | AT2G40540 | KT2 (POTASSIUM TRANSPORTER 2); potassium ion transmembrane transporter |
| A_84_P19580 | 8.00E-04 | 3.173 | AT4G14760 | M protein repeat-containing protein |
| A_84_P12032 | 0.0178 | 3.1725 | AT5G01580 | OSH1 (OAS HIGH ACCUMULATION 1); catalytic |
| A_84_P553993 | 0.0218 | 3.1724 | AT5G42880 | hypothetical protein |
| A_84_P10477 | 0.0355 | 3.1691 | AT1G13080 | protein coding |
| A_84_P790872 | 0.0445 | 3.1663 | AT5G67050 | lipase class 3 family protein |
| A_84_P56690 | 5.00E-04 | 3.1656 | AT4G32770 | VTE1 (VITAMIN E DEFICIENT 1) |
| A_84_P18962 | 0.0478 | 3.1586 | AT1G75550 | protein coding |
| A_84_P585078 | 0.0366 | 3.1585 | AT5G17780 | hydrolase, alpha/beta fold family protein |
| A_84_P565120 | 3.00E-04 | 3.1579 | AT3G08505 | protein coding |
| A_84_P591506 | 0.0157 | 3.1579 | AT1G05870 | protein coding |
| A_84_P607303 | 0.0113 | 3.1454 | AT5G12300 | C2 domain-containing protein |
| A_84_P12749 | 0.0058 | 3.1441 | AT3G47180 | protein coding |
| A_84_P235073 | 0.0026 | 3.1407 | AT1G73870 | protein coding |
| A_84_P16971 | 0.0029 | 3.1394 | AT5G28840 | GME (GDP-D-MANNOSE 3',5'-EPIMERASE); GDP-mannose 3,5-epimerase/ NAD binding / catalytic |
| A_84_P536709 | 0.0258 | 3.1376 | AT2G32500 | hypothetical protein |
| A_84_P502099 | 0.002 | 3.1282 | AT2G34530 | hypothetical protein |
| A_84_P112152 | 0.0112 | 3.1263 | AT5G66270 | zinc finger (CCCH-type) family protein |
| A_84_P262140 | 0.0034 | 3.1252 | AT3G19370 | protein coding |
| A_84_P15253 | 0.0057 | 3.1248 | AT1G27720 | protein coding |
| A_84_P139499 | 0.0028 | 3.1195 | AT3G26450 | protein coding |
| A_84_P23183 | 0.0046 | 3.1179 | AT1G42430 | protein coding |
| A_84_P22155 | 0.0251 | 3.1159 | AT3G24170 | protein coding |
| A_84_P12590 | 0.0057 | 3.115 | AT2G38230 | ATPDX1.1 (PYRIDOXINE BIOSYNTHESIS 1.1); protein heterodimerization |
| A_84_P14091 | 0.0068 | 3.1113 | AT5G60890 | ATMYB34/ATR1/MYB34 (ALTERED TRYPTOPHAN REGULATION, MYB DOMAIN PROTEIN 34); DNA binding / kinase/ tra |
| A_84_P16798 | 0.0016 | 3.1099 | AT5G13360 | auxin-responsive GH3 family protein |
| A_84_P14117 | 0.0202 | 3.1098 | AT5G67090 | subtilase family protein |
| A_84_P64244 | 0.0412 | 3.1075 | AT5G27290 | hypothetical protein |
| A_84_P799163 | 0.0208 | 3.1047 | AT2G46630 | pseudo |
| A_84_P21970 | 0.0039 | 3.0907 | AT2G22200 | AP2 domain-containing transcription factor |
| A_84_P512232 | 0.0348 | 3.0882 | AT5G05350 | hypothetical protein |
| A_84_P553960 | 0.016 | 3.0877 | AT5G27240 | DNAJ heat shock N-terminal domain-containing protein |
| A_84_P19080 | 0.0011 | 3.0852 | AT1G19700 | protein coding |
| A_84_P101346 | 0.0077 | 3.0849 | AT2G44300 | lipid transfer protein-related |
| A_84_P130476 | 0.0418 | 3.0831 | AT5G62140 | hypothetical protein |
| A_84_P764875 | 0.0181 | 3.0809 | AT4G37925 | NDH-M (SUBUNIT NDH-M OF NAD(P)H:PLASTOQUINONE DEHYDROGENASE COMPLEX) |
| A_84_P19161 | 0.0055 | 3.0737 | AT2G32860 | glycosyl hydrolase family 1 protein |
| A_84_P862144 | 0.0309 | 3.0642 | AT1G73390 | protein coding |
| A_84_P518964 | 0.0491 | 3.0624 | AT2G32640 | hypothetical protein |
| A_84_P582075 | 0.0214 | 3.0614 | AT3G14170 | protein coding |
| A_84_P24132 | 0.0062 | 3.0597 | AT3G55120 | protein coding |
| A_84_P581126 | 0.0022 | 3.0574 | AT2G46670 | pseudo-response regulator, putative / timing of CAB expression 1-like protein, putative |
| A_84_P13729 | 0.0048 | 3.057 | AT1G74160 | protein coding |
| A_84_P12512 | 0.0327 | 3.0568 | AT2G29890 | VLN1 (VILLIN 1); actin binding |
| A_84_P822023 | 0.0014 | 3.0555 | AT1G23090 | protein coding |
| A_84_P12750 | 0.0014 | 3.0511 | AT3G47430 | protein coding |
| A_84_P824779 | 0.0339 | 3.0503 | AT4G01883 | hypothetical protein |
| A_84_P221276 | 0.0212 | 3.0489 | AT5G22390 | hypothetical protein |
| A_84_P19314 | 0.0091 | 3.0484 | AT3G24715 | protein coding |
| A_84_P109502 | 0.0055 | 3.0479 | AT5G05210 | nucleolar matrix protein-related |
| A_84_P19740 | 0.0167 | 3.0471 | AT5G54300 | hypothetical protein |
| A_84_P18784 | 0.0024 | 3.0465 | AT5G51460 | ATTPPA (Arabidopsis thaliana trehalose-6-phosphate phosphatase); trehalose-phosphatase |
| A_84_P834243 | 0.0398 | 3.0453 | AT5G17040 | UDP-glucoronosyl/UDP-glucosyl transferase family protein |
| A_84_P61730 | 0.0147 | 3.0448 | AT1G13730 | protein coding |
| A_84_P18342 | 0.0103 | 3.0421 | AT3G16050 | protein coding |
| A_84_P500756 | 0.0041 | 3.042 | AT5G28910 | hypothetical protein |
| A_84_P10499 | 0 | 3.0402 | AT1G08250 | protein coding |
| A_84_P13090 | 0.0446 | 3.0363 | AT5G45950 | GDSL-motif lipase/hydrolase family protein |
| A_84_P15196 | 2.00E-04 | 3.0345 | AT1G03080 | protein coding |
| A_84_P145709 | 0.0419 | 3.03 | AT1G13130 | protein coding |
| A_84_P151108 | 0.0071 | 3.0294 | AT1G74880 | protein coding |
| A_84_P603395 | 0.0076 | 3.0264 | AT2G41330 | glutaredoxin family protein |
| A_84_P10999 | 0.0144 | 3.0207 | AT4G23750 | CRF2 (CYTOKININ RESPONSE FACTOR 2); DNA binding / transcription factor |
| A_84_P200834 | 0.0074 | 3.0203 | AT1G58070 | protein coding |
| A_84_P765100 | 0.0227 | 3.019 | AT4G17098 | misc_RNA |
| A_84_P87489 | 0.0042 | 3.0133 | AT4G36640 | SEC14 cytosolic factor family protein / phosphoglyceride transfer family protein |
| A_84_P750611 | 0.0041 | 3.0129 | AT1G50180 | protein coding |
| A_84_P161503 | 0.0059 | 3.0107 | AT5G17300 | myb family transcription factor |
| A_84_P15406 | 0.0191 | 3.0078 | AT2G30150 | UDP-glucoronosyl/UDP-glucosyl transferase family protein |
| A_84_P11501 | 0.0169 | 3.0077 | AT1G53680 | protein coding |
| A_84_P10151 | 0.0363 | 3.0004 | AT5G04530 | beta-ketoacyl-CoA synthase family protein |
| A_84_P211708 | 0.0091 | 3.0003 | AT5G41620 | hypothetical protein |
| A_84_P16266 | 0.0065 | 2.998 | AT1G57770 | protein coding |
| A_84_P581112 | 0.0125 | 2.9959 | AT2G42150 | DNA-binding bromodomain-containing protein |
| A_84_P305110 | 5.00E-04 | 2.9926 | AT5G52230 | MBD13 (methyl-CpG-binding domain 13) |
| A_84_P18633 | 0.0128 | 2.9886 | AT4G14440 | enoyl-CoA hydratase/isomerase family protein |
| A_84_P835335 | 0.0049 | 2.9865 | AT5G63700 | zinc finger (C3HC4 type RING finger) family protein |
| A_84_P10947 | 0.0358 | 2.9812 | AT4G04610 | APR1 (PAPS REDUCTASE HOMOLOG 19) |
| A_84_P12643 | 0.0012 | 2.981 | AT3G05620 | protein coding |
| A_84_P276620 | 4.00E-04 | 2.9766 | AT3G26960 | protein coding |
| A_84_P216708 | 0.0068 | 2.9671 | AT2G42975 | hypothetical protein |
| A_84_P860076 | 0.0049 | 2.9667 | AT5G52920 | PKP-BETA1/PKP1/PKP2 (PLASTIDIC PYRUVATE KINASE 1); pyruvate kinase |
| A_84_P225469 | 4.00E-04 | 2.9636 | AT5G52420 | hypothetical protein |
| A_84_P23271 | 0.0397 | 2.9579 | AT4G18480 | CHLI1 (CHLORINA 42); magnesium chelatase |
| A_84_P11730 | 0.0078 | 2.9514 | AT3G21560 | protein coding |
| A_84_P10109 | 0.0198 | 2.9462 | AT4G36240 | zinc finger (GATA type) family protein |
| A_84_P22755 | 0.0274 | 2.9451 | AT1G26820 | protein coding |
| A_84_P15111 | 0.0106 | 2.9438 | AT1G19835 | protein coding |
| A_84_P830659 | 0.0196 | 2.9426 | AT1G01320 | protein coding |
| A_84_P121162 | 0.0248 | 2.9388 | AT3G44730 | protein coding |
| A_84_P50620 | 0.023 | 2.9362 | AT3G19720 | protein coding |
| A_84_P822975 | 0.0197 | 2.9336 | AT5G15910 | dehydrogenase-related |
| A_84_P21609 | 0.0141 | 2.9305 | AT1G56170 | protein coding |
| A_84_P18150 | 0.0127 | 2.9294 | AT1G32060 | protein coding |
| A_84_P584694 | 0.0472 | 2.9291 | AT2G40390 | hypothetical protein |
| A_84_P133465 | 0.0101 | 2.9256 | AT2G40400 | hypothetical protein |
| A_84_P18326 | 0.0017 | 2.9239 | AT3G08730 | protein coding |
| A_84_P20405 | 0.0156 | 2.9239 | AT4G05220 | harpin-induced protein-related / HIN1-related / harpin-responsive protein-related |
| A_84_P161083 | 0.005 | 2.922 | AT5G52410 | hypothetical protein |
| A_84_P14914 | 0.0062 | 2.9192 | AT5G17230 | PSY (PHYTOENE SYNTHASE); geranylgeranyl-diphosphate geranylgeranyltransferase |
| A_84_P537376 | 0.0497 | 2.9152 | AT1G29420 | protein coding |
| A_84_P21274 | 0.0112 | 2.9137 | AT1G62950 | protein coding |
| A_84_P59240 | 5.00E-04 | 2.9118 | AT2G39450 | ATMTP11/MTP11; cation transmembrane transporter/ manganese ion transmembrane transporter/ manganese: |
| A_84_P18514 | 0.0463 | 2.9057 | AT1G06000 | protein coding |
| A_84_P808645 | 0.0443 | 2.9055 | AT4G38970 | fructose-bisphosphate aldolase, putative |
| A_84_P22556 | 0.0313 | 2.9026 | AT5G48060 | C2 domain-containing protein |
| A_84_P797482 | 0.0439 | 2.9007 | AT3G59970 | protein coding |
| A_84_P838751 | 0.006 | 2.9001 | AT2G40260 | myb family transcription factor |
| A_84_P769622 | 0.0281 | 2.8985 | AT5G19221 | misc_RNA |
| A_84_P14108 | 0.0046 | 2.8963 | AT5G64940 | ATATH13 (ABC2 homolog 13) |
| A_84_P836767 | 0.0491 | 2.8938 | AT2G39470 | PPL2 (PSBP-LIKE PROTEIN 2); calcium ion binding |
| A_84_P20377 | 6.00E-04 | 2.886 | AT3G62160 | protein coding |
| A_84_P11887 | 1.00E-04 | 2.8844 | AT4G02660 | WD-40 repeat family protein / beige-related |
| A_84_P14436 | 0.039 | 2.8837 | AT2G39690 | hypothetical protein |
| A_84_P808741 | 0.0076 | 2.8821 | AT3G26650 | protein coding |
| A_84_P11833 | 0.0436 | 2.8766 | AT3G53830 | protein coding |
| A_84_P10418 | 0.0409 | 2.875 | AT1G51440 | protein coding |
| A_84_P18973 | 0.0201 | 2.8746 | AT1G09340 | protein coding |
| A_84_P503753 | 0.0187 | 2.8718 | AT1G63930 | protein coding |
| A_84_P211188 | 0.0306 | 2.8709 | AT5G58260 | hypothetical protein |
| A_84_P168683 | 9.00E-04 | 2.8619 | AT2G40960 | nucleic acid binding |
| A_84_P19730 | 0.0111 | 2.8535 | AT1G64330 | protein coding |
| A_84_P542156 | 0.0035 | 2.8516 | AT3G27270 | protein coding |
| A_84_P592414 | 0.0014 | 2.8507 | AT1G10800 | protein coding |
| A_84_P855265 | 0.0258 | 2.8502 | AT2G23670 | YCF37 (Arabidopsis homolog of Synechocystis YCF37) |
| A_84_P817213 | 2.00E-04 | 2.847 | AT2G46370 | JAR1 (JASMONATE RESISTANT 1) |
| A_84_P12728 | 6.00E-04 | 2.8426 | AT3G12020 | protein coding |
| A_84_P769220 | 0.0281 | 2.8416 | AT5G18404 | hypothetical protein |
| A_84_P21451 | 0.0368 | 2.8291 | AT4G35090 | CAT2 (CATALASE 2); catalase |
| A_84_P560168 | 0.0117 | 2.829 | AT5G24630 | BIN4 (BRASSINOSTEROID-INSENSITIVE4) |
| A_84_P15782 | 0.0448 | 2.826 | AT4G37550 | formamidase, putative / formamide amidohydrolase, putative |
| A_84_P11328 | 0.0222 | 2.8258 | AT1G20470 | protein coding |
| A_84_P590067 | 0.0122 | 2.8217 | AT3G05340 | protein coding |
| A_84_P246645 | 0.0109 | 2.8117 | AT2G40150 | hypothetical protein |
| A_84_P20545 | 0.0066 | 2.811 | AT5G02120 | OHP (ONE HELIX PROTEIN) |
| A_84_P15540 | 0.0208 | 2.8065 | AT1G12900 | protein coding |
| A_84_P832698 | 0.017 | 2.8034 | AT5G56000 | heat shock protein 81-4 (HSP81-4) |
| A_84_P24025 | 0.0049 | 2.8021 | AT3G18100 | protein coding |
| A_84_P271630 | 0.0305 | 2.8019 | AT1G44446 | protein coding |
| A_84_P818712 | 0.0264 | 2.7934 | AT5G17170 | ENH1 (ENHANCER OF SOS3-1) |
| A_84_P578752 | 5.00E-04 | 2.7909 | AT4G37685 | hypothetical protein |
| A_84_P21177 | 0.0102 | 2.7904 | AT3G17611 | protein coding |
| A_84_P756180 | 0.0108 | 2.7896 | AT2G14890 | AGP9 (ARABINOGALACTAN PROTEIN 9) |
| A_84_P584290 | 0.007 | 2.7893 | AT5G56050 | hypothetical protein |
| A_84_P14381 | 0.0395 | 2.7875 | AT1G14490 | protein coding |
| A_84_P18556 | 0.0463 | 2.7863 | AT1G29070 | protein coding |
| A_84_P16024 | 0.0021 | 2.7843 | AT5G19310 | homeotic gene regulator, putative |
| A_84_P17327 | 0.012 | 2.7838 | AT2G34210 | transcription initiation factor |
| A_84_P790792 | 7.00E-04 | 2.7818 | AT4G37682 | hypothetical protein |
| A_84_P553076 | 8.00E-04 | 2.7815 | AT5G34830 | hypothetical protein |
| A_84_P13416 | 0.0395 | 2.781 | AT1G11570 | protein coding |
| A_84_P16994 | 0.0221 | 2.7806 | AT2G20835 | hypothetical protein |
| A_84_P19769 | 0.0431 | 2.7775 | AT5G61570 | protein kinase family protein |
| A_84_P16316 | 0.0118 | 2.7774 | AT2G23180 | CYP96A1 (cytochrome P450, family 96, subfamily A, polypeptide 1); oxygen binding |
| A_84_P16440 | 0.0119 | 2.7746 | AT3G04140 | protein coding |
| A_84_P18858 | 0.0047 | 2.7736 | AT5G19870 | hypothetical protein |
| A_84_P273130 | 0.0466 | 2.7668 | AT5G04320 | hypothetical protein |
| A_84_P795561 | 0.0043 | 2.7603 | AT5G04140 | GLS1/GLU1/GLUS (FERREDOXIN-DEPENDENT GLUTAMATE SYNTHASE 1); glutamate synthase (ferredoxin) |
| A_84_P767575 | 0.0012 | 2.7597 | AT1G61210 | protein coding |
| A_84_P19603 | 0.0025 | 2.7558 | AT1G62740 | protein coding |
| A_84_P217518 | 0.0057 | 2.7548 | AT1G79270 | protein coding |
| A_84_P279540 | 0.0077 | 2.7485 | AT4G34630 | hypothetical protein |
| A_84_P793157 | 0.0099 | 2.7482 | AT1G16080 | protein coding |
| A_84_P22433 | 0.0026 | 2.7478 | AT5G01260 | glycoside hydrolase starch-binding domain-containing protein |
| A_84_P20447 | 0.0081 | 2.7449 | AT4G21940 | CPK15 (calcium-dependent protein kinase 15); calmodulin-dependent protein kinase/ kinase |
| A_84_P569564 | 4.00E-04 | 2.7436 | AT3G05270 | protein coding |
| A_84_P162443 | 0.0234 | 2.7435 | AT5G64510 | hypothetical protein |
| A_84_P17752 | 7.00E-04 | 2.7431 | AT5G17310 | UTP--glucose-1-phosphate uridylyltransferase, putative / UDP-glucose pyrophosphorylase, putative / U |
| A_84_P818059 | 0.0246 | 2.7423 | AT5G57345 | hypothetical protein |
| A_84_P18011 | 0.0205 | 2.7406 | AT1G75690 | protein coding |
| A_84_P283680 | 9.00E-04 | 2.7383 | AT3G07090 | protein coding |
| A_84_P12629 | 0.0121 | 2.7372 | AT3G03550 | protein coding |
| A_84_P275610 | 0.0218 | 2.7337 | AT3G20570 | protein coding |
| A_84_P245515 | 0.0311 | 2.7334 | AT5G62840 | phosphoglycerate/bisphosphoglycerate mutase family protein |
| A_84_P152528 | 0.0359 | 2.7317 | AT5G23060 | hypothetical protein |
| A_84_P10932 | 0.0039 | 2.7312 | AT4G00430 | TMP-C (PLASMA MEMBRANE INTRINSIC PROTEIN 1;4); water channel |
| A_84_P17411 | 0.0326 | 2.7309 | AT3G17040 | protein coding |
| A_84_P14968 | 0.0302 | 2.728 | AT1G47900 | protein coding |
| A_84_P15788 | 0.0291 | 2.7212 | AT4G38840 | auxin-responsive protein, putative |
| A_84_P854492 | 0.0205 | 2.7204 | AT3G21760 | protein coding |
| A_84_P173141 | 0.0025 | 2.7154 | AT3G01510 | protein coding |
| A_84_P19204 | 0.0422 | 2.7143 | AT2G24230 | leucine-rich repeat transmembrane protein kinase, putative |
| A_84_P22644 | 0.0164 | 2.7142 | AT5G20360 | octicosapeptide/Phox/Bem1p (PB1) domain-containing protein / tetratricopeptide repeat (TPR)-containi |
| A_84_P534067 | 0.0056 | 2.7136 | AT2G38640 | hypothetical protein |
| A_84_P14620 | 0.0386 | 2.7095 | AT3G52310 | protein coding |
| A_84_P793193 | 0.001 | 2.7088 | AT2G22170 | lipid-associated family protein |
| A_84_P14084 | 0.0207 | 2.7065 | AT1G01700 | protein coding |
| A_84_P527481 | 0.0041 | 2.7063 | AT5G61300 | hypothetical protein |
| A_84_P19216 | 0.0188 | 2.7046 | AT2G21330 | fructose-bisphosphate aldolase, putative |
| A_84_P569557 | 0.0112 | 2.703 | AT3G02930 | protein coding |
| A_84_P14472 | 0.0313 | 2.7019 | AT2G37040 | PAL1 (PHE AMMONIA LYASE 1); phenylalanine ammonia-lyase |
| A_84_P16044 | 3.00E-04 | 2.6991 | AT5G58160 | actin binding |
| A_84_P58610 | 0.0052 | 2.6989 | AT4G01570 | pentatricopeptide (PPR) repeat-containing protein |
| A_84_P21738 | 0.0421 | 2.6969 | AT3G28345 | protein coding |
| A_84_P236753 | 0.0156 | 2.6945 | AT1G75170 | protein coding |
| A_84_P540652 | 0.0309 | 2.693 | AT5G15900 | hypothetical protein |
| A_84_P766880 | 0.0024 | 2.69 | AT5G13770 | pentatricopeptide (PPR) repeat-containing protein |
| A_84_P271340 | 0.0124 | 2.6864 | AT3G09350 | protein coding |
| A_84_P210818 | 0.0287 | 2.6842 | AT3G07210 | protein coding |
| A_84_P67034 | 0.0047 | 2.6839 | AT1G55910 | protein coding |
| A_84_P824785 | 0.001 | 2.6813 | AT3G09920 | protein coding |
| A_84_P19402 | 0.0197 | 2.681 | AT3G55060 | protein coding |
| A_84_P548404 | 0.0208 | 2.6789 | AT3G52115 | protein coding |
| A_84_P21319 | 0.0035 | 2.676 | AT3G61220 | protein coding |
| A_84_P819176 | 0.007 | 2.6712 | AT5G01030 | hypothetical protein |
| A_84_P18401 | 0.0075 | 2.671 | AT3G12580 | protein coding |
| A_84_P824991 | 0.0061 | 2.6677 | AT1G79430 | protein coding |
| A_84_P10513 | 0.0063 | 2.6674 | AT1G63640 | protein coding |
| A_84_P203848 | 0.0265 | 2.6664 | AT2G19380 | RNA recognition motif (RRM)-containing protein |
| A_84_P19808 | 0.0083 | 2.6643 | AT1G27920 | protein coding |
| A_84_P806656 | 0.0266 | 2.6642 | AT5G17920 | ATCIMS (COBALAMIN-INDEPENDENT METHIONINE SYNTHASE); 5-methyltetrahydropteroyltriglutamate-homocystei |
| A_84_P596836 | 0.0275 | 2.6626 | AT3G48260 | protein coding |
| A_84_P15656 | 0.0069 | 2.6622 | AT3G51820 | protein coding |
| A_84_P546306 | 0.0448 | 2.6605 | AT1G60640 | protein coding |
| A_84_P22454 | 0.0286 | 2.6574 | AT5G07240 | IQD24 (IQ-domain 24); calmodulin binding |
| A_84_P276310 | 0.0026 | 2.6563 | AT3G27050 | protein coding |
| A_84_P57010 | 0.0209 | 2.6553 | AT4G37540 | LBD39 (LOB DOMAIN-CONTAINING PROTEIN 39) |
| A_84_P10331 | 0.0221 | 2.6543 | AT5G65820 | pentatricopeptide (PPR) repeat-containing protein |
| A_84_P19537 | 0.0153 | 2.6468 | AT4G29990 | light repressible receptor protein kinase |
| A_84_P19003 | 7.00E-04 | 2.6463 | AT1G02950 | protein coding |
| A_84_P12948 | 0.0204 | 2.6457 | AT4G37930 | SHM1 (SERINE HYDROXYMETHYLTRANSFERASE 1); glycine hydroxymethyltransferase/ poly(U) binding |
| A_84_P17206 | 0.0121 | 2.6452 | AT1G47580 | protein coding |
| A_84_P531636 | 0.034 | 2.6441 | AT4G14840 | hypothetical protein |
| A_84_P562344 | 0.0484 | 2.6387 | AT2G20240 | hypothetical protein |
| A_84_P18354 | 0.0105 | 2.6322 | AT3G20780 | protein coding |
| A_84_P20849 | 0.0395 | 2.6304 | AT1G27480 | protein coding |
| A_84_P18267 | 0.0083 | 2.6279 | AT2G19650 | DC1 domain-containing protein |
| A_84_P182114 | 0.0195 | 2.6255 | AT5G02950 | PWWP domain-containing protein |
| A_84_P832193 | 0.0459 | 2.6254 | AT4G19530 | disease resistance protein (TIR-NBS-LRR class), putative |
| A_84_P21532 | 0.0024 | 2.6237 | AT5G14570 | ATNRT2.7 (ARABIDOPSIS THALIANA HIGH AFFINITY NITRATE TRANSPORTER 2.7); nitrate transmembrane transpo |
| A_84_P184384 | 0.0279 | 2.623 | AT1G68440 | protein coding |
| A_84_P868133 | 0.0455 | 2.6215 | AT5G62890 | permease, putative |
| A_84_P18951 | 0.0044 | 2.6211 | AT1G05170 | protein coding |
| A_84_P532625 | 0.0178 | 2.6159 | AT4G39810 | exonuclease family protein |
| A_84_P524589 | 0.0022 | 2.6112 | AT4G24972 | TPD1 (TAPETUM DETERMINANT 1) |
| A_84_P18583 | 0.0381 | 2.61 | AT4G28160 | hydroxyproline-rich glycoprotein family protein |
| A_84_P10387 | 0.048 | 2.6072 | AT1G71360 | protein coding |
| A_84_P195554 | 0.0454 | 2.6059 | AT5G50100 | hypothetical protein |
| A_84_P21543 | 0.0201 | 2.6057 | AT5G19220 | ADG2 (ADPG PYROPHOSPHORYLASE 2); glucose-1-phosphate adenylyltransferase |
| A_84_P140379 | 3.00E-04 | 2.6051 | AT3G27350 | protein coding |
| A_84_P248035 | 0.0013 | 2.6045 | AT5G53280 | PDV1 (PLASTID DIVISION1) |
| A_84_P228819 | 0.0435 | 2.6041 | AT2G38210 | PDX1L4 (PUTATIVE PDX1-LIKE PROTEIN 4) |
| A_84_P815477 | 0.0104 | 2.5979 | AT3G10420 | protein coding |
| A_84_P139809 | 0.0222 | 2.5925 | AT3G48420 | protein coding |
| A_84_P204928 | 0.0096 | 2.5921 | AT4G01050 | hydroxyproline-rich glycoprotein family protein |
| A_84_P513248 | 0.0075 | 2.5916 | AT5G53020 | hypothetical protein |
| A_84_P831427 | 0.0462 | 2.5912 | AT5G66310 | kinesin motor family protein |
| A_84_P14810 | 0.0432 | 2.587 | AT4G30490 | AFG1-like ATPase family protein |
| A_84_P858932 | 0.0057 | 2.582 | AT4G26650 | RNA recognition motif (RRM)-containing protein |
| A_84_P764162 | 0.0178 | 2.578 | AT4G27660 | hypothetical protein |
| A_84_P850729 | 0.0232 | 2.5779 | AT3G47295 | protein coding |
| A_84_P807419 | 0.0297 | 2.5779 | AT4G25100 | FSD1 (FE SUPEROXIDE DISMUTASE 1); iron superoxide dismutase |
| A_84_P566564 | 0.0171 | 2.5778 | AT1G13220 | protein coding |
| A_84_P305650 | 6.00E-04 | 2.5769 | AT2G30500 | kinase interacting family protein |
| A_84_P818035 | 8.00E-04 | 2.5764 | AT3G52920 | protein coding |
| A_84_P566577 | 0.0362 | 2.5756 | AT1G16730 | protein coding |
| A_84_P853745 | 0.0273 | 2.572 | AT3G06650 | protein coding |
| A_84_P12986 | 0.0122 | 2.5706 | AT5G04020 | calmodulin-binding protein-related (PICBP) |
| A_84_P812285 | 0.0153 | 2.5696 | AT2G42600 | ATPPC2 (PHOSPHOENOLPYRUVATE CARBOXYLASE 2); phosphoenolpyruvate carboxylase |
| A_84_P287150 | 0.0451 | 2.5658 | AT5G56010 | HSP81-3 (Heat shock protein 81-3); ATP binding |
| A_84_P204138 | 0.0117 | 2.5649 | AT1G66080 | protein coding |
| A_84_P139179 | 0.05 | 2.5646 | AT4G29140 | MATE efflux protein-related |
| A_84_P843476 | 0.0374 | 2.5638 | AT2G20190 | ATCLASP/CLASP; binding |
| A_84_P861010 | 0.0237 | 2.563 | AT3G14172 | protein coding |
| A_84_P22974 | 0.0044 | 2.5628 | AT1G19715 | protein coding |
| A_84_P831523 | 0.0247 | 2.5603 | AT1G06220 | protein coding |
| A_84_P23898 | 0.0265 | 2.5597 | AT2G46660 | CYP78A6 (cytochrome P450, family 78, subfamily A, polypeptide 6); oxygen binding |
| A_84_P787143 | 0.0178 | 2.5584 | AT5G49560 | hypothetical protein |
| A_84_P19287 | 0.0018 | 2.5577 | AT3G16060 | protein coding |
| A_84_P162193 | 0.0385 | 2.557 | AT5G62070 | IQD23 (IQ-domain 23); calmodulin binding |
| A_84_P24171 | 5.00E-04 | 2.5566 | AT4G00360 | CYP86A2 (ABERRANT INDUCTION OF TYPE THREE GENES 1); oxygen binding |
| A_84_P17127 | 0.0011 | 2.5526 | AT1G69670 | protein coding |
| A_84_P573880 | 0.0335 | 2.5511 | AT1G79150 | protein coding |
| A_84_P14014 | 0.0162 | 2.5509 | AT5G40210 | nodulin MtN21 family protein |
| A_84_P23557 | 4.00E-04 | 2.5509 | AT5G62430 | CDF1 (CYCLING DOF FACTOR 1); DNA binding / protein binding / transcription factor |
| A_84_P785653 | 0.0367 | 2.55 | AT3G60220 | protein coding |
| A_84_P20472 | 0.0105 | 2.5467 | AT4G27520 | plastocyanin-like domain-containing protein |
| A_84_P537401 | 0.0104 | 2.5465 | AT1G47410 | protein coding |
| A_84_P94969 | 3.00E-04 | 2.5459 | AT5G06980 | hypothetical protein |
| A_84_P197004 | 0.0086 | 2.5443 | AT1G42550 | protein coding |
| A_84_P753373 | 0.0014 | 2.5438 | AT1G76065 | protein coding |
| A_84_P10926 | 0.0437 | 2.5434 | AT3G63140 | protein coding |
| A_84_P16561 | 0.0393 | 2.5426 | AT3G54220 | protein coding |
| A_84_P23925 | 0.0251 | 2.5419 | AT2G02480 | STI (STICHEL); ATP binding / DNA binding / DNA-directed DNA polymerase |
| A_84_P832468 | 0.0024 | 2.5408 | AT1G79280 | protein coding |
| A_84_P112622 | 0.0074 | 2.5399 | AT1G10360 | protein coding |
| A_84_P243405 | 0.048 | 2.5393 | AT2G33180 | hypothetical protein |
| A_84_P58480 | 0.001 | 2.5365 | AT3G01160 | protein coding |
| A_84_P230669 | 0.0018 | 2.5334 | AT4G16490 | armadillo/beta-catenin repeat family protein |
| A_84_P799168 | 0.0031 | 2.5316 | AT1G03060 | protein coding |
| A_84_P15466 | 0.0012 | 2.5309 | AT3G05430 | protein coding |
| A_84_P10431 | 0.0136 | 2.5254 | AT1G80750 | protein coding |
| A_84_P311403 | 0.0017 | 2.5232 | AT2G23420 | nicotinate phosphoribosyltransferase family protein / NAPRTase family protein |
| A_84_P581411 | 3.00E-04 | 2.5191 | AT4G32295 | hypothetical protein |
| A_84_P15460 | 0.0174 | 2.5183 | AT3G03900 | protein coding |
| A_84_P21812 | 0.0238 | 2.5174 | AT1G04240 | protein coding |
| A_84_P11317 | 0.044 | 2.5157 | AT5G34940 | ATGUS3 (ARABIDOPSIS THALIANA GLUCURONIDASE 3); beta-glucuronidase |
| A_84_P271510 | 0.0224 | 2.5141 | AT1G76450 | protein coding |
| A_84_P523278 | 5.00E-04 | 2.5131 | AT1G68910 | protein coding |
| A_84_P15303 | 0.011 | 2.5069 | AT1G55480 | protein coding |
| A_84_P604009 | 0.048 | 2.5067 | AT1G18485 | protein coding |
| A_84_P588374 | 0.0161 | 2.5066 | AT3G44735 | protein coding |
| A_84_P803861 | 0.014 | 2.5054 | AT5G40340 | PWWP domain-containing protein |
| A_84_P786216 | 0.0457 | 2.5041 | AT1G53800 | protein coding |
| A_84_P199584 | 0.0078 | 2.504 | AT3G14200 | protein coding |
| A_84_P18648 | 0.0049 | 2.5009 | AT4G37320 | CYP81D5 (cytochrome P450, family 81, subfamily D, polypeptide 5); oxygen binding |
| A_84_P597910 | 0.0129 | 2.4997 | AT1G68870 | protein coding |
| A_84_P18987 | 0.0223 | 2.4988 | AT1G13170 | protein coding |
| A_84_P536338 | 0.0178 | 2.495 | AT5G56210 | WIP2 (WPP-DOMAIN INTERACTING PROTEIN 2); protein heterodimerization/ protein homodimerization |
| A_84_P18690 | 0.0081 | 2.4947 | AT5G13400 | proton-dependent oligopeptide transport (POT) family protein |
| A_84_P13726 | 0.0286 | 2.4931 | AT3G54420 | protein coding |
| A_84_P16630 | 0.0445 | 2.4929 | AT4G09750 | short-chain dehydrogenase/reductase (SDR) family protein |
| A_84_P810284 | 0.0488 | 2.4922 | AT5G55180 | glycosyl hydrolase family 17 protein |
| A_84_P838052 | 0.0093 | 2.4907 | AT1G04250 | protein coding |
| A_84_P828541 | 0.0259 | 2.4903 | AT1G19970 | protein coding |
| A_84_P85639 | 0.0025 | 2.4877 | AT3G24190 | protein coding |
| A_84_P808064 | 0.0088 | 2.4871 | AT3G50820 | protein coding |
| A_84_P14493 | 0.0237 | 2.4858 | AT2G35650 | ATCSLA07 (CELLULOSE SYNTHASE LIKE); transferase, transferring glycosyl groups |
| A_84_P23418 | 0.0114 | 2.4841 | AT5G12860 | DIT1 (DICARBOXYLATE TRANSPORTER 1); oxoglutarate:malate antiporter |
| A_84_P161603 | 0.0439 | 2.4839 | AT1G33260 | protein coding |
| A_84_P16879 | 0.0191 | 2.483 | AT5G47800 | phototropic-responsive NPH3 family protein |
| A_84_P17668 | 0.0251 | 2.4825 | AT4G35250 | vestitone reductase-related |
| A_84_P587696 | 0.0344 | 2.482 | AT5G03510 | zinc finger (C2H2 type) family protein |
| A_84_P166653 | 0.0024 | 2.482 | AT1G07010 | protein coding |
| A_84_P578709 | 0.0353 | 2.4802 | AT4G25280 | adenylate kinase family protein |
| A_84_P597037 | 0.0235 | 2.4781 | AT1G74790 | protein coding |
| A_84_P18618 | 0.0317 | 2.4765 | AT4G36160 | ANAC076/VND2 (VASCULAR-RELATED NAC-DOMAIN 2); transcription factor |
| A_84_P249845 | 0.0085 | 2.4748 | AT3G46870 | protein coding |
| A_84_P307830 | 0.0192 | 2.4709 | AT2G26570 | hypothetical protein |
| A_84_P19251 | 0.0416 | 2.47 | AT3G02210 | protein coding |
| A_84_P17005 | 0.012 | 2.4696 | AT1G71500 | protein coding |
| A_84_P789714 | 0.0015 | 2.4692 | AT2G32240 | hypothetical protein |
| A_84_P12466 | 0.0397 | 2.4683 | AT1G58290 | protein coding |
| A_84_P13724 | 0.0045 | 2.4682 | AT3G54050 | protein coding |
| A_84_P157605 | 0.0076 | 2.4608 | AT2G31940 | hypothetical protein |
| A_84_P597072 | 0.0219 | 2.4603 | AT2G07787 | hypothetical protein |
| A_84_P130886 | 1.00E-04 | 2.4594 | AT2G34450 | high mobility group (HMG1/2) family protein |
| A_84_P276230 | 0.0072 | 2.458 | AT3G15790 | protein coding |
| A_84_P164433 | 0.002 | 2.4555 | AT4G02920 | hypothetical protein |
| A_84_P18494 | 0.0136 | 2.4554 | AT1G12260 | protein coding |
| A_84_P24179 | 0.0091 | 2.4548 | AT4G02390 | APP (ARABIDOPSIS POLY(ADP-RIBOSE) POLYMERASE); NAD+ ADP-ribosyltransferase |
| A_84_P825864 | 0.0318 | 2.4523 | AT2G45190 | AFO (ABNORMAL FLORAL ORGANS); transcription factor |
| A_84_P750690 | 0.0338 | 2.4522 | AT1G29380 | protein coding |
| A_84_P20288 | 0.0014 | 2.452 | AT3G13620 | protein coding |
| A_84_P836732 | 0.012 | 2.4495 | AT3G14810 | protein coding |
| A_84_P832134 | 0.003 | 2.4483 | AT2G33435 | RNA recognition motif (RRM)-containing protein |
| A_84_P13974 | 0.0211 | 2.4435 | AT1G49580 | protein coding |
| A_84_P863795 | 0.0436 | 2.4419 | AT1G30960 | protein coding |
| A_84_P17013 | 0.0018 | 2.4418 | AT1G19140 | protein coding |
| A_84_P612355 | 0.0239 | 2.4377 | AT3G13600 | protein coding |
| A_84_P74654 | 0.0155 | 2.4356 | AT4G37920 | hypothetical protein |
| A_84_P20265 | 0.0111 | 2.4348 | AT3G15090 | protein coding |
| A_84_P171243 | 0.0174 | 2.4336 | AT5G42070 | hypothetical protein |
| A_84_P17354 | 0.0088 | 2.4316 | AT3G11450 | protein coding |
| A_84_P21845 | 0.0029 | 2.4314 | AT1G10650 | protein coding |
| A_84_P14894 | 0.0303 | 2.4314 | AT5G09760 | pectinesterase family protein |
| A_84_P254210 | 0.0017 | 2.4286 | AT2G27140 | heat shock family protein |
| A_84_P14635 | 0.0345 | 2.4277 | AT1G02460 | protein coding |
| A_84_P85959 | 0.0121 | 2.4235 | AT5G02970 | hydrolase, alpha/beta fold family protein |
| A_84_P296664 | 0.0167 | 2.4233 | AT3G21550 | protein coding |
| A_84_P17160 | 0.0053 | 2.4231 | AT1G70610 | protein coding |
| A_84_P260190 | 0.0266 | 2.4224 | AT2G02820 | MYB88 (myb domain protein 88); DNA binding / transcription factor |
| A_84_P17413 | 0.0215 | 2.4213 | AT3G25230 | protein coding |
| A_84_P755321 | 0.012 | 2.4188 | AT2G41350 | hypothetical protein |
| A_84_P89869 | 0.0328 | 2.4179 | AT1G63310 | protein coding |
| A_84_P115992 | 0.0016 | 2.4172 | AT3G17510 | protein coding |
| A_84_P19932 | 0.0361 | 2.4159 | AT1G11790 | protein coding |
| A_84_P13368 | 0.0419 | 2.4152 | AT1G20160 | protein coding |
| A_84_P186644 | 0.0015 | 2.4138 | AT2G37240 | antioxidant/ oxidoreductase |
| A_84_P763635 | 0.043 | 2.4096 | AT4G16155 | dihydrolipoamide dehydrogenase 2, plastidic / lipoamide dehydrogenase 2 (PTLPD2) |
| A_84_P12374 | 0.0418 | 2.4074 | AT1G15290 | protein coding |
| A_84_P826255 | 0.0224 | 2.4057 | AT1G05230 | protein coding |
| A_84_P215208 | 0.0148 | 2.4047 | AT3G57980 | protein coding |
| A_84_P10984 | 0.0106 | 2.402 | AT4G20070 | ATAAH (ARABIDOPSIS THALIANA ALLANTOATE AMIDOHYDROLASE); allantoate deiminase/ metallopeptidase |
| A_84_P18344 | 0.0309 | 2.3986 | AT3G15800 | protein coding |
| A_84_P18110 | 0.0176 | 2.3982 | AT1G68790 | protein coding |
| A_84_P513620 | 0.028 | 2.3982 | AT2G28105 | hypothetical protein |
| A_84_P512133 | 0.0297 | 2.398 | AT4G15810 | chloroplast outer membrane protein, putative |
| A_84_P19451 | 0.0241 | 2.3974 | AT4G02780 | GA1 (GA REQUIRING 1); ent-copalyl diphosphate synthase |
| A_84_P835789 | 4.00E-04 | 2.3959 | AT2G27950 | hypothetical protein |
| A_84_P609769 | 0.0121 | 2.3935 | AT3G54730 | protein coding |
| A_84_P21186 | 0.0351 | 2.392 | AT3G21670 | protein coding |
| A_84_P212728 | 0.0133 | 2.3918 | AT5G56860 | GNC (GATA, NITRATE-INDUCIBLE, CARBON METABOLISM-INVOLVED); transcription factor |
| A_84_P19504 | 0.043 | 2.3901 | AT4G22730 | leucine-rich repeat transmembrane protein kinase, putative |
| A_84_P574977 | 0.0359 | 2.3896 | AT3G24840 | protein coding |
| A_84_P11005 | 0.0157 | 2.3891 | AT4G25120 | UvrD/REP helicase family protein |
| A_84_P310373 | 0.0312 | 2.3886 | AT2G28150 | hypothetical protein |
| A_84_P818777 | 0.0379 | 2.387 | AT1G74690 | protein coding |
| A_84_P189624 | 0.0188 | 2.3864 | AT1G70890 | protein coding |
| A_84_P51250 | 0.0223 | 2.3853 | AT5G61040 | hypothetical protein |
| A_84_P759140 | 0.0347 | 2.3826 | AT3G16950 | protein coding |
| A_84_P11977 | 0.0077 | 2.3823 | AT4G30950 | FAD6 (FATTY ACID DESATURASE 6); omega-6 fatty acid desaturase |
| A_84_P769455 | 0.0477 | 2.3812 | AT5G39471 | protein binding / zinc ion binding |
| A_84_P21207 | 0.0432 | 2.3807 | AT3G26570 | protein coding |
| A_84_P19745 | 0.0141 | 2.3805 | AT5G55860 | hypothetical protein |
| A_84_P504684 | 0.028 | 2.3743 | AT1G78610 | protein coding |
| A_84_P54840 | 0.0346 | 2.3703 | AT3G19270 | protein coding |
| A_84_P21596 | 0.0312 | 2.3672 | AT5G44530 | subtilase family protein |
| A_84_P21865 | 0.001 | 2.3646 | AT1G76570 | protein coding |
| A_84_P115682 | 0.0341 | 2.3642 | AT3G01810 | protein coding |
| A_84_P835688 | 0.0067 | 2.3634 | AT5G02670 | hypothetical protein |
| A_84_P859476 | 0.0152 | 2.3608 | AT5G23760 | heavy-metal-associated domain-containing protein |
| A_84_P14618 | 0.0032 | 2.3605 | AT3G11964 | protein coding |
| A_84_P23094 | 0.0435 | 2.3599 | AT3G12860 | protein coding |
| A_84_P17857 | 0.0484 | 2.3584 | AT5G57030 | LUT2 (LUTEIN DEFICIENT 2); lycopene epsilon cyclase |
| A_84_P16252 | 0.0135 | 2.3577 | AT1G70410 | protein coding |
| A_84_P209288 | 0.0268 | 2.357 | AT5G63040 | hypothetical protein |
| A_84_P183904 | 0.0491 | 2.3564 | AT5G54180 | PTAC15 (PLASTID TRANSCRIPTIONALLY ACTIVE15) |
| A_84_P822777 | 0.0408 | 2.3542 | AT5G44670 | hypothetical protein |
| A_84_P751136 | 0.0174 | 2.3539 | AT1G58025 | protein coding |
| A_84_P17243 | 0.008 | 2.351 | AT2G33440 | splicing factor family protein |
| A_84_P23545 | 0.0041 | 2.3499 | AT5G59920 | ULI3 (UV-B light insensitive 3) |
| A_84_P14698 | 0.0099 | 2.3492 | AT3G61080 | protein coding |
| A_84_P16596 | 0.0257 | 2.3488 | AT3G62820 | protein coding |
| A_84_P62580 | 0.0394 | 2.3476 | AT2G34670 | proline-rich family protein |
| A_84_P764344 | 0.0109 | 2.3468 | AT4G02400 | hypothetical protein |
| A_84_P515035 | 0.0476 | 2.346 | AT5G54850 | hypothetical protein |
| A_84_P18047 | 0.0162 | 2.3448 | AT1G15100 | protein coding |
| A_84_P813729 | 0.0188 | 2.3432 | AT1G26850 | protein coding |
| A_84_P14488 | 0.0449 | 2.3421 | AT2G25620 | protein phosphatase 2C, putative / PP2C, putative |
| A_84_P14746 | 0.0244 | 2.34 | AT4G11660 | AT-HSFB2B (Arabidopsis thaliana heat shock transcription factor B2B); transcription factor |
| A_84_P526932 | 0.0049 | 2.3391 | AT2G30575 | GAUT5/LGT5 (Galacturonosyltransferase 5); polygalacturonate 4-alpha-galacturonosyltransferase/ trans |
| A_84_P21433 | 0.0331 | 2.3381 | AT4G30860 | ASHR3 (ASH1-RELATED 3); protein binding / zinc ion binding |
| A_84_P12811 | 0.0219 | 2.3376 | AT3G61870 | protein coding |
| A_84_P836040 | 0.0223 | 2.3366 | AT4G32420 | peptidyl-prolyl cis-trans isomerase cyclophilin-type family protein |
| A_84_P827109 | 0.026 | 2.3362 | AT4G14605 | mitochondrial transcription termination factor-related / mTERF-related |
| A_84_P21588 | 0.0129 | 2.3346 | AT5G42270 | VAR1 (VARIEGATED 1); ATP-dependent peptidase/ ATPase/ metallopeptidase |
| A_84_P553355 | 0.0427 | 2.3335 | AT1G52827 | protein coding |
| A_84_P21546 | 0.0014 | 2.3328 | AT5G23120 | HCF136 (High chlorophyll fluorescence 136) |
| A_84_P22878 | 0.0233 | 2.3323 | AT1G62400 | protein coding |
| A_84_P511284 | 0.0345 | 2.3322 | AT4G24910 | hypothetical protein |
| A_84_P12664 | 0.0101 | 2.3318 | AT3G22420 | protein coding |
| A_84_P18449 | 0.0071 | 2.3317 | AT1G06720 | protein coding |
| A_84_P70424 | 0.018 | 2.3317 | AT5G44660 | hypothetical protein |
| A_84_P750294 | 3.00E-04 | 2.3309 | AT1G07390 | protein coding |
| A_84_P51520 | 0.012 | 2.3296 | AT5G17670 | hydrolase, acting on ester bonds |
| A_84_P16999 | 0.0452 | 2.3279 | AT2G04038 | ATBZIP48 (ARABIDOPSIS THALIANA BASIC LEUCINE-ZIPPER 48); DNA binding / transcription factor |
| A_84_P801128 | 0.0265 | 2.3244 | AT1G34000 | protein coding |
| A_84_P23465 | 0.011 | 2.3243 | AT5G38140 | histone-like transcription factor (CBF/NF-Y) family protein |
| A_84_P838144 | 0.0013 | 2.3211 | AT5G65720 | ATNFS1/ATNIFS1/NFS1/NIFS1 (ARABIOPSIS THALIANA NITROGEN FIXATION S HOMOLOG 1); cysteine desulfurase/ |
| A_84_P20218 | 0.0479 | 2.3206 | AT3G08740 | protein coding |
| A_84_P20116 | 0.0061 | 2.3202 | AT2G29410 | MTPB1; efflux transmembrane transporter/ zinc ion transmembrane transporter |
| A_84_P802969 | 0.0475 | 2.3166 | AT4G20360 | AtRABE1b/AtRab8D (Arabidopsis Rab GTPase homolog E1b); translation elongation factor |
| A_84_P553260 | 0.0242 | 2.3153 | AT1G13160 | protein coding |
| A_84_P17956 | 0.0484 | 2.3125 | AT1G15710 | protein coding |
| A_84_P20900 | 0.0331 | 2.3119 | AT1G15960 | protein coding |
| A_84_P292314 | 0.0023 | 2.3119 | AT1G06690 | protein coding |
| A_84_P596990 | 6.00E-04 | 2.3118 | AT1G61215 | protein coding |
| A_84_P18300 | 0.0167 | 2.3107 | AT3G11490 | protein coding |
| A_84_P23507 | 0.0189 | 2.3092 | AT5G49480 | ATCP1 (CA2+-BINDING PROTEIN 1); calcium ion binding |
| A_84_P13730 | 0.0476 | 2.3054 | AT3G55340 | protein coding |
| A_84_P23805 | 0.0124 | 2.3053 | AT1G19710 | protein coding |
| A_84_P11445 | 0.0104 | 2.3048 | AT1G08260 | protein coding |
| A_84_P12066 | 0.0473 | 2.304 | AT5G11410 | protein kinase family protein |
| A_84_P14723 | 0.007 | 2.3013 | AT4G03070 | AOP1 (2-oxoglutarate?dependent dioxygenase 1.1); oxidoreductase, acting on paired donors, with incor |
| A_84_P17689 | 0.0154 | 2.3001 | AT4G15130 | cholinephosphate cytidylyltransferase, putative / phosphorylcholine transferase, putative / CTP:phos |
| A_84_P251015 | 0.0418 | 2.2998 | AT4G14490 | forkhead-associated domain-containing protein / FHA domain-containing protein |
| A_84_P759826 | 0.0027 | 2.2989 | AT3G11950 | protein coding |
| A_84_P130716 | 0.0074 | 2.2977 | AT2G27740 | hypothetical protein |
| A_84_P528550 | 0.0404 | 2.2946 | AT1G51110 | protein coding |
| A_84_P63864 | 1.00E-04 | 2.2934 | AT2G05620 | PGR5 (PROTON GRADIENT REGULATION 5) |
| A_84_P11700 | 0.0318 | 2.2916 | AT1G01420 | protein coding |
| A_84_P559896 | 0.0261 | 2.29 | AT3G44450 | protein coding |
| A_84_P176164 | 0.0042 | 2.2888 | AT2G45300 | 3-phosphoshikimate 1-carboxyvinyltransferase / 5-enolpyruvylshikimate-3-phosphate / EPSP synthase |
| A_84_P17519 | 0.0087 | 2.2868 | AT3G57040 | protein coding |
| A_84_P16991 | 0.0286 | 2.2858 | AT5G58380 | CIPK10 (CBL-INTERACTING PROTEIN KINASE 10); kinase |
| A_84_P832506 | 0.0462 | 2.2856 | AT4G25640 | MATE efflux family protein |
| A_84_P21687 | 7.00E-04 | 2.2841 | AT1G42970 | protein coding |
| A_84_P12678 | 0.0205 | 2.2839 | AT1G53590 | protein coding |
| A_84_P267620 | 0.0389 | 2.2815 | AT1G15180 | protein coding |
| A_84_P20381 | 0.0143 | 2.2812 | AT3G63110 | protein coding |
| A_84_P18746 | 0.0403 | 2.2804 | AT5G40950 | RPL27; structural constituent of ribosome |
| A_84_P835475 | 0.023 | 2.2795 | AT4G17750 | HSF1 (ARABIDOPSIS HEAT SHOCK FACTOR 1); DNA binding / transcription factor |
| A_84_P223109 | 0.003 | 2.2787 | AT2G46420 | hypothetical protein |
| A_84_P592399 | 0.0084 | 2.2764 | AT1G06380 | protein coding |
| A_84_P307590 | 0.0308 | 2.2753 | AT1G45688 | protein coding |
| A_84_P10735 | 0.0267 | 2.2744 | AT3G05060 | protein coding |
| A_84_P19858 | 0.0102 | 2.2733 | AT1G67700 | protein coding |
| A_84_P11831 | 0.0086 | 2.2727 | AT3G53440 | protein coding |
| A_84_P758419 | 0.0161 | 2.271 | AT2G20362 | hypothetical protein |
| A_84_P14312 | 0.0115 | 2.2698 | AT1G48650 | protein coding |
| A_84_P12366 | 0.047 | 2.2697 | AT1G20850 | protein coding |
| A_84_P12110 | 0.0142 | 2.2696 | AT5G35630 | GS2 (GLUTAMINE SYNTHETASE 2); glutamate-ammonia ligase |
| A_84_P168613 | 0.0049 | 2.2694 | AT3G09670 | protein coding |
| A_84_P278190 | 9.00E-04 | 2.2688 | AT5G23890 | hypothetical protein |
| A_84_P12823 | 0.0268 | 2.2681 | AT4G00490 | BAM2/BMY9 (BETA-AMYLASE 2); beta-amylase |
| A_84_P21641 | 0.0137 | 2.268 | AT5G57160 | ATLIG4 (ARABIDOPSIS THALIANA DNA LIGASE IV) |
| A_84_P102136 | 0.0011 | 2.2675 | AT4G18740 | hypothetical protein |
| A_84_P17848 | 0.0209 | 2.2673 | AT5G54280 | ATM2 (ARABIDOPSIS THALIANA MYOSIN 4) |
| A_84_P18688 | 0.0156 | 2.2668 | AT1G18360 | protein coding |
| A_84_P803031 | 0.0049 | 2.2663 | AT2G07728 | hypothetical protein |
| A_84_P764514 | 0.0143 | 2.2661 | AT4G09970 | hypothetical protein |
| A_84_P830366 | 0.0303 | 2.2658 | AT5G37530 | thiF family protein |
| A_84_P249465 | 0.0408 | 2.2648 | AT1G19520 | protein coding |
| A_84_P17953 | 0.0454 | 2.2634 | AT1G12420 | protein coding |
| A_84_P136215 | 0.0268 | 2.2603 | AT1G32080 | protein coding |
| A_84_P10031 | 0.0156 | 2.2593 | AT4G18260 | cytochrome B561-related |
| A_84_P506441 | 0.0056 | 2.2584 | AT1G75310 | protein coding |
| A_84_P834919 | 0.0095 | 2.2572 | AT1G17210 | protein coding |
| A_84_P166453 | 0.0077 | 2.2568 | AT1G27760 | protein coding |
| A_84_P13396 | 0.043 | 2.2513 | AT1G02280 | protein coding |
| A_84_P20882 | 0.0334 | 2.2512 | AT1G32730 | protein coding |
| A_84_P751783 | 0.004 | 2.2489 | AT1G02335 | protein coding |
| A_84_P10992 | 0.0044 | 2.2486 | AT4G21990 | APR3 (APS REDUCTASE 3) |
| A_84_P146548 | 0.0095 | 2.248 | AT2G35840 | sucrose-phosphatase 1 (SPP1) |
| A_84_P22919 | 0.0332 | 2.2452 | AT2G33530 | SCPL46 (serine carboxypeptidase-like 46); serine carboxypeptidase |
| A_84_P16762 | 0.0041 | 2.2427 | AT5G02280 | synbindin, putative |
| A_84_P23194 | 0.0186 | 2.2415 | AT3G56940 | protein coding |
| A_84_P750155 | 0.0228 | 2.2397 | AT1G20970 | protein coding |
| A_84_P15259 | 0.0161 | 2.2365 | AT1G06490 | protein coding |
| A_84_P19411 | 0.0077 | 2.2339 | AT3G57060 | protein coding |
| A_84_P823363 | 0.0468 | 2.2328 | AT5G57960 | GTP-binding family protein |
| A_84_P805087 | 0.0051 | 2.2323 | AT1G07920 | protein coding |
| A_84_P826226 | 0.0481 | 2.2323 | AT4G14200 | hypothetical protein |
| A_84_P123262 | 0.0079 | 2.2301 | AT1G61740 | protein coding |
| A_84_P809942 | 0.0057 | 2.2263 | AT3G61240 | protein coding |
| A_84_P853373 | 0.0054 | 2.225 | AT2G34040 | apoptosis inhibitory 5 (API5) family protein |
| A_84_P11857 | 9.00E-04 | 2.2205 | AT1G05760 | protein coding |
| A_84_P848800 | 0.0346 | 2.2204 | AT2G35940 | BLH1 (embryo sac development arrest 29) |
| A_84_P834927 | 0.0118 | 2.2195 | AT1G63850 | protein coding |
| A_84_P15292 | 2.00E-04 | 2.217 | AT1G13460 | protein coding |
| A_84_P12077 | 0.0116 | 2.2166 | AT5G15240 | amino acid transporter family protein |
| A_84_P23529 | 0.0474 | 2.216 | AT5G55920 | nucleolar protein, putative |
| A_84_P838155 | 0.019 | 2.214 | AT2G18220 | hypothetical protein |
| A_84_P11706 | 0.0291 | 2.2139 | AT3G05830 | protein coding |
| A_84_P137629 | 0.0053 | 2.2127 | AT5G64170 | dentin sialophosphoprotein-related |
| A_84_P750185 | 0.033 | 2.2124 | AT1G15910 | protein coding |
| A_84_P19279 | 0.0309 | 2.2113 | AT3G07050 | protein coding |
| A_84_P543437 | 0.0073 | 2.2086 | AT5G63320 | hypothetical protein |
| A_84_P18261 | 0.0264 | 2.2082 | AT2G37420 | kinesin motor protein-related |
| A_84_P135535 | 0.0328 | 2.2079 | AT2G35480 | hypothetical protein |
| A_84_P758327 | 0.0426 | 2.2078 | AT2G40711 | hypothetical protein |
| A_84_P855479 | 0.0475 | 2.2069 | AT4G37820 | hypothetical protein |
| A_84_P21616 | 0.0243 | 2.2066 | AT5G49700 | DNA-binding protein-related |
| A_84_P16212 | 0.0329 | 2.2061 | AT1G74470 | protein coding |
| A_84_P10321 | 0.0078 | 2.2055 | AT5G63380 | 4-coumarate--CoA ligase family protein / 4-coumaroyl-CoA synthase family protein |
| A_84_P20865 | 0.0247 | 2.2049 | AT1G04420 | protein coding |
| A_84_P20685 | 0.0121 | 2.2048 | AT5G54100 | band 7 family protein |
| A_84_P532171 | 0.0192 | 2.2033 | AT1G77620 | protein coding |
| A_84_P81399 | 0.0052 | 2.2024 | AT1G63170 | protein coding |
| A_84_P525206 | 0.0145 | 2.2015 | AT2G42820 | HVA22F (HVA22-LIKE PROTEIN F) |
| A_84_P561855 | 0.0132 | 2.2013 | AT4G39050 | kinesin-related protein (MKRP2) |
| A_84_P23248 | 0.0252 | 2.2012 | AT4G08850 | leucine-rich repeat family protein / protein kinase family protein |
| A_84_P15474 | 0.0284 | 2.2011 | AT3G01990 | protein coding |
| A_84_P277480 | 0.0411 | 2.1993 | AT1G79200 | protein coding |
| A_84_P11371 | 0.0076 | 2.1991 | AT1G64680 | protein coding |
| A_84_P805476 | 0.0025 | 2.1967 | AT1G67090 | protein coding |
| A_84_P819621 | 0.0014 | 2.1963 | AT4G18390 | TCP family transcription factor, putative |
| A_84_P784282 | 8.00E-04 | 2.196 | AT4G16630 | DEAD/DEAH box helicase, putative (RH28) |
| A_84_P764417 | 0.0216 | 2.1959 | AT4G15820 | wound-responsive protein-related |
| A_84_P513798 | 0.0054 | 2.1959 | AT3G44380 | protein coding |
| A_84_P822749 | 0.003 | 2.1958 | AT1G18660 | protein coding |
| A_84_P22514 | 0.0212 | 2.1947 | AT5G36700 | ATPGLP1/PGLP1 (2-PHOSPHOGLYCOLATE PHOSPHATASE 1) |
| A_84_P69524 | 0.016 | 2.1931 | AT5G38690 | hypothetical protein |
| A_84_P750263 | 0.0013 | 2.1895 | AT1G17230 | protein coding |
| A_84_P536757 | 0.0312 | 2.188 | AT2G46160 | zinc finger (C3HC4-type RING finger) family protein |
| A_84_P829972 | 0.0019 | 2.1879 | AT1G66540 | protein coding |
| A_84_P11381 | 0.0047 | 2.187 | AT1G09780 | protein coding |
| A_84_P220058 | 0.0257 | 2.1855 | AT3G12920 | protein coding |
| A_84_P234663 | 0.0418 | 2.185 | AT5G55950 | transporter-related |
| A_84_P817271 | 0.0338 | 2.1844 | AT4G36530 | hydrolase, alpha/beta fold family protein |
| A_84_P14418 | 0.0305 | 2.1822 | AT2G40230 | transferase family protein |
| A_84_P200614 | 0.03 | 2.1804 | AT3G28460 | protein coding |
| A_84_P568295 | 0.0291 | 2.1765 | AT4G11400 | ARID/BRIGHT DNA-binding domain-containing protein / ELM2 domain-containing protein / Myb-like DNA-bi |
| A_84_P98456 | 0.0196 | 2.1746 | AT1G01030 | protein coding |
| A_84_P218248 | 0.0455 | 2.1745 | AT2G01590 | hypothetical protein |
| A_84_P15461 | 0.0341 | 2.1705 | AT3G05370 | protein coding |
| A_84_P822799 | 0.0318 | 2.1702 | AT5G45590 | structural constituent of ribosome |
| A_84_P54890 | 0.0431 | 2.1677 | AT2G38300 | DNA binding / transcription factor |
| A_84_P15654 | 0.0042 | 2.1664 | AT3G44850 | protein coding |
| A_84_P833346 | 0.017 | 2.1654 | AT1G77800 | protein coding |
| A_84_P255410 | 0.0213 | 2.164 | AT2G43340 | hypothetical protein |
| A_84_P266090 | 0.0068 | 2.1618 | AT1G12650 | protein coding |
| A_84_P22424 | 0.0121 | 2.1614 | AT4G16660 | heat shock protein 70, putative / HSP70, putative |
| A_84_P842442 | 0.0046 | 2.1609 | AT5G17890 | LIM domain-containing protein / disease resistance protein-related |
| A_84_P14526 | 0.0317 | 2.1604 | AT3G09150 | protein coding |
| A_84_P21781 | 0.0199 | 2.1588 | AT1G70000 | protein coding |
| A_84_P20803 | 0.004 | 2.1566 | AT1G11340 | protein coding |
| A_84_P855199 | 0.0177 | 2.1559 | AT5G13650 | elongation factor family protein |
| A_84_P206918 | 0.0043 | 2.1539 | AT1G75100 | protein coding |
| A_84_P15076 | 0.0035 | 2.1537 | AT5G20270 | HHP1 (HEPTAHELICAL TRANSMEMBRANE PROTEIN1) |
| A_84_P19369 | 0.0109 | 2.1533 | AT3G47500 | protein coding |
| A_84_P838191 | 0.0397 | 2.1529 | AT1G76720 | protein coding |
| A_84_P797554 | 0.0296 | 2.1525 | AT1G74260 | protein coding |
| A_84_P545078 | 0.0235 | 2.1522 | AT5G13340 | hypothetical protein |
| A_84_P155585 | 0.0308 | 2.1514 | AT2G21385 | hypothetical protein |
| A_84_P22292 | 0.0075 | 2.1513 | AT4G04020 | FIB (FIBRILLIN); structural molecule |
| A_84_P766109 | 0.005 | 2.1485 | AT5G65770 | LINC4 (LITTLE NUCLEI4) |
| A_84_P845074 | 0.0197 | 2.1485 | AT4G32330 | hypothetical protein |
| A_84_P73254 | 0.0165 | 2.1479 | AT1G26220 | protein coding |
| A_84_P852812 | 0.024 | 2.1471 | AT5G44750 | REV1 (Reversionless 1); damaged DNA binding / magnesium ion binding / nucleotidyltransferase |
| A_84_P19310 | 0.0107 | 2.1455 | AT3G12780 | protein coding |
| A_84_P19683 | 0.0205 | 2.1443 | AT5G38720 | hypothetical protein |
| A_84_P50780 | 0.0227 | 2.1434 | AT5G67370 | hypothetical protein |
| A_84_P23201 | 0.0088 | 2.1432 | AT3G58720 | protein coding |
| A_84_P13551 | 0.0152 | 2.1431 | AT2G18990 | TXND9 (THIOREDOXIN DOMAIN-CONTAINING PROTEIN 9 HOMOLOG) |
| A_84_P17558 | 0.0023 | 2.1422 | AT4G02560 | LD (LUMINIDEPENDENS); transcription factor |
| A_84_P184664 | 0.0235 | 2.1418 | AT5G26742 | EMB1138 (EMBRYO DEFECTIVE 1138); ATP binding / ATP-dependent helicase |
| A_84_P130546 | 5.00E-04 | 2.1416 | AT3G15470 | protein coding |
| A_84_P233869 | 0.0325 | 2.1402 | AT4G01150 | hypothetical protein |
| A_84_P17300 | 0.049 | 2.1401 | AT2G45120 | zinc finger (C2H2 type) family protein |
| A_84_P838010 | 0.0029 | 2.1396 | AT1G79830 | protein coding |
| A_84_P815904 | 0.002 | 2.1391 | AT3G42170 | pseudo |
| A_84_P790574 | 0.0272 | 2.1389 | AT1G77250 | protein coding |
| A_84_P10352 | 5.00E-04 | 2.1387 | AT1G27910 | protein coding |
| A_84_P129746 | 0.0048 | 2.1374 | AT1G06910 | protein coding |
| A_84_P23846 | 0.0392 | 2.1366 | AT2G47180 | ATGOLS1 (ARABIDOPSIS THALIANA GALACTINOL SYNTHASE 1); transferase, transferring hexosyl groups |
| A_84_P594406 | 0.0389 | 2.1361 | AT2G07772 | hypothetical protein |
| A_84_P754730 | 0.0165 | 2.1346 | AT1G16489 | misc_RNA |
| A_84_P23411 | 0.0171 | 2.1343 | AT5G10480 | PAS2 (PASTICCINO 2) |
| A_84_P831180 | 0.0442 | 2.1336 | AT1G77680 | protein coding |
| A_84_P836418 | 0.0304 | 2.1332 | AT5G47480 | hypothetical protein |
| A_84_P124122 | 0.0027 | 2.1322 | AT5G67240 | exonuclease |
| A_84_P834855 | 0.0221 | 2.1309 | AT2G16485 | zinc ion binding |
| A_84_P21006 | 0.0029 | 2.1293 | AT2G43750 | OASB (O-ACETYLSERINE (THIOL) LYASE B); cysteine synthase |
| A_84_P231019 | 0.0044 | 2.1291 | AT3G44110 | protein coding |
| A_84_P758290 | 0.0451 | 2.1289 | AT2G07815 | hypothetical protein |
| A_84_P11591 | 0.0016 | 2.1289 | AT2G23340 | AP2 domain-containing transcription factor, putative |
| A_84_P808903 | 0.0223 | 2.1288 | AT2G04390 | 40S ribosomal protein S17 (RPS17A) |
| A_84_P812434 | 0.0206 | 2.1277 | AT1G22882 | protein coding |
| A_84_P819419 | 0.0239 | 2.1258 | AT1G58602 | protein coding |
| A_84_P244115 | 0.0112 | 2.1241 | AT3G58110 | protein coding |
| A_84_P13986 | 0.0048 | 2.1197 | AT5G24970 | ABC1 family protein |
| A_84_P23054 | 0.0053 | 2.1115 | AT3G09540 | protein coding |
| A_84_P813108 | 0.0088 | 2.1108 | AT2G04039 | hypothetical protein |
| A_84_P19873 | 0.034 | 2.1107 | AT1G67230 | protein coding |
| A_84_P580899 | 0.0134 | 2.1082 | AT1G50575 | protein coding |
| A_84_P812218 | 0.0142 | 2.1078 | AT1G78900 | protein coding |
| A_84_P805395 | 0.0322 | 2.1055 | AT3G23820 | protein coding |
| A_84_P598300 | 0.0334 | 2.1053 | AT4G13650 | pentatricopeptide (PPR) repeat-containing protein |
| A_84_P123932 | 0.007 | 2.1039 | AT1G79510 | protein coding |
| A_84_P830735 | 0.0447 | 2.1039 | AT4G32920 | glycine-rich protein |
| A_84_P729473 | 0.0493 | 2.1034 | AT1G50055 | misc_RNA |
| A_84_P560054 | 0.0075 | 2.1018 | AT4G29905 | hypothetical protein |
| A_84_P217768 | 0.0045 | 2.1006 | AT3G24150 | protein coding |
| A_84_P19774 | 0.035 | 2.1001 | AT5G62670 | AHA11 (ARABIDOPSIS H(+)-ATPASE 11); ATPase |
| A_84_P22251 | 0.0489 | 2.0997 | AT3G57560 | protein coding |
| A_84_P78659 | 0.0089 | 2.0977 | AT5G04830 | hypothetical protein |
| A_84_P19044 | 0.0335 | 2.0967 | AT1G21440 | protein coding |
| A_84_P805443 | 0.0393 | 2.0966 | AT5G38420 | ribulose bisphosphate carboxylase small chain 2B / RuBisCO small subunit 2B (RBCS-2B) (ATS2B) |
| A_84_P849984 | 0.0112 | 2.0965 | AT3G56080 | protein coding |
| A_84_P69514 | 0.04 | 2.0952 | AT3G48200 | protein coding |
| A_84_P815442 | 5.00E-04 | 2.0924 | AT1G63940 | protein coding |
| A_84_P10985 | 0.0196 | 2.0921 | AT1G29200 | protein coding |
| A_84_P591014 | 0.0091 | 2.0918 | AT3G19080 | protein coding |
| A_84_P10522 | 0.0133 | 2.0917 | AT1G77720 | protein coding |
| A_84_P19882 | 0.0253 | 2.0901 | AT1G03440 | protein coding |
| A_84_P12084 | 0.0044 | 2.0888 | AT5G18620 | CHR17 (CHROMATIN REMODELING FACTOR17); DNA-dependent ATPase |
| A_84_P832577 | 0.0016 | 2.0851 | AT5G22820 | binding |
| A_84_P12112 | 0.019 | 2.085 | AT5G36890 | glycosyl hydrolase family 1 protein |
| A_84_P13021 | 0.0383 | 2.0833 | AT5G14640 | protein kinase family protein |
| A_84_P16518 | 0.0124 | 2.083 | AT1G02720 | protein coding |
| A_84_P10003 | 0.0058 | 2.0803 | AT4G05090 | inositol monophosphatase family protein |
| A_84_P116142 | 0.036 | 2.0793 | AT3G27860 | protein coding |
| A_84_P765008 | 0.0311 | 2.0787 | AT4G30975 | misc_RNA |
| A_84_P828413 | 0.0161 | 2.0782 | AT2G32250 | FRS2 (FAR1-RELATED SEQUENCE 2); zinc ion binding |
| A_84_P12881 | 0.0464 | 2.076 | AT4G21750 | ATML1 (MERISTEM LAYER 1); DNA binding |
| A_84_P17657 | 0.0164 | 2.0738 | AT4G32730 | PC-MYB1 (myb domain protein 3R1); DNA binding / transcription factor |
| A_84_P161933 | 0.0016 | 2.0737 | AT1G50660 | protein coding |
| A_84_P12002 | 0.0474 | 2.0727 | AT4G37670 | GCN5-related N-acetyltransferase (GNAT) family protein / amino acid kinase family protein |
| A_84_P18027 | 0.0401 | 2.0717 | AT1G09310 | protein coding |
| A_84_P11954 | 0.0483 | 2.0716 | AT4G25960 | PGP2 (P-GLYCOPROTEIN 2); ATPase, coupled to transmembrane movement of substances |
| A_84_P95079 | 0.0044 | 2.0716 | AT5G38610 | invertase/pectin methylesterase inhibitor family protein |
| A_84_P768803 | 0.0044 | 2.0707 | AT5G64572 | misc_RNA |
| A_84_P24024 | 0.0231 | 2.0696 | AT3G21510 | protein coding |
| A_84_P11687 | 0.0034 | 2.0689 | AT3G01350 | protein coding |
| A_84_P22582 | 0.0143 | 2.0672 | AT5G55630 | KCO1 (TWO PORE K CHANNEL); calcium-activated potassium channel/ outward rectifier potassium channel |
| A_84_P818881 | 0.0164 | 2.067 | AT3G26710 | protein coding |
| A_84_P270770 | 0.0127 | 2.067 | AT2G36500 | CBS domain-containing protein / octicosapeptide/Phox/Bemp1 (PB1) domain-containing protein |
| A_84_P144679 | 0.0104 | 2.0668 | AT3G51950 | protein coding |
| A_84_P150518 | 0.0478 | 2.0667 | AT4G28220 | NDB1 (NAD(P)H DEHYDROGENASE B1); NADH dehydrogenase/ disulfide oxidoreductase |
| A_84_P17992 | 0.029 | 2.0642 | AT1G08790 | protein coding |
| A_84_P23177 | 9.00E-04 | 2.0631 | AT3G53240 | protein coding |
| A_84_P249405 | 0.0089 | 2.0627 | AT1G50890 | protein coding |
| A_84_P261010 | 0.0119 | 2.0626 | AT5G42810 | ATIPK1 (Inositol-pentakisphosphate 2-kinase 1); inositol pentakisphosphate 2-kinase/ inositol tetrak |
| A_84_P854678 | 0.0143 | 2.062 | AT1G48520 | protein coding |
| A_84_P793464 | 0.0212 | 2.0608 | AT5G01920 | STN8 (state transition 8); kinase |
| A_84_P822197 | 0.0123 | 2.0608 | AT2G44970 | lipase-related |
| A_84_P206238 | 0.0029 | 2.0605 | AT5G22060 | ATJ2 (Arabidopsis thaliana DnaJ homologue 2) |
| A_84_P59160 | 0.0255 | 2.0597 | AT5G23780 | agenet domain-containing protein |
| A_84_P852838 | 0.0031 | 2.0596 | AT1G09840 | protein coding |
| A_84_P15763 | 0.0163 | 2.0585 | AT1G50450 | protein coding |
| A_84_P15610 | 0.0181 | 2.0583 | AT3G53320 | protein coding |
| A_84_P184084 | 0.0485 | 2.0569 | AT5G10470 | kinesin motor protein-related |
| A_84_P12188 | 0.0063 | 2.0568 | AT5G57990 | UBP23 (UBIQUITIN-SPECIFIC PROTEASE 23); ubiquitin-specific protease |
| A_84_P602443 | 0.0309 | 2.0562 | AT2G21350 | hypothetical protein |
| A_84_P820116 | 0.0057 | 2.0561 | AT5G45490 | disease resistance protein-related |
| A_84_P14259 | 0.0015 | 2.0556 | AT1G10740 | protein coding |
| A_84_P11219 | 1.00E-04 | 2.054 | AT5G50990 | hypothetical protein |
| A_84_P19951 | 0.009 | 2.0537 | AT1G22450 | protein coding |
| A_84_P297994 | 0.0225 | 2.0536 | AT1G52510 | protein coding |
| A_84_P752946 | 0.0039 | 2.0531 | AT1G18750 | protein coding |
| A_84_P784980 | 0.0368 | 2.0528 | AT4G00180 | YAB3 (YABBY3) |
| A_84_P13530 | 0.0168 | 2.0525 | AT2G24190 | short-chain dehydrogenase/reductase (SDR) family protein |
| A_84_P10150 | 0.0362 | 2.052 | AT5G03940 | FFC (FIFTY-FOUR CHLOROPLAST HOMOLOGUE); 7S RNA binding / GTP binding / mRNA binding |
| A_84_P20665 | 0.0205 | 2.0516 | AT5G48300 | ADG1 (ADP GLUCOSE PYROPHOSPHORYLASE SMALL SUBUNIT 1); glucose-1-phosphate adenylyltransferase |
| A_84_P21602 | 0.002 | 2.0514 | AT5G46070 | GTP binding / GTPase |
| A_84_P21716 | 0.0027 | 2.0508 | AT2G17900 | SDG37 (SET DOMAIN GROUP 37); zinc ion binding |
| A_84_P11462 | 0.0303 | 2.049 | AT1G73690 | protein coding |
| A_84_P20522 | 0.0215 | 2.0488 | AT4G39950 | CYP79B2 (cytochrome P450, family 79, subfamily B, polypeptide 2); oxygen binding |
| A_84_P11359 | 0.0218 | 2.0463 | AT1G64490 | protein coding |
| A_84_P150998 | 3.00E-04 | 2.0462 | AT2G30800 | HVT1 (HELICASE IN VASCULAR TISSUE AND TAPETUM); ATP binding / helicase/ nucleic acid binding |
| A_84_P577905 | 0.0086 | 2.0454 | AT5G11460 | senescence-associated protein-related |
| A_84_P12818 | 0.0218 | 2.0454 | AT3G63410 | protein coding |
| A_84_P601401 | 0.037 | 2.0454 | AT1G49405 | protein coding |
| A_84_P220638 | 0.002 | 2.044 | AT3G45210 | protein coding |
| A_84_P230579 | 0.0092 | 2.044 | AT3G57070 | protein coding |
| A_84_P834140 | 0.049 | 2.0433 | AT3G24080 | protein coding |
| A_84_P19563 | 0.0106 | 2.0431 | AT4G35970 | APX5; L-ascorbate peroxidase/ peroxidase |
| A_84_P13664 | 0.0249 | 2.0414 | AT3G21390 | protein coding |
| A_84_P201848 | 0.0066 | 2.0413 | AT3G48710 | protein coding |
| A_84_P784376 | 0.0327 | 2.0412 | AT3G61550 | protein coding |
| A_84_P856672 | 0.0053 | 2.0402 | AT1G47530 | protein coding |
| A_84_P19067 | 0.027 | 2.0399 | AT1G32550 | protein coding |
| A_84_P10412 | 0.0362 | 2.0377 | AT1G03830 | protein coding |
| A_84_P763834 | 0.046 | 2.0375 | AT4G39235 | hypothetical protein |
| A_84_P17551 | 1.00E-04 | 2.0371 | AT4G00880 | auxin-responsive family protein |
| A_84_P19485 | 0.036 | 2.0367 | AT4G17880 | basic helix-loop-helix (bHLH) family protein |
| A_84_P809488 | 0.0036 | 2.0361 | AT3G02470 | protein coding |
| A_84_P802675 | 0.0445 | 2.0354 | AT1G20340 | protein coding |
| A_84_P200434 | 0.0131 | 2.0354 | AT4G20170 | hypothetical protein |
| A_84_P843180 | 9.00E-04 | 2.0347 | AT1G58250 | protein coding |
| A_84_P197514 | 0.0133 | 2.032 | AT5G55580 | mitochondrial transcription termination factor family protein / mTERF family protein |
| A_84_P806129 | 0.0041 | 2.0307 | AT5G38410 | ribulose bisphosphate carboxylase small chain 3B / RuBisCO small subunit 3B (RBCS-3B) (ATS3B) |
| A_84_P233279 | 0.0425 | 2.0303 | AT1G64530 | protein coding |
| A_84_P274710 | 0.0415 | 2.0296 | AT4G14510 | hypothetical protein |
| A_84_P185644 | 0.0343 | 2.0295 | AT3G11690 | protein coding |
| A_84_P20071 | 0.0144 | 2.0294 | AT2G31750 | UGT74D1 (UDP-GLUCOSYL TRANSFERASE 74D1); UDP-glycosyltransferase/ abscisic acid glucosyltransferase/ |
| A_84_P803652 | 0.0374 | 2.0293 | AT1G37130 | protein coding |
| A_84_P12935 | 0.0015 | 2.0286 | AT4G34250 | fatty acid elongase, putative |
| A_84_P836947 | 0.0029 | 2.028 | AT4G14920 | PHD finger transcription factor, putative |
| A_84_P834503 | 0.0104 | 2.0276 | AT5G20490 | XIK (Myosin-like protein XIK); motor/ protein binding |
| A_84_P233129 | 0.0271 | 2.0272 | AT3G23070 | protein coding |
| A_84_P752982 | 0.0182 | 2.0272 | AT1G05320 | protein coding |
| A_84_P542301 | 4.00E-04 | 2.0248 | AT4G21060 | galactosyltransferase family protein |
| A_84_P569682 | 0.0057 | 2.0236 | AT3G52340 | protein coding |
| A_84_P75354 | 0.0076 | 2.0235 | AT3G51510 | protein coding |
| A_84_P166753 | 0.0362 | 2.0234 | AT5G38520 | hydrolase, alpha/beta fold family protein |
| A_84_P284920 | 0.0073 | 2.0227 | AT3G01060 | protein coding |
| A_84_P15492 | 0.0171 | 2.0223 | AT3G02760 | protein coding |
| A_84_P20875 | 0.0188 | 2.0214 | AT1G64860 | protein coding |
| A_84_P760552 | 0.0213 | 2.0196 | AT3G23900 | protein coding |
| A_84_P787450 | 0.0469 | 2.0194 | AT5G11780 | hypothetical protein |
| A_84_P753683 | 0.0443 | 2.017 | AT1G24577 | protein coding |
| A_84_P22016 | 0.0013 | 2.0167 | AT2G26250 | FDH (FIDDLEHEAD); acyltransferase |
| A_84_P819339 | 0.0392 | 2.0157 | AT3G44670 | protein coding |
| A_84_P14083 | 0.0165 | 2.0151 | AT5G59250 | sugar transporter family protein |
| A_84_P830423 | 0.0063 | 2.0151 | AT3G44200 | protein coding |
| A_84_P791064 | 0.0318 | 2.0151 | AT5G50290 | hypothetical protein |
| A_84_P812096 | 0.0141 | 2.013 | AT1G79550 | protein coding |
| A_84_P16069 | 0.0246 | 2.0128 | AT1G02330 | protein coding |
| A_84_P610677 | 0.0401 | 2.0125 | AT3G57200 | protein coding |
| A_84_P804241 | 0.0401 | 2.0121 | AT1G03370 | protein coding |
| A_84_P10778 | 0.0334 | 2.0082 | AT3G16140 | protein coding |
| A_84_P165523 | 0.0103 | 2.0082 | AT4G28706 | pfkB-type carbohydrate kinase family protein |
| A_84_P586204 | 0.0036 | 2.0079 | AT1G16800 | protein coding |
| A_84_P847389 | 0.026 | 2.0073 | AT1G18190 | protein coding |
| A_84_P238443 | 0.0016 | 2.0064 | AT2G39170 | hypothetical protein |
| A_84_P22953 | 0.0262 | 2.0064 | AT2G25140 | CLPB-M/CLPB4/HSP98.7 (HEAT SHOCK PROTEIN 98.7); ATP binding / ATPase |
| A_84_P511073 | 0.0078 | 2.0056 | AT3G10440 | protein coding |
| A_84_P215078 | 0.0422 | 2.0047 | AT5G51840 | hypothetical protein |
| A_84_P165613 | 0.0395 | 2.0042 | AT4G29410 | 60S ribosomal protein L28 (RPL28C) |
| A_84_P15148 | 0.0099 | 2.0022 | AT1G61190 | protein coding |
| A_84_P76094 | 0.0086 | 2.0015 | AT3G52220 | protein coding |
| A_84_P853833 | 0.0376 | 2.0001 | AT5G06440 | hypothetical protein |
| A_84_P817774 | 0.0454 | 0.4996 | AT5G61900 | BON1 (BONZAI1); calcium-dependent phospholipid binding |
| A_84_P828862 | 0.0271 | 0.4993 | AT5G45370 | nodulin-related / integral membrane family protein |
| A_84_P762765 | 0.0303 | 0.4992 | AT3G27416 | protein coding |
| A_84_P760282 | 0.037 | 0.4992 | AT3G57765 | ncRNA |
| A_84_P760189 | 0.0374 | 0.4991 | AT3G43340 | protein coding |
| A_84_P590207 | 0.0019 | 0.4988 | AT3G58270 | protein coding |
| A_84_P16649 | 0.0304 | 0.4978 | AT1G17590 | protein coding |
| A_84_P15554 | 8.00E-04 | 0.4976 | AT3G22180 | protein coding |
| A_84_P19587 | 0.0088 | 0.4972 | AT1G19370 | protein coding |
| A_84_P86169 | 0.0023 | 0.4962 | AT4G18610 | hypothetical protein |
| A_84_P196604 | 0.0479 | 0.4955 | AT2G40530 | hypothetical protein |
| A_84_P752636 | 0.0166 | 0.4954 | AT1G15400 | protein coding |
| A_84_P11537 | 0.0026 | 0.495 | AT1G16670 | protein coding |
| A_84_P828905 | 0.0171 | 0.495 | AT2G44450 | glycosyl hydrolase family 1 protein |
| A_84_P784428 | 0.0053 | 0.4947 | AT4G19160 | hypothetical protein |
| A_84_P575740 | 0.0433 | 0.4947 | AT2G35550 | ATBPC7/BBR/BPC7/BPC7 (BASIC PENTACYSTEINE 7) |
| A_84_P17925 | 0.0234 | 0.4946 | AT5G11060 | KNAT4 (KNOTTED1-LIKE HOMEOBOX GENE 4); transcription factor |
| A_84_P706642 | 0.0425 | 0.4941 | AT5G10946 | hypothetical protein |
| A_84_P819742 | 0.0021 | 0.4939 | AT1G14685 | protein coding |
| A_84_P12261 | 0.0338 | 0.4935 | AT5G04260 | thioredoxin family protein |
| A_84_P814660 | 0.0366 | 0.493 | AT5G39130 | germin-like protein, putative |
| A_84_P11674 | 0.0016 | 0.4929 | AT2G28940 | protein kinase family protein |
| A_84_P819872 | 0.0225 | 0.4929 | AT4G26670 | mitochondrial import inner membrane translocase subunit Tim17/Tim22/Tim23 family protein |
| A_84_P593146 | 0.0327 | 0.4926 | AT5G40570 | surfeit locus protein 2 family protein / SURF2 family protein |
| A_84_P806445 | 0.0463 | 0.4925 | AT4G16830 | nuclear RNA-binding protein (RGGA) |
| A_84_P831327 | 0.0334 | 0.4922 | AT2G45500 | ATP binding |
| A_84_P10977 | 0.0085 | 0.4916 | AT4G18270 | ATTRANS11 (Arabidopsis thaliana translocase 11); catalytic |
| A_84_P22232 | 5.00E-04 | 0.4914 | AT1G06760 | protein coding |
| A_84_P13982 | 0.0163 | 0.4907 | AT5G23870 | pectinacetylesterase family protein |
| A_84_P822774 | 0.0312 | 0.4906 | AT3G50210 | protein coding |
| A_84_P12840 | 0.0212 | 0.4906 | AT4G05330 | AGD13 (ARF-GAP DOMAIN 13); ARF GTPase activator/ zinc ion binding |
| A_84_P12621 | 0.0317 | 0.4905 | AT2G47060 | serine/threonine protein kinase, putative |
| A_84_P261600 | 0.0144 | 0.4904 | AT1G14340 | protein coding |
| A_84_P21005 | 0.0367 | 0.4898 | AT2G39890 | ProT1 (PROLINE TRANSPORTER 1) |
| A_84_P586725 | 0.0092 | 0.4898 | AT4G16215 | hypothetical protein |
| A_84_P260330 | 0.0234 | 0.4896 | AT1G72630 | protein coding |
| A_84_P786308 | 0.0197 | 0.4891 | AT3G17210 | protein coding |
| A_84_P10548 | 0.0066 | 0.489 | AT1G67880 | protein coding |
| A_84_P20965 | 0.009 | 0.489 | AT1G17970 | protein coding |
| A_84_P11128 | 0.0182 | 0.489 | AT5G13870 | EXGT-A4 (ENDOXYLOGLUCAN TRANSFERASE A4); hydrolase, acting on glycosyl bonds |
| A_84_P821845 | 0.0067 | 0.4888 | AT3G15510 | protein coding |
| A_84_P821393 | 0.0346 | 0.4883 | AT1G17160 | protein coding |
| A_84_P21062 | 0.0031 | 0.4881 | AT2G29400 | TOPP1 (TYPE ONE PROTEIN PHOSPHATASE 1); protein serine/threonine phosphatase |
| A_84_P820387 | 0.0247 | 0.4878 | AT3G12700 | protein coding |
| A_84_P18053 | 0.0021 | 0.4877 | AT1G72830 | protein coding |
| A_84_P501920 | 0.0181 | 0.4873 | AT1G50950 | protein coding |
| A_84_P785842 | 0.0103 | 0.4873 | AT5G66930 | hypothetical protein |
| A_84_P12947 | 0.002 | 0.4871 | AT4G37730 | ATBZIP7 (ARABIDOPSIS THALIANA BASIC LEUCINE-ZIPPER 7); DNA binding / transcription factor |
| A_84_P845392 | 0.0215 | 0.4868 | AT1G75340 | protein coding |
| A_84_P23141 | 0.0249 | 0.4863 | AT3G45040 | protein coding |
| A_84_P858372 | 0.0128 | 0.4862 | AT3G48680 | protein coding |
| A_84_P521588 | 0.0447 | 0.4853 | AT2G19200 | hypothetical protein |
| A_84_P853798 | 0.0395 | 0.4849 | AT4G35750 | Rho-GTPase-activating protein-related |
| A_84_P88439 | 0.0086 | 0.4848 | AT1G45976 | protein coding |
| A_84_P829098 | 0.0115 | 0.4845 | AT3G13810 | protein coding |
| A_84_P816989 | 0.0128 | 0.4843 | AT4G32060 | calcium-binding EF hand family protein |
| A_84_P169543 | 0.0167 | 0.4842 | AT1G32540 | protein coding |
| A_84_P810864 | 0.0487 | 0.4842 | AT4G05590 | hypothetical protein |
| A_84_P835598 | 0.0132 | 0.4835 | AT5G22700 | F-box family protein |
| A_84_P11892 | 0.0492 | 0.4835 | AT4G04620 | ATG8B (AUTOPHAGY 8B); microtubule binding |
| A_84_P156095 | 2.00E-04 | 0.4832 | AT4G30690 | translation initiation factor 3 (IF-3) family protein |
| A_84_P17337 | 0.0451 | 0.4826 | AT2G01150 | RHA2B (RING-H2 FINGER PROTEIN 2B); protein binding / zinc ion binding |
| A_84_P819560 | 0.0287 | 0.4826 | AT4G00780 | meprin and TRAF homology domain-containing protein / MATH domain-containing protein |
| A_84_P820690 | 0.0246 | 0.4825 | AT3G16740 | protein coding |
| A_84_P770257 | 0.0467 | 0.4823 | AT2G07674 | hypothetical protein |
| A_84_P106832 | 0.0328 | 0.4816 | AT5G13500 | hypothetical protein |
| A_84_P10665 | 0.0125 | 0.4816 | AT2G31230 | ATERF15 (ETHYLENE-RESPONSIVE ELEMENT BINDING FACTOR 15); DNA binding / transcription activator/ tran |
| A_84_P18189 | 0.0346 | 0.4815 | AT2G33480 | ANAC041 (Arabidopsis NAC domain containing protein 41); transcription factor |
| A_84_P13835 | 0.0078 | 0.4812 | AT4G24060 | Dof-type zinc finger domain-containing protein |
| A_84_P10800 | 0.0235 | 0.4809 | AT3G18370 | protein coding |
| A_84_P218298 | 0.0374 | 0.4809 | AT2G42760 | hypothetical protein |
| A_84_P10305 | 0.0372 | 0.4798 | AT5G59730 | ATEXO70H7 (EXOCYST SUBUNIT EXO70 FAMILY PROTEIN H7); protein binding |
| A_84_P19632 | 0.0128 | 0.4794 | AT5G11740 | AGP15 (ARABINOGALACTAN PROTEIN 15) |
| A_84_P816866 | 0.0054 | 0.4787 | AT5G59950 | RNA and export factor-binding protein, putative |
| A_84_P753384 | 0.0071 | 0.4787 | AT1G24825 | miscRNA |
| A_84_P583737 | 0.0439 | 0.4787 | AT2G20500 | hypothetical protein |
| A_84_P860600 | 0.037 | 0.478 | AT1G13360 | protein coding |
| A_84_P15545 | 0.0434 | 0.4779 | AT3G13380 | protein coding |
| A_84_P24003 | 0.0062 | 0.4777 | AT3G07690 | protein coding |
| A_84_P16304 | 0.0066 | 0.4777 | AT2G16720 | MYB7 (myb domain protein 7); DNA binding / transcription factor |
| A_84_P20488 | 0.0496 | 0.4775 | AT4G31060 | AP2 domain-containing transcription factor, putative |
| A_84_P569157 | 0.0362 | 0.4772 | AT5G67410 | hypothetical protein |
| A_84_P20203 | 0.0307 | 0.477 | AT3G01770 | protein coding |
| A_84_P11193 | 0.0381 | 0.477 | AT5G44210 | ATERF-9/ATERF9/ERF9 (ERF domain protein 9); DNA binding / transcription factor/ transcription repres |
| A_84_P605975 | 0.0136 | 0.4765 | AT2G04800 | hypothetical protein |
| A_84_P812952 | 0.0249 | 0.4758 | AT5G45350 | proline-rich family protein |
| A_84_P13923 | 0.0236 | 0.4758 | AT5G01820 | ATSR1 (SERINE/THREONINE PROTEIN KINASE 1); kinase |
| A_84_P817305 | 0.015 | 0.4757 | AT3G03920 | protein coding |
| A_84_P12660 | 0.0411 | 0.4756 | AT3G06860 | protein coding |
| A_84_P287270 | 0.0077 | 0.4756 | AT5G03160 | DNAJ heat shock N-terminal domain-containing protein |
| A_84_P10084 | 0.0055 | 0.4755 | AT4G30440 | GAE1 (UDP-D-GLUCURONATE 4-EPIMERASE 1); UDP-glucuronate 4-epimerase/ catalytic |
| A_84_P23302 | 0.0138 | 0.4753 | AT1G17290 | protein coding |
| A_84_P750661 | 0.0346 | 0.4752 | AT1G07170 | protein coding |
| A_84_P14082 | 0.0199 | 0.4747 | AT5G58980 | ceramidase family protein |
| A_84_P72214 | 0.0478 | 0.4745 | AT2G04690 | cellular repressor of E1A-stimulated genes (CREG) family |
| A_84_P10296 | 0.0011 | 0.4744 | AT5G57660 | zinc finger (B-box type) family protein |
| A_84_P190014 | 0.0203 | 0.4744 | AT4G36030 | armadillo/beta-catenin repeat family protein |
| A_84_P148728 | 0.0496 | 0.4743 | AT1G08980 | protein coding |
| A_84_P823247 | 0.0071 | 0.4739 | AT3G12100 | protein coding |
| A_84_P271800 | 0.0384 | 0.4737 | AT4G23170 | EP1; protein kinase |
| A_84_P18307 | 0.0074 | 0.4736 | AT3G11650 | protein coding |
| A_84_P13166 | 0.0031 | 0.4732 | AT5G65670 | IAA9 (indoleacetic acid-induced protein 9); transcription factor |
| A_84_P857361 | 0.0171 | 0.4726 | AT4G28400 | protein phosphatase 2C, putative / PP2C, putative |
| A_84_P247085 | 0.0388 | 0.4726 | AT3G26580 | protein coding |
| A_84_P591730 | 0.0198 | 0.4725 | AT2G06255 | ELF4-L3 (ELF4-LIKE 3) |
| A_84_P18016 | 0.0101 | 0.4724 | AT1G70360 | protein coding |
| A_84_P525395 | 0.0165 | 0.4721 | AT4G02075 | PIT1 (PITCHOUN 1); protein binding / zinc ion binding |
| A_84_P16408 | 0.0207 | 0.472 | AT3G11440 | protein coding |
| A_84_P94069 | 0.0103 | 0.4716 | AT5G53160 | hypothetical protein |
| A_84_P19751 | 0.0148 | 0.4715 | AT5G57580 | calmodulin-binding protein |
| A_84_P13734 | 0.0042 | 0.4711 | AT3G56190 | protein coding |
| A_84_P12090 | 0.0169 | 0.4707 | AT5G23190 | CYP86B1 (cytochrome P450, family 86, subfamily B, polypeptide 1); oxygen binding |
| A_84_P21849 | 0.0077 | 0.4706 | AT1G18720 | protein coding |
| A_84_P12837 | 0.0246 | 0.47 | AT4G04630 | hypothetical protein |
| A_84_P793408 | 0.0355 | 0.4699 | AT1G74670 | protein coding |
| A_84_P816463 | 0.0278 | 0.4699 | AT5G02290 | NAK; kinase |
| A_84_P229609 | 0.0133 | 0.4695 | AT4G01950 | ATGPAT3/GPAT3 (GLYCEROL-3-PHOSPHATE ACYLTRANSFERASE 3); acyltransferase |
| A_84_P21324 | 0.0341 | 0.469 | AT3G62422 | protein coding |
| A_84_P756124 | 0.0424 | 0.469 | AT2G05810 | armadillo/beta-catenin repeat family protein |
| A_84_P16553 | 0.0121 | 0.4685 | AT3G52790 | protein coding |
| A_84_P612049 | 0.0083 | 0.4684 | AT1G29400 | protein coding |
| A_84_P805936 | 0.0229 | 0.4682 | AT1G12810 | protein coding |
| A_84_P817886 | 0.0156 | 0.4682 | AT2G18730 | diacylglycerol kinase, putative |
| A_84_P198764 | 0.0085 | 0.4681 | AT3G49790 | protein coding |
| A_84_P832377 | 4.00E-04 | 0.4675 | AT1G01950 | protein coding |
| A_84_P19365 | 0.0344 | 0.4674 | AT3G46600 | protein coding |
| A_84_P853537 | 0.0203 | 0.4668 | AT1G67060 | protein coding |
| A_84_P589734 | 0.033 | 0.4663 | AT1G13610 | protein coding |
| A_84_P553302 | 0.0355 | 0.4662 | AT1G25500 | protein coding |
| A_84_P869171 | 0.0052 | 0.466 | AT5G36290 | hypothetical protein |
| A_84_P15959 | 0.0258 | 0.4659 | AT5G55170 | SUM3 (SMALL UBIQUITIN-LIKE MODIFIER 3) |
| A_84_P856279 | 0.0261 | 0.4659 | AT5G53350 | CLPX (Clp protease regulatory subunit X); ATPase |
| A_84_P844220 | 0.0032 | 0.4647 | AT4G12750 | sequence-specific DNA binding / transcription factor |
| A_84_P87419 | 0.038 | 0.4644 | AT3G25290 | protein coding |
| A_84_P22935 | 0.0293 | 0.464 | AT2G22680 | zinc finger (C3HC4-type RING finger) family protein |
| A_84_P22009 | 0.0401 | 0.4638 | AT2G45660 | AGL20 (AGAMOUS-LIKE 20); transcription factor |
| A_84_P23665 | 0.0141 | 0.4637 | AT1G03610 | protein coding |
| A_84_P848008 | 0.0018 | 0.4635 | AT2G27920 | SCPL51; serine carboxypeptidase |
| A_84_P23188 | 0.0086 | 0.4635 | AT3G55450 | protein coding |
| A_84_P758371 | 0.0328 | 0.4635 | AT2G36854 | hypothetical protein |
| A_84_P20840 | 0.0129 | 0.4633 | AT1G08900 | protein coding |
| A_84_P790686 | 0.004 | 0.4632 | AT3G23460 | protein coding |
| A_84_P857824 | 0.0178 | 0.4632 | AT3G28910 | protein coding |
| A_84_P20935 | 0.0372 | 0.4626 | AT1G21830 | protein coding |
| A_84_P595110 | 0.0021 | 0.4626 | AT1G20696 | protein coding |
| A_84_P10829 | 0.0112 | 0.4624 | AT3G13730 | protein coding |
| A_84_P590959 | 0.0058 | 0.4624 | AT3G03180 | protein coding |
| A_84_P15881 | 0.0205 | 0.4624 | AT5G26040 | HDA2 (histone deacetylase 2); histone deacetylase |
| A_84_P20783 | 0.0354 | 0.4624 | AT2G14080 | disease resistance protein (TIR-NBS-LRR class), putative |
| A_84_P11049 | 0.0077 | 0.4617 | AT4G34970 | actin binding |
| A_84_P11796 | 0.0165 | 0.4615 | AT3G45590 | protein coding |
| A_84_P554154 | 0.0203 | 0.4614 | AT1G13245 | protein coding |
| A_84_P298504 | 0.0396 | 0.4614 | AT2G24140 | hypothetical protein |
| A_84_P81259 | 0.003 | 0.4611 | AT4G21510 | F-box family protein |
| A_84_P20185 | 0.031 | 0.4607 | AT2G25490 | EBF1 (EIN3-BINDING F BOX PROTEIN 1); ubiquitin-protein ligase |
| A_84_P15649 | 0.0191 | 0.4604 | AT3G62650 | protein coding |
| A_84_P713168 | 3.00E-04 | 0.4604 | AT1G15405 | misc_RNA |
| A_84_P824660 | 0.0109 | 0.4602 | AT4G10925 | F-box family protein |
| A_84_P151138 | 0.0392 | 0.4599 | AT3G63210 | protein coding |
| A_84_P826907 | 0.0094 | 0.4598 | AT3G50950 | protein coding |
| A_84_P284310 | 0.0383 | 0.4598 | AT2G01520 | MLP328 (MLP-LIKE PROTEIN 328) |
| A_84_P19868 | 0.0121 | 0.4592 | AT1G03960 | protein coding |
| A_84_P809720 | 7.00E-04 | 0.4585 | AT3G15353 | protein coding |
| A_84_P787971 | 0.0109 | 0.4584 | AT2G43470 | hypothetical protein |
| A_84_P832988 | 0.0229 | 0.4578 | AT3G08800 | protein coding |
| A_84_P23861 | 0.0158 | 0.4578 | AT2G46610 | arginine/serine-rich splicing factor, putative |
| A_84_P19898 | 0.0061 | 0.4576 | AT1G80960 | protein coding |
| A_84_P766867 | 0.0106 | 0.4569 | AT5G63470 | CCAAT-box binding transcription factor Hap5a, putative |
| A_84_P17106 | 0.0159 | 0.4568 | AT1G34670 | protein coding |
| A_84_P825944 | 0.0471 | 0.4566 | AT4G16860 | RPP4 (RECOGNITION OF PERONOSPORA PARASITICA 4) |
| A_84_P17864 | 0.0034 | 0.4563 | AT5G58860 | CYP86A1 (cytochrome P450, family 86, subfamily A, polypeptide 1); oxygen binding |
| A_84_P805180 | 0.0125 | 0.456 | AT5G66240 | transducin family protein / WD-40 repeat family protein |
| A_84_P15206 | 0.0062 | 0.456 | AT1G16250 | protein coding |
| A_84_P837228 | 0.0129 | 0.4558 | AT5G04980 | endonuclease/exonuclease/phosphatase family protein |
| A_84_P513735 | 0.0182 | 0.4557 | AT3G13857 | protein coding |
| A_84_P235233 | 0.0282 | 0.4556 | AT4G36610 | hydrolase, alpha/beta fold family protein |
| A_84_P60750 | 0.0263 | 0.4552 | AT3G19895 | protein coding |
| A_84_P821361 | 0.0053 | 0.4552 | AT4G02600 | ATMLO1/MLO1 (MILDEW RESISTANCE LOCUS O 1); calmodulin binding |
| A_84_P500277 | 0.0467 | 0.4549 | AT2G17705 | peptide-methionine-(S)-S-oxide reductase |
| A_84_P267210 | 0.0032 | 0.4537 | AT5G03455 | CDC25 |
| A_84_P212118 | 0.0363 | 0.4536 | AT4G04960 | lectin protein kinase, putative |
| A_84_P85609 | 0.0024 | 0.4535 | AT3G09030 | protein coding |
| A_84_P12439 | 0.0187 | 0.4534 | AT1G68110 | protein coding |
| A_84_P788394 | 0.0196 | 0.4533 | AT1G37150 | protein coding |
| A_84_P181204 | 0.0084 | 0.4532 | AT4G18380 | F-box family protein |
| A_84_P118832 | 0.0069 | 0.4531 | AT2G19350 | hypothetical protein |
| A_84_P552183 | 0.0215 | 0.453 | AT5G27020 | hypothetical protein |
| A_84_P11324 | 0.0018 | 0.4529 | AT1G20540 | protein coding |
| A_84_P72294 | 0.03 | 0.4527 | AT5G11610 | exostosin family protein |
| A_84_P22255 | 0.049 | 0.4523 | AT3G58710 | protein coding |
| A_84_P547686 | 0.0074 | 0.4518 | AT4G36550 | binding / ubiquitin-protein ligase |
| A_84_P23786 | 0.0312 | 0.4517 | AT1G68820 | protein coding |
| A_84_P592825 | 0.0024 | 0.4512 | AT3G23480 | protein coding |
| A_84_P23320 | 0.0329 | 0.4511 | AT4G29820 | ATCFIM-25/CFIM-25 (ARABIDOPSIS HOMOLOG OF CFIM-25) |
| A_84_P827605 | 0.03 | 0.4505 | AT4G34100 | protein binding / zinc ion binding |
| A_84_P67134 | 0.0228 | 0.4504 | AT5G64552 | CPuORF22 (Conserved peptide upstream open reading frame 22) |
| A_84_P849266 | 0.0058 | 0.4501 | AT5G20120 | hypothetical protein |
| A_84_P16285 | 0.0434 | 0.4501 | AT2G29720 | CTF2B; monooxygenase |
| A_84_P824901 | 9.00E-04 | 0.4495 | AT3G11100 | protein coding |
| A_84_P19570 | 0.0406 | 0.4493 | AT4G38550 | hypothetical protein |
| A_84_P567531 | 0.0091 | 0.4485 | AT1G49170 | protein coding |
| A_84_P715317 | 0.0096 | 0.4484 | AT1G72416 | protein coding |
| A_84_P13850 | 0.0135 | 0.4472 | AT4G27260 | GH3.5/WES1; indole-3-acetic acid amido synthetase |
| A_84_P111022 | 0.0219 | 0.4468 | AT1G33970 | protein coding |
| A_84_P823935 | 0.0395 | 0.4463 | AT4G10000 | hypothetical protein |
| A_84_P175691 | 0.0214 | 0.4454 | AT3G60040 | protein coding |
| A_84_P16088 | 0.0171 | 0.4451 | AT1G24440 | protein coding |
| A_84_P15229 | 0.001 | 0.4451 | AT1G79650 | protein coding |
| A_84_P850685 | 0.0042 | 0.445 | AT2G18440 | misc_RNA |
| A_84_P804017 | 0.0025 | 0.4447 | AT5G10336 | hypothetical protein |
| A_84_P10975 | 0.0148 | 0.4447 | AT4G13850 | ATGRP2 (GLYCINE-RICH RNA-BINDING PROTEIN 2) |
| A_84_P11819 | 0.0141 | 0.4443 | AT3G50740 | protein coding |
| A_84_P283660 | 2.00E-04 | 0.444 | AT2G42040 | hypothetical protein |
| A_84_P12407 | 0.0348 | 0.4439 | AT1G73700 | protein coding |
| A_84_P760354 | 0.0262 | 0.4438 | AT3G56705 | ncRNA |
| A_84_P11451 | 0.0448 | 0.4436 | AT1G78280 | protein coding |
| A_84_P759269 | 0.0418 | 0.4435 | AT3G43790 | protein coding |
| A_84_P18284 | 0.0314 | 0.4434 | AT2G44750 | TPK2 (THIAMIN PYROPHOSPHOKINASE 2); thiamin diphosphokinase |
| A_84_P14824 | 0.035 | 0.4433 | AT4G34020 | DJ-1 family protein |
| A_84_P821604 | 0.0194 | 0.443 | AT5G44290 | protein kinase family protein |
| A_84_P13866 | 0.016 | 0.443 | AT4G30660 | hydrophobic protein, putative / low temperature and salt responsive protein, putative |
| A_84_P13952 | 0.0323 | 0.443 | AT5G10570 | basic helix-loop-helix (bHLH) family protein |
| A_84_P19875 | 0.0129 | 0.4429 | AT1G60610 | protein coding |
| A_84_P15965 | 0.0216 | 0.4427 | AT5G56980 | hypothetical protein |
| A_84_P11822 | 0.0444 | 0.4422 | AT3G51450 | protein coding |
| A_84_P790000 | 0.0249 | 0.442 | AT2G27490 | ATCOAE; ATP binding / dephospho-CoA kinase |
| A_84_P125571 | 0.0266 | 0.4418 | AT5G48560 | basic helix-loop-helix (bHLH) family protein |
| A_84_P515753 | 0.0037 | 0.4416 | AT4G31510 | hypothetical protein |
| A_84_P18227 | 0.0181 | 0.4413 | AT2G02760 | ATUBC2 (UBIQUITING-CONJUGATING ENZYME 2); ubiquitin-protein ligase |
| A_84_P22698 | 6.00E-04 | 0.4406 | AT1G64460 | protein coding |
| A_84_P21943 | 0.0481 | 0.4405 | AT1G32170 | protein coding |
| A_84_P140429 | 1.00E-04 | 0.4403 | AT2G41780 | hypothetical protein |
| A_84_P704941 | 0.0224 | 0.4396 | AT1G18075 | misc_RNA |
| A_84_P18676 | 2.00E-04 | 0.4392 | AT5G08520 | myb family transcription factor |
| A_84_P22087 | 0.0011 | 0.4383 | AT3G10190 | protein coding |
| A_84_P173301 | 0.0061 | 0.4381 | AT5G49350 | hypothetical protein |
| A_84_P199074 | 9.00E-04 | 0.4378 | AT3G48330 | protein coding |
| A_84_P309133 | 0.01 | 0.4377 | AT5G18065 | hypothetical protein |
| A_84_P516320 | 0.0025 | 0.4377 | AT2G34655 | hypothetical protein |
| A_84_P10312 | 0.0112 | 0.4375 | AT5G61250 | ATGUS1 (ARABIDOPSIS THALIANA GLUCURONIDASE 1); beta-glucuronidase |
| A_84_P17903 | 0.003 | 0.4374 | AT5G14960 | DEL2/E2FD/E2L1 (DP-E2F-LIKE 2); DNA binding / transcription factor |
| A_84_P256230 | 0.0099 | 0.4373 | AT5G18150 | hypothetical protein |
| A_84_P267870 | 0.0184 | 0.4361 | AT5G14930 | SAG101 (SENESCENCE-ASSOCIATED GENE 101) |
| A_84_P861017 | 0.0074 | 0.436 | AT2G36670 | aspartyl protease family protein |
| A_84_P131556 | 0.0335 | 0.4358 | AT2G42570 | hypothetical protein |
| A_84_P15839 | 0.0197 | 0.4357 | AT5G09540 | DNAJ heat shock N-terminal domain-containing protein |
| A_84_P16679 | 0.0036 | 0.4355 | AT4G25620 | hydroxyproline-rich glycoprotein family protein |
| A_84_P582789 | 0.0294 | 0.4354 | AT1G80450 | protein coding |
| A_84_P11032 | 0.002 | 0.4353 | AT4G30940 | potassium channel tetramerisation domain-containing protein |
| A_84_P14907 | 0.0282 | 0.4353 | AT5G13700 | APAO/ATPAO1 (POLYAMINE OXIDASE 1); FAD binding / polyamine oxidase |
| A_84_P274610 | 0.0488 | 0.435 | AT2G39000 | GCN5-related N-acetyltransferase (GNAT) family protein |
| A_84_P13569 | 0.0121 | 0.4349 | AT2G23770 | protein kinase family protein / peptidoglycan-binding LysM domain-containing protein |
| A_84_P801401 | 0.0236 | 0.4347 | AT5G52510 | scarecrow-like transcription factor 8 (SCL8) |
| A_84_P14348 | 0.0476 | 0.4345 | AT1G13700 | protein coding |
| A_84_P18701 | 0.0046 | 0.4343 | AT5G18310 | hypothetical protein |
| A_84_P97886 | 0.0296 | 0.4343 | AT2G40130 | heat shock protein-related |
| A_84_P23730 | 0.0282 | 0.4342 | AT1G53910 | protein coding |
| A_84_P533074 | 0.0132 | 0.4336 | AT1G78895 | protein coding |
| A_84_P278280 | 0.0065 | 0.4336 | AT2G39570 | ACT domain-containing protein |
| A_84_P588546 | 0.0037 | 0.4334 | AT4G34480 | glycosyl hydrolase family 17 protein |
| A_84_P18716 | 0.0373 | 0.4331 | AT5G25350 | EBF2 (EIN3-BINDING F BOX PROTEIN 2) |
| A_84_P11544 | 8.00E-04 | 0.4328 | AT1G33590 | protein coding |
| A_84_P177344 | 0.0281 | 0.4327 | AT3G26180 | protein coding |
| A_84_P513144 | 0.022 | 0.4327 | AT5G14550 | hypothetical protein |
| A_84_P751163 | 0.0213 | 0.4324 | AT1G24880 | protein coding |
| A_84_P15716 | 0.0149 | 0.4321 | AT4G21560 | vacuolar protein sorting-associated protein 28 family protein / VPS28 family protein |
| A_84_P828765 | 0.0302 | 0.4311 | AT2G43930 | protein kinase family protein |
| A_84_P81529 | 0.0263 | 0.4309 | AT2G43060 | transcription factor |
| A_84_P16670 | 0.0039 | 0.4308 | AT4G23660 | ATPPT1 (ARABIDOPSIS THALIANA POLYPRENYLTRANSFERASE 1); 4-hydroxybenzoate nonaprenyltransferase |
| A_84_P297384 | 0.0459 | 0.4295 | AT4G31730 | GDU1 (GLUTAMINE DUMPER 1) |
| A_84_P24076 | 0.0245 | 0.429 | AT3G12070 | protein coding |
| A_84_P22694 | 0.0037 | 0.4288 | AT1G55680 | protein coding |
| A_84_P22303 | 0.0179 | 0.4285 | AT4G09030 | AGP10 (Arabinogalactan protein 10) |
| A_84_P542432 | 0.0198 | 0.4284 | AT5G20810 | auxin-responsive protein, putative / small auxin up RNA (SAUR_B) |
| A_84_P258110 | 0.0145 | 0.4281 | AT1G65800 | protein coding |
| A_84_P829500 | 0.0478 | 0.4279 | AT1G73300 | protein coding |
| A_84_P11493 | 0.0165 | 0.4278 | AT1G68050 | protein coding |
| A_84_P86109 | 0.0385 | 0.4278 | AT1G80210 | protein coding |
| A_84_P261730 | 0.0047 | 0.4276 | AT3G05220 | protein coding |
| A_84_P823211 | 0.0113 | 0.4273 | AT5G42420 | transporter-related |
| A_84_P283770 | 0.0023 | 0.4271 | AT3G27540 | protein coding |
| A_84_P15354 | 0.0261 | 0.4271 | AT2G20780 | mannitol transporter, putative |
| A_84_P786702 | 0.0429 | 0.4266 | AT3G09830 | protein coding |
| A_84_P18042 | 0.0375 | 0.4264 | AT1G10690 | protein coding |
| A_84_P23079 | 0.0474 | 0.4262 | AT3G18080 | protein coding |
| A_84_P800461 | 0.0382 | 0.4262 | AT1G78130 | protein coding |
| A_84_P824673 | 0.0241 | 0.4262 | AT1G08890 | protein coding |
| A_84_P16736 | 0.022 | 0.4258 | AT4G39270 | leucine-rich repeat transmembrane protein kinase, putative |
| A_84_P766012 | 0.009 | 0.4252 | AT5G39190 | GLP2A (GERMIN-LIKE PROTEIN 2A); manganese ion binding / metal ion binding / nutrient reservoir |
| A_84_P13433 | 0.0235 | 0.425 | AT1G32270 | protein coding |
| A_84_P851019 | 0.0138 | 0.4249 | AT1G73950 | protein coding |
| A_84_P19557 | 0.0089 | 0.4245 | AT4G34750 | auxin-responsive protein, putative / small auxin up RNA (SAUR_E) |
| A_84_P53790 | 0.0247 | 0.4242 | AT1G49500 | protein coding |
| A_84_P22506 | 0.014 | 0.4241 | AT5G27320 | ATGID1C/GID1C (GA INSENSITIVE DWARF1C); hydrolase |
| A_84_P13549 | 0.0088 | 0.4237 | AT2G39420 | esterase/lipase/thioesterase family protein |
| A_84_P15958 | 0.0143 | 0.4231 | AT5G54940 | eukaryotic translation initiation factor SUI1, putative |
| A_84_P828790 | 0.0158 | 0.423 | AT3G13430 | protein coding |
| A_84_P21382 | 0.0472 | 0.4227 | AT4G19200 | proline-rich family protein |
| A_84_P507987 | 0.0438 | 0.4225 | AT1G03200 | protein coding |
| A_84_P833933 | 0.0474 | 0.4224 | AT5G16910 | ATCSLD2 (Cellulose synthase-like D2); cellulose synthase/ transferase, transferring glycosyl groups |
| A_84_P157225 | 0.0023 | 0.4222 | AT2G41760 | hypothetical protein |
| A_84_P237233 | 0.0183 | 0.4221 | AT5G05190 | hypothetical protein |
| A_84_P21720 | 0.0467 | 0.422 | AT1G35320 | protein coding |
| A_84_P14435 | 0.0081 | 0.4219 | AT2G26710 | BAS1/CYP734A1 (PHYB ACTIVATION TAGGED SUPPRESSOR 1); oxygen binding / steroid hydroxylase |
| A_84_P19791 | 0.0065 | 0.4214 | AT5G66910 | disease resistance protein (CC-NBS-LRR class), putative |
| A_84_P159915 | 0.0302 | 0.4213 | AT4G27970 | SLAH2 (SLAC1 HOMOLOGUE 2); transporter |
| A_84_P299330 | 0.0024 | 0.4211 | AT1G13930 | protein coding |
| A_84_P792853 | 0.0236 | 0.4209 | AT1G53790 | protein coding |
| A_84_P12423 | 0.0435 | 0.4201 | AT1G26670 | protein coding |
| A_84_P227579 | 0.0284 | 0.4201 | AT4G15540 | hypothetical protein |
| A_84_P240305 | 0.0191 | 0.42 | AT2G46550 | hypothetical protein |
| A_84_P250505 | 0.0349 | 0.4199 | AT5G46780 | VQ motif-containing protein |
| A_84_P19788 | 0.0246 | 0.4195 | AT5G66210 | CPK28 (calcium-dependent protein kinase 28) |
| A_84_P833779 | 0.0292 | 0.4187 | AT1G69450 | protein coding |
| A_84_P13311 | 0.0118 | 0.4186 | AT1G20840 | protein coding |
| A_84_P13699 | 0.0281 | 0.4179 | AT3G48550 | protein coding |
| A_84_P817969 | 0.0393 | 0.4171 | AT1G75390 | - |
| A_84_P16098 | 0.0086 | 0.417 | AT1G03730 | protein coding |
| A_84_P13528 | 0.0123 | 0.417 | AT2G36900 | MEMB11 (Golgi SNARE protein membrin 11); SNAP receptor |
| A_84_P11956 | 0.0032 | 0.4165 | AT1G51550 | protein coding |
| A_84_P134195 | 0.0038 | 0.4156 | AT2G37478 | CPuORF52 (Conserved peptide upstream open reading frame 52) |
| A_84_P23843 | 0.0417 | 0.4156 | AT2G03200 | aspartyl protease family protein |
| A_84_P562146 | 0.014 | 0.4149 | AT1G19397 | protein coding |
| A_84_P18117 | 0.0056 | 0.4149 | AT1G75800 | protein coding |
| A_84_P17641 | 0.0132 | 0.4146 | AT4G29050 | lectin protein kinase family protein |
| A_84_P14652 | 0.0335 | 0.4143 | AT3G50310 | protein coding |
| A_84_P16871 | 0.0271 | 0.4143 | AT5G45800 | MEE62 (maternal effect embryo arrest 62); ATP binding / protein serine/threonine kinase |
| A_84_P56830 | 0.0026 | 0.4142 | AT4G19700 | protein binding / zinc ion binding |
| A_84_P89509 | 0.016 | 0.4141 | AT5G18490 | hypothetical protein |
| A_84_P18736 | 0.024 | 0.4139 | AT5G38280 | PR5K (PR5-like receptor kinase); kinase/ transmembrane receptor protein serine/threonine kinase |
| A_84_P305230 | 0.0224 | 0.4138 | AT5G18780 | F-box family protein |
| A_84_P568372 | 0.0091 | 0.4137 | AT1G22985 | protein coding |
| A_84_P757964 | 0.003 | 0.4133 | AT2G41945 | hypothetical protein |
| A_84_P16781 | 0.0133 | 0.4132 | AT1G06460 | protein coding |
| A_84_P14584 | 0.0212 | 0.4127 | AT3G26280 | protein coding |
| A_84_P761688 | 0.0064 | 0.4114 | AT3G52535 | miscRNA |
| A_84_P16782 | 0.014 | 0.4111 | AT5G07880 | SNAP29 (Synaptosomal-associated protein SNAP25-like 29) |
| A_84_P12173 | 0.012 | 0.4109 | AT5G53730 | harpin-induced family protein / HIN1 family protein / harpin-responsive family protein |
| A_84_P555382 | 0.0486 | 0.4106 | AT3G09950 | protein coding |
| A_84_P10083 | 0.0306 | 0.4106 | AT4G30290 | ATXTH19 (XYLOGLUCAN ENDOTRANSGLUCOSYLASE/HYDROLASE 19); hydrolase, acting on glycosyl bonds |
| A_84_P194014 | 0.0268 | 0.4104 | AT5G51370 | F-box family protein |
| A_84_P192974 | 0.0048 | 0.4099 | AT5G65660 | hydroxyproline-rich glycoprotein family protein |
| A_84_P608472 | 0.032 | 0.4098 | AT1G28760 | protein coding |
| A_84_P576925 | 0.0032 | 0.4098 | AT4G22390 | hypothetical protein |
| A_84_P251975 | 0.0364 | 0.4096 | AT5G56520 | hypothetical protein |
| A_84_P23020 | 0.0061 | 0.4096 | AT2G22850 | ATBZIP6 (ARABIDOPSIS THALIANA BASIC LEUCINE-ZIPPER 6); DNA binding / transcription factor |
| A_84_P556854 | 0.0244 | 0.4092 | AT1G25275 | protein coding |
| A_84_P753163 | 0.032 | 0.4089 | AT1G58245 | protein coding |
| A_84_P16207 | 0.0206 | 0.4084 | AT1G68360 | protein coding |
| A_84_P830586 | 0.007 | 0.4078 | AT5G22310 | hypothetical protein |
| A_84_P570105 | 0.001 | 0.4076 | AT1G06810 | protein coding |
| A_84_P758943 | 0.0252 | 0.4073 | AT3G62750 | protein coding |
| A_84_P15159 | 6.00E-04 | 0.407 | AT1G70090 | protein coding |
| A_84_P22964 | 0.0254 | 0.4068 | AT2G02360 | ATPP2-B10 (Phloem protein 2-B10); carbohydrate binding |
| A_84_P10490 | 0.0045 | 0.4067 | AT1G27630 | protein coding |
| A_84_P14919 | 0.0126 | 0.4066 | AT5G18640 | lipase class 3 family protein |
| A_84_P123572 | 0.0061 | 0.4059 | AT5G01700 | protein phosphatase 2C, putative / PP2C, putative |
| A_84_P19527 | 0.0161 | 0.4058 | AT4G27745 | hypothetical protein |
| A_84_P13328 | 0.0053 | 0.4055 | AT1G78080 | protein coding |
| A_84_P548594 | 0.0134 | 0.405 | AT5G05840 | hypothetical protein |
| A_84_P182054 | 0.0169 | 0.4046 | AT5G37690 | GDSL-motif lipase/hydrolase family protein |
| A_84_P171853 | 0.0189 | 0.4044 | AT1G13390 | protein coding |
| A_84_P24020 | 0.0142 | 0.4039 | AT1G26440 | protein coding |
| A_84_P825490 | 0.0033 | 0.4038 | AT2G02370 | hypothetical protein |
| A_84_P755055 | 0.0277 | 0.4036 | AT2G29050 | ATRBL1 (ARABIDOPSIS THALIANA RHOMBOID-LIKE 1) |
| A_84_P820217 | 0.0171 | 0.4036 | AT2G21240 | ATBPC4/BBR/BPC4/BPC4 (BASIC PENTACYSTEINE 4) |
| A_84_P17200 | 0.0224 | 0.4035 | AT1G16110 | protein coding |
| A_84_P24052 | 0.0133 | 0.4034 | AT3G27280 | protein coding |
| A_84_P13593 | 0.0198 | 0.4034 | AT3G04670 | protein coding |
| A_84_P156715 | 0.0272 | 0.4034 | AT5G62770 | hypothetical protein |
| A_84_P11771 | 0.0147 | 0.4031 | AT3G22930 | protein coding |
| A_84_P275770 | 0.0274 | 0.4029 | AT1G12730 | protein coding |
| A_84_P17961 | 0.0242 | 0.4028 | AT1G02350 | protein coding |
| A_84_P17292 | 0.0387 | 0.4022 | AT2G22430 | ATHB6 (ARABIDOPSIS THALIANA HOMEOBOX PROTEIN 6); transcription factor |
| A_84_P17571 | 0.0164 | 0.4018 | AT4G08470 | MAPKKK10 (Mitogen-activated protein kinase kinase kinase 10); kinase |
| A_84_P11424 | 0.018 | 0.4011 | AT1G32700 | protein coding |
| A_84_P102486 | 0.0295 | 0.4011 | AT2G46490 | hypothetical protein |
| A_84_P200494 | 0.0149 | 0.4009 | AT5G40960 | hypothetical protein |
| A_84_P12220 | 0.0477 | 0.4008 | AT1G52330 | protein coding |
| A_84_P67194 | 0.0275 | 0.4008 | AT4G18596 | pollen Ole e 1 allergen and extensin family protein |
| A_84_P10447 | 0.0017 | 0.4003 | AT1G77210 | protein coding |
| A_84_P22063 | 0.0273 | 0.4001 | AT2G48010 | RKF3 (RECEPTOR-LIKE KINASE IN IN FLOWERS 3); kinase |
| A_84_P15345 | 0.0461 | 0.4001 | AT2G30210 | LAC3 (laccase 3); copper ion binding / oxidoreductase |
| A_84_P189784 | 0.0111 | 0.3999 | AT3G18690 | protein coding |
| A_84_P21164 | 0.0206 | 0.3999 | AT3G08760 | protein coding |
| A_84_P268330 | 0.0164 | 0.3999 | AT1G80570 | protein coding |
| A_84_P69044 | 0.0021 | 0.3994 | AT1G05710 | protein coding |
| A_84_P23268 | 0.0158 | 0.3989 | AT4G13830 | J20 (DNAJ-LIKE 20); heat shock protein binding |
| A_84_P765891 | 0.004 | 0.3985 | AT4G12382 | hypothetical protein |
| A_84_P259140 | 0.0264 | 0.3984 | AT1G28050 | protein coding |
| A_84_P69104 | 0.0366 | 0.3981 | AT1G77145 | protein coding |
| A_84_P63754 | 0.0296 | 0.3979 | AT5G54585 | hypothetical protein |
| A_84_P809661 | 0.0484 | 0.3978 | AT3G54020 | protein coding |
| A_84_P55630 | 0.0105 | 0.3976 | AT1G02300 | protein coding |
| A_84_P828366 | 0.0049 | 0.3975 | AT1G33610 | protein coding |
| A_84_P267120 | 0.0156 | 0.3963 | AT1G74950 | protein coding |
| A_84_P10632 | 0.0081 | 0.3961 | AT2G16600 | ROC3 (rotamase CyP 3); peptidyl-prolyl cis-trans isomerase |
| A_84_P187904 | 7.00E-04 | 0.3961 | AT1G17620 | protein coding |
| A_84_P786210 | 0.0386 | 0.3958 | AT4G31550 | WRKY11 (WRKY DNA-binding protein 11); transcription factor |
| A_84_P20731 | 0.0177 | 0.3958 | AT5G65530 | protein kinase, putative |
| A_84_P764106 | 0.0318 | 0.395 | AT4G27740 | hypothetical protein |
| A_84_P19045 | 0.0097 | 0.3942 | AT1G68190 | protein coding |
| A_84_P195044 | 0.0146 | 0.3941 | AT1G09575 | protein coding |
| A_84_P307230 | 0.0456 | 0.3941 | AT1G71910 | protein coding |
| A_84_P826922 | 0.0366 | 0.394 | AT2G17280 | phosphoglycerate/bisphosphoglycerate mutase family protein |
| A_84_P602587 | 0.0108 | 0.3936 | AT3G15358 | protein coding |
| A_84_P800757 | 0.0454 | 0.393 | AT5G64260 | phosphate-responsive protein, putative |
| A_84_P22624 | 0.0454 | 0.3928 | AT5G65790 | MYB68 (myb domain protein 68); DNA binding / transcription factor |
| A_84_P766678 | 0.0191 | 0.3923 | AT5G53420 | hypothetical protein |
| A_84_P231449 | 0.0484 | 0.3919 | AT2G46620 | AAA-type ATPase family protein |
| A_84_P222609 | 0.0326 | 0.3907 | AT1G24265 | protein coding |
| A_84_P21236 | 0.028 | 0.3904 | AT3G19553 | protein coding |
| A_84_P825937 | 0.0186 | 0.3903 | AT4G16960 | disease resistance protein (TIR-NBS-LRR class), putative |
| A_84_P22018 | 0.0205 | 0.3901 | AT2G02340 | ATPP2-B8 (Phloem protein 2-B8); carbohydrate binding |
| A_84_P785340 | 0.0148 | 0.3896 | AT1G56150 | protein coding |
| A_84_P787702 | 0.0027 | 0.3894 | AT3G61640 | protein coding |
| A_84_P21357 | 0.0252 | 0.3894 | AT4G09020 | ATISA3/ISA3 (ISOAMYLASE 3); alpha-amylase |
| A_84_P788715 | 0.0151 | 0.3885 | AT1G18470 | protein coding |
| A_84_P580517 | 0.0216 | 0.388 | AT4G33310 | hypothetical protein |
| A_84_P23891 | 0.0468 | 0.3875 | AT2G32830 | PHT5 (phosphate transporter 5); inorganic phosphate transmembrane transporter/ phosphate transmembra |
| A_84_P778821 | 0.0141 | 0.3872 | AT3G62770 | protein coding |
| A_84_P22948 | 0.0079 | 0.3871 | AT2G01450 | ATMPK17 (Arabidopsis thaliana MAP kinase 17); MAP kinase |
| A_84_P514608 | 9.00E-04 | 0.387 | AT3G09032 | protein coding |
| A_84_P83309 | 0.0463 | 0.3862 | AT3G07000 | protein coding |
| A_84_P16006 | 0.0083 | 0.3858 | AT5G66590 | allergen V5/Tpx-1-related family protein |
| A_84_P767689 | 0.006 | 0.3852 | AT5G40395 | ncRNA |
| A_84_P21571 | 0.0068 | 0.3845 | AT5G37720 | RNA and export factor-binding protein, putative |
| A_84_P11866 | 0.006 | 0.3844 | AT3G61850 | protein coding |
| A_84_P788340 | 0.0394 | 0.3824 | AT2G26310 | hypothetical protein |
| A_84_P535181 | 5.00E-04 | 0.3816 | AT4G13195 | CLE44 (CLAVATA3/ESR-RELATED 44) |
| A_84_P18001 | 0.025 | 0.3812 | AT1G65500 | protein coding |
| A_84_P15635 | 0.0112 | 0.3811 | AT3G59080 | protein coding |
| A_84_P715804 | 0.0373 | 0.3802 | AT5G18540 | hypothetical protein |
| A_84_P834809 | 0.0236 | 0.3787 | AT3G12220 | protein coding |
| A_84_P768708 | 2.00E-04 | 0.3784 | AT5G01215 | miscRNA |
| A_84_P17808 | 0.012 | 0.3783 | AT5G43440 | 2-oxoglutarate-dependent dioxygenase, putative |
| A_84_P10686 | 0.0081 | 0.3779 | AT2G17450 | RHA3A (RING-H2 finger A3A); protein binding / zinc ion binding |
| A_84_P550347 | 0.0287 | 0.3778 | AT4G39720 | VQ motif-containing protein |
| A_84_P18936 | 0.0182 | 0.3775 | AT1G03470 | protein coding |
| A_84_P515570 | 0.0302 | 0.3773 | AT3G28850 | protein coding |
| A_84_P846788 | 0.0054 | 0.3772 | AT1G69760 | protein coding |
| A_84_P244595 | 0.0309 | 0.3768 | AT5G67620 | hypothetical protein |
| A_84_P11659 | 0.0457 | 0.3765 | AT2G39400 | hydrolase, alpha/beta fold family protein |
| A_84_P505223 | 0.0242 | 0.3763 | AT5G35732 | hypothetical protein |
| A_84_P764548 | 0.0336 | 0.3762 | AT4G18197 | AT4G18200/ATPUP7/PEX17 (ARABIDOPSIS THALIANA PURINE PERMEASE 7); purine transmembrane transporter |
| A_84_P541229 | 0.0089 | 0.3729 | AT3G13062 | protein coding |
| A_84_P22466 | 0.0487 | 0.3728 | AT5G10860 | CBS domain-containing protein |
| A_84_P13315 | 0.0036 | 0.3727 | AT1G16370 | protein coding |
| A_84_P108412 | 0.0117 | 0.3721 | AT4G22690 | CYP706A1 (cytochrome P450, family 706, subfamily A, polypeptide 1); oxygen binding |
| A_84_P542830 | 0.0361 | 0.3707 | AT2G03310 | hypothetical protein |
| A_84_P196034 | 0.003 | 0.3704 | AT5G05090 | myb family transcription factor |
| A_84_P20289 | 0.025 | 0.3701 | AT3G28270 | protein coding |
| A_84_P846334 | 0.0415 | 0.3695 | AT3G26170 | protein coding |
| A_84_P10669 | 0.0193 | 0.3691 | AT2G31390 | pfkB-type carbohydrate kinase family protein |
| A_84_P12034 | 0.0185 | 0.3681 | AT5G01950 | ATP binding / kinase/ protein serine/threonine kinase |
| A_84_P14199 | 0.0022 | 0.3679 | AT1G67340 | protein coding |
| A_84_P21618 | 0.0215 | 0.3679 | AT5G50160 | ATFRO8/FRO8 (FERRIC REDUCTION OXIDASE 8); ferric-chelate reductase/ oxidoreductase |
| A_84_P16656 | 0.0346 | 0.3675 | AT4G20140 | leucine-rich repeat transmembrane protein kinase, putative |
| A_84_P16688 | 0.0163 | 0.3672 | AT4G27470 | zinc finger (C3HC4-type RING finger) family protein |
| A_84_P14034 | 0.0029 | 0.3671 | AT5G45750 | AtRABA1c (Arabidopsis Rab GTPase homolog A1c); GTP binding |
| A_84_P11244 | 0.0058 | 0.367 | AT5G58430 | ATEXO70B1 (exocyst subunit EXO70 family protein B1); protein binding |
| A_84_P810497 | 0.0082 | 0.3666 | AT1G23490 | protein coding |
| A_84_P19102 | 6.00E-04 | 0.3665 | AT1G51940 | protein coding |
| A_84_P726784 | 0.0119 | 0.3663 | AT4G36648 | misc_RNA |
| A_84_P13560 | 0.0308 | 0.3661 | AT2G32150 | haloacid dehalogenase-like hydrolase family protein |
| A_84_P21097 | 0.0185 | 0.3659 | AT1G67820 | protein coding |
| A_84_P232439 | 0.0383 | 0.3644 | AT4G31800 | WRKY18 (WRKY DNA-binding protein 18); transcription factor |
| A_84_P12503 | 0.0256 | 0.3642 | AT2G25900 | ATCTH (Arabidopsis thaliana Cys3His zinc finger protein); transcription factor |
| A_84_P768715 | 0.0157 | 0.3636 | AT5G15265 | hypothetical protein |
| A_84_P10585 | 0.0026 | 0.3632 | AT1G62430 | protein coding |
| A_84_P217328 | 2.00E-04 | 0.3632 | AT4G05070 | hypothetical protein |
| A_84_P587454 | 0.0033 | 0.3626 | AT3G19850 | protein coding |
| A_84_P307240 | 0.0237 | 0.3621 | AT2G01670 | ATNUDT17 (Arabidopsis thaliana Nudix hydrolase homolog 17); hydrolase |
| A_84_P837722 | 0.0218 | 0.362 | AT2G38255 | hypothetical protein |
| A_84_P151158 | 0.0032 | 0.3619 | AT5G56550 | hypothetical protein |
| A_84_P23818 | 0.0167 | 0.3616 | AT1G78960 | protein coding |
| A_84_P600095 | 0.0248 | 0.3616 | AT4G19645 | hypothetical protein |
| A_84_P764095 | 0.0432 | 0.3612 | AT4G29850 | hypothetical protein |
| A_84_P23073 | 0.008 | 0.3611 | AT3G26790 | protein coding |
| A_84_P807916 | 0.0302 | 0.361 | AT2G38390 | peroxidase, putative |
| A_84_P791432 | 0.0048 | 0.3608 | AT2G23400 | dehydrodolichyl diphosphate synthase, putative / DEDOL-PP synthase, putative |
| A_84_P16720 | 0.0426 | 0.3606 | AT4G34880 | amidase family protein |
| A_84_P11284 | 0.0482 | 0.3605 | AT5G67520 | adenylylsulfate kinase, putative |
| A_84_P15417 | 0.0048 | 0.3604 | AT2G05940 | protein kinase, putative |
| A_84_P10721 | 0.0179 | 0.3602 | AT1G07280 | protein coding |
| A_84_P21890 | 0.0186 | 0.3602 | AT1G70740 | protein coding |
| A_84_P752529 | 0.0367 | 0.3596 | AT1G22370 | protein coding |
| A_84_P760811 | 0.031 | 0.3582 | AT3G58900 | protein coding |
| A_84_P145509 | 0.0045 | 0.3578 | AT3G50840 | protein coding |
| A_84_P14870 | 0.0298 | 0.3578 | AT5G02260 | ATEXPA9 (ARABIDOPSIS THALIANA EXPANSIN A9) |
| A_84_P15168 | 0.0147 | 0.3577 | AT1G05300 | protein coding |
| A_84_P20509 | 0.0277 | 0.3575 | AT1G48260 | protein coding |
| A_84_P14142 | 0.0095 | 0.3568 | AT5G09480 | hydroxyproline-rich glycoprotein family protein |
| A_84_P290124 | 0.0311 | 0.3567 | AT2G22860 | ATPSK2 (PHYTOSULFOKINE 2 PRECURSOR); growth factor |
| A_84_P10523 | 0.0048 | 0.3566 | AT1G34040 | protein coding |
| A_84_P523919 | 0.008 | 0.3563 | AT5G56230 | prenylated rab acceptor (PRA1) family protein |
| A_84_P178544 | 0.0446 | 0.3559 | AT1G56220 | protein coding |
| A_84_P13727 | 0.0342 | 0.3559 | AT3G54810 | protein coding |
| A_84_P10568 | 0.0341 | 0.3555 | AT1G07000 | protein coding |
| A_84_P270200 | 0.0414 | 0.3552 | AT2G47200 | hypothetical protein |
| A_84_P559517 | 0.0473 | 0.3544 | AT1G28330 | protein coding |
| A_84_P171153 | 0.0019 | 0.354 | AT2G37940 | hypothetical protein |
| A_84_P18760 | 0.0089 | 0.3535 | AT5G44980 | F-box family protein |
| A_84_P216618 | 0.0107 | 0.3534 | AT5G35560 | DENN (AEX-3) domain-containing protein |
| A_84_P20235 | 0.0353 | 0.3533 | AT3G26770 | protein coding |
| A_84_P19444 | 0.005 | 0.3531 | AT1G12160 | protein coding |
| A_84_P16522 | 0.0272 | 0.3526 | AT3G45640 | protein coding |
| A_84_P15485 | 0.001 | 0.3526 | AT3G11820 | protein coding |
| A_84_P304680 | 0.0186 | 0.3524 | AT2G30600 | BTB/POZ domain-containing protein |
| A_84_P133885 | 0.0021 | 0.3519 | AT3G52710 | protein coding |
| A_84_P207698 | 0.0037 | 0.3519 | AT3G52070 | protein coding |
| A_84_P13790 | 0.0403 | 0.3513 | AT4G08950 | phosphate-responsive protein, putative (EXO) |
| A_84_P579587 | 0.0255 | 0.3512 | AT4G20830 | FAD-binding domain-containing protein |
| A_84_P17921 | 0.0174 | 0.3505 | AT5G19550 | ASP2 (ASPARTATE AMINOTRANSFERASE 2) |
| A_84_P13029 | 0.0159 | 0.3502 | AT5G18630 | lipase class 3 family protein |
| A_84_P100536 | 0.0222 | 0.3501 | AT5G03470 | ATB' ALPHA (PP2A, B' subunit, alpha isoform); protein phosphatase type 2A regulator |
| A_84_P11669 | 0.0311 | 0.3497 | AT2G03530 | UPS2 (UREIDE PERMEASE 2) |
| A_84_P760268 | 0.0311 | 0.3494 | AT3G56825 | ncRNA |
| A_84_P849853 | 0.0444 | 0.349 | AT1G67865 | protein coding |
| A_84_P78785 | 0.0089 | 0.3481 | AT3G03650 | protein coding |
| A_84_P765711 | 0.0311 | 0.348 | AT4G24026 | hypothetical protein |
| A_84_P119812 | 0.0146 | 0.3472 | AT1G50740 | protein coding |
| A_84_P53240 | 0.0121 | 0.3467 | AT1G22160 | protein coding |
| A_84_P284070 | 0.0064 | 0.3465 | AT3G15530 | protein coding |
| A_84_P21800 | 0.0132 | 0.3462 | AT1G70290 | protein coding |
| A_84_P11184 | 0.0351 | 0.3442 | AT1G72240 | protein coding |
| A_84_P21710 | 0.0021 | 0.3438 | AT1G28070 | protein coding |
| A_84_P10646 | 0.0174 | 0.3435 | AT2G14070 | wound-responsive protein-related |
| A_84_P159375 | 0.0185 | 0.3434 | AT4G38080 | hydroxyproline-rich glycoprotein family protein |
| A_84_P763062 | 0.0072 | 0.3429 | AT4G22710 | CYP706A2 (cytochrome P450, family 706, subfamily A, polypeptide 2); oxygen binding |
| A_84_P573310 | 0.0185 | 0.3423 | AT4G01330 | protein kinase family protein |
| A_84_P520746 | 0.005 | 0.3423 | AT2G37530 | hypothetical protein |
| A_84_P207868 | 0.0141 | 0.3421 | AT4G00940 | Dof-type zinc finger domain-containing protein |
| A_84_P802490 | 0.0384 | 0.3419 | AT1G01180 | protein coding |
| A_84_P262890 | 0.0066 | 0.3418 | AT4G17215 | hypothetical protein |
| A_84_P847330 | 0.0119 | 0.3416 | AT3G46970 | protein coding |
| A_84_P17009 | 0.0427 | 0.3415 | AT1G20823 | protein coding |
| A_84_P14280 | 0.0055 | 0.3414 | AT1G08290 | protein coding |
| A_84_P792801 | 0.0054 | 0.3414 | AT3G18530 | protein coding |
| A_84_P135955 | 0.0229 | 0.3413 | AT4G29980 | hypothetical protein |
| A_84_P558862 | 0.0195 | 0.3413 | AT2G39705 | DVL11/RTFL8 (ROTUNDIFOLIA LIKE 8) |
| A_84_P846020 | 0.0015 | 0.3411 | AT4G35785 | transformer serine/arginine-rich ribonucleoprotein, putative |
| A_84_P859794 | 0.041 | 0.3405 | AT2G45170 | AtATG8e (AUTOPHAGY 8E); microtubule binding |
| A_84_P14030 | 0.011 | 0.3397 | AT5G44730 | haloacid dehalogenase-like hydrolase family protein |
| A_84_P175431 | 0.0064 | 0.3392 | AT5G10750 | hypothetical protein |
| A_84_P21670 | 0.0281 | 0.3385 | AT5G63800 | BGAL6/MUM2 (MUCILAGE-MODIFIED 2); beta-galactosidase |
| A_84_P17499 | 0.0319 | 0.3382 | AT3G52800 | protein coding |
| A_84_P61310 | 0.0054 | 0.3372 | AT5G63595 | flavonol synthase, putative |
| A_84_P18380 | 0.0071 | 0.3371 | AT1G12950 | protein coding |
| A_84_P18419 | 0.0043 | 0.337 | AT3G46590 | protein coding |
| A_84_P21910 | 0.0066 | 0.337 | AT1G12710 | protein coding |
| A_84_P296414 | 0.0494 | 0.3369 | AT5G57500 | transferase, transferring glycosyl groups |
| A_84_P10793 | 0.0203 | 0.3366 | AT3G23150 | protein coding |
| A_84_P68014 | 0.0122 | 0.3361 | AT4G34150 | C2 domain-containing protein |
| A_84_P23535 | 3.00E-04 | 0.3357 | AT5G57630 | CIPK21 (CBL-INTERACTING PROTEIN KINASE 21); kinase |
| A_84_P829110 | 0.0305 | 0.3351 | AT4G30350 | heat shock protein-related |
| A_84_P203368 | 0.033 | 0.3348 | AT3G28840 | protein coding |
| A_84_P22055 | 0.006 | 0.3344 | AT2G38380 | peroxidase 22 (PER22) (P22) (PRXEA) / basic peroxidase E |
| A_84_P16283 | 0.0103 | 0.3344 | AT2G43290 | MSS3 (MULTICOPY SUPPRESSORS OF SNF4 DEFICIENCY IN YEAST 3); calcium ion binding |
| A_84_P757747 | 0.0131 | 0.3344 | AT2G35945 | misc_RNA |
| A_84_P123242 | 0.0212 | 0.3344 | AT5G40460 | hypothetical protein |
| A_84_P20120 | 0.0193 | 0.3343 | AT2G27690 | CYP94C1 (cytochrome P450, family 94, subfamily C, polypeptide 1); oxygen binding |
| A_84_P12720 | 0.041 | 0.334 | AT3G13750 | protein coding |
| A_84_P583681 | 0.0178 | 0.3335 | AT1G77150 | protein coding |
| A_84_P184164 | 0.011 | 0.3328 | AT1G07090 | protein coding |
| A_84_P731072 | 0.0468 | 0.3326 | AT2G27830 | hypothetical protein |
| A_84_P767724 | 0.0044 | 0.3315 | AT5G54075 | ncRNA |
| A_84_P22178 | 0.0355 | 0.3308 | AT3G27150 | protein coding |
| A_84_P162983 | 0.0326 | 0.3307 | AT2G43010 | PIF4 (PHYTOCHROME INTERACTING FACTOR 4); DNA binding / transcription factor |
| A_84_P110902 | 0.0285 | 0.3303 | AT5G60680 | hypothetical protein |
| A_84_P19074 | 0.0297 | 0.3297 | AT1G18570 | protein coding |
| A_84_P533281 | 0.0088 | 0.3293 | AT3G18300 | protein coding |
| A_84_P521593 | 0.0243 | 0.329 | AT2G20515 | hypothetical protein |
| A_84_P182154 | 0.005 | 0.3289 | AT5G49730 | ATFRO6/FRO6 (FERRIC REDUCTION OXIDASE 6); ferric-chelate reductase/ oxidoreductase |
| A_84_P18029 | 0.0227 | 0.3288 | AT1G10550 | protein coding |
| A_84_P18886 | 0.0018 | 0.3287 | AT1G20620 | protein coding |
| A_84_P723766 | 0.0016 | 0.3284 | AT3G55850 | protein coding |
| A_84_P22719 | 0.04 | 0.3282 | AT1G03620 | protein coding |
| A_84_P603350 | 0.0052 | 0.3281 | AT2G28660 | copper-binding family protein |
| A_84_P23977 | 0.0358 | 0.3269 | AT3G09010 | protein coding |
| A_84_P20795 | 0.0458 | 0.3264 | AT1G15890 | protein coding |
| A_84_P15934 | 0.0406 | 0.3262 | AT5G47960 | SMG1 (SMALL MOLECULAR WEIGHT G-PROTEIN 1); GTP binding |
| A_84_P835924 | 0.024 | 0.326 | AT3G18770 | protein coding |
| A_84_P19586 | 0.0426 | 0.326 | AT4G16600 | glycogenin glucosyltransferase (glycogenin)-related |
| A_84_P110642 | 0.0254 | 0.3246 | AT2G36320 | zinc finger (AN1-like) family protein |
| A_84_P799565 | 0.0406 | 0.3245 | AT1G21100 | protein coding |
| A_84_P809753 | 0.0404 | 0.3243 | AT3G59940 | protein coding |
| A_84_P767547 | 2.00E-04 | 0.3238 | AT5G50645 | hypothetical protein |
| A_84_P13746 | 0.0044 | 0.3234 | AT3G59350 | protein coding |
| A_84_P23166 | 0.0371 | 0.323 | AT3G50700 | protein coding |
| A_84_P22522 | 0.0038 | 0.3224 | AT5G39020 | protein kinase family protein |
| A_84_P10178 | 0.0252 | 0.322 | AT5G12050 | hypothetical protein |
| A_84_P861603 | 0.0173 | 0.3217 | AT4G38930 | ubiquitin fusion degradation UFD1 family protein |
| A_84_P12218 | 0.006 | 0.3217 | AT5G64870 | hypothetical protein |
| A_84_P22593 | 0.0082 | 0.3202 | AT1G01640 | protein coding |
| A_84_P20232 | 0.0226 | 0.3195 | AT3G14310 | protein coding |
| A_84_P17660 | 0.0033 | 0.3188 | AT4G33490 | aspartic-type endopeptidase/ pepsin A |
| A_84_P591780 | 0.0096 | 0.3186 | AT2G26695 | binding |
| A_84_P71404 | 0.013 | 0.3171 | AT3G15840 | protein coding |
| A_84_P14319 | 0.0039 | 0.3154 | AT1G63800 | protein coding |
| A_84_P119892 | 0.039 | 0.3151 | AT4G17670 | senescence-associated protein-related |
| A_84_P21203 | 0.0425 | 0.3147 | AT1G26730 | protein coding |
| A_84_P737916 | 0.0262 | 0.3141 | AT2G01818 | zinc ion binding |
| A_84_P304080 | 3.00E-04 | 0.3138 | AT4G36930 | SPT (SPATULA); DNA binding / transcription factor |
| A_84_P848547 | 0.0368 | 0.3137 | AT5G43780 | APS4 |
| A_84_P10473 | 0.0212 | 0.3131 | AT1G59870 | protein coding |
| A_84_P23050 | 0.0448 | 0.3131 | AT3G04720 | protein coding |
| A_84_P23633 | 0.019 | 0.3125 | AT1G43910 | protein coding |
| A_84_P532174 | 0.0355 | 0.3123 | AT1G78815 | protein coding |
| A_84_P23391 | 0.0402 | 0.3121 | AT1G62840 | protein coding |
| A_84_P21802 | 0.0422 | 0.312 | AT1G78780 | protein coding |
| A_84_P230699 | 0.0149 | 0.3119 | AT5G05530 | zinc finger (C3HC4-type RING finger) family protein |
| A_84_P262420 | 0.0055 | 0.3118 | AT2G33050 | leucine-rich repeat family protein |
| A_84_P19768 | 8.00E-04 | 0.3118 | AT5G61430 | ANAC100/ATNAC5 (Arabidopsis NAC domain containing protein 100); transcription factor |
| A_84_P291354 | 0.0362 | 0.3114 | AT1G10990 | protein coding |
| A_84_P16007 | 3.00E-04 | 0.3109 | AT5G66880 | SNRK2-3/SNRK2.3/SRK2I (SNF1-RELATED PROTEIN KINASE 2.3); kinase/ protein kinase |
| A_84_P21049 | 0.0259 | 0.3108 | AT2G26560 | PLP2 (PHOSPHOLIPASE A 2A); nutrient reservoir |
| A_84_P17717 | 0.0067 | 0.3101 | AT5G05320 | monooxygenase, putative (MO3) |
| A_84_P852797 | 0.0107 | 0.3099 | AT1G19270 | protein coding |
| A_84_P10481 | 0.0318 | 0.3096 | AT1G14040 | protein coding |
| A_84_P18681 | 0.0282 | 0.3086 | AT5G10520 | RBK1 (ROP BINDING PROTEIN KINASES 1); kinase |
| A_84_P765675 | 0.0131 | 0.3079 | AT4G39404 | misc_RNA |
| A_84_P598157 | 0.0228 | 0.3076 | AT3G19150 | protein coding |
| A_84_P12886 | 0.0129 | 0.3076 | AT4G23130 | CRK5 (CYSTEINE-RICH RLK5); kinase |
| A_84_P825275 | 0.0016 | 0.3073 | AT1G61260 | protein coding |
| A_84_P851315 | 0.0087 | 0.3073 | AT1G63750 | protein coding |
| A_84_P132645 | 0.0115 | 0.3064 | AT2G20670 | hypothetical protein |
| A_84_P855355 | 0.0109 | 0.3063 | AT5G49740 | ATFRO7/FRO7 (FERRIC REDUCTION OXIDASE 7); ferric-chelate reductase/ oxidoreductase |
| A_84_P174511 | 0.0028 | 0.3044 | AT5G16110 | hypothetical protein |
| A_84_P23687 | 0.0012 | 0.3039 | AT1G27290 | protein coding |
| A_84_P789365 | 0.0436 | 0.3039 | AT3G61060 | protein coding |
| A_84_P299940 | 0.0451 | 0.3037 | AT1G01355 | protein coding |
| A_84_P841439 | 0.0219 | 0.3017 | AT4G09260 | hypothetical protein |
| A_84_P20050 | 0.0245 | 0.3015 | AT1G14370 | protein coding |
| A_84_P24128 | 0.0182 | 0.3015 | AT3G54150 | protein coding |
| A_84_P19482 | 0.034 | 0.3015 | AT4G13300 | ATTPS13/TPS13 (TERPENOID SYNTHASE13); cyclase |
| A_84_P11150 | 7.00E-04 | 0.3013 | AT5G24590 | ANAC091/TIP (TCV-INTERACTING PROTEIN); transcription coactivator/ transcription factor |
| A_84_P294174 | 0.0108 | 0.301 | AT1G69810 | protein coding |
| A_84_P11803 | 0.0385 | 0.3008 | AT3G46930 | protein coding |
| A_84_P824914 | 0.0128 | 0.3008 | AT4G18205 | AT4G18200 |
| A_84_P19754 | 0.0473 | 0.3002 | AT5G58540 | protein kinase family protein |
| A_84_P20450 | 0.0073 | 0.2999 | AT4G22770 | DNA-binding family protein |
| A_84_P10224 | 0.0011 | 0.2991 | AT5G37770 | TCH2 (TOUCH 2); calcium ion binding |
| A_84_P825020 | 6.00E-04 | 0.298 | AT1G22790 | protein coding |
| A_84_P18180 | 0.0499 | 0.2973 | AT2G31880 | leucine-rich repeat transmembrane protein kinase, putative |
| A_84_P242573 | 0.0179 | 0.2964 | AT5G54470 | zinc finger (B-box type) family protein |
| A_84_P739698 | 0.0294 | 0.2956 | AT3G06900 | ncRNA |
| A_84_P14602 | 0.0103 | 0.2949 | AT1G59590 | protein coding |
| A_84_P796437 | 0.0409 | 0.2949 | AT1G80160 | protein coding |
| A_84_P19414 | 0.0188 | 0.2948 | AT3G57680 | protein coding |
| A_84_P10978 | 0.0344 | 0.294 | AT4G18550 | lipase class 3 family protein |
| A_84_P21825 | 0.0167 | 0.2937 | AT1G13210 | protein coding |
| A_84_P14249 | 0.0109 | 0.2929 | AT1G36060 | protein coding |
| A_84_P22322 | 0.0091 | 0.2928 | AT4G13810 | disease resistance family protein / LRR family protein |
| A_84_P23960 | 0.0381 | 0.2918 | AT2G44590 | ADL1D; GTP binding / GTPase |
| A_84_P545146 | 0.016 | 0.2917 | AT5G40180 | hypothetical protein |
| A_84_P12212 | 0.0479 | 0.2912 | AT1G56060 | protein coding |
| A_84_P18081 | 0.0056 | 0.2909 | AT1G76410 | protein coding |
| A_84_P17189 | 0.0173 | 0.2905 | AT1G25560 | protein coding |
| A_84_P21584 | 4.00E-04 | 0.2904 | AT1G72210 | protein coding |
| A_84_P551899 | 0.0443 | 0.2902 | AT3G28100 | protein coding |
| A_84_P724318 | 0.0021 | 0.2899 | AT5G06865 | miscRNA |
| A_84_P274410 | 0.0458 | 0.2896 | AT1G68330 | protein coding |
| A_84_P11385 | 0.0172 | 0.289 | AT1G09070 | protein coding |
| A_84_P15840 | 0.0083 | 0.2887 | AT1G31290 | protein coding |
| A_84_P804573 | 0.0027 | 0.2887 | AT2G43150 | proline-rich extensin-like family protein |
| A_84_P13104 | 0.0183 | 0.2886 | AT5G49520 | WRKY48 (WRKY DNA-binding protein 48); transcription factor |
| A_84_P230649 | 0.0372 | 0.2885 | AT5G37740 | C2 domain-containing protein |
| A_84_P151098 | 0.0283 | 0.2875 | AT1G70690 | protein coding |
| A_84_P247225 | 0.0112 | 0.2868 | AT1G01130 | protein coding |
| A_84_P17400 | 0.0341 | 0.2867 | AT3G14050 | protein coding |
| A_84_P266280 | 0.0294 | 0.2867 | AT4G01610 | cathepsin B-like cysteine protease, putative |
| A_84_P21836 | 0.0015 | 0.2866 | AT1G72940 | protein coding |
| A_84_P12357 | 0.0336 | 0.2862 | AT1G10480 | protein coding |
| A_84_P750624 | 0.0436 | 0.2859 | AT1G11580 | protein coding |
| A_84_P133525 | 0.0107 | 0.2857 | AT3G49780 | protein coding |
| A_84_P18593 | 0.0076 | 0.2855 | AT4G30370 | zinc finger (C3HC4-type RING finger) family protein |
| A_84_P21822 | 0.0069 | 0.2853 | AT1G21000 | protein coding |
| A_84_P22983 | 0.0121 | 0.2849 | AT2G18670 | zinc finger (C3HC4-type RING finger) family protein |
| A_84_P169283 | 0.0338 | 0.2847 | AT5G06570 | hydrolase |
| A_84_P13568 | 0.0421 | 0.2842 | AT2G33830 | dormancy/auxin associated family protein |
| A_84_P759968 | 0.0098 | 0.284 | AT3G57930 | protein coding |
| A_84_P564464 | 0 | 0.2837 | AT4G27460 | CBS domain-containing protein |
| A_84_P56760 | 0.0072 | 0.2836 | AT3G19680 | protein coding |
| A_84_P798326 | 0.0046 | 0.2833 | AT5G50670 | squamosa promoter-binding protein, putative |
| A_84_P813144 | 3.00E-04 | 0.2827 | AT3G58790 | protein coding |
| A_84_P90769 | 0.0024 | 0.2824 | AT2G24240 | potassium channel tetramerisation domain-containing protein |
| A_84_P16271 | 0.0036 | 0.2818 | AT1G73330 | protein coding |
| A_84_P11054 | 0.0116 | 0.2813 | AT4G36040 | DNAJ heat shock N-terminal domain-containing protein (J11) |
| A_84_P595270 | 0.0021 | 0.2808 | AT2G01580 | hypothetical protein |
| A_84_P23415 | 0.0068 | 0.2782 | AT5G11570 | proton-dependent oligopeptide transport (POT) family protein |
| A_84_P14895 | 0.0309 | 0.2781 | AT5G10050 | short-chain dehydrogenase/reductase (SDR) family protein |
| A_84_P22312 | 0.0063 | 0.2781 | AT4G11360 | RHA1B (RING-H2 finger A1B); protein binding / zinc ion binding |
| A_84_P90969 | 0.0471 | 0.278 | AT5G64800 | CLE21 (CLAVATA3/ESR-RELATED 21); receptor binding |
| A_84_P216308 | 0.005 | 0.2779 | AT2G47270 | transcription factor/ transcription regulator |
| A_84_P17683 | 0.0378 | 0.2777 | AT4G39580 | kelch repeat-containing F-box family protein |
| A_84_P96276 | 0.0018 | 0.2776 | AT5G60100 | APRR3 (PSEUDO-RESPONSE REGULATOR 3); transcription regulator |
| A_84_P14471 | 0.049 | 0.2773 | AT2G36950 | heavy-metal-associated domain-containing protein |
| A_84_P23512 | 0.0121 | 0.2773 | AT5G50950 | fumarate hydratase, putative / fumarase, putative |
| A_84_P93839 | 0.0052 | 0.277 | AT1G78460 | protein coding |
| A_84_P297414 | 0.0076 | 0.2767 | AT3G62070 | protein coding |
| A_84_P17011 | 0.0335 | 0.2764 | AT1G15850 | protein coding |
| A_84_P16571 | 0.0401 | 0.2762 | AT3G56400 | protein coding |
| A_84_P138149 | 0.028 | 0.2758 | AT1G72800 | protein coding |
| A_84_P861998 | 0.0038 | 0.2745 | AT1G32790 | protein coding |
| A_84_P825325 | 0.0058 | 0.2745 | AT5G57340 | hypothetical protein |
| A_84_P21296 | 0.018 | 0.2744 | AT3G55430 | protein coding |
| A_84_P146578 | 0.0021 | 0.2743 | AT4G32020 | hypothetical protein |
| A_84_P596321 | 0.0257 | 0.2733 | AT3G06435 | protein coding |
| A_84_P509078 | 0.0416 | 0.273 | AT1G69900 | protein coding |
| A_84_P503493 | 0.0072 | 0.2726 | AT5G49015 | Expressed protein |
| A_84_P531612 | 0.0052 | 0.2724 | AT4G04745 | hypothetical protein |
| A_84_P14131 | 0.0307 | 0.2718 | AT5G20250 | DIN10 (DARK INDUCIBLE 10); hydrolase, hydrolyzing O-glycosyl compounds |
| A_84_P72634 | 0.0035 | 0.2711 | AT4G36500 | hypothetical protein |
| A_84_P518558 | 0.0425 | 0.271 | AT5G47060 | senescence-associated protein-related |
| A_84_P19082 | 0.0236 | 0.27 | AT1G22190 | protein coding |
| A_84_P17357 | 0.0051 | 0.2673 | AT3G10300 | protein coding |
| A_84_P843500 | 0.0048 | 0.2664 | AT2G15910 | CSL zinc finger domain-containing protein |
| A_84_P12969 | 0.0431 | 0.2661 | AT4G17230 | SCL13 (SCARECROW-LIKE 13); transcription factor |
| A_84_P516656 | 0.004 | 0.2659 | AT4G35985 | senescence/dehydration-associated protein-related |
| A_84_P19313 | 0.0365 | 0.2652 | AT3G26160 | protein coding |
| A_84_P261290 | 0.0027 | 0.2642 | AT1G31540 | protein coding |
| A_84_P21537 | 0.0203 | 0.2641 | AT5G17850 | cation exchanger, putative (CAX8) |
| A_84_P14935 | 0.0032 | 0.2624 | AT5G26030 | FC1 (FERROCHELATASE 1); ferrochelatase |
| A_84_P216078 | 0.0441 | 0.2623 | AT5G39785 | structural constituent of ribosome |
| A_84_P10831 | 0.0127 | 0.2615 | AT3G25600 | protein coding |
| A_84_P103806 | 0.0019 | 0.2611 | AT5G21940 | hypothetical protein |
| A_84_P13976 | 0.0029 | 0.2611 | AT5G19120 | aspartic-type endopeptidase/ pepsin A |
| A_84_P12695 | 0.0186 | 0.261 | AT3G25730 | protein coding |
| A_84_P12862 | 0.004 | 0.2609 | AT4G13190 | kinase |
| A_84_P12934 | 0.0304 | 0.2608 | AT4G33920 | protein phosphatase 2C family protein / PP2C family protein |
| A_84_P823010 | 0.0361 | 0.2607 | AT2G48140 | EDA4 (embryo sac development arrest 4); lipid binding |
| A_84_P223589 | 0.0365 | 0.2601 | AT4G16000 | hypothetical protein |
| A_84_P305680 | 3.00E-04 | 0.2591 | AT4G20390 | integral membrane family protein |
| A_84_P740964 | 0.0444 | 0.2591 | AT5G08760 | hypothetical protein |
| A_84_P829270 | 0.0359 | 0.259 | AT1G66620 | protein coding |
| A_84_P14327 | 0.0131 | 0.2589 | AT1G78090 | protein coding |
| A_84_P533108 | 0.0447 | 0.2588 | AT2G15830 | hypothetical protein |
| A_84_P60770 | 0.025 | 0.2581 | AT5G03230 | hypothetical protein |
| A_84_P299620 | 0.0047 | 0.2565 | AT3G05120 | protein coding |
| A_84_P11614 | 0.0104 | 0.2565 | AT2G16570 | ATASE (GLN PHOSPHORIBOSYL PYROPHOSPHATE AMIDOTRANSFERASE 1); amidophosphoribosyltransferase |
| A_84_P12382 | 0.0397 | 0.2556 | AT1G53830 | protein coding |
| A_84_P769417 | 0.0049 | 0.2538 | AT5G41761 | hypothetical protein |
| A_84_P799139 | 0.0248 | 0.2528 | AT3G52430 | protein coding |
| A_84_P800816 | 0.0096 | 0.2524 | AT3G10985 | protein coding |
| A_84_P24144 | 0.0039 | 0.2516 | AT3G57640 | protein coding |
| A_84_P830058 | 0.0396 | 0.2505 | AT1G63860 | pseudo |
| A_84_P610276 | 0.0216 | 0.2505 | AT1G34315 | protein coding |
| A_84_P21447 | 0.0321 | 0.2498 | AT4G34380 | transducin family protein / WD-40 repeat family protein |
| A_84_P18796 | 0.0043 | 0.2492 | AT5G54960 | PDC2 (PYRUVATE DECARBOXYLASE-2); pyruvate decarboxylase |
| A_84_P17822 | 0.0063 | 0.249 | AT5G47070 | protein kinase, putative |
| A_84_P566826 | 0.0056 | 0.2486 | AT2G34090 | MEE18 (maternal effect embryo arrest 18) |
| A_84_P10198 | 0.0131 | 0.2484 | AT5G22920 | zinc finger (C3HC4-type RING finger) family protein |
| A_84_P528200 | 0.0206 | 0.2467 | AT5G02200 | FHL (FAR-RED-ELONGATED HYPOCOTYL1-LIKE); protein binding |
| A_84_P55800 | 0.0015 | 0.2466 | AT5G12950 | catalytic |
| A_84_P10871 | 0.0235 | 0.2463 | AT3G50280 | protein coding |
| A_84_P15790 | 0.0093 | 0.2459 | AT4G39260 | ATGRP8/GR-RBP8 (COLD, CIRCADIAN RHYTHM, AND RNA BINDING 1, GLYCINE-RICH PROTEIN 8); RNA binding |
| A_84_P17485 | 0.0104 | 0.2458 | AT3G49530 | protein coding |
| A_84_P10372 | 0.0223 | 0.2454 | AT5G38450 | CYP735A1 (cytochrome P450, family 735, subfamily A, polypeptide 1); oxygen binding |
| A_84_P711969 | 0.0134 | 0.245 | AT1G11175 | misc_RNA |
| A_84_P821710 | 0.0284 | 0.2437 | AT5G66390 | peroxidase 72 (PER72) (P72) (PRXR8) |
| A_84_P23217 | 0.0048 | 0.2427 | AT3G62720 | protein coding |
| A_84_P758386 | 0.0061 | 0.2424 | AT2G22496 | misc_RNA |
| A_84_P13987 | 0.0302 | 0.2416 | AT5G25190 | ethylene-responsive element-binding protein, putative |
| A_84_P310313 | 0 | 0.241 | AT2G26190 | calmodulin-binding family protein |
| A_84_P22673 | 4.00E-04 | 0.2406 | AT2G40435 | transcription regulator |
| A_84_P107082 | 0.0213 | 0.2406 | AT3G03270 | protein coding |
| A_84_P768720 | 0.0314 | 0.2394 | AT5G38005 | misc_RNA |
| A_84_P255510 | 0.0015 | 0.239 | AT1G72450 | protein coding |
| A_84_P56360 | 0.0126 | 0.2385 | AT5G57010 | calmodulin-binding family protein |
| A_84_P21458 | 0.0104 | 0.2378 | AT4G37610 | BT5 (BTB and TAZ domain protein 5); protein binding / transcription regulator |
| A_84_P851226 | 0.0292 | 0.2373 | AT3G46640 | protein coding |
| A_84_P752556 | 0.0064 | 0.2371 | AT1G53160 | protein coding |
| A_84_P11360 | 0.0186 | 0.2356 | AT1G52040 | protein coding |
| A_84_P18627 | 0.0315 | 0.2353 | AT4G39090 | RD19 (RESPONSIVE TO DEHYDRATION 19); cysteine-type peptidase |
| A_84_P764462 | 0.0021 | 0.2352 | AT4G03510 | RMA1 (Ring finger protein with Membrane Anchor 1); protein binding / ubiquitin-protein ligase/ zinc |
| A_84_P132245 | 0.0056 | 0.2351 | AT5G50940 | pseudo |
| A_84_P17634 | 0.0094 | 0.235 | AT4G27480 | glycosyltransferase family 14 protein / core-2/I-branching enzyme family protein |
| A_84_P17434 | 0.0017 | 0.2331 | AT3G20600 | protein coding |
| A_84_P854061 | 0.0249 | 0.2326 | AT5G03610 | GDSL-motif lipase/hydrolase family protein |
| A_84_P18010 | 0.0424 | 0.2323 | AT1G77240 | protein coding |
| A_84_P567134 | 0.0364 | 0.2317 | AT4G27657 | hypothetical protein |
| A_84_P12184 | 0.0327 | 0.2316 | AT5G56870 | BGAL4 (beta-galactosidase 4); beta-galactosidase |
| A_84_P11816 | 0.0046 | 0.2314 | AT3G50120 | protein coding |
| A_84_P17597 | 0.0116 | 0.2314 | AT4G18880 | AT-HSFA4A (Arabidopsis thaliana heat shock transcription factor A4A); DNA binding / transcription fa |
| A_84_P13059 | 0.033 | 0.2314 | AT5G37600 | ATGSR1 (Arabidopsis thaliana glutamine synthase clone R1); glutamate-ammonia ligase |
| A_84_P243255 | 0.0219 | 0.2309 | AT5G44260 | zinc finger (CCCH-type) family protein |
| A_84_P283560 | 0.011 | 0.2306 | AT2G24600 | ankyrin repeat family protein |
| A_84_P501178 | 0.0449 | 0.2304 | AT2G23810 | TET8 (TETRASPANIN8) |
| A_84_P13341 | 1.00E-04 | 0.23 | AT1G78270 | protein coding |
| A_84_P260570 | 0.0047 | 0.2299 | AT3G14700 | protein coding |
| A_84_P20175 | 0.0344 | 0.2299 | AT2G01180 | ATPAP1 (PHOSPHATIDIC ACID PHOSPHATASE 1); phosphatidate phosphatase |
| A_84_P13567 | 0.0311 | 0.2295 | AT2G47130 | short-chain dehydrogenase/reductase (SDR) family protein |
| A_84_P18414 | 0.0166 | 0.2293 | AT1G02610 | protein coding |
| A_84_P19477 | 0.047 | 0.2286 | AT4G12080 | DNA-binding family protein |
| A_84_P784441 | 0.0079 | 0.2283 | AT4G18950 | ankyrin protein kinase, putative |
| A_84_P235993 | 0.028 | 0.2279 | AT5G58630 | hypothetical protein |
| A_84_P218338 | 0.028 | 0.2277 | AT2G20920 | hypothetical protein |
| A_84_P542893 | 0.0013 | 0.2273 | AT2G30230 | hypothetical protein |
| A_84_P21267 | 0.0046 | 0.2272 | AT3G49130 | protein coding |
| A_84_P22342 | 0.0061 | 0.2271 | AT4G22780 | ACR7 (ACT Domain Repeat 7) |
| A_84_P166103 | 0.037 | 0.2267 | AT5G08240 | hypothetical protein |
| A_84_P587368 | 0.0229 | 0.2266 | AT2G42140 | VQ motif-containing protein |
| A_84_P13216 | 0.0068 | 0.2266 | AT1G20510 | protein coding |
| A_84_P11140 | 0.0013 | 0.2265 | AT5G18840 | sugar transporter, putative |
| A_84_P279580 | 0.0138 | 0.2261 | AT3G50800 | protein coding |
| A_84_P141879 | 0.0094 | 0.225 | AT3G56780 | protein coding |
| A_84_P545592 | 0.0076 | 0.2248 | AT2G39650 | hypothetical protein |
| A_84_P17015 | 0.0286 | 0.2246 | AT1G02340 | protein coding |
| A_84_P12049 | 0.0227 | 0.2246 | AT5G06720 | peroxidase, putative |
| A_84_P13462 | 0.0432 | 0.2244 | AT2G33580 | protein kinase family protein / peptidoglycan-binding LysM domain-containing protein |
| A_84_P16687 | 0.0034 | 0.2233 | AT1G21920 | protein coding |
| A_84_P10076 | 0.0362 | 0.2231 | AT4G28720 | flavin-containing monooxygenase family protein / FMO family protein |
| A_84_P11080 | 0.0208 | 0.2228 | AT4G17615 | CBL1 (CALCINEURIN B-LIKE PROTEIN 1); calcium ion binding |
| A_84_P85619 | 0.0165 | 0.2227 | AT1G63090 | protein coding |
| A_84_P15883 | 0.0481 | 0.2226 | AT5G26920 | calmodulin binding |
| A_84_P141359 | 0.0029 | 0.2222 | AT2G24550 | hypothetical protein |
| A_84_P22683 | 0.006 | 0.2218 | AT3G29035 | protein coding |
| A_84_P19777 | 0.0304 | 0.2214 | AT5G63560 | transferase family protein |
| A_84_P12962 | 0.0161 | 0.2211 | AT4G15280 | UDP-glucoronosyl/UDP-glucosyl transferase family protein |
| A_84_P22377 | 0.0041 | 0.2208 | AT4G30430 | TET9 (TETRASPANIN9) |
| A_84_P11798 | 0.025 | 0.2207 | AT3G45970 | protein coding |
| A_84_P757111 | 0.0092 | 0.2203 | AT2G31425 | enzyme inhibitor/ pectinesterase |
| A_84_P612122 | 0.0037 | 0.22 | AT1G64065 | protein coding |
| A_84_P210868 | 0.0392 | 0.22 | AT4G16146 | hypothetical protein |
| A_84_P574406 | 0.0184 | 0.2198 | AT5G39240 | hypothetical protein |
| A_84_P286390 | 0.0466 | 0.2194 | AT3G25780 | protein coding |
| A_84_P307510 | 0.0177 | 0.2176 | AT4G08040 | ACS11 (1-Amino-cyclopropane-1-carboxylate synthase 11); 1-aminocyclopropane-1-carboxylate synthase |
| A_84_P753488 | 0.0076 | 0.217 | AT1G13448 | misc_RNA |
| A_84_P12709 | 0.0013 | 0.2166 | AT3G19240 | protein coding |
| A_84_P115752 | 0.0445 | 0.2166 | AT5G42530 | hypothetical protein |
| A_84_P230289 | 0.0078 | 0.2166 | AT2G44080 | ARL (ARGOS-LIKE) |
| A_84_P21002 | 0.0246 | 0.216 | AT1G51090 | protein coding |
| A_84_P69014 | 0.0019 | 0.216 | AT5G51390 | hypothetical protein |
| A_84_P188764 | 1.00E-04 | 0.2158 | AT4G04330 | hypothetical protein |
| A_84_P535780 | 0.0199 | 0.2158 | AT2G22905 | Expressed protein |
| A_84_P118712 | 1.00E-04 | 0.2156 | AT1G56300 | protein coding |
| A_84_P16052 | 5.00E-04 | 0.2156 | AT2G20625 | hypothetical protein |
| A_84_P52990 | 0.0143 | 0.2154 | AT5G16570 | GLN1;4 (Glutamine synthetase 1;4); glutamate-ammonia ligase |
| A_84_P68194 | 0.0023 | 0.2143 | AT5G46710 | zinc-binding family protein |
| A_84_P508448 | 0.0216 | 0.2141 | AT3G23870 | protein coding |
| A_84_P12564 | 0.0197 | 0.2133 | AT2G44500 | hypothetical protein |
| A_84_P17134 | 0.0274 | 0.2128 | AT1G73750 | protein coding |
| A_84_P14902 | 0.0068 | 0.2123 | AT5G11670 | ATNADP-ME2 (NADP-MALIC ENZYME 2); malate dehydrogenase (oxaloacetate-decarboxylating) (NADP+)/ malic |
| A_84_P10363 | 0.0064 | 0.2121 | AT1G28230 | protein coding |
| A_84_P23679 | 0.0081 | 0.2119 | AT1G80920 | protein coding |
| A_84_P21880 | 0.0346 | 0.2116 | AT1G19780 | protein coding |
| A_84_P124252 | 0.0016 | 0.2113 | AT2G41010 | ATCAMBP25 (ARABIDOPSIS THALIANA CALMODULIN (CAM)-BINDING PROTEIN OF 25 KDA); calmodulin binding |
| A_84_P24087 | 0.0426 | 0.2092 | AT3G45060 | protein coding |
| A_84_P577170 | 0.0061 | 0.2092 | AT5G64190 | hypothetical protein |
| A_84_P11714 | 0.0026 | 0.2091 | AT3G04210 | protein coding |
| A_84_P826990 | 0.0291 | 0.2089 | AT1G63720 | protein coding |
| A_84_P11627 | 0.0403 | 0.2086 | AT2G45040 | matrix metalloproteinase |
| A_84_P79759 | 0.0152 | 0.2084 | AT5G62280 | hypothetical protein |
| A_84_P17675 | 0.0065 | 0.2084 | AT4G37780 | AtMYB87/MYB87 (myb domain protein 87); DNA binding / transcription factor |
| A_84_P14468 | 0.0057 | 0.208 | AT1G75450 | protein coding |
| A_84_P549947 | 0.0383 | 0.2076 | AT2G18210 | hypothetical protein |
| A_84_P21202 | 0.0102 | 0.2067 | AT3G12830 | protein coding |
| A_84_P10170 | 0.0253 | 0.2055 | AT5G10140 | FLC (FLOWERING LOCUS C); transcription factor |
| A_84_P14817 | 1.00E-04 | 0.2052 | AT1G27100 | protein coding |
| A_84_P733130 | 0.0161 | 0.2043 | AT1G61275 | ncRNA |
| A_84_P176794 | 0.0433 | 0.204 | AT1G70990 | protein coding |
| A_84_P287800 | 0.0207 | 0.2039 | AT2G44130 | kelch repeat-containing F-box family protein |
| A_84_P64294 | 0.029 | 0.2034 | AT3G23790 | protein coding |
| A_84_P22548 | 0.0086 | 0.2033 | AT5G46080 | protein kinase family protein |
| A_84_P510027 | 0.0184 | 0.2018 | AT2G04495 | hypothetical protein |
| A_84_P18528 | 0.0042 | 0.2014 | AT4G11290 | peroxidase, putative |
| A_84_P16757 | 0.0133 | 0.2014 | AT5G01200 | myb family transcription factor |
| A_84_P18859 | 0.02 | 0.2014 | AT5G28630 | glycine-rich protein |
| A_84_P18935 | 0.0328 | 0.2007 | AT1G80660 | protein coding |
| A_84_P14705 | 0.031 | 0.1998 | AT3G62780 | protein coding |
| A_84_P862538 | 0.0026 | 0.1993 | AT5G20010 | RAN-1 (RAS RELATED NUCLEAR PROTEIN); GTP binding |
| A_84_P270470 | 8.00E-04 | 0.1991 | AT3G54000 | protein coding |
| A_84_P175621 | 0.0492 | 0.199 | AT4G11880 | AGL14 (AGAMOUS-LIKE 14); DNA binding / transcription factor |
| A_84_P18211 | 0.0099 | 0.1979 | AT2G26530 | AR781 |
| A_84_P14905 | 0.0045 | 0.1971 | AT1G18350 | protein coding |
| A_84_P15304 | 0.0013 | 0.1967 | AT1G65390 | protein coding |
| A_84_P720691 | 0.0271 | 0.1966 | AT4G26488 | misc_RNA |
| A_84_P862560 | 0.007 | 0.196 | AT3G17800 | protein coding |
| A_84_P758465 | 0.0183 | 0.1958 | AT3G42130 | protein coding |
| A_84_P586009 | 0.0203 | 0.195 | AT5G28610 | hypothetical protein |
| A_84_P23997 | 0.0293 | 0.1949 | AT3G04060 | protein coding |
| A_84_P285960 | 0.0014 | 0.1945 | AT5G52910 | ATIM (TIMELESS) |
| A_84_P16821 | 0.0402 | 0.1942 | AT5G24470 | APRR5 (PSEUDO-RESPONSE REGULATOR 5); transcription regulator |
| A_84_P125601 | 0.0386 | 0.1929 | AT3G22240 | protein coding |
| A_84_P12113 | 0.023 | 0.1926 | AT1G68600 | protein coding |
| A_84_P12601 | 0.0066 | 0.1923 | AT2G42350 | zinc finger (C3HC4-type RING finger) family protein |
| A_84_P817391 | 0.0028 | 0.1922 | AT2G17710 | hypothetical protein |
| A_84_P17256 | 0.0177 | 0.1913 | AT2G40140 | CZF1/ZFAR1; transcription factor |
| A_84_P14054 | 0.0414 | 0.191 | AT5G51060 | RHD2 (ROOT HAIR DEFECTIVE 2) |
| A_84_P127531 | 0.0223 | 0.1908 | AT1G21310 | protein coding |
| A_84_P16345 | 0.0173 | 0.1903 | AT2G27220 | BLH5 (BELL1-LIKE HOMEODOMAIN 5); DNA binding / transcription factor |
| A_84_P19149 | 0.0237 | 0.1902 | AT2G18370 | protease inhibitor/seed storage/lipid transfer protein (LTP) family protein |
| A_84_P14321 | 0.0061 | 0.1898 | AT1G74440 | protein coding |
| A_84_P521683 | 0.0037 | 0.1894 | AT2G46970 | PIL1 (PHYTOCHROME INTERACTING FACTOR 3-LIKE 1); transcription factor |
| A_84_P12642 | 0.0181 | 0.1889 | AT3G10020 | protein coding |
| A_84_P593234 | 0.0088 | 0.1888 | AT5G66070 | zinc finger (C3HC4-type RING finger) family protein |
| A_84_P18198 | 0.0272 | 0.1884 | AT2G16660 | nodulin family protein |
| A_84_P290684 | 0.0467 | 0.1882 | AT4G17490 | ATERF6 (ETHYLENE RESPONSIVE ELEMENT BINDING FACTOR 6); DNA binding / transcription factor |
| A_84_P76134 | 0.0052 | 0.1876 | AT4G14270 | hypothetical protein |
| A_84_P12182 | 0.0163 | 0.1876 | AT5G56300 | GAMT2; S-adenosylmethionine-dependent methyltransferase/ gibberellin carboxyl-O-methyltransferase |
| A_84_P765122 | 0.0438 | 0.1865 | AT3G56210 | protein coding |
| A_84_P18532 | 0.0112 | 0.185 | AT4G12290 | copper amine oxidase, putative |
| A_84_P16754 | 0.0019 | 0.1838 | AT4G36740 | ATHB40/HB-5 (ARABIDOPSIS THALIANA HOMEOBOX PROTEIN 40); DNA binding / transcription factor |
| A_84_P60290 | 0.001 | 0.1812 | AT5G65300 | hypothetical protein |
| A_84_P17461 | 0.0027 | 0.1804 | AT3G43430 | protein coding |
| A_84_P21994 | 0.0285 | 0.1802 | AT2G26440 | pectinesterase family protein |
| A_84_P16813 | 0.0059 | 0.1801 | AT5G19140 | auxin/aluminum-responsive protein, putative |
| A_84_P16851 | 0.0208 | 0.1792 | AT5G40240 | nodulin MtN21 family protein |
| A_84_P23164 | 0.0474 | 0.1778 | AT3G50260 | protein coding |
| A_84_P817057 | 0.0425 | 0.1774 | AT3G50930 | protein coding |
| A_84_P204118 | 0.0118 | 0.1767 | AT5G50450 | zinc finger (MYND type) family protein |
| A_84_P11197 | 0.0017 | 0.1762 | AT5G45110 | NPR3 (NPR1-LIKE PROTEIN 3); protein binding |
| A_84_P536372 | 0.016 | 0.1755 | AT5G66640 | LIM domain-containing protein-related |
| A_84_P16877 | 0.0126 | 0.175 | AT5G47240 | ATNUDT8 (Arabidopsis thaliana Nudix hydrolase homolog 8); hydrolase |
| A_84_P16214 | 0.0203 | 0.1746 | AT1G70800 | protein coding |
| A_84_P754635 | 0.0031 | 0.1736 | AT1G70581 | misc_RNA |
| A_84_P17392 | 0.0106 | 0.1731 | AT3G16720 | protein coding |
| A_84_P13751 | 0.0101 | 0.173 | AT3G60490 | protein coding |
| A_84_P754341 | 7.00E-04 | 0.1723 | AT1G53887 | protein coding |
| A_84_P19361 | 0.0241 | 0.1717 | AT3G45860 | protein coding |
| A_84_P103346 | 0.0212 | 0.1708 | AT1G74940 | protein coding |
| A_84_P19320 | 0.0168 | 0.1707 | AT3G16510 | protein coding |
| A_84_P17759 | 0.0013 | 0.1707 | AT5G19160 | hypothetical protein |
| A_84_P242113 | 0.0092 | 0.1694 | AT4G19520 | disease resistance protein (TIR-NBS-LRR class), putative |
| A_84_P51550 | 0.0398 | 0.1691 | AT2G40750 | WRKY54 (WRKY DNA-binding protein 54); transcription factor |
| A_84_P18782 | 0.004 | 0.1689 | AT1G64380 | protein coding |
| A_84_P19561 | 0.0146 | 0.1659 | AT4G35480 | RHA3B (RING-H2 finger A3B); protein binding / zinc ion binding |
| A_84_P275050 | 0.0076 | 0.1638 | AT3G27210 | protein coding |
| A_84_P16605 | 0.0128 | 0.163 | AT4G00870 | basic helix-loop-helix (bHLH) family protein |
| A_84_P177674 | 0.0016 | 0.1618 | AT5G25210 | hypothetical protein |
| A_84_P606215 | 0.037 | 0.1611 | AT3G50470 | protein coding |
| A_84_P249535 | 0.0143 | 0.1608 | AT1G13340 | protein coding |
| A_84_P13913 | 0.0094 | 0.16 | AT1G19380 | protein coding |
| A_84_P16437 | 0.0211 | 0.1596 | AT3G09870 | protein coding |
| A_84_P595174 | 0.001 | 0.1596 | AT1G50750 | protein coding |
| A_84_P23204 | 0.0492 | 0.1595 | AT3G59480 | protein coding |
| A_84_P19239 | 0.0175 | 0.1594 | AT1G44830 | protein coding |
| A_84_P21194 | 0.0234 | 0.1593 | AT3G23240 | protein coding |
| A_84_P88889 | 0.0042 | 0.159 | AT2G05580 | pseudo |
| A_84_P825767 | 0.0054 | 0.1588 | AT1G56240 | protein coding |
| A_84_P503102 | 5.00E-04 | 0.1587 | AT3G15440 | protein coding |
| A_84_P757973 | 0.0325 | 0.1582 | AT2G44578 | protein binding / zinc ion binding |
| A_84_P22512 | 0.0254 | 0.1577 | AT5G35580 | kinase |
| A_84_P16468 | 0.0386 | 0.1576 | AT3G26740 | protein coding |
| A_84_P14304 | 0.0491 | 0.1575 | AT1G50560 | protein coding |
| A_84_P14978 | 0.019 | 0.1574 | AT5G45380 | sodium:solute symporter family protein |
| A_84_P23975 | 0.0152 | 0.1574 | AT3G01080 | protein coding |
| A_84_P12321 | 0.0217 | 0.1573 | AT1G61290 | protein coding |
| A_84_P289354 | 0.0183 | 0.1559 | AT2G15890 | MEE14 (maternal effect embryo arrest 14) |
| A_84_P91089 | 0.0285 | 0.1559 | AT5G39100 | GLP6 (GERMIN-LIKE PROTEIN 6); manganese ion binding / metal ion binding / nutrient reservoir |
| A_84_P16120 | 0.0069 | 0.1554 | AT1G10140 | protein coding |
| A_84_P11125 | 0.0182 | 0.1546 | AT5G12880 | proline-rich family protein |
| A_84_P23396 | 0.0068 | 0.1541 | AT5G06320 | NHL3 (NDR1/HIN1-like 3) |
| A_84_P59510 | 0.0072 | 0.1537 | AT4G32480 | hypothetical protein |
| A_84_P292144 | 0.0412 | 0.1533 | AT3G23550 | protein coding |
| A_84_P14748 | 0.0118 | 0.1533 | AT1G17420 | protein coding |
| A_84_P828978 | 0.0112 | 0.1532 | AT3G45960 | protein coding |
| A_84_P13816 | 0.0012 | 0.1531 | AT4G19420 | pectinacetylesterase family protein |
| A_84_P606349 | 0.0274 | 0.1531 | AT4G28460 | hypothetical protein |
| A_84_P10158 | 0.0035 | 0.1525 | AT5G06690 | (THIOREDOXIN-LIKE 5); thiol-disulfide exchange intermediate |
| A_84_P18291 | 0.0018 | 0.1524 | AT2G27500 | glycosyl hydrolase family 17 protein |
| A_84_P23180 | 0.0071 | 0.1522 | AT3G53800 | protein coding |
| A_84_P12550 | 0.0336 | 0.152 | AT2G32660 | disease resistance family protein / LRR family protein |
| A_84_P802615 | 0.0101 | 0.1511 | AT5G62360 | invertase/pectin methylesterase inhibitor family protein |
| A_84_P580265 | 0.0442 | 0.1508 | AT3G07195 | protein coding |
| A_84_P20325 | 0.0019 | 0.1506 | AT3G50060 | protein coding |
| A_84_P555359 | 0.012 | 0.1488 | AT3G02800 | protein coding |
| A_84_P157735 | 0.01 | 0.1485 | AT3G51400 | protein coding |
| A_84_P168223 | 2.00E-04 | 0.1472 | AT1G19770 | protein coding |
| A_84_P20470 | 0.0205 | 0.1464 | AT1G21910 | protein coding |
| A_84_P19000 | 0.0385 | 0.1461 | AT1G70820 | protein coding |
| A_84_P52180 | 0.012 | 0.146 | AT5G60910 | AGL8 (AGAMOUS-LIKE 8); transcription factor |
| A_84_P13875 | 0.0093 | 0.1447 | AT4G32940 | GAMMA-VPE (Vacuolar processing enzyme gamma); cysteine-type endopeptidase |
| A_84_P19490 | 0.0106 | 0.1439 | AT4G19170 | NCED4 (NINE-CIS-EPOXYCAROTENOID DIOXYGENASE 4) |
| A_84_P145309 | 0.0133 | 0.1439 | AT4G13820 | disease resistance family protein / LRR family protein |
| A_84_P186924 | 0.0414 | 0.1433 | AT1G65790 | protein coding |
| A_84_P769325 | 0.0011 | 0.1431 | AT5G11412 | nucleic acid binding / nucleotide binding |
| A_84_P766097 | 0.0055 | 0.1425 | AT5G63770 | ATDGK2 (DIACYLGLYCEROL KINASE 2); diacylglycerol kinase |
| A_84_P15517 | 0.0129 | 0.1423 | AT3G21720 | protein coding |
| A_84_P227469 | 9.00E-04 | 0.1421 | AT3G30350 | protein coding |
| A_84_P21754 | 0.017 | 0.1421 | AT1G23710 | protein coding |
| A_84_P21745 | 0.0327 | 0.1419 | AT1G02400 | protein coding |
| A_84_P13103 | 0.0364 | 0.1411 | AT5G49270 | COBL9/MRH4/SHV2 (COBRA-LIKE 9, SHAVEN 2); carbohydrate binding |
| A_84_P541993 | 0.0103 | 0.1406 | AT2G27310 | F-box family protein |
| A_84_P183184 | 0.0359 | 0.1406 | AT4G26320 | AGP13 (ARABINOGALACTAN PROTEIN 13) |
| A_84_P16606 | 0.0265 | 0.1404 | AT4G01250 | WRKY22 (WRKY DNA-binding protein 22); transcription factor |
| A_84_P22840 | 0.0169 | 0.1399 | AT1G68840 | protein coding |
| A_84_P825292 | 0.0308 | 0.1398 | AT2G14210 | ANR1; DNA binding / transcription factor |
| A_84_P20890 | 0.0135 | 0.1392 | AT1G72920 | protein coding |
| A_84_P19584 | 0.0058 | 0.1384 | AT4G15975 | zinc finger (C3HC4-type RING finger) family protein |
| A_84_P808468 | 0.0035 | 0.1384 | AT5G26310 | UGT72E3; UDP-glycosyltransferase/ coniferyl-alcohol glucosyltransferase/ transferase, transferring g |
| A_84_P245045 | 0.0015 | 0.1375 | AT5G64660 | U-box domain-containing protein |
| A_84_P19511 | 0.0234 | 0.1374 | AT4G24380 | hypothetical protein |
| A_84_P66984 | 0.0478 | 0.1367 | AT1G64360 | protein coding |
| A_84_P12443 | 0.0114 | 0.1365 | AT1G80440 | protein coding |
| A_84_P81409 | 0.0089 | 0.1363 | AT3G15630 | protein coding |
| A_84_P594418 | 2.00E-04 | 0.1363 | AT2G17660 | nitrate-responsive NOI protein, putative |
| A_84_P594026 | 0.0199 | 0.1363 | AT5G35110 | hypothetical protein |
| A_84_P10858 | 1.00E-04 | 0.1362 | AT3G47160 | protein coding |
| A_84_P193844 | 0.0015 | 0.1361 | AT5G42965 | nucleic acid binding / ribonuclease H |
| A_84_P20649 | 0.0018 | 0.1346 | AT5G44350 | ethylene-responsive nuclear protein -related |
| A_84_P750962 | 0.0024 | 0.1345 | AT1G18200 | protein coding |
| A_84_P21661 | 0.001 | 0.1345 | AT5G61590 | AP2 domain-containing transcription factor family protein |
| A_84_P11470 | 0.0053 | 0.1339 | AT1G66400 | protein coding |
| A_84_P54250 | 0.0214 | 0.1337 | AT1G19960 | protein coding |
| A_84_P75644 | 9.00E-04 | 0.1332 | AT2G05440 | glycine-rich protein |
| A_84_P22967 | 0.044 | 0.1331 | AT2G22500 | mitochondrial substrate carrier family protein |
| A_84_P527902 | 0.0026 | 0.1325 | AT3G03870 | protein coding |
| A_84_P234563 | 1.00E-04 | 0.1324 | AT2G25250 | hypothetical protein |
| A_84_P12093 | 0.0296 | 0.132 | AT5G24090 | acidic endochitinase (CHIB1) |
| A_84_P603801 | 4.00E-04 | 0.1318 | AT5G26731 | hypothetical protein |
| A_84_P15199 | 0.0219 | 0.1317 | AT1G59950 | protein coding |
| A_84_P297344 | 0.0464 | 0.1315 | AT5G23240 | DNAJ heat shock N-terminal domain-containing protein |
| A_84_P17192 | 0.0013 | 0.1314 | AT1G22130 | protein coding |
| A_84_P14822 | 0.0244 | 0.1313 | AT4G33450 | AtMYB69 (myb domain protein 69); DNA binding / transcription factor |
| A_84_P18479 | 0.0113 | 0.13 | AT3G60550 | protein coding |
| A_84_P21996 | 0.0248 | 0.1293 | AT2G26390 | serpin, putative / serine protease inhibitor, putative |
| A_84_P806915 | 0.0025 | 0.1292 | AT5G42200 | zinc finger (C3HC4-type RING finger) family protein |
| A_84_P13733 | 0.0329 | 0.1266 | AT3G55980 | protein coding |
| A_84_P75664 | 0.0138 | 0.1259 | AT2G36220 | hypothetical protein |
| A_84_P763492 | 0.0268 | 0.1251 | AT4G12510 | protease inhibitor/seed storage/lipid transfer protein (LTP) family protein |
| A_84_P502958 | 4.00E-04 | 0.123 | AT2G21500 | protein binding / zinc ion binding |
| A_84_P11932 | 0.0033 | 0.1225 | AT1G29160 | protein coding |
| A_84_P772475 | 0.0399 | 0.1222 | AT1G66760 | protein coding |
| A_84_P10533 | 0.011 | 0.1222 | AT1G75380 | protein coding |
| A_84_P19539 | 0.0099 | 0.1222 | AT4G30380 | EXLB2 (EXPANSIN-LIKE B2 PRECURSOR) |
| A_84_P10555 | 0.0487 | 0.1205 | AT1G53540 | protein coding |
| A_84_P13235 | 0.0272 | 0.1204 | AT1G13260 | protein coding |
| A_84_P849144 | 0.0327 | 0.1201 | AT5G41750 | disease resistance protein (TIR-NBS-LRR class), putative |
| A_84_P20314 | 0.0206 | 0.1192 | AT3G47350 | protein coding |
| A_84_P20293 | 0.0235 | 0.1176 | AT3G12500 | protein coding |
| A_84_P730385 | 0.0293 | 0.1175 | AT1G08115 | ncRNA |
| A_84_P106016 | 0.0352 | 0.1174 | AT1G15125 | protein coding |
| A_84_P11191 | 0.0046 | 0.1172 | AT5G43620 | S-locus protein-related |
| A_84_P95119 | 0.014 | 0.117 | AT3G14840 | protein coding |
| A_84_P581876 | 0.0153 | 0.1168 | AT1G76955 | protein coding |
| A_84_P580314 | 0.0279 | 0.1165 | AT3G22235 | protein coding |
| A_84_P607949 | 0.0024 | 0.1164 | AT3G28620 | protein coding |
| A_84_P18604 | 0.0045 | 0.1159 | AT4G33020 | ZIP9 (ZINC TRANSPORTER 9 PRECURSOR); cation transmembrane transporter |
| A_84_P15741 | 0.0023 | 0.1156 | AT4G27280 | calcium-binding EF hand family protein |
| A_84_P233559 | 0.021 | 0.1148 | AT1G10340 | protein coding |
| A_84_P16276 | 0.0275 | 0.1148 | AT1G63530 | protein coding |
| A_84_P740197 | 2.00E-04 | 0.1136 | AT1G69572 | misc_RNA |
| A_84_P605805 | 0.0229 | 0.1134 | AT1G21326 | protein coding |
| A_84_P20714 | 0.0026 | 0.1129 | AT5G61440 | thioredoxin family protein |
| A_84_P23626 | 0.0328 | 0.1129 | AT1G71450 | protein coding |
| A_84_P216248 | 9.00E-04 | 0.1123 | AT1G69890 | protein coding |
| A_84_P293424 | 0.0084 | 0.1119 | AT2G05510 | glycine-rich protein |
| A_84_P20938 | 0.0449 | 0.1109 | AT1G69880 | protein coding |
| A_84_P22149 | 2.00E-04 | 0.1109 | AT3G13310 | protein coding |
| A_84_P16749 | 0 | 0.1105 | AT4G16780 | ATHB-2 (ARABIDOPSIS THALIANA HOMEOBOX PROTEIN 2); DNA binding / transcription factor |
| A_84_P128141 | 0.0354 | 0.1101 | AT5G39890 | hypothetical protein |
| A_84_P16551 | 0.0234 | 0.1074 | AT3G52400 | protein coding |
| A_84_P16626 | 0.0331 | 0.1064 | AT4G08770 | peroxidase, putative |
| A_84_P814710 | 0.0129 | 0.1058 | AT1G14870 | protein coding |
| A_84_P11143 | 0.0429 | 0.1056 | AT5G22570 | WRKY38 (WRKY DNA-binding protein 38); transcription factor |
| A_84_P553867 | 0.0331 | 0.1054 | AT4G37720 | ATPSK6 (PHYTOSULFOKINE 6 PRECURSOR); growth factor |
| A_84_P556737 | 0.0275 | 0.1051 | AT5G65207 | hypothetical protein |
| A_84_P19215 | 0.0425 | 0.1043 | AT2G21510 | DNAJ heat shock N-terminal domain-containing protein |
| A_84_P602371 | 0.0076 | 0.1032 | AT1G73510 | protein coding |
| A_84_P586578 | 0.0499 | 0.1029 | AT3G22231 | protein coding |
| A_84_P13332 | 0.0064 | 0.1028 | AT1G22570 | protein coding |
| A_84_P168853 | 0.0314 | 0.1028 | AT3G02550 | protein coding |
| A_84_P15742 | 0.0332 | 0.1028 | AT4G27450 | hypothetical protein |
| A_84_P577514 | 0.0385 | 0.1028 | AT2G31210 | basic helix-loop-helix (bHLH) family protein |
| A_84_P206968 | 0.0038 | 0.1026 | AT1G57990 | protein coding |
| A_84_P279900 | 0.0255 | 0.102 | AT5G39180 | germin-like protein, putative |
| A_84_P137619 | 0.0299 | 0.1019 | AT5G54490 | PBP1 (PINOID-BINDING PROTEIN 1); calcium ion binding |
| A_84_P19944 | 5.00E-04 | 0.1012 | AT1G72910 | protein coding |
| A_84_P16173 | 0.0379 | 0.1012 | AT1G28480 | protein coding |
| A_84_P22597 | 0.0071 | 0.1006 | AT5G59550 | zinc finger (C3HC4-type RING finger) family protein |
| A_84_P132345 | 0.002 | 0.1005 | AT4G32340 | hypothetical protein |
| A_84_P602094 | 0.0013 | 0.1005 | AT5G54720 | ankyrin repeat family protein |
| A_84_P23274 | 0.0014 | 0.0992 | AT1G29290 | protein coding |
| A_84_P171073 | 0.0261 | 0.0992 | AT1G35210 | protein coding |
| A_84_P20535 | 0.0033 | 0.0991 | AT4G17500 | ATERF-1 (ETHYLENE RESPONSIVE ELEMENT BINDING FACTOR 1); DNA binding / transcription activator/ trans |
| A_84_P212828 | 1.00E-04 | 0.0986 | AT4G30280 | ATXTH18/XTH18 (XYLOGLUCAN ENDOTRANSGLUCOSYLASE/HYDROLASE 18); hydrolase, acting on glycosyl bonds |
| A_84_P306860 | 0.036 | 0.0979 | AT5G10695 | hypothetical protein |
| A_84_P23096 | 0.0228 | 0.0972 | AT3G26200 | protein coding |
| A_84_P576707 | 0.0037 | 0.0969 | AT3G09020 | protein coding |
| A_84_P19790 | 0.0084 | 0.0968 | AT5G66690 | UGT72E2; UDP-glycosyltransferase/ coniferyl-alcohol glucosyltransferase/ transferase, transferring g |
| A_84_P223559 | 0.0189 | 0.0968 | AT2G14560 | hypothetical protein |
| A_84_P17859 | 0.0018 | 0.0963 | AT5G57560 | TCH4 (TOUCH 4); hydrolase, acting on glycosyl bonds / xyloglucan:xyloglucosyl transferase |
| A_84_P19389 | 0.0015 | 0.0962 | AT3G52450 | protein coding |
| A_84_P239215 | 0.0349 | 0.095 | AT2G26010 | PDF1.3 (plant defensin 1.3) |
| A_84_P13801 | 0.036 | 0.0936 | AT4G11650 | ATOSM34 (OSMOTIN 34) |
| A_84_P605309 | 0.0349 | 0.0935 | AT3G43930 | protein coding |
| A_84_P23014 | 0.043 | 0.0935 | AT2G44581 | protein binding / zinc ion binding |
| A_84_P15329 | 0.0157 | 0.0935 | AT2G40000 | hypothetical protein |
| A_84_P16258 | 4.00E-04 | 0.0916 | AT1G49230 | protein coding |
| A_84_P571270 | 0.002 | 0.0915 | AT2G28056 | misc_RNA |
| A_84_P13113 | 0.018 | 0.0911 | AT5G52050 | MATE efflux protein-related |
| A_84_P146938 | 0.0486 | 0.0908 | AT1G02450 | protein coding |
| A_84_P23963 | 0.0148 | 0.0904 | AT2G30040 | MAPKKK14 (Mitogen-activated protein kinase kinase kinase 14); kinase |
| A_84_P23919 | 0.025 | 0.0892 | AT2G35980 | YLS9 (YELLOW-LEAF-SPECIFIC GENE 9) |
| A_84_P229729 | 0.014 | 0.0892 | AT2G20142 | transmembrane receptor |
| A_84_P753024 | 0.0269 | 0.0884 | AT1G67265 | protein coding |
| A_84_P23347 | 0.0114 | 0.0883 | AT4G36010 | pathogenesis-related thaumatin family protein |
| A_84_P217688 | 0.022 | 0.088 | AT1G76600 | protein coding |
| A_84_P517162 | 0.0082 | 0.0871 | AT2G20630 | protein phosphatase 2C, putative / PP2C, putative |
| A_84_P54170 | 2.00E-04 | 0.0863 | AT3G57450 | protein coding |
| A_84_P584450 | 0.0074 | 0.0857 | AT1G27890 | protein coding |
| A_84_P14013 | 0.0244 | 0.0852 | AT5G40000 | AAA-type ATPase family protein |
| A_84_P799760 | 0.0024 | 0.0843 |  |  |
| A_84_P768849 | 0.0074 | 0.0832 | AT5G09978 | PEP7 (ELICITOR PEPTIDE 7 PRECURSOR) |
| A_84_P537220 | 0.0382 | 0.0832 | AT5G57510 | hypothetical protein |
| A_84_P218328 | 1.00E-04 | 0.0827 | AT3G23170 | protein coding |
| A_84_P15156 | 0.0014 | 0.0825 | AT1G61470 | protein coding |
| A_84_P611334 | 0.0277 | 0.0821 | AT2G22880 | VQ motif-containing protein |
| A_84_P301260 | 0.0113 | 0.0816 | AT1G74450 | protein coding |
| A_84_P599034 | 0.0043 | 0.0793 | AT3G16860 | protein coding |
| A_84_P161293 | 0.0375 | 0.0787 | AT5G39110 | germin-like protein, putative |
| A_84_P18158 | 0.0131 | 0.0773 | AT1G57850 | protein coding |
| A_84_P764908 | 0.0235 | 0.077 | AT4G35655 | hypothetical protein |
| A_84_P19207 | 0.0446 | 0.0767 | AT2G40340 | AP2 domain-containing transcription factor, putative (DRE2B) |
| A_84_P21874 | 0.0466 | 0.0761 | AT1G27730 | protein coding |
| A_84_P22782 | 0.0021 | 0.076 | AT1G72950 | protein coding |
| A_84_P65864 | 2.00E-04 | 0.076 | AT1G18740 | protein coding |
| A_84_P17582 | 0.0161 | 0.076 | AT4G11280 | ACS6 (1-AMINOCYCLOPROPANE-1-CARBOXYLIC ACID (ACC) SYNTHASE 6) |
| A_84_P18813 | 0.0169 | 0.0756 | AT5G59490 | haloacid dehalogenase-like hydrolase family protein |
| A_84_P19616 | 0.0394 | 0.0755 | AT5G07200 | YAP169 (GIBBERELLIN 20 OXIDASE 3); gibberellin 20-oxidase |
| A_84_P558681 | 0.0021 | 0.0752 | AT1G53903 | protein coding |
| A_84_P242183 | 0.0067 | 0.0749 | AT1G70390 | protein coding |
| A_84_P522306 | 0.0013 | 0.0748 | AT1G27820 | protein coding |
| A_84_P576622 | 0.009 | 0.0745 | AT2G32200 | hypothetical protein |
| A_84_P15734 | 0.003 | 0.0742 | AT4G25810 | XTR6 (XYLOGLUCAN ENDOTRANSGLYCOSYLASE 6); hydrolase, acting on glycosyl bonds |
| A_84_P311633 | 0.0041 | 0.0727 | AT5G11070 | hypothetical protein |
| A_84_P268090 | 0.0138 | 0.0724 | AT2G01300 | hypothetical protein |
| A_84_P196694 | 0.0285 | 0.0713 | AT5G44430 | PDF1.2c (plant defensin 1.2c) |
| A_84_P12994 | 0.0128 | 0.0709 | AT5G06730 | peroxidase, putative |
| A_84_P242895 | 0.0043 | 0.0705 | AT1G66160 | protein coding |
| A_84_P535878 | 0.0091 | 0.0701 | AT3G03280 | protein coding |
| A_84_P225679 | 0.0307 | 0.0696 | AT5G39120 | germin-like protein, putative |
| A_84_P14505 | 0.0291 | 0.0695 | AT2G32140 | transmembrane receptor |
| A_84_P17650 | 0.0079 | 0.0695 | AT4G31020 | hypothetical protein |
| A_84_P179084 | 0.0011 | 0.068 | AT5G35735 | auxin-responsive family protein |
| A_84_P209328 | 0.0016 | 0.0674 | AT1G20630 | protein coding |
| A_84_P769028 | 0.0021 | 0.0673 | AT5G44065 | hypothetical protein |
| A_84_P10846 | 0.0095 | 0.0672 | AT3G44350 | protein coding |
| A_84_P22467 | 0.0072 | 0.067 | AT5G11140 | hypothetical protein |
| A_84_P12143 | 0.0204 | 0.065 | AT5G45340 | CYP707A3 (cytochrome P450, family 707, subfamily A, polypeptide 3); oxygen binding |
| A_84_P67084 | 0.0047 | 0.0649 | AT1G25400 | protein coding |
| A_84_P16923 | 0.0278 | 0.0633 | AT5G59820 | RHL41 (RESPONSIVE TO HIGH LIGHT 41); nucleic acid binding / transcription factor/ zinc ion binding |
| A_84_P596516 | 0.0043 | 0.0632 | AT4G14450 | hypothetical protein |
| A_84_P14985 | 0.0116 | 0.0632 | AT5G47220 | ATERF-2/ATERF2/ERF2 (ETHYLENE RESPONSE FACTOR 2); DNA binding / transcription activator/ transcripti |
| A_84_P22140 | 0.0018 | 0.063 | AT3G23250 | protein coding |
| A_84_P16621 | 0.0259 | 0.0628 | AT4G05170 | DNA binding / transcription factor |
| A_84_P561097 | 0.0081 | 0.0625 | AT5G46295 | hypothetical protein |
| A_84_P175491 | 0.0394 | 0.0613 | AT5G62520 | SRO5 (SIMILAR TO RCD ONE 5); NAD+ ADP-ribosyltransferase |
| A_84_P168493 | 0.002 | 0.0604 | AT3G46620 | protein coding |
| A_84_P23603 | 6.00E-04 | 0.0599 | AT1G34060 | protein coding |
| A_84_P500133 | 0.0149 | 0.0596 | AT1G42980 | protein coding |
| A_84_P296724 | 0.0132 | 0.0571 | AT5G51190 | AP2 domain-containing transcription factor, putative |
| A_84_P22607 | 0.0082 | 0.057 | AT5G61600 | ethylene-responsive element-binding family protein |
| A_84_P219668 | 0.0078 | 0.0562 | AT3G04640 | protein coding |
| A_84_P140849 | 0.0167 | 0.0548 | AT1G32920 | protein coding |
| A_84_P141659 | 0.0311 | 0.0537 | AT4G01360 | hypothetical protein |
| A_84_P17055 | 1.00E-04 | 0.0536 | AT1G65480 | protein coding |
| A_84_P17343 | 0.0286 | 0.0527 | AT2G44840 | ATERF13/EREBP (ETHYLENE-RESPONSIVE ELEMENT BINDING FACTOR 13); DNA binding / transcription factor |
| A_84_P852303 | 0.0297 | 0.0517 | AT4G37710 | VQ motif-containing protein |
| A_84_P769115 | 0.0167 | 0.0516 | AT5G42053 | hypothetical protein |
| A_84_P12324 | 0.0047 | 0.0513 | AT1G70130 | protein coding |
| A_84_P863296 | 0.0026 | 0.0506 | AT5G62530 | ALDH12A1 (Aldehyde dehydrogenase 12A1); 1-pyrroline-5-carboxylate dehydrogenase/ 3-chloroallyl aldeh |
| A_84_P539348 | 0.0034 | 0.0502 | AT2G25735 | hypothetical protein |
| A_84_P50100 | 4.00E-04 | 0.0501 | AT3G55240 | protein coding |
| A_84_P19559 | 0.0346 | 0.0499 | AT1G15010 | protein coding |
| A_84_P185784 | 0.001 | 0.0498 | AT2G39920 | acid phosphatase class B family protein |
| A_84_P19362 | 0.0221 | 0.049 | AT3G46090 | protein coding |
| A_84_P310613 | 0.0062 | 0.0489 | AT2G26020 | PDF1.2b (plant defensin 1.2b) |
| A_84_P824268 | 0.0092 | 0.0477 | AT1G56660 | protein coding |
| A_84_P55550 | 0.0088 | 0.0477 | AT4G32280 | IAA29 (indoleacetic acid-induced protein 29); transcription factor |
| A_84_P537328 | 0.0063 | 0.0458 | AT1G12805 | protein coding |
| A_84_P158475 | 6.00E-04 | 0.0457 | AT2G41640 | hypothetical protein |
| A_84_P16729 | 4.00E-04 | 0.0456 | AT4G37770 | ACS8 (1-Amino-cyclopropane-1-carboxylate synthase 8) |
| A_84_P140239 | 0.0427 | 0.0454 | AT5G02580 | hypothetical protein |
| A_84_P21035 | 0.0057 | 0.0454 | AT2G47810 | histone-like transcription factor (CBF/NF-Y) family protein |
| A_84_P598642 | 0.0495 | 0.0448 | AT1G07135 | protein coding |
| A_84_P15931 | 0.0026 | 0.0448 | AT5G47230 | ERF5 (ETHYLENE RESPONSIVE ELEMENT BINDING FACTOR 5); DNA binding / transcription activator/ transcri |
| A_84_P102986 | 3.00E-04 | 0.0436 | AT2G27080 | harpin-induced protein-related / HIN1-related / harpin-responsive protein-related |
| A_84_P17470 | 0.0041 | 0.0428 | AT3G46070 | protein coding |
| A_84_P11903 | 7.00E-04 | 0.0419 | AT1G06160 | protein coding |
| A_84_P21946 | 0 | 0.0416 | AT1G73540 | protein coding |
| A_84_P764543 | 0.001 | 0.0411 | AT4G39403 | PLS (POLARIS) |
| A_84_P21931 | 0.023 | 0.0402 | AT1G28370 | protein coding |
| A_84_P23516 | 0.0309 | 0.0394 | AT5G51990 | CBF4/DREB1D (C- REPEAT-BINDING FACTOR 4); DNA binding / transcription activator/ transcription facto |
| A_84_P23501 | 0.0305 | 0.0392 | AT5G47850 | protein kinase, putative |
| A_84_P138279 | 0.0252 | 0.0382 | AT1G58420 | protein coding |
| A_84_P768411 | 0.0018 | 0.0377 | AT5G16023 | DVL1/RTFL18 (DEVIL1) |
| A_84_P544251 | 0.0033 | 0.0359 | AT5G38700 | hypothetical protein |
| A_84_P20998 | 0.0015 | 0.0357 | AT1G35140 | protein coding |
| A_84_P89769 | 4.00E-04 | 0.0351 | AT2G35930 | U-box domain-containing protein |
| A_84_P97916 | 0.0283 | 0.0339 | AT4G29780 | hypothetical protein |
| A_84_P755281 | 0.0032 | 0.0336 | AT3G27140 | protein coding |
| A_84_P610481 | 0.0035 | 0.032 | AT2G34600 | JAZ7/TIFY5B (JASMONATE-ZIM-DOMAIN PROTEIN 7) |
| A_84_P10724 | 0.001 | 0.0319 | AT2G30020 | protein phosphatase 2C, putative / PP2C, putative |
| A_84_P166733 | 0.0013 | 0.0316 | AT5G17350 | hypothetical protein |
| A_84_P22489 | 8.00E-04 | 0.0315 | AT5G22250 | CCR4-NOT transcription complex protein, putative |
| A_84_P579612 | 2.00E-04 | 0.0309 | AT4G27652 | hypothetical protein |
| A_84_P260800 | 0.0321 | 0.0297 | AT4G19430 | hypothetical protein |
| A_84_P183724 | 0.0042 | 0.0286 | AT2G35290 | hypothetical protein |
| A_84_P715787 | 3.00E-04 | 0.027 | AT1G32928 | protein coding |
| A_84_P19516 | 0.0402 | 0.0269 | AT4G25470 | CBF2 (FREEZING TOLERANCE QTL 4); DNA binding / transcription activator/ transcription factor |
| A_84_P512655 | 0.0066 | 0.0261 | AT1G77640 | protein coding |
| A_84_P137009 | 0.0077 | 0.0247 | AT5G44420 | PDF1.2 (Low-molecular-weight cysteine-rich 77) |
| A_84_P18566 | 0.0132 | 0.0227 | AT4G24570 | mitochondrial substrate carrier family protein |
| A_84_P155845 | 0.0014 | 0.0225 | AT2G47950 | hypothetical protein |
| A_84_P756652 | 0.0012 | 0.0225 | AT2G35710 | glycogenin glucosyltransferase (glycogenin)-related |
| A_84_P14331 | 0.0273 | 0.0222 | AT1G76650 | protein coding |
| A_84_P767643 | 2.00E-04 | 0.0212 | AT1G29620 | protein coding |
| A_84_P573393 | 0.0031 | 0.0205 | AT4G27654 | hypothetical protein |
| A_84_P17463 | 4.00E-04 | 0.0173 | AT3G44260 | protein coding |
| A_84_P164303 | 8.00E-04 | 0.0166 | AT5G58680 | armadillo/beta-catenin repeat family protein |
| A_84_P11223 | 0.0021 | 0.0164 | AT5G52020 | AP2 domain-containing protein |
| A_84_P16799 | 1.00E-04 | 0.0155 | AT1G18300 | protein coding |
| A_84_P16247 | 0.017 | 0.0152 | AT1G12610 | protein coding |
| A_84_P595141 | 4.00E-04 | 0.0124 | AT1G30135 | protein coding |
| A_84_P66214 | 0.0232 | 0.0123 | AT3G02840 | protein coding |
| A_84_P156125 | 0.0052 | 0.0118 | AT3G61190 | protein coding |
| A_84_P272600 | 0.0061 | 0.01 | AT5G21960 | AP2 domain-containing transcription factor, putative |
| A_84_P554567 | 1.00E-04 | 0.009 | AT3G28340 | protein coding |
| A_84_P198484 | 0.0012 | 0.008 | AT3G10930 | protein coding |
| A_84_P709533 | 0.002 | 0.0072 | AT4G13395 | DVL10/RTFL12 (ROTUNDIFOLIA LIKE 12) |
| A_84_P19028 | 0.0035 | 0.0066 | AT1G74930 | protein coding |
| A_84_P712672 | 6.00E-04 | 0.0059 | AT3G15210 | protein coding |
| A_84_P11046 | 0.0255 | 0.0057 | AT4G34410 | AP2 domain-containing transcription factor, putative |
| A_84_P17145 | 0.0014 | 0.0053 | AT1G63030 | protein coding |
| A_84_P18908 | 0 | 0.0046 | AT1G33760 | protein coding |
| A_84_P20976 | 3.00E-04 | 0.0016 | AT1G19210 | protein coding |
