# Supplementary material for: The bZIP protein from Tamarix hispida, ThbZIP1, is ACGT elements binding factor that enhances abiotic stress signaling in transgenic Arabidopsis
Source: BMC Plant Biol. 2013 Oct 4;13:151. doi: 10.1186/1471-2229-13-151 (PMC3852707; doi:10.1186/1471-2229-13-151)
Supplement: Additional file 5: Figure S3 — Comparison of the results of microarray and real-time RT-PCR. The significantly differentially regulated genes detected by microarray were randomly selected for real-time RT-PCR analysis. Correlation analysis of the results between real-time RT-PCR and cDNA microarray were calculated (P < 0.05). [file 1471-2229-13-151-S5.doc]

**Additional file 5: Figure S3**


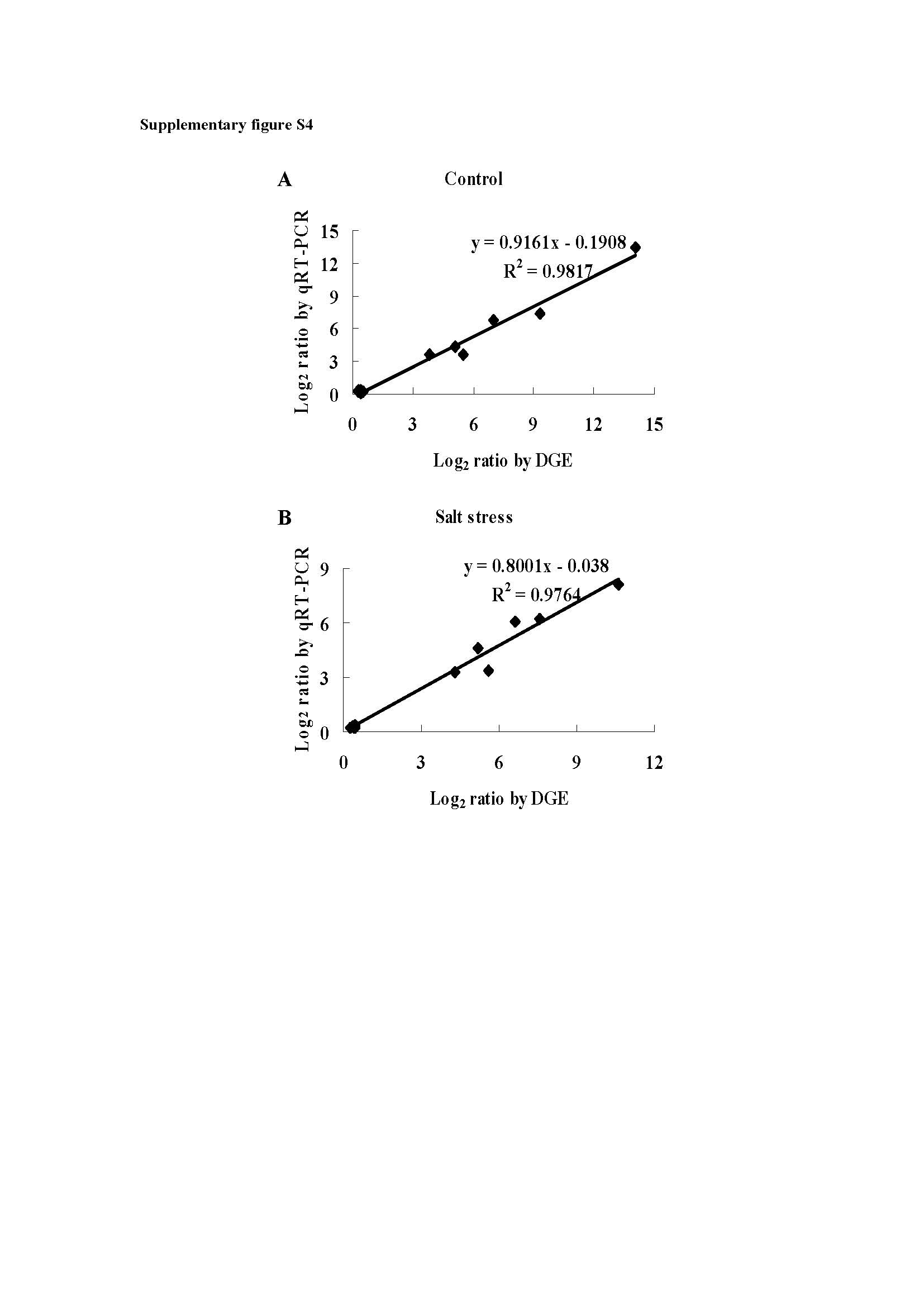


**Fig. S3** Comparison of the results of microarray and real-time RT-PCR. The significantly differentially regulated genes detected by microarray were randomly selected for real-time RT-PCR analysis. Correlation analysis of the results between real time RT-PCR and cDNA microarray were calculated (P < 0.05).
