# Supplementary material for: The bZIP protein from Tamarix hispida, ThbZIP1, is ACGT elements binding factor that enhances abiotic stress signaling in transgenic Arabidopsis
Source: BMC Plant Biol. 2013 Oct 4;13:151. doi: 10.1186/1471-2229-13-151 (PMC3852707; doi:10.1186/1471-2229-13-151)
Supplement: Additional file 6: Table S3 — Go analysis of the genes in response to NaCl treatment. [file 1471-2229-13-151-S6.doc]

**Additional file 6: Table S3** [**Go analysis of the gene**](http://sas.ebioservice.com/bioinfoplug/molnet/temp/BH11163/74154069/gotree.htm)**s in response to NaCl treatment.**

| **GOId** | **Name** | [**Hits**](javascript:void(0);) | [**Total**](javascript:void(0);) | | [**Percent**](javascript:void(0);) | | | [**Enrichment test pvalue**](javascript:void(0);) | | | | [**qvalue**](javascript:void(0);) |
| --- | --- | --- | --- | --- | --- | --- | --- | --- | --- | --- | --- | --- |
| The genes up-regulated in response to NaCl stress. | | | | | | | | | | | | |
| GO:0003674 | molecular_function | [885](http://sas.ebioservice.com/bioinfoplug_molnetgotree.showgene.do?acc=GO:0003674&name=molecular_function&recordid=74154067&loginid=BH11163) | 29448 | | | 3.01% | | | 0.9925 | | 0.4178 | |
| GO:0003824 | catalytic activity | [354](http://sas.ebioservice.com/bioinfoplug_molnetgotree.showgene.do?acc=GO:0003824&name=catalytic+activity&recordid=74154067&loginid=BH11163) | 8137 | | | 4.35% | | | 0.0 | | 0.0 | |
| GO:0009975 | cyclase activity | [2](http://sas.ebioservice.com/bioinfoplug_molnetgotree.showgene.do?acc=GO:0009975&name=cyclase+activity&recordid=74154067&loginid=BH11163) | 15 | | | 13.33% | | | 0.09 | | 0.1057 | |
| GO:0016491 | oxidoreductase activity | [66](http://sas.ebioservice.com/bioinfoplug_molnetgotree.showgene.do?acc=GO:0016491&name=oxidoreductase+activity&recordid=74154067&loginid=BH11163) | 1420 | | | 4.65% | | | 9.0E-4 | | 0.0038 | |
| GO:0016740 | transferase activity | [113](http://sas.ebioservice.com/bioinfoplug_molnetgotree.showgene.do?acc=GO:0016740&name=transferase+activity&recordid=74154067&loginid=BH11163) | 2722 | | | 4.15% | | | 0.0010 | | 0.0039 | |
| GO:0016787 | hydrolase activity | [122](http://sas.ebioservice.com/bioinfoplug_molnetgotree.showgene.do?acc=GO:0016787&name=hydrolase+activity&recordid=74154067&loginid=BH11163) | 2933 | | | 4.16% | | | 6.0E-4 | | 0.0026 | |
| GO:0016829 | lyase activity | [19](http://sas.ebioservice.com/bioinfoplug_molnetgotree.showgene.do?acc=GO:0016829&name=lyase+activity&recordid=74154067&loginid=BH11163) | 333 | | | 5.71% | | | 0.01 | | 0.025 | |
| GO:0016853 | isomerase activity | [15](http://sas.ebioservice.com/bioinfoplug_molnetgotree.showgene.do?acc=GO:0016853&name=isomerase+activity&recordid=74154067&loginid=BH11163) | 220 | | | 6.82% | | | 0.0049 | | 0.0157 | |
| GO:0016874 | ligase activity | [14](http://sas.ebioservice.com/bioinfoplug_molnetgotree.showgene.do?acc=GO:0016874&name=ligase+activity&recordid=74154067&loginid=BH11163) | 453 | | | 3.09% | | | 0.5306 | | 0.2883 | |
| GO:0005198 | structural molecule activity | [8](http://sas.ebioservice.com/bioinfoplug_molnetgotree.showgene.do?acc=GO:0005198&name=structural+molecule+activity&recordid=74154067&loginid=BH11163) | 501 | | | 1.6% | | | 0.9855 | | 0.4178 | |
| GO:0003735 | structural constituent of ribosome | [7](http://sas.ebioservice.com/bioinfoplug_molnetgotree.showgene.do?acc=GO:0003735&name=structural+constituent+of+ribosome&recordid=74154067&loginid=BH11163) | 361 | | | 1.94% | | | 0.9247 | | 0.404 | |
| GO:0005215 | transporter activity | [47](http://sas.ebioservice.com/bioinfoplug_molnetgotree.showgene.do?acc=GO:0005215&name=transporter+activity&recordid=74154067&loginid=BH11163) | 1222 | | | 3.85% | | | 0.0763 | | 0.0949 | |
| GO:0015238 | drug transporter activity | [5](http://sas.ebioservice.com/bioinfoplug_molnetgotree.showgene.do?acc=GO:0015238&name=drug+transporter+activity&recordid=74154067&loginid=BH11163) | 67 | | | 7.46% | | | 0.0639 | | 0.0837 | |
| GO:0022857 | transmembrane transporter activity | [34](http://sas.ebioservice.com/bioinfoplug_molnetgotree.showgene.do?acc=GO:0022857&name=transmembrane+transporter+activity&recordid=74154067&loginid=BH11163) | 930 | | | 3.66% | | | 0.1797 | | 0.157 | |
| GO:0022892 | substrate-specific transporter activity | [22](http://sas.ebioservice.com/bioinfoplug_molnetgotree.showgene.do?acc=GO:0022892&name=substrate-specific+transporter+activity&recordid=74154067&loginid=BH11163) | 865 | | | 2.54% | | | 0.8398 | | 0.3794 | |
| GO:0051184 | cofactor transporter activity | [1](http://sas.ebioservice.com/bioinfoplug_molnetgotree.showgene.do?acc=GO:0051184&name=cofactor+transporter+activity&recordid=74154067&loginid=BH11163) | 10 | | | 10.0% | | | 0.2834 | | 0.2038 | |
| GO:0005488 | binding | [349](http://sas.ebioservice.com/bioinfoplug_molnetgotree.showgene.do?acc=GO:0005488&name=binding&recordid=74154067&loginid=BH11163) | 10009 | | | 3.49% | | | 0.0031 | | 0.0111 | |
| GO:0000166 | nucleotide binding | [92](http://sas.ebioservice.com/bioinfoplug_molnetgotree.showgene.do?acc=GO:0000166&name=nucleotide+binding&recordid=74154067&loginid=BH11163) | 2177 | | | 4.23% | | | 0.0017 | | 0.0066 | |
| GO:0003676 | nucleic acid binding | [125](http://sas.ebioservice.com/bioinfoplug_molnetgotree.showgene.do?acc=GO:0003676&name=nucleic+acid+binding&recordid=74154067&loginid=BH11163) | 3939 | | | 3.17% | | | 0.3703 | | 0.2373 | |
| GO:0003682 | chromatin binding | [2](http://sas.ebioservice.com/bioinfoplug_molnetgotree.showgene.do?acc=GO:0003682&name=chromatin+binding&recordid=74154067&loginid=BH11163) | 38 | | | 5.26% | | | 0.3361 | | 0.2207 | |
| GO:0005515 | protein binding | [107](http://sas.ebioservice.com/bioinfoplug_molnetgotree.showgene.do?acc=GO:0005515&name=protein+binding&recordid=74154067&loginid=BH11163) | 2640 | | | 4.05% | | | 0.0027 | | 0.01 | |
| GO:0008144 | drug binding | [2](http://sas.ebioservice.com/bioinfoplug_molnetgotree.showgene.do?acc=GO:0008144&name=drug+binding&recordid=74154067&loginid=BH11163) | 24 | | | 8.33% | | | 0.1812 | | 0.157 | |
| GO:0008289 | lipid binding | [5](http://sas.ebioservice.com/bioinfoplug_molnetgotree.showgene.do?acc=GO:0008289&name=lipid+binding&recordid=74154067&loginid=BH11163) | 267 | | | 1.87% | | | 0.911 | | 0.3996 | |
| GO:0019825 | oxygen binding | [10](http://sas.ebioservice.com/bioinfoplug_molnetgotree.showgene.do?acc=GO:0019825&name=oxygen+binding&recordid=74154067&loginid=BH11163) | 231 | | | 4.33% | | | 0.1856 | | 0.1571 | |
| GO:0030246 | carbohydrate binding | [3](http://sas.ebioservice.com/bioinfoplug_molnetgotree.showgene.do?acc=GO:0030246&name=carbohydrate+binding&recordid=74154067&loginid=BH11163) | 153 | | | 1.96% | | | 0.8478 | | 0.3798 | |
| GO:0042277 | peptide binding | [3](http://sas.ebioservice.com/bioinfoplug_molnetgotree.showgene.do?acc=GO:0042277&name=peptide+binding&recordid=74154067&loginid=BH11163) | 28 | | | 10.71% | | | 0.0641 | | 0.0837 | |
| GO:0043167 | ion binding | [74](http://sas.ebioservice.com/bioinfoplug_molnetgotree.showgene.do?acc=GO:0043167&name=ion+binding&recordid=74154067&loginid=BH11163) | 2255 | | | 3.28% | | | 0.3015 | | 0.2089 | |
| GO:0043176 | amine binding | [3](http://sas.ebioservice.com/bioinfoplug_molnetgotree.showgene.do?acc=GO:0043176&name=amine+binding&recordid=74154067&loginid=BH11163) | 23 | | | 13.04% | | | 0.0413 | | 0.0631 | |
| GO:0046906 | tetrapyrrole binding | [12](http://sas.ebioservice.com/bioinfoplug_molnetgotree.showgene.do?acc=GO:0046906&name=tetrapyrrole+binding&recordid=74154067&loginid=BH11163) | 320 | | | 3.75% | | | 0.2899 | | 0.2045 | |
| GO:0048037 | cofactor binding | [6](http://sas.ebioservice.com/bioinfoplug_molnetgotree.showgene.do?acc=GO:0048037&name=cofactor+binding&recordid=74154067&loginid=BH11163) | 264 | | | 2.27% | | | 0.819 | | 0.3764 | |
| GO:0051540 | metal cluster binding | [2](http://sas.ebioservice.com/bioinfoplug_molnetgotree.showgene.do?acc=GO:0051540&name=metal+cluster+binding&recordid=74154067&loginid=BH11163) | 40 | | | 5.0% | | | 0.3579 | | 0.2335 | |
| GO:0009055 | electron carrier activity | [21](http://sas.ebioservice.com/bioinfoplug_molnetgotree.showgene.do?acc=GO:0009055&name=electron+carrier+activity&recordid=74154067&loginid=BH11163) | 495 | | | 4.24% | | | 0.0955 | | 0.111 | |
| GO:0016209 | antioxidant activity | [4](http://sas.ebioservice.com/bioinfoplug_molnetgotree.showgene.do?acc=GO:0016209&name=antioxidant+activity&recordid=74154067&loginid=BH11163) | 126 | | | 3.17% | | | 0.5454 | | 0.2883 | |
| GO:0004601 | peroxidase activity | [4](http://sas.ebioservice.com/bioinfoplug_molnetgotree.showgene.do?acc=GO:0004601&name=peroxidase+activity&recordid=74154067&loginid=BH11163) | 109 | | | 3.67% | | | 0.4367 | | 0.264 | |
| GO:0030234 | enzyme regulator activity | [9](http://sas.ebioservice.com/bioinfoplug_molnetgotree.showgene.do?acc=GO:0030234&name=enzyme+regulator+activity&recordid=74154067&loginid=BH11163) | 342 | | | 2.63% | | | 0.7229 | | 0.3442 | |
| GO:0004857 | enzyme inhibitor activity | [6](http://sas.ebioservice.com/bioinfoplug_molnetgotree.showgene.do?acc=GO:0004857&name=enzyme+inhibitor+activity&recordid=74154067&loginid=BH11163) | 174 | | | 3.45% | | | 0.4498 | | 0.2641 | |
| GO:0008047 | enzyme activator activity | [1](http://sas.ebioservice.com/bioinfoplug_molnetgotree.showgene.do?acc=GO:0008047&name=enzyme+activator+activity&recordid=74154067&loginid=BH11163) | 74 | | | 1.35% | | | 0.8971 | | 0.3968 | |
| GO:0019208 | phosphatase regulator activity | [1](http://sas.ebioservice.com/bioinfoplug_molnetgotree.showgene.do?acc=GO:0019208&name=phosphatase+regulator+activity&recordid=74154067&loginid=BH11163) | 18 | | | 5.56% | | | 0.4377 | | 0.264 | |
| GO:0060589 | nucleoside-triphosphatase regulator activity | [2](http://sas.ebioservice.com/bioinfoplug_molnetgotree.showgene.do?acc=GO:0060589&name=nucleoside-triphosphatase+regulator+activity&recordid=74154067&loginid=BH11163) | 90 | | | 2.22% | | | 0.7644 | | 0.3544 | |
| GO:0030528 | transcription regulator activity | [69](http://sas.ebioservice.com/bioinfoplug_molnetgotree.showgene.do?acc=GO:0030528&name=transcription+regulator+activity&recordid=74154067&loginid=BH11163) | 1885 | | | 3.66% | | | 0.0833 | | 0.1016 | |
| GO:0000156 | two-component response regulator activity | [3](http://sas.ebioservice.com/bioinfoplug_molnetgotree.showgene.do?acc=GO:0000156&name=two-component+response+regulator+activity&recordid=74154067&loginid=BH11163) | 35 | | | 8.57% | | | 0.1036 | | 0.1171 | |
| GO:0003700 | transcription factor activity | [59](http://sas.ebioservice.com/bioinfoplug_molnetgotree.showgene.do?acc=GO:0003700&name=transcription+factor+activity&recordid=74154067&loginid=BH11163) | 1677 | | | 3.52% | | | 0.165 | | 0.1477 | |
| GO:0003711 | transcription elongation regulator activity | [1](http://sas.ebioservice.com/bioinfoplug_molnetgotree.showgene.do?acc=GO:0003711&name=transcription+elongation+regulator+activity&recordid=74154067&loginid=BH11163) | 9 | | | 11.11% | | | 0.2613 | | 0.1971 | |
| GO:0003712 | transcription cofactor activity | [1](http://sas.ebioservice.com/bioinfoplug_molnetgotree.showgene.do?acc=GO:0003712&name=transcription+cofactor+activity&recordid=74154067&loginid=BH11163) | 28 | | | 3.57% | | | 0.5847 | | 0.3012 | |
| GO:0003715 | transcription termination factor activity | [1](http://sas.ebioservice.com/bioinfoplug_molnetgotree.showgene.do?acc=GO:0003715&name=transcription+termination+factor+activity&recordid=74154067&loginid=BH11163) | 2 | | | 50.0% | | | 0.0869 | | 0.1043 | |
| GO:0016563 | transcription activator activity | [4](http://sas.ebioservice.com/bioinfoplug_molnetgotree.showgene.do?acc=GO:0016563&name=transcription+activator+activity&recordid=74154067&loginid=BH11163) | 134 | | | 2.99% | | | 0.5927 | | 0.3012 | |
| GO:0016564 | transcription repressor activity | [3](http://sas.ebioservice.com/bioinfoplug_molnetgotree.showgene.do?acc=GO:0016564&name=transcription+repressor+activity&recordid=74154067&loginid=BH11163) | 48 | | | 6.25% | | | 0.1946 | | 0.1583 | |
| GO:0016986 | transcription initiation factor activity | [4](http://sas.ebioservice.com/bioinfoplug_molnetgotree.showgene.do?acc=GO:0016986&name=transcription+initiation+factor+activity&recordid=74154067&loginid=BH11163) | 20 | | | 20.0% | | | 0.0052 | | 0.0162 | |
| GO:0045182 | translation regulator activity | [8](http://sas.ebioservice.com/bioinfoplug_molnetgotree.showgene.do?acc=GO:0045182&name=translation+regulator+activity&recordid=74154067&loginid=BH11163) | 140 | | | 5.71% | | | 0.0763 | | 0.0949 | |
| GO:0008135 | translation factor activity, nucleic acid binding | [8](http://sas.ebioservice.com/bioinfoplug_molnetgotree.showgene.do?acc=GO:0008135&name=translation+factor+activity%2C+nucleic+acid+binding&recordid=74154067&loginid=BH11163) | 139 | | | 5.76% | | | 0.074 | | 0.0942 | |
| GO:0045735 | nutrient reservoir activity | [1](http://sas.ebioservice.com/bioinfoplug_molnetgotree.showgene.do?acc=GO:0045735&name=nutrient+reservoir+activity&recordid=74154067&loginid=BH11163) | 58 | | | 1.72% | | | 0.8328 | | 0.3779 | |
| GO:0060089 | molecular transducer activity | [15](http://sas.ebioservice.com/bioinfoplug_molnetgotree.showgene.do?acc=GO:0060089&name=molecular+transducer+activity&recordid=74154067&loginid=BH11163) | 375 | | | 4.0% | | | 0.1917 | | 0.1571 | |
| GO:0004871 | signal transducer activity | [15](http://sas.ebioservice.com/bioinfoplug_molnetgotree.showgene.do?acc=GO:0004871&name=signal+transducer+activity&recordid=74154067&loginid=BH11163) | 375 | | | 4.0% | | | 0.1917 | | 0.1571 | |
| GO:0005575 | cellular_component | [839](http://sas.ebioservice.com/bioinfoplug_molnetgotree.showgene.do?acc=GO:0005575&name=cellular_component&recordid=74154067&loginid=BH11163) | 28657 | | | 2.93% | | | 1.0 | | 0.4178 | |
| GO:0005576 | extracellular region | [29](http://sas.ebioservice.com/bioinfoplug_molnetgotree.showgene.do?acc=GO:0005576&name=extracellular+region&recordid=74154067&loginid=BH11163) | 438 | | | 6.62% | | | 2.0E-4 | | 9.0E-4 | |
| GO:0048046 | apoplast | [26](http://sas.ebioservice.com/bioinfoplug_molnetgotree.showgene.do?acc=GO:0048046&name=apoplast&recordid=74154067&loginid=BH11163) | 329 | | | 7.9% | | | 0.0 | | 2.0E-4 | |
| GO:0044421 | extracellular region part | [1](http://sas.ebioservice.com/bioinfoplug_molnetgotree.showgene.do?acc=GO:0044421&name=extracellular+region+part&recordid=74154067&loginid=BH11163) | 23 | | | 4.35% | | | 0.5167 | | 0.2883 | |
| GO:0005623 | cell | [592](http://sas.ebioservice.com/bioinfoplug_molnetgotree.showgene.do?acc=GO:0005623&name=cell&recordid=74154067&loginid=BH11163) | 15202 | | | 3.89% | | | 0.0 | | 0.0 | |
| GO:0044464 | cell part | [592](http://sas.ebioservice.com/bioinfoplug_molnetgotree.showgene.do?acc=GO:0044464&name=cell+part&recordid=74154067&loginid=BH11163) | 15202 | | | 3.89% | | | 0.0 | | 0.0 | |
| GO:0031974 | membrane-enclosed lumen | [28](http://sas.ebioservice.com/bioinfoplug_molnetgotree.showgene.do?acc=GO:0031974&name=membrane-enclosed+lumen&recordid=74154067&loginid=BH11163) | 551 | | | 5.08% | | | 0.0091 | | 0.024 | |
| GO:0043233 | organelle lumen | [28](http://sas.ebioservice.com/bioinfoplug_molnetgotree.showgene.do?acc=GO:0043233&name=organelle+lumen&recordid=74154067&loginid=BH11163) | 545 | | | 5.14% | | | 0.0080 | | 0.0216 | |
| GO:0031975 | envelope | [60](http://sas.ebioservice.com/bioinfoplug_molnetgotree.showgene.do?acc=GO:0031975&name=envelope&recordid=74154067&loginid=BH11163) | 672 | | | 8.93% | | | 0.0 | | 0.0 | |
| GO:0031967 | organelle envelope | [60](http://sas.ebioservice.com/bioinfoplug_molnetgotree.showgene.do?acc=GO:0031967&name=organelle+envelope&recordid=74154067&loginid=BH11163) | 672 | | | 8.93% | | | 0.0 | | 0.0 | |
| GO:0032991 | macromolecular complex | [58](http://sas.ebioservice.com/bioinfoplug_molnetgotree.showgene.do?acc=GO:0032991&name=macromolecular+complex&recordid=74154067&loginid=BH11163) | 1731 | | | 3.35% | | | 0.2733 | | 0.1983 | |
| GO:0030529 | ribonucleoprotein complex | [14](http://sas.ebioservice.com/bioinfoplug_molnetgotree.showgene.do?acc=GO:0030529&name=ribonucleoprotein+complex&recordid=74154067&loginid=BH11163) | 579 | | | 2.42% | | | 0.8473 | | 0.3798 | |
| GO:0032993 | protein-DNA complex | [1](http://sas.ebioservice.com/bioinfoplug_molnetgotree.showgene.do?acc=GO:0032993&name=protein-DNA+complex&recordid=74154067&loginid=BH11163) | 48 | | | 2.08% | | | 0.7735 | | 0.3571 | |
| GO:0043234 | protein complex | [46](http://sas.ebioservice.com/bioinfoplug_molnetgotree.showgene.do?acc=GO:0043234&name=protein+complex&recordid=74154067&loginid=BH11163) | 1124 | | | 4.09% | | | 0.0359 | | 0.0594 | |
| GO:0043226 | organelle | [356](http://sas.ebioservice.com/bioinfoplug_molnetgotree.showgene.do?acc=GO:0043226&name=organelle&recordid=74154067&loginid=BH11163) | 7608 | | | 4.68% | | | 0.0 | | 0.0 | |
| GO:0010168 | ER body | [1](http://sas.ebioservice.com/bioinfoplug_molnetgotree.showgene.do?acc=GO:0010168&name=ER+body&recordid=74154067&loginid=BH11163) | 3 | | | 33.33% | | | 0.1141 | | 0.1245 | |
| GO:0043227 | membrane-bounded organelle | [340](http://sas.ebioservice.com/bioinfoplug_molnetgotree.showgene.do?acc=GO:0043227&name=membrane-bounded+organelle&recordid=74154067&loginid=BH11163) | 7214 | | | 4.71% | | | 0.0 | | 0.0 | |
| GO:0043228 | non-membrane-bounded organelle | [43](http://sas.ebioservice.com/bioinfoplug_molnetgotree.showgene.do?acc=GO:0043228&name=non-membrane-bounded+organelle&recordid=74154067&loginid=BH11163) | 963 | | | 4.47% | | | 0.0124 | | 0.0297 | |
| GO:0043229 | intracellular organelle | [356](http://sas.ebioservice.com/bioinfoplug_molnetgotree.showgene.do?acc=GO:0043229&name=intracellular+organelle&recordid=74154067&loginid=BH11163) | 7607 | | | 4.68% | | | 0.0 | | 0.0 | |
| GO:0044422 | organelle part | [145](http://sas.ebioservice.com/bioinfoplug_molnetgotree.showgene.do?acc=GO:0044422&name=organelle+part&recordid=74154067&loginid=BH11163) | 2358 | | | 6.15% | | | 0.0 | | 0.0 | |
| GO:0044421 | extracellular region part | [1](http://sas.ebioservice.com/bioinfoplug_molnetgotree.showgene.do?acc=GO:0044421&name=extracellular+region+part&recordid=74154067&loginid=BH11163) | 23 | | | 4.35% | | | 0.5167 | | 0.2883 | |
| GO:0031012 | extracellular matrix | [1](http://sas.ebioservice.com/bioinfoplug_molnetgotree.showgene.do?acc=GO:0031012&name=extracellular+matrix&recordid=74154067&loginid=BH11163) | 15 | | | 6.67% | | | 0.3841 | | 0.2417 | |
| GO:0044422 | organelle part | [145](http://sas.ebioservice.com/bioinfoplug_molnetgotree.showgene.do?acc=GO:0044422&name=organelle+part&recordid=74154067&loginid=BH11163) | 2358 | | | 6.15% | | | 0.0 | | 0.0 | |
| GO:0031090 | organelle membrane | [50](http://sas.ebioservice.com/bioinfoplug_molnetgotree.showgene.do?acc=GO:0031090&name=organelle+membrane&recordid=74154067&loginid=BH11163) | 743 | | | 6.73% | | | 0.0 | | 0.0 | |
| GO:0043233 | organelle lumen | [28](http://sas.ebioservice.com/bioinfoplug_molnetgotree.showgene.do?acc=GO:0043233&name=organelle+lumen&recordid=74154067&loginid=BH11163) | 545 | | | 5.14% | | | 0.0080 | | 0.0216 | |
| GO:0044446 | intracellular organelle part | [145](http://sas.ebioservice.com/bioinfoplug_molnetgotree.showgene.do?acc=GO:0044446&name=intracellular+organelle+part&recordid=74154067&loginid=BH11163) | 2357 | | | 6.15% | | | 0.0 | | 0.0 | |
| GO:0044464 | cell part | [592](http://sas.ebioservice.com/bioinfoplug_molnetgotree.showgene.do?acc=GO:0044464&name=cell+part&recordid=74154067&loginid=BH11163) | 15202 | | | 3.89% | | | 0.0 | | 0.0 | |
| GO:0000267 | cell fraction | [1](http://sas.ebioservice.com/bioinfoplug_molnetgotree.showgene.do?acc=GO:0000267&name=cell+fraction&recordid=74154067&loginid=BH11163) | 37 | | | 2.7% | | | 0.6839 | | 0.3286 | |
| GO:0005622 | intracellular | [404](http://sas.ebioservice.com/bioinfoplug_molnetgotree.showgene.do?acc=GO:0005622&name=intracellular&recordid=74154067&loginid=BH11163) | 8856 | | | 4.56% | | | 0.0 | | 0.0 | |
| GO:0008287 | protein serine/threonine phosphatase complex | [2](http://sas.ebioservice.com/bioinfoplug_molnetgotree.showgene.do?acc=GO:0008287&name=protein+serine%2Fthreonine+phosphatase+complex&recordid=74154067&loginid=BH11163) | 58 | | | 3.45% | | | 0.538 | | 0.2883 | |
| GO:0012505 | endomembrane system | [106](http://sas.ebioservice.com/bioinfoplug_molnetgotree.showgene.do?acc=GO:0012505&name=endomembrane+system&recordid=74154067&loginid=BH11163) | 4046 | | | 2.62% | | | 0.9657 | | 0.4133 | |
| GO:0016020 | membrane | [193](http://sas.ebioservice.com/bioinfoplug_molnetgotree.showgene.do?acc=GO:0016020&name=membrane&recordid=74154067&loginid=BH11163) | 4532 | | | 4.26% | | | 0.0 | | 0.0 | |
| GO:0030312 | external encapsulating structure | [18](http://sas.ebioservice.com/bioinfoplug_molnetgotree.showgene.do?acc=GO:0030312&name=external+encapsulating+structure&recordid=74154067&loginid=BH11163) | 553 | | | 3.25% | | | 0.4394 | | 0.264 | |
| GO:0044424 | intracellular part | [386](http://sas.ebioservice.com/bioinfoplug_molnetgotree.showgene.do?acc=GO:0044424&name=intracellular+part&recordid=74154067&loginid=BH11163) | 8479 | | | 4.55% | | | 0.0 | | 0.0 | |
| GO:0044425 | membrane part | [40](http://sas.ebioservice.com/bioinfoplug_molnetgotree.showgene.do?acc=GO:0044425&name=membrane+part&recordid=74154067&loginid=BH11163) | 1305 | | | 3.07% | | | 0.5315 | | 0.2883 | |
| GO:0008150 | biological_process | [844](http://sas.ebioservice.com/bioinfoplug_molnetgotree.showgene.do?acc=GO:0008150&name=biological_process&recordid=74154067&loginid=BH11163) | 28723 | | | 2.94% | | | 1.0 | | 0.4178 | |
| GO:0000003 | reproduction | [32](http://sas.ebioservice.com/bioinfoplug_molnetgotree.showgene.do?acc=GO:0000003&name=reproduction&recordid=74154067&loginid=BH11163) | 937 | | | 3.42% | | | 0.3023 | | 0.2089 | |
| GO:0019953 | sexual reproduction | [3](http://sas.ebioservice.com/bioinfoplug_molnetgotree.showgene.do?acc=GO:0019953&name=sexual+reproduction&recordid=74154067&loginid=BH11163) | 60 | | | 5.0% | | | 0.2902 | | 0.2045 | |
| GO:0032504 | multicellular organism reproduction | [1](http://sas.ebioservice.com/bioinfoplug_molnetgotree.showgene.do?acc=GO:0032504&name=multicellular+organism+reproduction&recordid=74154067&loginid=BH11163) | 34 | | | 2.94% | | | 0.6538 | | 0.317 | |
| GO:0022414 | reproductive process | [32](http://sas.ebioservice.com/bioinfoplug_molnetgotree.showgene.do?acc=GO:0022414&name=reproductive+process&recordid=74154067&loginid=BH11163) | 920 | | | 3.48% | | | 0.2685 | | 0.1971 | |
| GO:0002376 | immune system process | [9](http://sas.ebioservice.com/bioinfoplug_molnetgotree.showgene.do?acc=GO:0002376&name=immune+system+process&recordid=74154067&loginid=BH11163) | 313 | | | 2.88% | | | 0.6251 | | 0.3059 | |
| GO:0002252 | immune effector process | [1](http://sas.ebioservice.com/bioinfoplug_molnetgotree.showgene.do?acc=GO:0002252&name=immune+effector+process&recordid=74154067&loginid=BH11163) | 25 | | | 4.0% | | | 0.5452 | | 0.2883 | |
| GO:0002253 | activation of immune response | [2](http://sas.ebioservice.com/bioinfoplug_molnetgotree.showgene.do?acc=GO:0002253&name=activation+of+immune+response&recordid=74154067&loginid=BH11163) | 29 | | | 6.9% | | | 0.2362 | | 0.1877 | |
| GO:0006955 | immune response | [9](http://sas.ebioservice.com/bioinfoplug_molnetgotree.showgene.do?acc=GO:0006955&name=immune+response&recordid=74154067&loginid=BH11163) | 312 | | | 2.88% | | | 0.6214 | | 0.3059 | |
| GO:0002682 | regulation of immune system process | [3](http://sas.ebioservice.com/bioinfoplug_molnetgotree.showgene.do?acc=GO:0002682&name=regulation+of+immune+system+process&recordid=74154067&loginid=BH11163) | 37 | | | 8.11% | | | 0.1163 | | 0.1245 | |
| GO:0002684 | positive regulation of immune system process | [2](http://sas.ebioservice.com/bioinfoplug_molnetgotree.showgene.do?acc=GO:0002684&name=positive+regulation+of+immune+system+process&recordid=74154067&loginid=BH11163) | 29 | | | 6.9% | | | 0.2362 | | 0.1877 | |
| GO:0008152 | metabolic process | [322](http://sas.ebioservice.com/bioinfoplug_molnetgotree.showgene.do?acc=GO:0008152&name=metabolic+process&recordid=74154067&loginid=BH11163) | 9176 | | | 3.51% | | | 0.0035 | | 0.0118 | |
| GO:0006807 | nitrogen compound metabolic process | [30](http://sas.ebioservice.com/bioinfoplug_molnetgotree.showgene.do?acc=GO:0006807&name=nitrogen+compound+metabolic+process&recordid=74154067&loginid=BH11163) | 451 | | | 6.65% | | | 1.0E-4 | | 7.0E-4 | |
| GO:0009056 | catabolic process | [29](http://sas.ebioservice.com/bioinfoplug_molnetgotree.showgene.do?acc=GO:0009056&name=catabolic+process&recordid=74154067&loginid=BH11163) | 686 | | | 4.23% | | | 0.0605 | | 0.0831 | |
| GO:0009058 | biosynthetic process | [143](http://sas.ebioservice.com/bioinfoplug_molnetgotree.showgene.do?acc=GO:0009058&name=biosynthetic+process&recordid=74154067&loginid=BH11163) | 4277 | | | 3.34% | | | 0.1561 | | 0.1435 | |
| GO:0015976 | carbon utilization | [3](http://sas.ebioservice.com/bioinfoplug_molnetgotree.showgene.do?acc=GO:0015976&name=carbon+utilization&recordid=74154067&loginid=BH11163) | 17 | | | 17.65% | | | 0.0207 | | 0.042 | |
| GO:0019748 | secondary metabolic process | [19](http://sas.ebioservice.com/bioinfoplug_molnetgotree.showgene.do?acc=GO:0019748&name=secondary+metabolic+process&recordid=74154067&loginid=BH11163) | 383 | | | 4.96% | | | 0.0341 | | 0.0594 | |
| GO:0042440 | pigment metabolic process | [13](http://sas.ebioservice.com/bioinfoplug_molnetgotree.showgene.do?acc=GO:0042440&name=pigment+metabolic+process&recordid=74154067&loginid=BH11163) | 110 | | | 11.82% | | | 1.0E-4 | | 4.0E-4 | |
| GO:0042445 | hormone metabolic process | [3](http://sas.ebioservice.com/bioinfoplug_molnetgotree.showgene.do?acc=GO:0042445&name=hormone+metabolic+process&recordid=74154067&loginid=BH11163) | 100 | | | 3.0% | | | 0.5969 | | 0.3019 | |
| GO:0043170 | macromolecule metabolic process | [158](http://sas.ebioservice.com/bioinfoplug_molnetgotree.showgene.do?acc=GO:0043170&name=macromolecule+metabolic+process&recordid=74154067&loginid=BH11163) | 6189 | | | 2.55% | | | 0.9962 | | 0.4178 | |
| GO:0044237 | cellular metabolic process | [257](http://sas.ebioservice.com/bioinfoplug_molnetgotree.showgene.do?acc=GO:0044237&name=cellular+metabolic+process&recordid=74154067&loginid=BH11163) | 7840 | | | 3.28% | | | 0.1308 | | 0.1345 | |
| GO:0044238 | primary metabolic process | [242](http://sas.ebioservice.com/bioinfoplug_molnetgotree.showgene.do?acc=GO:0044238&name=primary+metabolic+process&recordid=74154067&loginid=BH11163) | 7802 | | | 3.1% | | | 0.4521 | | 0.2641 | |
| GO:0055114 | oxidation reduction | [10](http://sas.ebioservice.com/bioinfoplug_molnetgotree.showgene.do?acc=GO:0055114&name=oxidation+reduction&recordid=74154067&loginid=BH11163) | 163 | | | 6.13% | | | 0.0353 | | 0.0594 | |
| GO:0009892 | negative regulation of metabolic process | [9](http://sas.ebioservice.com/bioinfoplug_molnetgotree.showgene.do?acc=GO:0009892&name=negative+regulation+of+metabolic+process&recordid=74154067&loginid=BH11163) | 190 | | | 4.74% | | | 0.1425 | | 0.1345 | |
| GO:0009893 | positive regulation of metabolic process | [4](http://sas.ebioservice.com/bioinfoplug_molnetgotree.showgene.do?acc=GO:0009893&name=positive+regulation+of+metabolic+process&recordid=74154067&loginid=BH11163) | 80 | | | 5.0% | | | 0.2419 | | 0.1895 | |
| GO:0019222 | regulation of metabolic process | [60](http://sas.ebioservice.com/bioinfoplug_molnetgotree.showgene.do?acc=GO:0019222&name=regulation+of+metabolic+process&recordid=74154067&loginid=BH11163) | 1928 | | | 3.11% | | | 0.4822 | | 0.2726 | |
| GO:0009987 | cellular process | [367](http://sas.ebioservice.com/bioinfoplug_molnetgotree.showgene.do?acc=GO:0009987&name=cellular+process&recordid=74154067&loginid=BH11163) | 10432 | | | 3.52% | | | 0.0012 | | 0.0047 | |
| GO:0001708 | cell fate specification | [3](http://sas.ebioservice.com/bioinfoplug_molnetgotree.showgene.do?acc=GO:0001708&name=cell+fate+specification&recordid=74154067&loginid=BH11163) | 25 | | | 12.0% | | | 0.0499 | | 0.0743 | |
| GO:0001709 | cell fate determination | [1](http://sas.ebioservice.com/bioinfoplug_molnetgotree.showgene.do?acc=GO:0001709&name=cell+fate+determination&recordid=74154067&loginid=BH11163) | 4 | | | 25.0% | | | 0.1405 | | 0.1345 | |
| GO:0006413 | translational initiation | [1](http://sas.ebioservice.com/bioinfoplug_molnetgotree.showgene.do?acc=GO:0006413&name=translational+initiation&recordid=74154067&loginid=BH11163) | 76 | | | 1.32% | | | 0.9032 | | 0.3978 | |
| GO:0007017 | microtubule-based process | [11](http://sas.ebioservice.com/bioinfoplug_molnetgotree.showgene.do?acc=GO:0007017&name=microtubule-based+process&recordid=74154067&loginid=BH11163) | 107 | | | 10.28% | | | 8.0E-4 | | 0.0036 | |
| GO:0007049 | cell cycle | [14](http://sas.ebioservice.com/bioinfoplug_molnetgotree.showgene.do?acc=GO:0007049&name=cell+cycle&recordid=74154067&loginid=BH11163) | 233 | | | 6.01% | | | 0.017 | | 0.0366 | |
| GO:0007059 | chromosome segregation | [2](http://sas.ebioservice.com/bioinfoplug_molnetgotree.showgene.do?acc=GO:0007059&name=chromosome+segregation&recordid=74154067&loginid=BH11163) | 22 | | | 9.09% | | | 0.1598 | | 0.1456 | |
| GO:0007154 | cell communication | [35](http://sas.ebioservice.com/bioinfoplug_molnetgotree.showgene.do?acc=GO:0007154&name=cell+communication&recordid=74154067&loginid=BH11163) | 1267 | | | 2.76% | | | 0.7613 | | 0.3544 | |
| GO:0007155 | cell adhesion | [1](http://sas.ebioservice.com/bioinfoplug_molnetgotree.showgene.do?acc=GO:0007155&name=cell+adhesion&recordid=74154067&loginid=BH11163) | 17 | | | 5.88% | | | 0.4203 | | 0.2584 | |
| GO:0008037 | cell recognition | [1](http://sas.ebioservice.com/bioinfoplug_molnetgotree.showgene.do?acc=GO:0008037&name=cell+recognition&recordid=74154067&loginid=BH11163) | 30 | | | 3.33% | | | 0.6091 | | 0.3059 | |
| GO:0008219 | cell death | [9](http://sas.ebioservice.com/bioinfoplug_molnetgotree.showgene.do?acc=GO:0008219&name=cell+death&recordid=74154067&loginid=BH11163) | 237 | | | 3.8% | | | 0.3143 | | 0.213 | |
| GO:0008283 | cell proliferation | [3](http://sas.ebioservice.com/bioinfoplug_molnetgotree.showgene.do?acc=GO:0008283&name=cell+proliferation&recordid=74154067&loginid=BH11163) | 58 | | | 5.17% | | | 0.2739 | | 0.1983 | |
| GO:0010118 | stomatal movement | [4](http://sas.ebioservice.com/bioinfoplug_molnetgotree.showgene.do?acc=GO:0010118&name=stomatal+movement&recordid=74154067&loginid=BH11163) | 43 | | | 9.3% | | | 0.0511 | | 0.075 | |
| GO:0016043 | cellular component organization | [40](http://sas.ebioservice.com/bioinfoplug_molnetgotree.showgene.do?acc=GO:0016043&name=cellular+component+organization&recordid=74154067&loginid=BH11163) | 966 | | | 4.14% | | | 0.0415 | | 0.0631 | |
| GO:0016049 | cell growth | [7](http://sas.ebioservice.com/bioinfoplug_molnetgotree.showgene.do?acc=GO:0016049&name=cell+growth&recordid=74154067&loginid=BH11163) | 229 | | | 3.06% | | | 0.5592 | | 0.2927 | |
| GO:0016192 | vesicle-mediated transport | [1](http://sas.ebioservice.com/bioinfoplug_molnetgotree.showgene.do?acc=GO:0016192&name=vesicle-mediated+transport&recordid=74154067&loginid=BH11163) | 237 | | | 0.42% | | | 0.9993 | | 0.4178 | |
| GO:0016458 | gene silencing | [2](http://sas.ebioservice.com/bioinfoplug_molnetgotree.showgene.do?acc=GO:0016458&name=gene+silencing&recordid=74154067&loginid=BH11163) | 116 | | | 1.72% | | | 0.8707 | | 0.3884 | |
| GO:0019725 | cellular homeostasis | [11](http://sas.ebioservice.com/bioinfoplug_molnetgotree.showgene.do?acc=GO:0019725&name=cellular+homeostasis&recordid=74154067&loginid=BH11163) | 183 | | | 6.01% | | | 0.0318 | | 0.0594 | |
| GO:0022402 | cell cycle process | [10](http://sas.ebioservice.com/bioinfoplug_molnetgotree.showgene.do?acc=GO:0022402&name=cell+cycle+process&recordid=74154067&loginid=BH11163) | 130 | | | 7.69% | | | 0.0094 | | 0.0243 | |
| GO:0030029 | actin filament-based process | [7](http://sas.ebioservice.com/bioinfoplug_molnetgotree.showgene.do?acc=GO:0030029&name=actin+filament-based+process&recordid=74154067&loginid=BH11163) | 80 | | | 8.75% | | | 0.0153 | | 0.035 | |
| GO:0032506 | cytokinetic process | [2](http://sas.ebioservice.com/bioinfoplug_molnetgotree.showgene.do?acc=GO:0032506&name=cytokinetic+process&recordid=74154067&loginid=BH11163) | 12 | | | 16.67% | | | 0.0638 | | 0.0837 | |
| GO:0044085 | cellular component biogenesis | [25](http://sas.ebioservice.com/bioinfoplug_molnetgotree.showgene.do?acc=GO:0044085&name=cellular+component+biogenesis&recordid=74154067&loginid=BH11163) | 552 | | | 4.53% | | | 0.0418 | | 0.0631 | |
| GO:0044237 | cellular metabolic process | [257](http://sas.ebioservice.com/bioinfoplug_molnetgotree.showgene.do?acc=GO:0044237&name=cellular+metabolic+process&recordid=74154067&loginid=BH11163) | 7840 | | | 3.28% | | | 0.1308 | | 0.1345 | |
| GO:0045165 | cell fate commitment | [5](http://sas.ebioservice.com/bioinfoplug_molnetgotree.showgene.do?acc=GO:0045165&name=cell+fate+commitment&recordid=74154067&loginid=BH11163) | 32 | | | 15.63% | | | 0.0046 | | 0.0153 | |
| GO:0048468 | cell development | [6](http://sas.ebioservice.com/bioinfoplug_molnetgotree.showgene.do?acc=GO:0048468&name=cell+development&recordid=74154067&loginid=BH11163) | 158 | | | 3.8% | | | 0.3649 | | 0.2354 | |
| GO:0048869 | cellular developmental process | [17](http://sas.ebioservice.com/bioinfoplug_molnetgotree.showgene.do?acc=GO:0048869&name=cellular+developmental+process&recordid=74154067&loginid=BH11163) | 324 | | | 5.25% | | | 0.0282 | | 0.0542 | |
| GO:0051301 | cell division | [8](http://sas.ebioservice.com/bioinfoplug_molnetgotree.showgene.do?acc=GO:0051301&name=cell+division&recordid=74154067&loginid=BH11163) | 84 | | | 9.52% | | | 0.0063 | | 0.0179 | |
| GO:0051641 | cellular localization | [24](http://sas.ebioservice.com/bioinfoplug_molnetgotree.showgene.do?acc=GO:0051641&name=cellular+localization&recordid=74154067&loginid=BH11163) | 516 | | | 4.65% | | | 0.0356 | | 0.0594 | |
| GO:0051716 | cellular response to stimulus | [14](http://sas.ebioservice.com/bioinfoplug_molnetgotree.showgene.do?acc=GO:0051716&name=cellular+response+to+stimulus&recordid=74154067&loginid=BH11163) | 259 | | | 5.41% | | | 0.0357 | | 0.0594 | |
| GO:0055085 | transmembrane transport | [6](http://sas.ebioservice.com/bioinfoplug_molnetgotree.showgene.do?acc=GO:0055085&name=transmembrane+transport&recordid=74154067&loginid=BH11163) | 152 | | | 3.95% | | | 0.333 | | 0.22 | |
| GO:0048522 | positive regulation of cellular process | [8](http://sas.ebioservice.com/bioinfoplug_molnetgotree.showgene.do?acc=GO:0048522&name=positive+regulation+of+cellular+process&recordid=74154067&loginid=BH11163) | 132 | | | 6.06% | | | 0.0591 | | 0.0831 | |
| GO:0048523 | negative regulation of cellular process | [9](http://sas.ebioservice.com/bioinfoplug_molnetgotree.showgene.do?acc=GO:0048523&name=negative+regulation+of+cellular+process&recordid=74154067&loginid=BH11163) | 181 | | | 4.97% | | | 0.1166 | | 0.1245 | |
| GO:0050794 | regulation of cellular process | [102](http://sas.ebioservice.com/bioinfoplug_molnetgotree.showgene.do?acc=GO:0050794&name=regulation+of+cellular+process&recordid=74154067&loginid=BH11163) | 3034 | | | 3.36% | | | 0.1895 | | 0.1571 | |
| GO:0010926 | anatomical structure formation | [17](http://sas.ebioservice.com/bioinfoplug_molnetgotree.showgene.do?acc=GO:0010926&name=anatomical+structure+formation&recordid=74154067&loginid=BH11163) | 341 | | | 4.99% | | | 0.0416 | | 0.0631 | |
| GO:0022607 | cellular component assembly | [16](http://sas.ebioservice.com/bioinfoplug_molnetgotree.showgene.do?acc=GO:0022607&name=cellular+component+assembly&recordid=74154067&loginid=BH11163) | 272 | | | 5.88% | | | 0.0134 | | 0.0314 | |
| GO:0048646 | anatomical structure formation involved in morphogenesis | [1](http://sas.ebioservice.com/bioinfoplug_molnetgotree.showgene.do?acc=GO:0048646&name=anatomical+structure+formation+involved+in+morphogenesis&recordid=74154067&loginid=BH11163) | 94 | | | 1.06% | | | 0.944 | | 0.409 | |
| GO:0016265 | death | [9](http://sas.ebioservice.com/bioinfoplug_molnetgotree.showgene.do?acc=GO:0016265&name=death&recordid=74154067&loginid=BH11163) | 237 | | | 3.8% | | | 0.3143 | | 0.213 | |
| GO:0008219 | cell death | [9](http://sas.ebioservice.com/bioinfoplug_molnetgotree.showgene.do?acc=GO:0008219&name=cell+death&recordid=74154067&loginid=BH11163) | 237 | | | 3.8% | | | 0.3143 | | 0.213 | |
| GO:0022414 | reproductive process | [32](http://sas.ebioservice.com/bioinfoplug_molnetgotree.showgene.do?acc=GO:0022414&name=reproductive+process&recordid=74154067&loginid=BH11163) | 920 | | | 3.48% | | | 0.2685 | | 0.1971 | |
| GO:0003006 | reproductive developmental process | [26](http://sas.ebioservice.com/bioinfoplug_molnetgotree.showgene.do?acc=GO:0003006&name=reproductive+developmental+process&recordid=74154067&loginid=BH11163) | 764 | | | 3.4% | | | 0.3318 | | 0.22 | |
| GO:0007276 | gamete generation | [2](http://sas.ebioservice.com/bioinfoplug_molnetgotree.showgene.do?acc=GO:0007276&name=gamete+generation&recordid=74154067&loginid=BH11163) | 20 | | | 10.0% | | | 0.1389 | | 0.1345 | |
| GO:0009566 | fertilization | [1](http://sas.ebioservice.com/bioinfoplug_molnetgotree.showgene.do?acc=GO:0009566&name=fertilization&recordid=74154067&loginid=BH11163) | 24 | | | 4.17% | | | 0.5312 | | 0.2883 | |
| GO:0009856 | pollination | [3](http://sas.ebioservice.com/bioinfoplug_molnetgotree.showgene.do?acc=GO:0009856&name=pollination&recordid=74154067&loginid=BH11163) | 127 | | | 2.36% | | | 0.7485 | | 0.3517 | |
| GO:0048609 | reproductive process in a multicellular organism | [1](http://sas.ebioservice.com/bioinfoplug_molnetgotree.showgene.do?acc=GO:0048609&name=reproductive+process+in+a+multicellular+organism&recordid=74154067&loginid=BH11163) | 31 | | | 3.23% | | | 0.6208 | | 0.3059 | |
| GO:0022610 | biological adhesion | [1](http://sas.ebioservice.com/bioinfoplug_molnetgotree.showgene.do?acc=GO:0022610&name=biological+adhesion&recordid=74154067&loginid=BH11163) | 17 | | | 5.88% | | | 0.4203 | | 0.2584 | |
| GO:0007155 | cell adhesion | [1](http://sas.ebioservice.com/bioinfoplug_molnetgotree.showgene.do?acc=GO:0007155&name=cell+adhesion&recordid=74154067&loginid=BH11163) | 17 | | | 5.88% | | | 0.4203 | | 0.2584 | |
| GO:0032501 | multicellular organismal process | [59](http://sas.ebioservice.com/bioinfoplug_molnetgotree.showgene.do?acc=GO:0032501&name=multicellular+organismal+process&recordid=74154067&loginid=BH11163) | 1658 | | | 3.56% | | | 0.1449 | | 0.1356 | |
| GO:0003008 | system process | [1](http://sas.ebioservice.com/bioinfoplug_molnetgotree.showgene.do?acc=GO:0003008&name=system+process&recordid=74154067&loginid=BH11163) | 14 | | | 7.14% | | | 0.3652 | | 0.2354 | |
| GO:0007275 | multicellular organismal development | [57](http://sas.ebioservice.com/bioinfoplug_molnetgotree.showgene.do?acc=GO:0007275&name=multicellular+organismal+development&recordid=74154067&loginid=BH11163) | 1617 | | | 3.53% | | | 0.1663 | | 0.1477 | |
| GO:0009606 | tropism | [2](http://sas.ebioservice.com/bioinfoplug_molnetgotree.showgene.do?acc=GO:0009606&name=tropism&recordid=74154067&loginid=BH11163) | 49 | | | 4.08% | | | 0.4521 | | 0.2641 | |
| GO:0009845 | seed germination | [3](http://sas.ebioservice.com/bioinfoplug_molnetgotree.showgene.do?acc=GO:0009845&name=seed+germination&recordid=74154067&loginid=BH11163) | 65 | | | 4.62% | | | 0.3312 | | 0.22 | |
| GO:0032504 | multicellular organism reproduction | [1](http://sas.ebioservice.com/bioinfoplug_molnetgotree.showgene.do?acc=GO:0032504&name=multicellular+organism+reproduction&recordid=74154067&loginid=BH11163) | 34 | | | 2.94% | | | 0.6538 | | 0.317 | |
| GO:0048609 | reproductive process in a multicellular organism | [1](http://sas.ebioservice.com/bioinfoplug_molnetgotree.showgene.do?acc=GO:0048609&name=reproductive+process+in+a+multicellular+organism&recordid=74154067&loginid=BH11163) | 31 | | | 3.23% | | | 0.6208 | | 0.3059 | |
| GO:0051239 | regulation of multicellular organismal process | [10](http://sas.ebioservice.com/bioinfoplug_molnetgotree.showgene.do?acc=GO:0051239&name=regulation+of+multicellular+organismal+process&recordid=74154067&loginid=BH11163) | 202 | | | 4.95% | | | 0.1041 | | 0.1171 | |
| GO:0051241 | negative regulation of multicellular organismal process | [1](http://sas.ebioservice.com/bioinfoplug_molnetgotree.showgene.do?acc=GO:0051241&name=negative+regulation+of+multicellular+organismal+process&recordid=74154067&loginid=BH11163) | 9 | | | 11.11% | | | 0.2613 | | 0.1971 | |
| GO:0032502 | developmental process | [62](http://sas.ebioservice.com/bioinfoplug_molnetgotree.showgene.do?acc=GO:0032502&name=developmental+process&recordid=74154067&loginid=BH11163) | 1756 | | | 3.53% | | | 0.1516 | | 0.1406 | |
| GO:0003006 | reproductive developmental process | [26](http://sas.ebioservice.com/bioinfoplug_molnetgotree.showgene.do?acc=GO:0003006&name=reproductive+developmental+process&recordid=74154067&loginid=BH11163) | 764 | | | 3.4% | | | 0.3318 | | 0.22 | |
| GO:0007275 | multicellular organismal development | [57](http://sas.ebioservice.com/bioinfoplug_molnetgotree.showgene.do?acc=GO:0007275&name=multicellular+organismal+development&recordid=74154067&loginid=BH11163) | 1617 | | | 3.53% | | | 0.1663 | | 0.1477 | |
| GO:0007389 | pattern specification process | [7](http://sas.ebioservice.com/bioinfoplug_molnetgotree.showgene.do?acc=GO:0007389&name=pattern+specification+process&recordid=74154067&loginid=BH11163) | 119 | | | 5.88% | | | 0.0836 | | 0.1016 | |
| GO:0007568 | aging | [1](http://sas.ebioservice.com/bioinfoplug_molnetgotree.showgene.do?acc=GO:0007568&name=aging&recordid=74154067&loginid=BH11163) | 74 | | | 1.35% | | | 0.8971 | | 0.3968 | |
| GO:0009561 | megagametogenesis | [1](http://sas.ebioservice.com/bioinfoplug_molnetgotree.showgene.do?acc=GO:0009561&name=megagametogenesis&recordid=74154067&loginid=BH11163) | 45 | | | 2.22% | | | 0.752 | | 0.3517 | |
| GO:0009653 | anatomical structure morphogenesis | [12](http://sas.ebioservice.com/bioinfoplug_molnetgotree.showgene.do?acc=GO:0009653&name=anatomical+structure+morphogenesis&recordid=74154067&loginid=BH11163) | 389 | | | 3.08% | | | 0.5366 | | 0.2883 | |
| GO:0009790 | embryonic development | [12](http://sas.ebioservice.com/bioinfoplug_molnetgotree.showgene.do?acc=GO:0009790&name=embryonic+development&recordid=74154067&loginid=BH11163) | 415 | | | 2.89% | | | 0.6238 | | 0.3059 | |
| GO:0009791 | post-embryonic development | [34](http://sas.ebioservice.com/bioinfoplug_molnetgotree.showgene.do?acc=GO:0009791&name=post-embryonic+development&recordid=74154067&loginid=BH11163) | 928 | | | 3.66% | | | 0.1766 | | 0.1556 | |
| GO:0009838 | abscission | [1](http://sas.ebioservice.com/bioinfoplug_molnetgotree.showgene.do?acc=GO:0009838&name=abscission&recordid=74154067&loginid=BH11163) | 17 | | | 5.88% | | | 0.4203 | | 0.2584 | |
| GO:0010228 | vegetative to reproductive phase transition | [3](http://sas.ebioservice.com/bioinfoplug_molnetgotree.showgene.do?acc=GO:0010228&name=vegetative+to+reproductive+phase+transition&recordid=74154067&loginid=BH11163) | 80 | | | 3.75% | | | 0.4521 | | 0.2641 | |
| GO:0048532 | anatomical structure arrangement | [1](http://sas.ebioservice.com/bioinfoplug_molnetgotree.showgene.do?acc=GO:0048532&name=anatomical+structure+arrangement&recordid=74154067&loginid=BH11163) | 45 | | | 2.22% | | | 0.752 | | 0.3517 | |
| GO:0048589 | developmental growth | [2](http://sas.ebioservice.com/bioinfoplug_molnetgotree.showgene.do?acc=GO:0048589&name=developmental+growth&recordid=74154067&loginid=BH11163) | 105 | | | 1.9% | | | 0.8326 | | 0.3779 | |
| GO:0048646 | anatomical structure formation involved in morphogenesis | [1](http://sas.ebioservice.com/bioinfoplug_molnetgotree.showgene.do?acc=GO:0048646&name=anatomical+structure+formation+involved+in+morphogenesis&recordid=74154067&loginid=BH11163) | 94 | | | 1.06% | | | 0.944 | | 0.409 | |
| GO:0048856 | anatomical structure development | [44](http://sas.ebioservice.com/bioinfoplug_molnetgotree.showgene.do?acc=GO:0048856&name=anatomical+structure+development&recordid=74154067&loginid=BH11163) | 1307 | | | 3.37% | | | 0.295 | | 0.2066 | |
| GO:0048869 | cellular developmental process | [17](http://sas.ebioservice.com/bioinfoplug_molnetgotree.showgene.do?acc=GO:0048869&name=cellular+developmental+process&recordid=74154067&loginid=BH11163) | 324 | | | 5.25% | | | 0.0282 | | 0.0542 | |
| GO:0055046 | microgametogenesis | [2](http://sas.ebioservice.com/bioinfoplug_molnetgotree.showgene.do?acc=GO:0055046&name=microgametogenesis&recordid=74154067&loginid=BH11163) | 9 | | | 22.22% | | | 0.0409 | | 0.0631 | |
| GO:0050793 | regulation of developmental process | [12](http://sas.ebioservice.com/bioinfoplug_molnetgotree.showgene.do?acc=GO:0050793&name=regulation+of+developmental+process&recordid=74154067&loginid=BH11163) | 271 | | | 4.43% | | | 0.1423 | | 0.1345 | |
| GO:0051093 | negative regulation of developmental process | [3](http://sas.ebioservice.com/bioinfoplug_molnetgotree.showgene.do?acc=GO:0051093&name=negative+regulation+of+developmental+process&recordid=74154067&loginid=BH11163) | 92 | | | 3.26% | | | 0.5419 | | 0.2883 | |
| GO:0051094 | positive regulation of developmental process | [2](http://sas.ebioservice.com/bioinfoplug_molnetgotree.showgene.do?acc=GO:0051094&name=positive+regulation+of+developmental+process&recordid=74154067&loginid=BH11163) | 50 | | | 4.0% | | | 0.4621 | | 0.2669 | |
| GO:0040007 | growth | [9](http://sas.ebioservice.com/bioinfoplug_molnetgotree.showgene.do?acc=GO:0040007&name=growth&recordid=74154067&loginid=BH11163) | 291 | | | 3.09% | | | 0.5402 | | 0.2883 | |
| GO:0016049 | cell growth | [7](http://sas.ebioservice.com/bioinfoplug_molnetgotree.showgene.do?acc=GO:0016049&name=cell+growth&recordid=74154067&loginid=BH11163) | 229 | | | 3.06% | | | 0.5592 | | 0.2927 | |
| GO:0048589 | developmental growth | [2](http://sas.ebioservice.com/bioinfoplug_molnetgotree.showgene.do?acc=GO:0048589&name=developmental+growth&recordid=74154067&loginid=BH11163) | 105 | | | 1.9% | | | 0.8326 | | 0.3779 | |
| GO:0040008 | regulation of growth | [2](http://sas.ebioservice.com/bioinfoplug_molnetgotree.showgene.do?acc=GO:0040008&name=regulation+of+growth&recordid=74154067&loginid=BH11163) | 48 | | | 4.17% | | | 0.442 | | 0.264 | |
| GO:0045926 | negative regulation of growth | [1](http://sas.ebioservice.com/bioinfoplug_molnetgotree.showgene.do?acc=GO:0045926&name=negative+regulation+of+growth&recordid=74154067&loginid=BH11163) | 9 | | | 11.11% | | | 0.2613 | | 0.1971 | |
| GO:0045927 | positive regulation of growth | [1](http://sas.ebioservice.com/bioinfoplug_molnetgotree.showgene.do?acc=GO:0045927&name=positive+regulation+of+growth&recordid=74154067&loginid=BH11163) | 4 | | | 25.0% | | | 0.1405 | | 0.1345 | |
| GO:0048511 | rhythmic process | [6](http://sas.ebioservice.com/bioinfoplug_molnetgotree.showgene.do?acc=GO:0048511&name=rhythmic+process&recordid=74154067&loginid=BH11163) | 49 | | | 12.24% | | | 0.0058 | | 0.017 | |
| GO:0007623 | circadian rhythm | [6](http://sas.ebioservice.com/bioinfoplug_molnetgotree.showgene.do?acc=GO:0007623&name=circadian+rhythm&recordid=74154067&loginid=BH11163) | 49 | | | 12.24% | | | 0.0058 | | 0.017 | |
| GO:0050896 | response to stimulus | [153](http://sas.ebioservice.com/bioinfoplug_molnetgotree.showgene.do?acc=GO:0050896&name=response+to+stimulus&recordid=74154067&loginid=BH11163) | 3431 | | | 4.46% | | | 0.0 | | 0.0 | |
| GO:0006950 | response to stress | [79](http://sas.ebioservice.com/bioinfoplug_molnetgotree.showgene.do?acc=GO:0006950&name=response+to+stress&recordid=74154067&loginid=BH11163) | 1955 | | | 4.04% | | | 0.0102 | | 0.025 | |
| GO:0006955 | immune response | [9](http://sas.ebioservice.com/bioinfoplug_molnetgotree.showgene.do?acc=GO:0006955&name=immune+response&recordid=74154067&loginid=BH11163) | 312 | | | 2.88% | | | 0.6214 | | 0.3059 | |
| GO:0009605 | response to external stimulus | [10](http://sas.ebioservice.com/bioinfoplug_molnetgotree.showgene.do?acc=GO:0009605&name=response+to+external+stimulus&recordid=74154067&loginid=BH11163) | 332 | | | 3.01% | | | 0.5702 | | 0.2969 | |
| GO:0009607 | response to biotic stimulus | [18](http://sas.ebioservice.com/bioinfoplug_molnetgotree.showgene.do?acc=GO:0009607&name=response+to+biotic+stimulus&recordid=74154067&loginid=BH11163) | 583 | | | 3.09% | | | 0.5269 | | 0.2883 | |
| GO:0009628 | response to abiotic stimulus | [81](http://sas.ebioservice.com/bioinfoplug_molnetgotree.showgene.do?acc=GO:0009628&name=response+to+abiotic+stimulus&recordid=74154067&loginid=BH11163) | 1156 | | | 7.01% | | | 0.0 | | 0.0 | |
| GO:0009719 | response to endogenous stimulus | [37](http://sas.ebioservice.com/bioinfoplug_molnetgotree.showgene.do?acc=GO:0009719&name=response+to+endogenous+stimulus&recordid=74154067&loginid=BH11163) | 832 | | | 4.45% | | | 0.0203 | | 0.042 | |
| GO:0042221 | response to chemical stimulus | [70](http://sas.ebioservice.com/bioinfoplug_molnetgotree.showgene.do?acc=GO:0042221&name=response+to+chemical+stimulus&recordid=74154067&loginid=BH11163) | 1746 | | | 4.01% | | | 0.0175 | | 0.0369 | |
| GO:0051606 | detection of stimulus | [1](http://sas.ebioservice.com/bioinfoplug_molnetgotree.showgene.do?acc=GO:0051606&name=detection+of+stimulus&recordid=74154067&loginid=BH11163) | 37 | | | 2.7% | | | 0.6839 | | 0.3286 | |
| GO:0051716 | cellular response to stimulus | [14](http://sas.ebioservice.com/bioinfoplug_molnetgotree.showgene.do?acc=GO:0051716&name=cellular+response+to+stimulus&recordid=74154067&loginid=BH11163) | 259 | | | 5.41% | | | 0.0357 | | 0.0594 | |
| GO:0048583 | regulation of response to stimulus | [7](http://sas.ebioservice.com/bioinfoplug_molnetgotree.showgene.do?acc=GO:0048583&name=regulation+of+response+to+stimulus&recordid=74154067&loginid=BH11163) | 150 | | | 4.67% | | | 0.19 | | 0.1571 | |
| GO:0048584 | positive regulation of response to stimulus | [3](http://sas.ebioservice.com/bioinfoplug_molnetgotree.showgene.do?acc=GO:0048584&name=positive+regulation+of+response+to+stimulus&recordid=74154067&loginid=BH11163) | 57 | | | 5.26% | | | 0.2658 | | 0.1971 | |
| GO:0048585 | negative regulation of response to stimulus | [2](http://sas.ebioservice.com/bioinfoplug_molnetgotree.showgene.do?acc=GO:0048585&name=negative+regulation+of+response+to+stimulus&recordid=74154067&loginid=BH11163) | 64 | | | 3.13% | | | 0.5898 | | 0.3012 | |
| GO:0051179 | localization | [71](http://sas.ebioservice.com/bioinfoplug_molnetgotree.showgene.do?acc=GO:0051179&name=localization&recordid=74154067&loginid=BH11163) | 1819 | | | 3.9% | | | 0.0276 | | 0.0542 | |
| GO:0033036 | macromolecule localization | [8](http://sas.ebioservice.com/bioinfoplug_molnetgotree.showgene.do?acc=GO:0033036&name=macromolecule+localization&recordid=74154067&loginid=BH11163) | 429 | | | 1.86% | | | 0.9506 | | 0.4102 | |
| GO:0051641 | cellular localization | [24](http://sas.ebioservice.com/bioinfoplug_molnetgotree.showgene.do?acc=GO:0051641&name=cellular+localization&recordid=74154067&loginid=BH11163) | 516 | | | 4.65% | | | 0.0356 | | 0.0594 | |
| GO:0032879 | regulation of localization | [1](http://sas.ebioservice.com/bioinfoplug_molnetgotree.showgene.do?acc=GO:0032879&name=regulation+of+localization&recordid=74154067&loginid=BH11163) | 20 | | | 5.0% | | | 0.4707 | | 0.269 | |
| GO:0051234 | establishment of localization | [68](http://sas.ebioservice.com/bioinfoplug_molnetgotree.showgene.do?acc=GO:0051234&name=establishment+of+localization&recordid=74154067&loginid=BH11163) | 1760 | | | 3.86% | | | 0.0365 | | 0.0594 | |
| GO:0051235 | maintenance of location | [2](http://sas.ebioservice.com/bioinfoplug_molnetgotree.showgene.do?acc=GO:0051235&name=maintenance+of+location&recordid=74154067&loginid=BH11163) | 15 | | | 13.33% | | | 0.09 | | 0.1057 | |
| GO:0051234 | establishment of localization | [68](http://sas.ebioservice.com/bioinfoplug_molnetgotree.showgene.do?acc=GO:0051234&name=establishment+of+localization&recordid=74154067&loginid=BH11163) | 1760 | | | 3.86% | | | 0.0365 | | 0.0594 | |
| GO:0006810 | transport | [66](http://sas.ebioservice.com/bioinfoplug_molnetgotree.showgene.do?acc=GO:0006810&name=transport&recordid=74154067&loginid=BH11163) | 1755 | | | 3.76% | | | 0.0597 | | 0.0831 | |
| GO:0045184 | establishment of protein localization | [6](http://sas.ebioservice.com/bioinfoplug_molnetgotree.showgene.do?acc=GO:0045184&name=establishment+of+protein+localization&recordid=74154067&loginid=BH11163) | 363 | | | 1.65% | | | 0.9654 | | 0.4133 | |
| GO:0051649 | establishment of localization in cell | [22](http://sas.ebioservice.com/bioinfoplug_molnetgotree.showgene.do?acc=GO:0051649&name=establishment+of+localization+in+cell&recordid=74154067&loginid=BH11163) | 494 | | | 4.45% | | | 0.0614 | | 0.0832 | |
| GO:0051656 | establishment of organelle localization | [2](http://sas.ebioservice.com/bioinfoplug_molnetgotree.showgene.do?acc=GO:0051656&name=establishment+of+organelle+localization&recordid=74154067&loginid=BH11163) | 11 | | | 18.18% | | | 0.0558 | | 0.0808 | |
| GO:0051704 | multi-organism process | [19](http://sas.ebioservice.com/bioinfoplug_molnetgotree.showgene.do?acc=GO:0051704&name=multi-organism+process&recordid=74154067&loginid=BH11163) | 691 | | | 2.75% | | | 0.7178 | | 0.3434 | |
| GO:0009856 | pollination | [3](http://sas.ebioservice.com/bioinfoplug_molnetgotree.showgene.do?acc=GO:0009856&name=pollination&recordid=74154067&loginid=BH11163) | 127 | | | 2.36% | | | 0.7485 | | 0.3517 | |
| GO:0051707 | response to other organism | [16](http://sas.ebioservice.com/bioinfoplug_molnetgotree.showgene.do?acc=GO:0051707&name=response+to+other+organism&recordid=74154067&loginid=BH11163) | 539 | | | 2.97% | | | 0.591 | | 0.3012 | |
| GO:0065007 | biological regulation | [127](http://sas.ebioservice.com/bioinfoplug_molnetgotree.showgene.do?acc=GO:0065007&name=biological+regulation&recordid=74154067&loginid=BH11163) | 3747 | | | 3.39% | | | 0.1365 | | 0.1345 | |
| GO:0050789 | regulation of biological process | [113](http://sas.ebioservice.com/bioinfoplug_molnetgotree.showgene.do?acc=GO:0050789&name=regulation+of+biological+process&recordid=74154067&loginid=BH11163) | 3304 | | | 3.42% | | | 0.1316 | | 0.1345 | |
| GO:0065008 | regulation of biological quality | [26](http://sas.ebioservice.com/bioinfoplug_molnetgotree.showgene.do?acc=GO:0065008&name=regulation+of+biological+quality&recordid=74154067&loginid=BH11163) | 610 | | | 4.26% | | | 0.0673 | | 0.0868 | |
| GO:0065009 | regulation of molecular function | [6](http://sas.ebioservice.com/bioinfoplug_molnetgotree.showgene.do?acc=GO:0065009&name=regulation+of+molecular+function&recordid=74154067&loginid=BH11163) | 143 | | | 4.2% | | | 0.2861 | | 0.2043 | |
| GO:0048518 | positive regulation of biological process | [13](http://sas.ebioservice.com/bioinfoplug_molnetgotree.showgene.do?acc=GO:0048518&name=positive+regulation+of+biological+process&recordid=74154067&loginid=BH11163) | 208 | | | 6.25% | | | 0.0159 | | 0.035 | |
| GO:0002684 | positive regulation of immune system process | [2](http://sas.ebioservice.com/bioinfoplug_molnetgotree.showgene.do?acc=GO:0002684&name=positive+regulation+of+immune+system+process&recordid=74154067&loginid=BH11163) | 29 | | | 6.9% | | | 0.2362 | | 0.1877 | |
| GO:0009893 | positive regulation of metabolic process | [4](http://sas.ebioservice.com/bioinfoplug_molnetgotree.showgene.do?acc=GO:0009893&name=positive+regulation+of+metabolic+process&recordid=74154067&loginid=BH11163) | 80 | | | 5.0% | | | 0.2419 | | 0.1895 | |
| GO:0045927 | positive regulation of growth | [1](http://sas.ebioservice.com/bioinfoplug_molnetgotree.showgene.do?acc=GO:0045927&name=positive+regulation+of+growth&recordid=74154067&loginid=BH11163) | 4 | | | 25.0% | | | 0.1405 | | 0.1345 | |
| GO:0048522 | positive regulation of cellular process | [8](http://sas.ebioservice.com/bioinfoplug_molnetgotree.showgene.do?acc=GO:0048522&name=positive+regulation+of+cellular+process&recordid=74154067&loginid=BH11163) | 132 | | | 6.06% | | | 0.0591 | | 0.0831 | |
| GO:0048584 | positive regulation of response to stimulus | [3](http://sas.ebioservice.com/bioinfoplug_molnetgotree.showgene.do?acc=GO:0048584&name=positive+regulation+of+response+to+stimulus&recordid=74154067&loginid=BH11163) | 57 | | | 5.26% | | | 0.2658 | | 0.1971 | |
| GO:0051094 | positive regulation of developmental process | [2](http://sas.ebioservice.com/bioinfoplug_molnetgotree.showgene.do?acc=GO:0051094&name=positive+regulation+of+developmental+process&recordid=74154067&loginid=BH11163) | 50 | | | 4.0% | | | 0.4621 | | 0.2669 | |
| GO:0048519 | negative regulation of biological process | [12](http://sas.ebioservice.com/bioinfoplug_molnetgotree.showgene.do?acc=GO:0048519&name=negative+regulation+of+biological+process&recordid=74154067&loginid=BH11163) | 346 | | | 3.47% | | | 0.3818 | | 0.2417 | |
| GO:0009892 | negative regulation of metabolic process | [9](http://sas.ebioservice.com/bioinfoplug_molnetgotree.showgene.do?acc=GO:0009892&name=negative+regulation+of+metabolic+process&recordid=74154067&loginid=BH11163) | 190 | | | 4.74% | | | 0.1425 | | 0.1345 | |
| GO:0045926 | negative regulation of growth | [1](http://sas.ebioservice.com/bioinfoplug_molnetgotree.showgene.do?acc=GO:0045926&name=negative+regulation+of+growth&recordid=74154067&loginid=BH11163) | 9 | | | 11.11% | | | 0.2613 | | 0.1971 | |
| GO:0048523 | negative regulation of cellular process | [9](http://sas.ebioservice.com/bioinfoplug_molnetgotree.showgene.do?acc=GO:0048523&name=negative+regulation+of+cellular+process&recordid=74154067&loginid=BH11163) | 181 | | | 4.97% | | | 0.1166 | | 0.1245 | |
| GO:0048585 | negative regulation of response to stimulus | [2](http://sas.ebioservice.com/bioinfoplug_molnetgotree.showgene.do?acc=GO:0048585&name=negative+regulation+of+response+to+stimulus&recordid=74154067&loginid=BH11163) | 64 | | | 3.13% | | | 0.5898 | | 0.3012 | |
| GO:0051093 | negative regulation of developmental process | [3](http://sas.ebioservice.com/bioinfoplug_molnetgotree.showgene.do?acc=GO:0051093&name=negative+regulation+of+developmental+process&recordid=74154067&loginid=BH11163) | 92 | | | 3.26% | | | 0.5419 | | 0.2883 | |
| GO:0051241 | negative regulation of multicellular organismal process | [1](http://sas.ebioservice.com/bioinfoplug_molnetgotree.showgene.do?acc=GO:0051241&name=negative+regulation+of+multicellular+organismal+process&recordid=74154067&loginid=BH11163) | 9 | | | 11.11% | | | 0.2613 | | 0.1971 | |
| GO:0050789 | regulation of biological process | [113](http://sas.ebioservice.com/bioinfoplug_molnetgotree.showgene.do?acc=GO:0050789&name=regulation+of+biological+process&recordid=74154067&loginid=BH11163) | 3304 | | | 3.42% | | | 0.1316 | | 0.1345 | |
| GO:0002682 | regulation of immune system process | [3](http://sas.ebioservice.com/bioinfoplug_molnetgotree.showgene.do?acc=GO:0002682&name=regulation+of+immune+system+process&recordid=74154067&loginid=BH11163) | 37 | | | 8.11% | | | 0.1163 | | 0.1245 | |
| GO:0019222 | regulation of metabolic process | [60](http://sas.ebioservice.com/bioinfoplug_molnetgotree.showgene.do?acc=GO:0019222&name=regulation+of+metabolic+process&recordid=74154067&loginid=BH11163) | 1928 | | | 3.11% | | | 0.4822 | | 0.2726 | |
| GO:0032879 | regulation of localization | [1](http://sas.ebioservice.com/bioinfoplug_molnetgotree.showgene.do?acc=GO:0032879&name=regulation+of+localization&recordid=74154067&loginid=BH11163) | 20 | | | 5.0% | | | 0.4707 | | 0.269 | |
| GO:0040008 | regulation of growth | [2](http://sas.ebioservice.com/bioinfoplug_molnetgotree.showgene.do?acc=GO:0040008&name=regulation+of+growth&recordid=74154067&loginid=BH11163) | 48 | | | 4.17% | | | 0.442 | | 0.264 | |
| GO:0048518 | positive regulation of biological process | [13](http://sas.ebioservice.com/bioinfoplug_molnetgotree.showgene.do?acc=GO:0048518&name=positive+regulation+of+biological+process&recordid=74154067&loginid=BH11163) | 208 | | | 6.25% | | | 0.0159 | | 0.035 | |
| GO:0048519 | negative regulation of biological process | [12](http://sas.ebioservice.com/bioinfoplug_molnetgotree.showgene.do?acc=GO:0048519&name=negative+regulation+of+biological+process&recordid=74154067&loginid=BH11163) | 346 | | | 3.47% | | | 0.3818 | | 0.2417 | |
| GO:0048583 | regulation of response to stimulus | [7](http://sas.ebioservice.com/bioinfoplug_molnetgotree.showgene.do?acc=GO:0048583&name=regulation+of+response+to+stimulus&recordid=74154067&loginid=BH11163) | 150 | | | 4.67% | | | 0.19 | | 0.1571 | |
| GO:0050793 | regulation of developmental process | [12](http://sas.ebioservice.com/bioinfoplug_molnetgotree.showgene.do?acc=GO:0050793&name=regulation+of+developmental+process&recordid=74154067&loginid=BH11163) | 271 | | | 4.43% | | | 0.1423 | | 0.1345 | |
| GO:0050794 | regulation of cellular process | [102](http://sas.ebioservice.com/bioinfoplug_molnetgotree.showgene.do?acc=GO:0050794&name=regulation+of+cellular+process&recordid=74154067&loginid=BH11163) | 3034 | | | 3.36% | | | 0.1895 | | 0.1571 | |
| GO:0051239 | regulation of multicellular organismal process | [10](http://sas.ebioservice.com/bioinfoplug_molnetgotree.showgene.do?acc=GO:0051239&name=regulation+of+multicellular+organismal+process&recordid=74154067&loginid=BH11163) | 202 | | | 4.95% | | | 0.1041 | | 0.1171 | |
| The genes down-regulated in response to NaCl stress | | | | | | | | | | | | |
| GO:0003674 | molecular_  function | [894](http://sas.ebioservice.com/bioinfoplug_molnetgotree.showgene.do?acc=GO:0003674&name=molecular_function&recordid=74154068&loginid=BH11163) | | 29448 | | | 3.04% | | 0.9229 | 0.6757 | | |
| GO:0003824 | catalytic activity | [267](http://sas.ebioservice.com/bioinfoplug_molnetgotree.showgene.do?acc=GO:0003824&name=catalytic+activity&recordid=74154068&loginid=BH11163) | | 8137 | | | 3.28% | | 0.1208 | 0.2047 | | |
| GO:0004133 | glycogen debranching enzyme activity | [1](http://sas.ebioservice.com/bioinfoplug_molnetgotree.showgene.do?acc=GO:0004133&name=glycogen+debranching+enzyme+activity&recordid=74154068&loginid=BH11163) | | 5 | | | 20.0% | | 0.1662 | 0.2588 | | |
| GO:0009975 | cyclase activity | [2](http://sas.ebioservice.com/bioinfoplug_molnetgotree.showgene.do?acc=GO:0009975&name=cyclase+activity&recordid=74154068&loginid=BH11163) | | 15 | | | 13.33% | | 0.09 | 0.1711 | | |
| GO:0016491 | oxidoreductase activity | [48](http://sas.ebioservice.com/bioinfoplug_molnetgotree.showgene.do?acc=GO:0016491&name=oxidoreductase+activity&recordid=74154068&loginid=BH11163) | | 1420 | | | 3.38% | | 0.2755 | 0.3724 | | |
| GO:0016740 | transferase activity | [108](http://sas.ebioservice.com/bioinfoplug_molnetgotree.showgene.do?acc=GO:0016740&name=transferase+activity&recordid=74154068&loginid=BH11163) | | 2722 | | | 3.97% | | 0.0049 | 0.0199 | | |
| GO:0016787 | hydrolase activity | [83](http://sas.ebioservice.com/bioinfoplug_molnetgotree.showgene.do?acc=GO:0016787&name=hydrolase+activity&recordid=74154068&loginid=BH11163) | | 2933 | | | 2.83% | | 0.7984 | 0.6757 | | |
| GO:0016829 | lyase activity | [8](http://sas.ebioservice.com/bioinfoplug_molnetgotree.showgene.do?acc=GO:0016829&name=lyase+activity&recordid=74154068&loginid=BH11163) | | 333 | | | 2.4% | | 0.8005 | 0.6757 | | |
| GO:0016853 | isomerase activity | [5](http://sas.ebioservice.com/bioinfoplug_molnetgotree.showgene.do?acc=GO:0016853&name=isomerase+activity&recordid=74154068&loginid=BH11163) | | 220 | | | 2.27% | | 0.8043 | 0.6757 | | |
| GO:0016874 | ligase activity | [19](http://sas.ebioservice.com/bioinfoplug_molnetgotree.showgene.do?acc=GO:0016874&name=ligase+activity&recordid=74154068&loginid=BH11163) | | 453 | | | 4.19% | | 0.1169 | 0.2035 | | |
| GO:0005198 | structural molecule activity | [2](http://sas.ebioservice.com/bioinfoplug_molnetgotree.showgene.do?acc=GO:0005198&name=structural+molecule+activity&recordid=74154068&loginid=BH11163) | | 501 | | | 0.4% | | 1.0 | 0.6757 | | |
| GO:0003735 | structural constituent of ribosome | [1](http://sas.ebioservice.com/bioinfoplug_molnetgotree.showgene.do?acc=GO:0003735&name=structural+constituent+of+ribosome&recordid=74154068&loginid=BH11163) | | 361 | | | 0.28% | | 1.0 | 0.6757 | | |
| GO:0030527 | structural constituent of chromatin | [1](http://sas.ebioservice.com/bioinfoplug_molnetgotree.showgene.do?acc=GO:0030527&name=structural+constituent+of+chromatin&recordid=74154068&loginid=BH11163) | | 5 | | | 20.0% | | 0.1662 | 0.2588 | | |
| GO:0005215 | transporter activity | [39](http://sas.ebioservice.com/bioinfoplug_molnetgotree.showgene.do?acc=GO:0005215&name=transporter+activity&recordid=74154068&loginid=BH11163) | | 1222 | | | 3.19% | | 0.4316 | 0.4941 | | |
| GO:0015238 | drug transporter activity | [5](http://sas.ebioservice.com/bioinfoplug_molnetgotree.showgene.do?acc=GO:0015238&name=drug+transporter+activity&recordid=74154068&loginid=BH11163) | | 67 | | | 7.46% | | 0.0639 | 0.1374 | | |
| GO:0022857 | transmembrane transporter activity | [35](http://sas.ebioservice.com/bioinfoplug_molnetgotree.showgene.do?acc=GO:0022857&name=transmembrane+transporter+activity&recordid=74154068&loginid=BH11163) | | 930 | | | 3.76% | | 0.1373 | 0.2223 | | |
| GO:0022892 | substrate-specific transporter activity | [30](http://sas.ebioservice.com/bioinfoplug_molnetgotree.showgene.do?acc=GO:0022892&name=substrate-specific+transporter+activity&recordid=74154068&loginid=BH11163) | | 865 | | | 3.47% | | 0.2817 | 0.3776 | | |
| GO:0005488 | binding | [388](http://sas.ebioservice.com/bioinfoplug_molnetgotree.showgene.do?acc=GO:0005488&name=binding&recordid=74154068&loginid=BH11163) | | 10009 | | | 3.88% | | 0.0 | 0.0 | | |
| GO:0000166 | nucleotide binding | [74](http://sas.ebioservice.com/bioinfoplug_molnetgotree.showgene.do?acc=GO:0000166&name=nucleotide+binding&recordid=74154068&loginid=BH11163) | | 2177 | | | 3.4% | | 0.206 | 0.3035 | | |
| GO:0001871 | pattern binding | [1](http://sas.ebioservice.com/bioinfoplug_molnetgotree.showgene.do?acc=GO:0001871&name=pattern+binding&recordid=74154068&loginid=BH11163) | | 17 | | | 5.88% | | 0.4203 | 0.491 | | |
| GO:0003676 | nucleic acid binding | [141](http://sas.ebioservice.com/bioinfoplug_molnetgotree.showgene.do?acc=GO:0003676&name=nucleic+acid+binding&recordid=74154068&loginid=BH11163) | | 3939 | | | 3.58% | | 0.0343 | 0.0863 | | |
| GO:0003682 | chromatin binding | [1](http://sas.ebioservice.com/bioinfoplug_molnetgotree.showgene.do?acc=GO:0003682&name=chromatin+binding&recordid=74154068&loginid=BH11163) | | 38 | | | 2.63% | | 0.6933 | 0.6757 | | |
| GO:0005515 | protein binding | [119](http://sas.ebioservice.com/bioinfoplug_molnetgotree.showgene.do?acc=GO:0005515&name=protein+binding&recordid=74154068&loginid=BH11163) | | 2640 | | | 4.51% | | 0.0 | 2.0E-4 | | |
| GO:0008289 | lipid binding | [7](http://sas.ebioservice.com/bioinfoplug_molnetgotree.showgene.do?acc=GO:0008289&name=lipid+binding&recordid=74154068&loginid=BH11163) | | 267 | | | 2.62% | | 0.7124 | 0.6757 | | |
| GO:0019825 | oxygen binding | [13](http://sas.ebioservice.com/bioinfoplug_molnetgotree.showgene.do?acc=GO:0019825&name=oxygen+binding&recordid=74154068&loginid=BH11163) | | 231 | | | 5.63% | | 0.0323 | 0.0826 | | |
| GO:0019842 | vitamin binding | [2](http://sas.ebioservice.com/bioinfoplug_molnetgotree.showgene.do?acc=GO:0019842&name=vitamin+binding&recordid=74154068&loginid=BH11163) | | 63 | | | 3.17% | | 0.5815 | 0.6135 | | |
| GO:0030246 | carbohydrate binding | [8](http://sas.ebioservice.com/bioinfoplug_molnetgotree.showgene.do?acc=GO:0030246&name=carbohydrate+binding&recordid=74154068&loginid=BH11163) | | 153 | | | 5.23% | | 0.1099 | 0.1997 | | |
| GO:0043167 | ion binding | [94](http://sas.ebioservice.com/bioinfoplug_molnetgotree.showgene.do?acc=GO:0043167&name=ion+binding&recordid=74154068&loginid=BH11163) | | 2255 | | | 4.17% | | 0.0023 | 0.0099 | | |
| GO:0043176 | amine binding | [1](http://sas.ebioservice.com/bioinfoplug_molnetgotree.showgene.do?acc=GO:0043176&name=amine+binding&recordid=74154068&loginid=BH11163) | | 23 | | | 4.35% | | 0.5167 | 0.5596 | | |
| GO:0046906 | tetrapyrrole binding | [16](http://sas.ebioservice.com/bioinfoplug_molnetgotree.showgene.do?acc=GO:0046906&name=tetrapyrrole+binding&recordid=74154068&loginid=BH11163) | | 320 | | | 5.0% | | 0.0461 | 0.1056 | | |
| GO:0048037 | cofactor binding | [7](http://sas.ebioservice.com/bioinfoplug_molnetgotree.showgene.do?acc=GO:0048037&name=cofactor+binding&recordid=74154068&loginid=BH11163) | | 264 | | | 2.65% | | 0.7017 | 0.6757 | | |
| GO:0009055 | electron carrier activity | [18](http://sas.ebioservice.com/bioinfoplug_molnetgotree.showgene.do?acc=GO:0009055&name=electron+carrier+activity&recordid=74154068&loginid=BH11163) | | 495 | | | 3.64% | | 0.274 | 0.3724 | | |
| GO:0016209 | antioxidant activity | [7](http://sas.ebioservice.com/bioinfoplug_molnetgotree.showgene.do?acc=GO:0016209&name=antioxidant+activity&recordid=74154068&loginid=BH11163) | | 126 | | | 5.56% | | 0.1038 | 0.195 | | |
| GO:0004601 | peroxidase activity | [7](http://sas.ebioservice.com/bioinfoplug_molnetgotree.showgene.do?acc=GO:0004601&name=peroxidase+activity&recordid=74154068&loginid=BH11163) | | 109 | | | 6.42% | | 0.0591 | 0.1295 | | |
| GO:0030234 | enzyme regulator activity | [7](http://sas.ebioservice.com/bioinfoplug_molnetgotree.showgene.do?acc=GO:0030234&name=enzyme+regulator+activity&recordid=74154068&loginid=BH11163) | | 342 | | | 2.05% | | 0.8985 | 0.6757 | | |
| GO:0004857 | enzyme inhibitor activity | [4](http://sas.ebioservice.com/bioinfoplug_molnetgotree.showgene.do?acc=GO:0004857&name=enzyme+inhibitor+activity&recordid=74154068&loginid=BH11163) | | 174 | | | 2.3% | | 0.781 | 0.6757 | | |
| GO:0008047 | enzyme activator activity | [2](http://sas.ebioservice.com/bioinfoplug_molnetgotree.showgene.do?acc=GO:0008047&name=enzyme+activator+activity&recordid=74154068&loginid=BH11163) | | 74 | | | 2.7% | | 0.6664 | 0.6694 | | |
| GO:0019207 | kinase regulator activity | [1](http://sas.ebioservice.com/bioinfoplug_molnetgotree.showgene.do?acc=GO:0019207&name=kinase+regulator+activity&recordid=74154068&loginid=BH11163) | | 42 | | | 2.38% | | 0.7283 | 0.6757 | | |
| GO:0019208 | phosphatase regulator activity | [1](http://sas.ebioservice.com/bioinfoplug_molnetgotree.showgene.do?acc=GO:0019208&name=phosphatase+regulator+activity&recordid=74154068&loginid=BH11163) | | 18 | | | 5.56% | | 0.4377 | 0.4956 | | |
| GO:0060589 | nucleoside-triphosphatase regulator activity | [1](http://sas.ebioservice.com/bioinfoplug_molnetgotree.showgene.do?acc=GO:0060589&name=nucleoside-triphosphatase+regulator+activity&recordid=74154068&loginid=BH11163) | | 90 | | | 1.11% | | 0.9367 | 0.6757 | | |
| GO:0030528 | transcription regulator activity | [119](http://sas.ebioservice.com/bioinfoplug_molnetgotree.showgene.do?acc=GO:0030528&name=transcription+regulator+activity&recordid=74154068&loginid=BH11163) | | 1885 | | | 6.31% | | 0.0 | 0.0 | | |
| GO:0000156 | two-component response regulator activity | [2](http://sas.ebioservice.com/bioinfoplug_molnetgotree.showgene.do?acc=GO:0000156&name=two-component+response+regulator+activity&recordid=74154068&loginid=BH11163) | | 35 | | | 5.71% | | 0.303 | 0.3963 | | |
| GO:0003700 | transcription factor activity | [116](http://sas.ebioservice.com/bioinfoplug_molnetgotree.showgene.do?acc=GO:0003700&name=transcription+factor+activity&recordid=74154068&loginid=BH11163) | | 1677 | | | 6.92% | | 0.0 | 0.0 | | |
| GO:0016563 | transcription activator activity | [11](http://sas.ebioservice.com/bioinfoplug_molnetgotree.showgene.do?acc=GO:0016563&name=transcription+activator+activity&recordid=74154068&loginid=BH11163) | | 134 | | | 8.21% | | 0.0043 | 0.0179 | | |
| GO:0016564 | transcription repressor activity | [6](http://sas.ebioservice.com/bioinfoplug_molnetgotree.showgene.do?acc=GO:0016564&name=transcription+repressor+activity&recordid=74154068&loginid=BH11163) | | 48 | | | 12.5% | | 0.0053 | 0.021 | | |
| GO:0031386 | protein tag | [1](http://sas.ebioservice.com/bioinfoplug_molnetgotree.showgene.do?acc=GO:0031386&name=protein+tag&recordid=74154068&loginid=BH11163) | | 4 | | | 25.0% | | 0.1405 | 0.2253 | | |
| GO:0045182 | translation regulator activity | [1](http://sas.ebioservice.com/bioinfoplug_molnetgotree.showgene.do?acc=GO:0045182&name=translation+regulator+activity&recordid=74154068&loginid=BH11163) | | 140 | | | 0.71% | | 0.9862 | 0.6757 | | |
| GO:0008135 | translation factor activity, nucleic acid binding | [1](http://sas.ebioservice.com/bioinfoplug_molnetgotree.showgene.do?acc=GO:0008135&name=translation+factor+activity%2C+nucleic+acid+binding&recordid=74154068&loginid=BH11163) | | 139 | | | 0.72% | | 0.9857 | 0.6757 | | |
| GO:0045735 | nutrient reservoir activity | [4](http://sas.ebioservice.com/bioinfoplug_molnetgotree.showgene.do?acc=GO:0045735&name=nutrient+reservoir+activity&recordid=74154068&loginid=BH11163) | | 58 | | | 6.9% | | 0.1137 | 0.2035 | | |
| GO:0060089 | molecular transducer activity | [21](http://sas.ebioservice.com/bioinfoplug_molnetgotree.showgene.do?acc=GO:0060089&name=molecular+transducer+activity&recordid=74154068&loginid=BH11163) | | 375 | | | 5.6% | | 0.0085 | 0.0309 | | |
| GO:0004871 | signal transducer activity | [21](http://sas.ebioservice.com/bioinfoplug_molnetgotree.showgene.do?acc=GO:0004871&name=signal+transducer+activity&recordid=74154068&loginid=BH11163) | | 375 | | | 5.6% | | 0.0085 | 0.0309 | | |
| GO:0005575 | cellular_component | [817](http://sas.ebioservice.com/bioinfoplug_molnetgotree.showgene.do?acc=GO:0005575&name=cellular_component&recordid=74154068&loginid=BH11163) | | 28657 | | | 2.85% | | 1.0 | 0.6757 | | |
| GO:0005576 | extracellular region | [20](http://sas.ebioservice.com/bioinfoplug_molnetgotree.showgene.do?acc=GO:0005576&name=extracellular+region&recordid=74154068&loginid=BH11163) | | 438 | | | 4.57% | | 0.0594 | 0.1295 | | |
| GO:0048046 | apoplast | [14](http://sas.ebioservice.com/bioinfoplug_molnetgotree.showgene.do?acc=GO:0048046&name=apoplast&recordid=74154068&loginid=BH11163) | | 329 | | | 4.26% | | 0.1487 | 0.2361 | | |
| GO:0044421 | extracellular region part | [3](http://sas.ebioservice.com/bioinfoplug_molnetgotree.showgene.do?acc=GO:0044421&name=extracellular+region+part&recordid=74154068&loginid=BH11163) | | 23 | | | 13.04% | | 0.0413 | 0.0966 | | |
| GO:0005623 | cell | [533](http://sas.ebioservice.com/bioinfoplug_molnetgotree.showgene.do?acc=GO:0005623&name=cell&recordid=74154068&loginid=BH11163) | | 15202 | | | 3.51% | | 0.0 | 1.0E-4 | | |
| GO:0044464 | cell part | [533](http://sas.ebioservice.com/bioinfoplug_molnetgotree.showgene.do?acc=GO:0044464&name=cell+part&recordid=74154068&loginid=BH11163) | | 15202 | | | 3.51% | | 0.0 | 1.0E-4 | | |
| GO:0031974 | membrane-enclosed lumen | [12](http://sas.ebioservice.com/bioinfoplug_molnetgotree.showgene.do?acc=GO:0031974&name=membrane-enclosed+lumen&recordid=74154068&loginid=BH11163) | | 551 | | | 2.18% | | 0.913 | 0.6757 | | |
| GO:0043233 | organelle lumen | [12](http://sas.ebioservice.com/bioinfoplug_molnetgotree.showgene.do?acc=GO:0043233&name=organelle+lumen&recordid=74154068&loginid=BH11163) | | 545 | | | 2.2% | | 0.9061 | 0.6757 | | |
| GO:0031975 | envelope | [5](http://sas.ebioservice.com/bioinfoplug_molnetgotree.showgene.do?acc=GO:0031975&name=envelope&recordid=74154068&loginid=BH11163) | | 672 | | | 0.74% | | 1.0 | 0.6757 | | |
| GO:0031967 | organelle envelope | [5](http://sas.ebioservice.com/bioinfoplug_molnetgotree.showgene.do?acc=GO:0031967&name=organelle+envelope&recordid=74154068&loginid=BH11163) | | 672 | | | 0.74% | | 1.0 | 0.6757 | | |
| GO:0032991 | macromolecular complex | [37](http://sas.ebioservice.com/bioinfoplug_molnetgotree.showgene.do?acc=GO:0032991&name=macromolecular+complex&recordid=74154068&loginid=BH11163) | | 1731 | | | 2.14% | | 0.9927 | 0.6757 | | |
| GO:0030529 | ribonucleoprotein complex | [10](http://sas.ebioservice.com/bioinfoplug_molnetgotree.showgene.do?acc=GO:0030529&name=ribonucleoprotein+complex&recordid=74154068&loginid=BH11163) | | 579 | | | 1.73% | | 0.9827 | 0.6757 | | |
| GO:0032993 | protein-DNA complex | [1](http://sas.ebioservice.com/bioinfoplug_molnetgotree.showgene.do?acc=GO:0032993&name=protein-DNA+complex&recordid=74154068&loginid=BH11163) | | 48 | | | 2.08% | | 0.7735 | 0.6757 | | |
| GO:0043234 | protein complex | [26](http://sas.ebioservice.com/bioinfoplug_molnetgotree.showgene.do?acc=GO:0043234&name=protein+complex&recordid=74154068&loginid=BH11163) | | 1124 | | | 2.31% | | 0.9449 | 0.6757 | | |
| GO:0043226 | organelle | [229](http://sas.ebioservice.com/bioinfoplug_molnetgotree.showgene.do?acc=GO:0043226&name=organelle&recordid=74154068&loginid=BH11163) | | 7608 | | | 3.01% | | 0.655 | 0.6652 | | |
| GO:0043227 | membrane-bounded organelle | [224](http://sas.ebioservice.com/bioinfoplug_molnetgotree.showgene.do?acc=GO:0043227&name=membrane-bounded+organelle&recordid=74154068&loginid=BH11163) | | 7214 | | | 3.11% | | 0.4482 | 0.4956 | | |
| GO:0043228 | non-membrane-bounded organelle | [11](http://sas.ebioservice.com/bioinfoplug_molnetgotree.showgene.do?acc=GO:0043228&name=non-membrane-bounded+organelle&recordid=74154068&loginid=BH11163) | | 963 | | | 1.14% | | 1.0 | 0.6757 | | |
| GO:0043229 | intracellular organelle | [229](http://sas.ebioservice.com/bioinfoplug_molnetgotree.showgene.do?acc=GO:0043229&name=intracellular+organelle&recordid=74154068&loginid=BH11163) | | 7607 | | | 3.01% | | 0.6541 | 0.6652 | | |
| GO:0044422 | organelle part | [38](http://sas.ebioservice.com/bioinfoplug_molnetgotree.showgene.do?acc=GO:0044422&name=organelle+part&recordid=74154068&loginid=BH11163) | | 2358 | | | 1.61% | | 1.0 | 0.6757 | | |
| GO:0044421 | extracellular region part | [3](http://sas.ebioservice.com/bioinfoplug_molnetgotree.showgene.do?acc=GO:0044421&name=extracellular+region+part&recordid=74154068&loginid=BH11163) | | 23 | | | 13.04% | | 0.0413 | 0.0966 | | |
| GO:0005615 | extracellular space | [1](http://sas.ebioservice.com/bioinfoplug_molnetgotree.showgene.do?acc=GO:0005615&name=extracellular+space&recordid=74154068&loginid=BH11163) | | 8 | | | 12.5% | | 0.2386 | 0.3393 | | |
| GO:0031012 | extracellular matrix | [2](http://sas.ebioservice.com/bioinfoplug_molnetgotree.showgene.do?acc=GO:0031012&name=extracellular+matrix&recordid=74154068&loginid=BH11163) | | 15 | | | 13.33% | | 0.09 | 0.1711 | | |
| GO:0044422 | organelle part | [38](http://sas.ebioservice.com/bioinfoplug_molnetgotree.showgene.do?acc=GO:0044422&name=organelle+part&recordid=74154068&loginid=BH11163) | | 2358 | | | 1.61% | | 1.0 | 0.6757 | | |
| GO:0031090 | organelle membrane | [9](http://sas.ebioservice.com/bioinfoplug_molnetgotree.showgene.do?acc=GO:0031090&name=organelle+membrane&recordid=74154068&loginid=BH11163) | | 743 | | | 1.21% | | 0.9997 | 0.6757 | | |
| GO:0043233 | organelle lumen | [12](http://sas.ebioservice.com/bioinfoplug_molnetgotree.showgene.do?acc=GO:0043233&name=organelle+lumen&recordid=74154068&loginid=BH11163) | | 545 | | | 2.2% | | 0.9061 | 0.6757 | | |
| GO:0044446 | intracellular organelle part | [38](http://sas.ebioservice.com/bioinfoplug_molnetgotree.showgene.do?acc=GO:0044446&name=intracellular+organelle+part&recordid=74154068&loginid=BH11163) | | 2357 | | | 1.61% | | 1.0 | 0.6757 | | |
| GO:0044464 | cell part | [533](http://sas.ebioservice.com/bioinfoplug_molnetgotree.showgene.do?acc=GO:0044464&name=cell+part&recordid=74154068&loginid=BH11163) | | 15202 | | | 3.51% | | 0.0 | 1.0E-4 | | |
| GO:0000267 | cell fraction | [1](http://sas.ebioservice.com/bioinfoplug_molnetgotree.showgene.do?acc=GO:0000267&name=cell+fraction&recordid=74154068&loginid=BH11163) | | 37 | | | 2.7% | | 0.6839 | 0.6737 | | |
| GO:0005622 | intracellular | [270](http://sas.ebioservice.com/bioinfoplug_molnetgotree.showgene.do?acc=GO:0005622&name=intracellular&recordid=74154068&loginid=BH11163) | | 8856 | | | 3.05% | | 0.5774 | 0.6132 | | |
| GO:0008287 | protein serine/threonine phosphatase complex | [5](http://sas.ebioservice.com/bioinfoplug_molnetgotree.showgene.do?acc=GO:0008287&name=protein+serine%2Fthreonine+phosphatase+complex&recordid=74154068&loginid=BH11163) | | 58 | | | 8.62% | | 0.0397 | 0.0966 | | |
| GO:0012505 | endomembrane system | [144](http://sas.ebioservice.com/bioinfoplug_molnetgotree.showgene.do?acc=GO:0012505&name=endomembrane+system&recordid=74154068&loginid=BH11163) | | 4046 | | | 3.56% | | 0.038 | 0.0941 | | |
| GO:0016020 | membrane | [163](http://sas.ebioservice.com/bioinfoplug_molnetgotree.showgene.do?acc=GO:0016020&name=membrane&recordid=74154068&loginid=BH11163) | | 4532 | | | 3.6% | | 0.0203 | 0.0594 | | |
| GO:0030312 | external encapsulating structure | [29](http://sas.ebioservice.com/bioinfoplug_molnetgotree.showgene.do?acc=GO:0030312&name=external+encapsulating+structure&recordid=74154068&loginid=BH11163) | | 553 | | | 5.24% | | 0.0054 | 0.021 | | |
| GO:0044424 | intracellular part | [252](http://sas.ebioservice.com/bioinfoplug_molnetgotree.showgene.do?acc=GO:0044424&name=intracellular+part&recordid=74154068&loginid=BH11163) | | 8479 | | | 2.97% | | 0.7442 | 0.6757 | | |
| GO:0044425 | membrane part | [55](http://sas.ebioservice.com/bioinfoplug_molnetgotree.showgene.do?acc=GO:0044425&name=membrane+part&recordid=74154068&loginid=BH11163) | | 1305 | | | 4.21% | | 0.0145 | 0.0484 | | |
| GO:0055044 | symplast | [1](http://sas.ebioservice.com/bioinfoplug_molnetgotree.showgene.do?acc=GO:0055044&name=symplast&recordid=74154068&loginid=BH11163) | | 15 | | | 6.67% | | 0.3841 | 0.4832 | | |
| GO:0009506 | plasmodesma | [1](http://sas.ebioservice.com/bioinfoplug_molnetgotree.showgene.do?acc=GO:0009506&name=plasmodesma&recordid=74154068&loginid=BH11163) | | 15 | | | 6.67% | | 0.3841 | 0.4832 | | |
| GO:0008150 | biological_process | [867](http://sas.ebioservice.com/bioinfoplug_molnetgotree.showgene.do?acc=GO:0008150&name=biological_process&recordid=74154068&loginid=BH11163) | | 28723 | | | 3.02% | | 0.9598 | 0.6757 | | |
| GO:0000003 | reproduction | [18](http://sas.ebioservice.com/bioinfoplug_molnetgotree.showgene.do?acc=GO:0000003&name=reproduction&recordid=74154068&loginid=BH11163) | | 937 | | | 1.92% | | 0.9876 | 0.6757 | | |
| GO:0019953 | sexual reproduction | [2](http://sas.ebioservice.com/bioinfoplug_molnetgotree.showgene.do?acc=GO:0019953&name=sexual+reproduction&recordid=74154068&loginid=BH11163) | | 60 | | | 3.33% | | 0.5558 | 0.594 | | |
| GO:0032504 | multicellular organism reproduction | [1](http://sas.ebioservice.com/bioinfoplug_molnetgotree.showgene.do?acc=GO:0032504&name=multicellular+organism+reproduction&recordid=74154068&loginid=BH11163) | | 34 | | | 2.94% | | 0.6538 | 0.6652 | | |
| GO:0022414 | reproductive process | [18](http://sas.ebioservice.com/bioinfoplug_molnetgotree.showgene.do?acc=GO:0022414&name=reproductive+process&recordid=74154068&loginid=BH11163) | | 920 | | | 1.96% | | 0.9844 | 0.6757 | | |
| GO:0002376 | immune system process | [37](http://sas.ebioservice.com/bioinfoplug_molnetgotree.showgene.do?acc=GO:0002376&name=immune+system+process&recordid=74154068&loginid=BH11163) | | 313 | | | 11.82% | | 0.0 | 0.0 | | |
| GO:0002252 | immune effector process | [4](http://sas.ebioservice.com/bioinfoplug_molnetgotree.showgene.do?acc=GO:0002252&name=immune+effector+process&recordid=74154068&loginid=BH11163) | | 25 | | | 16.0% | | 0.0103 | 0.0359 | | |
| GO:0002253 | activation of immune response | [4](http://sas.ebioservice.com/bioinfoplug_molnetgotree.showgene.do?acc=GO:0002253&name=activation+of+immune+response&recordid=74154068&loginid=BH11163) | | 29 | | | 13.79% | | 0.0162 | 0.0491 | | |
| GO:0006955 | immune response | [37](http://sas.ebioservice.com/bioinfoplug_molnetgotree.showgene.do?acc=GO:0006955&name=immune+response&recordid=74154068&loginid=BH11163) | | 312 | | | 11.86% | | 0.0 | 0.0 | | |
| GO:0002682 | regulation of immune system process | [6](http://sas.ebioservice.com/bioinfoplug_molnetgotree.showgene.do?acc=GO:0002682&name=regulation+of+immune+system+process&recordid=74154068&loginid=BH11163) | | 37 | | | 16.22% | | 0.0016 | 0.0073 | | |
| GO:0002684 | positive regulation of immune system process | [4](http://sas.ebioservice.com/bioinfoplug_molnetgotree.showgene.do?acc=GO:0002684&name=positive+regulation+of+immune+system+process&recordid=74154068&loginid=BH11163) | | 29 | | | 13.79% | | 0.0162 | 0.0491 | | |
| GO:0008152 | metabolic process | [307](http://sas.ebioservice.com/bioinfoplug_molnetgotree.showgene.do?acc=GO:0008152&name=metabolic+process&recordid=74154068&loginid=BH11163) | | 9176 | | | 3.35% | | 0.0465 | 0.1056 | | |
| GO:0006807 | nitrogen compound metabolic process | [11](http://sas.ebioservice.com/bioinfoplug_molnetgotree.showgene.do?acc=GO:0006807&name=nitrogen+compound+metabolic+process&recordid=74154068&loginid=BH11163) | | 451 | | | 2.44% | | 0.8154 | 0.6757 | | |
| GO:0009056 | catabolic process | [26](http://sas.ebioservice.com/bioinfoplug_molnetgotree.showgene.do?acc=GO:0009056&name=catabolic+process&recordid=74154068&loginid=BH11163) | | 686 | | | 3.79% | | 0.1703 | 0.2627 | | |
| GO:0009058 | biosynthetic process | [140](http://sas.ebioservice.com/bioinfoplug_molnetgotree.showgene.do?acc=GO:0009058&name=biosynthetic+process&recordid=74154068&loginid=BH11163) | | 4277 | | | 3.27% | | 0.2299 | 0.3297 | | |
| GO:0019748 | secondary metabolic process | [14](http://sas.ebioservice.com/bioinfoplug_molnetgotree.showgene.do?acc=GO:0019748&name=secondary+metabolic+process&recordid=74154068&loginid=BH11163) | | 383 | | | 3.66% | | 0.2995 | 0.395 | | |
| GO:0042440 | pigment metabolic process | [1](http://sas.ebioservice.com/bioinfoplug_molnetgotree.showgene.do?acc=GO:0042440&name=pigment+metabolic+process&recordid=74154068&loginid=BH11163) | | 110 | | | 0.91% | | 0.9655 | 0.6757 | | |
| GO:0042445 | hormone metabolic process | [7](http://sas.ebioservice.com/bioinfoplug_molnetgotree.showgene.do?acc=GO:0042445&name=hormone+metabolic+process&recordid=74154068&loginid=BH11163) | | 100 | | | 7.0% | | 0.0413 | 0.0966 | | |
| GO:0043170 | macromolecule metabolic process | [216](http://sas.ebioservice.com/bioinfoplug_molnetgotree.showgene.do?acc=GO:0043170&name=macromolecule+metabolic+process&recordid=74154068&loginid=BH11163) | | 6189 | | | 3.49% | | 0.024 | 0.0634 | | |
| GO:0044237 | cellular metabolic process | [262](http://sas.ebioservice.com/bioinfoplug_molnetgotree.showgene.do?acc=GO:0044237&name=cellular+metabolic+process&recordid=74154068&loginid=BH11163) | | 7840 | | | 3.34% | | 0.0692 | 0.145 | | |
| GO:0044238 | primary metabolic process | [262](http://sas.ebioservice.com/bioinfoplug_molnetgotree.showgene.do?acc=GO:0044238&name=primary+metabolic+process&recordid=74154068&loginid=BH11163) | | 7802 | | | 3.36% | | 0.0584 | 0.1295 | | |
| GO:0045730 | respiratory burst | [2](http://sas.ebioservice.com/bioinfoplug_molnetgotree.showgene.do?acc=GO:0045730&name=respiratory+burst&recordid=74154068&loginid=BH11163) | | 5 | | | 40.0% | | 0.0169 | 0.0502 | | |
| GO:0055114 | oxidation reduction | [4](http://sas.ebioservice.com/bioinfoplug_molnetgotree.showgene.do?acc=GO:0055114&name=oxidation+reduction&recordid=74154068&loginid=BH11163) | | 163 | | | 2.45% | | 0.7374 | 0.6757 | | |
| GO:0009892 | negative regulation of metabolic process | [4](http://sas.ebioservice.com/bioinfoplug_molnetgotree.showgene.do?acc=GO:0009892&name=negative+regulation+of+metabolic+process&recordid=74154068&loginid=BH11163) | | 190 | | | 2.11% | | 0.8338 | 0.6757 | | |
| GO:0009893 | positive regulation of metabolic process | [2](http://sas.ebioservice.com/bioinfoplug_molnetgotree.showgene.do?acc=GO:0009893&name=positive+regulation+of+metabolic+process&recordid=74154068&loginid=BH11163) | | 80 | | | 2.5% | | 0.7065 | 0.6757 | | |
| GO:0019222 | regulation of metabolic process | [105](http://sas.ebioservice.com/bioinfoplug_molnetgotree.showgene.do?acc=GO:0019222&name=regulation+of+metabolic+process&recordid=74154068&loginid=BH11163) | | 1928 | | | 5.45% | | 0.0 | 0.0 | | |
| GO:0009987 | cellular process | [357](http://sas.ebioservice.com/bioinfoplug_molnetgotree.showgene.do?acc=GO:0009987&name=cellular+process&recordid=74154068&loginid=BH11163) | | 10432 | | | 3.42% | | 0.0088 | 0.0312 | | |
| GO:0006413 | translational initiation | [1](http://sas.ebioservice.com/bioinfoplug_molnetgotree.showgene.do?acc=GO:0006413&name=translational+initiation&recordid=74154068&loginid=BH11163) | | 76 | | | 1.32% | | 0.9032 | 0.6757 | | |
| GO:0007049 | cell cycle | [2](http://sas.ebioservice.com/bioinfoplug_molnetgotree.showgene.do?acc=GO:0007049&name=cell+cycle&recordid=74154068&loginid=BH11163) | | 233 | | | 0.86% | | 0.9935 | 0.6757 | | |
| GO:0007154 | cell communication | [74](http://sas.ebioservice.com/bioinfoplug_molnetgotree.showgene.do?acc=GO:0007154&name=cell+communication&recordid=74154068&loginid=BH11163) | | 1267 | | | 5.84% | | 0.0 | 0.0 | | |
| GO:0007155 | cell adhesion | [1](http://sas.ebioservice.com/bioinfoplug_molnetgotree.showgene.do?acc=GO:0007155&name=cell+adhesion&recordid=74154068&loginid=BH11163) | | 17 | | | 5.88% | | 0.4203 | 0.491 | | |
| GO:0008219 | cell death | [18](http://sas.ebioservice.com/bioinfoplug_molnetgotree.showgene.do?acc=GO:0008219&name=cell+death&recordid=74154068&loginid=BH11163) | | 237 | | | 7.59% | | 7.0E-4 | 0.0035 | | |
| GO:0008283 | cell proliferation | [3](http://sas.ebioservice.com/bioinfoplug_molnetgotree.showgene.do?acc=GO:0008283&name=cell+proliferation&recordid=74154068&loginid=BH11163) | | 58 | | | 5.17% | | 0.2739 | 0.3724 | | |
| GO:0010118 | stomatal movement | [1](http://sas.ebioservice.com/bioinfoplug_molnetgotree.showgene.do?acc=GO:0010118&name=stomatal+movement&recordid=74154068&loginid=BH11163) | | 43 | | | 2.33% | | 0.7365 | 0.6757 | | |
| GO:0016043 | cellular component organization | [20](http://sas.ebioservice.com/bioinfoplug_molnetgotree.showgene.do?acc=GO:0016043&name=cellular+component+organization&recordid=74154068&loginid=BH11163) | | 966 | | | 2.07% | | 0.9757 | 0.6757 | | |
| GO:0016049 | cell growth | [10](http://sas.ebioservice.com/bioinfoplug_molnetgotree.showgene.do?acc=GO:0016049&name=cell+growth&recordid=74154068&loginid=BH11163) | | 229 | | | 4.37% | | 0.1792 | 0.2714 | | |
| GO:0016192 | vesicle-mediated transport | [8](http://sas.ebioservice.com/bioinfoplug_molnetgotree.showgene.do?acc=GO:0016192&name=vesicle-mediated+transport&recordid=74154068&loginid=BH11163) | | 237 | | | 3.38% | | 0.4481 | 0.4956 | | |
| GO:0016458 | gene silencing | [2](http://sas.ebioservice.com/bioinfoplug_molnetgotree.showgene.do?acc=GO:0016458&name=gene+silencing&recordid=74154068&loginid=BH11163) | | 116 | | | 1.72% | | 0.8707 | 0.6757 | | |
| GO:0019725 | cellular homeostasis | [9](http://sas.ebioservice.com/bioinfoplug_molnetgotree.showgene.do?acc=GO:0019725&name=cellular+homeostasis&recordid=74154068&loginid=BH11163) | | 183 | | | 4.92% | | 0.1221 | 0.2047 | | |
| GO:0022402 | cell cycle process | [1](http://sas.ebioservice.com/bioinfoplug_molnetgotree.showgene.do?acc=GO:0022402&name=cell+cycle+process&recordid=74154068&loginid=BH11163) | | 130 | | | 0.77% | | 0.9812 | 0.6757 | | |
| GO:0022406 | membrane docking | [2](http://sas.ebioservice.com/bioinfoplug_molnetgotree.showgene.do?acc=GO:0022406&name=membrane+docking&recordid=74154068&loginid=BH11163) | | 30 | | | 6.67% | | 0.2473 | 0.3486 | | |
| GO:0044085 | cellular component biogenesis | [6](http://sas.ebioservice.com/bioinfoplug_molnetgotree.showgene.do?acc=GO:0044085&name=cellular+component+biogenesis&recordid=74154068&loginid=BH11163) | | 552 | | | 1.09% | | 0.9993 | 0.6757 | | |
| GO:0044237 | cellular metabolic process | [262](http://sas.ebioservice.com/bioinfoplug_molnetgotree.showgene.do?acc=GO:0044237&name=cellular+metabolic+process&recordid=74154068&loginid=BH11163) | | 7840 | | | 3.34% | | 0.0692 | 0.145 | | |
| GO:0048468 | cell development | [3](http://sas.ebioservice.com/bioinfoplug_molnetgotree.showgene.do?acc=GO:0048468&name=cell+development&recordid=74154068&loginid=BH11163) | | 158 | | | 1.9% | | 0.8624 | 0.6757 | | |
| GO:0048469 | cell maturation | [3](http://sas.ebioservice.com/bioinfoplug_molnetgotree.showgene.do?acc=GO:0048469&name=cell+maturation&recordid=74154068&loginid=BH11163) | | 36 | | | 8.33% | | 0.1099 | 0.1997 | | |
| GO:0048869 | cellular developmental process | [7](http://sas.ebioservice.com/bioinfoplug_molnetgotree.showgene.do?acc=GO:0048869&name=cellular+developmental+process&recordid=74154068&loginid=BH11163) | | 324 | | | 2.16% | | 0.8669 | 0.6757 | | |
| GO:0051641 | cellular localization | [11](http://sas.ebioservice.com/bioinfoplug_molnetgotree.showgene.do?acc=GO:0051641&name=cellular+localization&recordid=74154068&loginid=BH11163) | | 516 | | | 2.13% | | 0.9176 | 0.6757 | | |
| GO:0051716 | cellular response to stimulus | [4](http://sas.ebioservice.com/bioinfoplug_molnetgotree.showgene.do?acc=GO:0051716&name=cellular+response+to+stimulus&recordid=74154068&loginid=BH11163) | | 259 | | | 1.54% | | 0.9559 | 0.6757 | | |
| GO:0055085 | transmembrane transport | [10](http://sas.ebioservice.com/bioinfoplug_molnetgotree.showgene.do?acc=GO:0055085&name=transmembrane+transport&recordid=74154068&loginid=BH11163) | | 152 | | | 6.58% | | 0.0239 | 0.0634 | | |
| GO:0048522 | positive regulation of cellular process | [7](http://sas.ebioservice.com/bioinfoplug_molnetgotree.showgene.do?acc=GO:0048522&name=positive+regulation+of+cellular+process&recordid=74154068&loginid=BH11163) | | 132 | | | 5.3% | | 0.1229 | 0.2047 | | |
| GO:0048523 | negative regulation of cellular process | [9](http://sas.ebioservice.com/bioinfoplug_molnetgotree.showgene.do?acc=GO:0048523&name=negative+regulation+of+cellular+process&recordid=74154068&loginid=BH11163) | | 181 | | | 4.97% | | 0.1166 | 0.2035 | | |
| GO:0050794 | regulation of cellular process | [168](http://sas.ebioservice.com/bioinfoplug_molnetgotree.showgene.do?acc=GO:0050794&name=regulation+of+cellular+process&recordid=74154068&loginid=BH11163) | | 3034 | | | 5.54% | | 0.0 | 0.0 | | |
| GO:0010926 | anatomical structure formation | [4](http://sas.ebioservice.com/bioinfoplug_molnetgotree.showgene.do?acc=GO:0010926&name=anatomical+structure+formation&recordid=74154068&loginid=BH11163) | | 341 | | | 1.17% | | 0.9925 | 0.6757 | | |
| GO:0022607 | cellular component assembly | [3](http://sas.ebioservice.com/bioinfoplug_molnetgotree.showgene.do?acc=GO:0022607&name=cellular+component+assembly&recordid=74154068&loginid=BH11163) | | 272 | | | 1.1% | | 0.9893 | 0.6757 | | |
| GO:0048646 | anatomical structure formation involved in morphogenesis | [1](http://sas.ebioservice.com/bioinfoplug_molnetgotree.showgene.do?acc=GO:0048646&name=anatomical+structure+formation+involved+in+morphogenesis&recordid=74154068&loginid=BH11163) | | 94 | | | 1.06% | | 0.944 | 0.6757 | | |
| GO:0016265 | death | [18](http://sas.ebioservice.com/bioinfoplug_molnetgotree.showgene.do?acc=GO:0016265&name=death&recordid=74154068&loginid=BH11163) | | 237 | | | 7.59% | | 7.0E-4 | 0.0035 | | |
| GO:0008219 | cell death | [18](http://sas.ebioservice.com/bioinfoplug_molnetgotree.showgene.do?acc=GO:0008219&name=cell+death&recordid=74154068&loginid=BH11163) | | 237 | | | 7.59% | | 7.0E-4 | 0.0035 | | |
| GO:0022414 | reproductive process | [18](http://sas.ebioservice.com/bioinfoplug_molnetgotree.showgene.do?acc=GO:0022414&name=reproductive+process&recordid=74154068&loginid=BH11163) | | 920 | | | 1.96% | | 0.9844 | 0.6757 | | |
| GO:0003006 | reproductive developmental process | [14](http://sas.ebioservice.com/bioinfoplug_molnetgotree.showgene.do?acc=GO:0003006&name=reproductive+developmental+process&recordid=74154068&loginid=BH11163) | | 764 | | | 1.83% | | 0.9862 | 0.6757 | | |
| GO:0007276 | gamete generation | [1](http://sas.ebioservice.com/bioinfoplug_molnetgotree.showgene.do?acc=GO:0007276&name=gamete+generation&recordid=74154068&loginid=BH11163) | | 20 | | | 5.0% | | 0.4707 | 0.5132 | | |
| GO:0009566 | fertilization | [1](http://sas.ebioservice.com/bioinfoplug_molnetgotree.showgene.do?acc=GO:0009566&name=fertilization&recordid=74154068&loginid=BH11163) | | 24 | | | 4.17% | | 0.5312 | 0.5714 | | |
| GO:0009856 | pollination | [2](http://sas.ebioservice.com/bioinfoplug_molnetgotree.showgene.do?acc=GO:0009856&name=pollination&recordid=74154068&loginid=BH11163) | | 127 | | | 1.57% | | 0.9006 | 0.6757 | | |
| GO:0048609 | reproductive process in a multicellular organism | [1](http://sas.ebioservice.com/bioinfoplug_molnetgotree.showgene.do?acc=GO:0048609&name=reproductive+process+in+a+multicellular+organism&recordid=74154068&loginid=BH11163) | | 31 | | | 3.23% | | 0.6208 | 0.6466 | | |
| GO:0022610 | biological adhesion | [1](http://sas.ebioservice.com/bioinfoplug_molnetgotree.showgene.do?acc=GO:0022610&name=biological+adhesion&recordid=74154068&loginid=BH11163) | | 17 | | | 5.88% | | 0.4203 | 0.491 | | |
| GO:0007155 | cell adhesion | [1](http://sas.ebioservice.com/bioinfoplug_molnetgotree.showgene.do?acc=GO:0007155&name=cell+adhesion&recordid=74154068&loginid=BH11163) | | 17 | | | 5.88% | | 0.4203 | 0.491 | | |
| GO:0032501 | multicellular organismal process | [53](http://sas.ebioservice.com/bioinfoplug_molnetgotree.showgene.do?acc=GO:0032501&name=multicellular+organismal+process&recordid=74154068&loginid=BH11163) | | 1658 | | | 3.2% | | 0.4084 | 0.491 | | |
| GO:0003008 | system process | [1](http://sas.ebioservice.com/bioinfoplug_molnetgotree.showgene.do?acc=GO:0003008&name=system+process&recordid=74154068&loginid=BH11163) | | 14 | | | 7.14% | | 0.3652 | 0.4665 | | |
| GO:0007275 | multicellular organismal development | [51](http://sas.ebioservice.com/bioinfoplug_molnetgotree.showgene.do?acc=GO:0007275&name=multicellular+organismal+development&recordid=74154068&loginid=BH11163) | | 1617 | | | 3.15% | | 0.4485 | 0.4956 | | |
| GO:0009845 | seed germination | [3](http://sas.ebioservice.com/bioinfoplug_molnetgotree.showgene.do?acc=GO:0009845&name=seed+germination&recordid=74154068&loginid=BH11163) | | 65 | | | 4.62% | | 0.3312 | 0.4298 | | |
| GO:0032504 | multicellular organism reproduction | [1](http://sas.ebioservice.com/bioinfoplug_molnetgotree.showgene.do?acc=GO:0032504&name=multicellular+organism+reproduction&recordid=74154068&loginid=BH11163) | | 34 | | | 2.94% | | 0.6538 | 0.6652 | | |
| GO:0035264 | multicellular organism growth | [1](http://sas.ebioservice.com/bioinfoplug_molnetgotree.showgene.do?acc=GO:0035264&name=multicellular+organism+growth&recordid=74154068&loginid=BH11163) | | 2 | | | 50.0% | | 0.0869 | 0.1691 | | |
| GO:0048609 | reproductive process in a multicellular organism | [1](http://sas.ebioservice.com/bioinfoplug_molnetgotree.showgene.do?acc=GO:0048609&name=reproductive+process+in+a+multicellular+organism&recordid=74154068&loginid=BH11163) | | 31 | | | 3.23% | | 0.6208 | 0.6466 | | |
| GO:0051239 | regulation of multicellular organismal process | [7](http://sas.ebioservice.com/bioinfoplug_molnetgotree.showgene.do?acc=GO:0051239&name=regulation+of+multicellular+organismal+process&recordid=74154068&loginid=BH11163) | | 202 | | | 3.47% | | 0.4321 | 0.4941 | | |
| GO:0032502 | developmental process | [65](http://sas.ebioservice.com/bioinfoplug_molnetgotree.showgene.do?acc=GO:0032502&name=developmental+process&recordid=74154068&loginid=BH11163) | | 1756 | | | 3.7% | | 0.0774 | 0.1582 | | |
| GO:0003006 | reproductive developmental process | [14](http://sas.ebioservice.com/bioinfoplug_molnetgotree.showgene.do?acc=GO:0003006&name=reproductive+developmental+process&recordid=74154068&loginid=BH11163) | | 764 | | | 1.83% | | 0.9862 | 0.6757 | | |
| GO:0007275 | multicellular organismal development | [51](http://sas.ebioservice.com/bioinfoplug_molnetgotree.showgene.do?acc=GO:0007275&name=multicellular+organismal+development&recordid=74154068&loginid=BH11163) | | 1617 | | | 3.15% | | 0.4485 | 0.4956 | | |
| GO:0007389 | pattern specification process | [1](http://sas.ebioservice.com/bioinfoplug_molnetgotree.showgene.do?acc=GO:0007389&name=pattern+specification+process&recordid=74154068&loginid=BH11163) | | 119 | | | 0.84% | | 0.9738 | 0.6757 | | |
| GO:0007568 | aging | [7](http://sas.ebioservice.com/bioinfoplug_molnetgotree.showgene.do?acc=GO:0007568&name=aging&recordid=74154068&loginid=BH11163) | | 74 | | | 9.46% | | 0.0106 | 0.0361 | | |
| GO:0009561 | megagametogenesis | [2](http://sas.ebioservice.com/bioinfoplug_molnetgotree.showgene.do?acc=GO:0009561&name=megagametogenesis&recordid=74154068&loginid=BH11163) | | 45 | | | 4.44% | | 0.4111 | 0.491 | | |
| GO:0009653 | anatomical structure morphogenesis | [10](http://sas.ebioservice.com/bioinfoplug_molnetgotree.showgene.do?acc=GO:0009653&name=anatomical+structure+morphogenesis&recordid=74154068&loginid=BH11163) | | 389 | | | 2.57% | | 0.7546 | 0.6757 | | |
| GO:0009790 | embryonic development | [3](http://sas.ebioservice.com/bioinfoplug_molnetgotree.showgene.do?acc=GO:0009790&name=embryonic+development&recordid=74154068&loginid=BH11163) | | 415 | | | 0.72% | | 0.9997 | 0.6757 | | |
| GO:0009791 | post-embryonic development | [21](http://sas.ebioservice.com/bioinfoplug_molnetgotree.showgene.do?acc=GO:0009791&name=post-embryonic+development&recordid=74154068&loginid=BH11163) | | 928 | | | 2.26% | | 0.9402 | 0.6757 | | |
| GO:0010228 | vegetative to reproductive phase transition | [3](http://sas.ebioservice.com/bioinfoplug_molnetgotree.showgene.do?acc=GO:0010228&name=vegetative+to+reproductive+phase+transition&recordid=74154068&loginid=BH11163) | | 80 | | | 3.75% | | 0.4521 | 0.4962 | | |
| GO:0021700 | developmental maturation | [3](http://sas.ebioservice.com/bioinfoplug_molnetgotree.showgene.do?acc=GO:0021700&name=developmental+maturation&recordid=74154068&loginid=BH11163) | | 47 | | | 6.38% | | 0.187 | 0.2806 | | |
| GO:0022611 | dormancy process | [1](http://sas.ebioservice.com/bioinfoplug_molnetgotree.showgene.do?acc=GO:0022611&name=dormancy+process&recordid=74154068&loginid=BH11163) | | 14 | | | 7.14% | | 0.3652 | 0.4665 | | |
| GO:0048589 | developmental growth | [2](http://sas.ebioservice.com/bioinfoplug_molnetgotree.showgene.do?acc=GO:0048589&name=developmental+growth&recordid=74154068&loginid=BH11163) | | 105 | | | 1.9% | | 0.8326 | 0.6757 | | |
| GO:0048646 | anatomical structure formation involved in morphogenesis | [1](http://sas.ebioservice.com/bioinfoplug_molnetgotree.showgene.do?acc=GO:0048646&name=anatomical+structure+formation+involved+in+morphogenesis&recordid=74154068&loginid=BH11163) | | 94 | | | 1.06% | | 0.944 | 0.6757 | | |
| GO:0048856 | anatomical structure development | [37](http://sas.ebioservice.com/bioinfoplug_molnetgotree.showgene.do?acc=GO:0048856&name=anatomical+structure+development&recordid=74154068&loginid=BH11163) | | 1307 | | | 2.83% | | 0.7168 | 0.6757 | | |
| GO:0048869 | cellular developmental process | [7](http://sas.ebioservice.com/bioinfoplug_molnetgotree.showgene.do?acc=GO:0048869&name=cellular+developmental+process&recordid=74154068&loginid=BH11163) | | 324 | | | 2.16% | | 0.8669 | 0.6757 | | |
| GO:0050793 | regulation of developmental process | [13](http://sas.ebioservice.com/bioinfoplug_molnetgotree.showgene.do?acc=GO:0050793&name=regulation+of+developmental+process&recordid=74154068&loginid=BH11163) | | 271 | | | 4.8% | | 0.0852 | 0.1691 | | |
| GO:0051093 | negative regulation of developmental process | [2](http://sas.ebioservice.com/bioinfoplug_molnetgotree.showgene.do?acc=GO:0051093&name=negative+regulation+of+developmental+process&recordid=74154068&loginid=BH11163) | | 92 | | | 2.17% | | 0.7747 | 0.6757 | | |
| GO:0051094 | positive regulation of developmental process | [5](http://sas.ebioservice.com/bioinfoplug_molnetgotree.showgene.do?acc=GO:0051094&name=positive+regulation+of+developmental+process&recordid=74154068&loginid=BH11163) | | 50 | | | 10.0% | | 0.0238 | 0.0634 | | |
| GO:0040007 | growth | [12](http://sas.ebioservice.com/bioinfoplug_molnetgotree.showgene.do?acc=GO:0040007&name=growth&recordid=74154068&loginid=BH11163) | | 291 | | | 4.12% | | 0.197 | 0.2929 | | |
| GO:0016049 | cell growth | [10](http://sas.ebioservice.com/bioinfoplug_molnetgotree.showgene.do?acc=GO:0016049&name=cell+growth&recordid=74154068&loginid=BH11163) | | 229 | | | 4.37% | | 0.1792 | 0.2714 | | |
| GO:0035264 | multicellular organism growth | [1](http://sas.ebioservice.com/bioinfoplug_molnetgotree.showgene.do?acc=GO:0035264&name=multicellular+organism+growth&recordid=74154068&loginid=BH11163) | | 2 | | | 50.0% | | 0.0869 | 0.1691 | | |
| GO:0035265 | organ growth | [2](http://sas.ebioservice.com/bioinfoplug_molnetgotree.showgene.do?acc=GO:0035265&name=organ+growth&recordid=74154068&loginid=BH11163) | | 13 | | | 15.38% | | 0.0722 | 0.1495 | | |
| GO:0048589 | developmental growth | [2](http://sas.ebioservice.com/bioinfoplug_molnetgotree.showgene.do?acc=GO:0048589&name=developmental+growth&recordid=74154068&loginid=BH11163) | | 105 | | | 1.9% | | 0.8326 | 0.6757 | | |
| GO:0040008 | regulation of growth | [1](http://sas.ebioservice.com/bioinfoplug_molnetgotree.showgene.do?acc=GO:0040008&name=regulation+of+growth&recordid=74154068&loginid=BH11163) | | 48 | | | 2.08% | | 0.7735 | 0.6757 | | |
| GO:0045926 | negative regulation of growth | [1](http://sas.ebioservice.com/bioinfoplug_molnetgotree.showgene.do?acc=GO:0045926&name=negative+regulation+of+growth&recordid=74154068&loginid=BH11163) | | 9 | | | 11.11% | | 0.2613 | 0.3622 | | |
| GO:0048511 | rhythmic process | [5](http://sas.ebioservice.com/bioinfoplug_molnetgotree.showgene.do?acc=GO:0048511&name=rhythmic+process&recordid=74154068&loginid=BH11163) | | 49 | | | 10.2% | | 0.0222 | 0.0625 | | |
| GO:0007623 | circadian rhythm | [5](http://sas.ebioservice.com/bioinfoplug_molnetgotree.showgene.do?acc=GO:0007623&name=circadian+rhythm&recordid=74154068&loginid=BH11163) | | 49 | | | 10.2% | | 0.0222 | 0.0625 | | |
| GO:0050896 | response to stimulus | [197](http://sas.ebioservice.com/bioinfoplug_molnetgotree.showgene.do?acc=GO:0050896&name=response+to+stimulus&recordid=74154068&loginid=BH11163) | | 3431 | | | 5.74% | | 0.0 | 0.0 | | |
| GO:0006950 | response to stress | [120](http://sas.ebioservice.com/bioinfoplug_molnetgotree.showgene.do?acc=GO:0006950&name=response+to+stress&recordid=74154068&loginid=BH11163) | | 1955 | | | 6.14% | | 0.0 | 0.0 | | |
| GO:0006955 | immune response | [37](http://sas.ebioservice.com/bioinfoplug_molnetgotree.showgene.do?acc=GO:0006955&name=immune+response&recordid=74154068&loginid=BH11163) | | 312 | | | 11.86% | | 0.0 | 0.0 | | |
| GO:0009605 | response to external stimulus | [24](http://sas.ebioservice.com/bioinfoplug_molnetgotree.showgene.do?acc=GO:0009605&name=response+to+external+stimulus&recordid=74154068&loginid=BH11163) | | 332 | | | 7.23% | | 2.0E-4 | 0.0012 | | |
| GO:0009607 | response to biotic stimulus | [45](http://sas.ebioservice.com/bioinfoplug_molnetgotree.showgene.do?acc=GO:0009607&name=response+to+biotic+stimulus&recordid=74154068&loginid=BH11163) | | 583 | | | 7.72% | | 0.0 | 0.0 | | |
| GO:0009628 | response to abiotic stimulus | [52](http://sas.ebioservice.com/bioinfoplug_molnetgotree.showgene.do?acc=GO:0009628&name=response+to+abiotic+stimulus&recordid=74154068&loginid=BH11163) | | 1156 | | | 4.5% | | 0.0056 | 0.0213 | | |
| GO:0009719 | response to endogenous stimulus | [56](http://sas.ebioservice.com/bioinfoplug_molnetgotree.showgene.do?acc=GO:0009719&name=response+to+endogenous+stimulus&recordid=74154068&loginid=BH11163) | | 832 | | | 6.73% | | 0.0 | 0.0 | | |
| GO:0042221 | response to chemical stimulus | [107](http://sas.ebioservice.com/bioinfoplug_molnetgotree.showgene.do?acc=GO:0042221&name=response+to+chemical+stimulus&recordid=74154068&loginid=BH11163) | | 1746 | | | 6.13% | | 0.0 | 0.0 | | |
| GO:0051606 | detection of stimulus | [1](http://sas.ebioservice.com/bioinfoplug_molnetgotree.showgene.do?acc=GO:0051606&name=detection+of+stimulus&recordid=74154068&loginid=BH11163) | | 37 | | | 2.7% | | 0.6839 | 0.6737 | | |
| GO:0051716 | cellular response to stimulus | [4](http://sas.ebioservice.com/bioinfoplug_molnetgotree.showgene.do?acc=GO:0051716&name=cellular+response+to+stimulus&recordid=74154068&loginid=BH11163) | | 259 | | | 1.54% | | 0.9559 | 0.6757 | | |
| GO:0048583 | regulation of response to stimulus | [19](http://sas.ebioservice.com/bioinfoplug_molnetgotree.showgene.do?acc=GO:0048583&name=regulation+of+response+to+stimulus&recordid=74154068&loginid=BH11163) | | 150 | | | 12.67% | | 0.0 | 0.0 | | |
| GO:0048584 | positive regulation of response to stimulus | [8](http://sas.ebioservice.com/bioinfoplug_molnetgotree.showgene.do?acc=GO:0048584&name=positive+regulation+of+response+to+stimulus&recordid=74154068&loginid=BH11163) | | 57 | | | 14.04% | | 7.0E-4 | 0.0035 | | |
| GO:0048585 | negative regulation of response to stimulus | [8](http://sas.ebioservice.com/bioinfoplug_molnetgotree.showgene.do?acc=GO:0048585&name=negative+regulation+of+response+to+stimulus&recordid=74154068&loginid=BH11163) | | 64 | | | 12.5% | | 0.0014 | 0.0063 | | |
| GO:0051179 | localization | [58](http://sas.ebioservice.com/bioinfoplug_molnetgotree.showgene.do?acc=GO:0051179&name=localization&recordid=74154068&loginid=BH11163) | | 1819 | | | 3.19% | | 0.4097 | 0.491 | | |
| GO:0033036 | macromolecule localization | [12](http://sas.ebioservice.com/bioinfoplug_molnetgotree.showgene.do?acc=GO:0033036&name=macromolecule+localization&recordid=74154068&loginid=BH11163) | | 429 | | | 2.8% | | 0.6672 | 0.6694 | | |
| GO:0051641 | cellular localization | [11](http://sas.ebioservice.com/bioinfoplug_molnetgotree.showgene.do?acc=GO:0051641&name=cellular+localization&recordid=74154068&loginid=BH11163) | | 516 | | | 2.13% | | 0.9176 | 0.6757 | | |
| GO:0051234 | establishment of localization | [56](http://sas.ebioservice.com/bioinfoplug_molnetgotree.showgene.do?acc=GO:0051234&name=establishment+of+localization&recordid=74154068&loginid=BH11163) | | 1760 | | | 3.18% | | 0.4182 | 0.491 | | |
| GO:0051234 | establishment of localization | [56](http://sas.ebioservice.com/bioinfoplug_molnetgotree.showgene.do?acc=GO:0051234&name=establishment+of+localization&recordid=74154068&loginid=BH11163) | | 1760 | | | 3.18% | | 0.4182 | 0.491 | | |
| GO:0006810 | transport | [56](http://sas.ebioservice.com/bioinfoplug_molnetgotree.showgene.do?acc=GO:0006810&name=transport&recordid=74154068&loginid=BH11163) | | 1755 | | | 3.19% | | 0.4099 | 0.491 | | |
| GO:0045184 | establishment of protein localization | [10](http://sas.ebioservice.com/bioinfoplug_molnetgotree.showgene.do?acc=GO:0045184&name=establishment+of+protein+localization&recordid=74154068&loginid=BH11163) | | 363 | | | 2.75% | | 0.6779 | 0.6737 | | |
| GO:0051649 | establishment of localization in cell | [11](http://sas.ebioservice.com/bioinfoplug_molnetgotree.showgene.do?acc=GO:0051649&name=establishment+of+localization+in+cell&recordid=74154068&loginid=BH11163) | | 494 | | | 2.23% | | 0.8902 | 0.6757 | | |
| GO:0051704 | multi-organism process | [45](http://sas.ebioservice.com/bioinfoplug_molnetgotree.showgene.do?acc=GO:0051704&name=multi-organism+process&recordid=74154068&loginid=BH11163) | | 691 | | | 6.51% | | 0.0 | 0.0 | | |
| GO:0009856 | pollination | [2](http://sas.ebioservice.com/bioinfoplug_molnetgotree.showgene.do?acc=GO:0009856&name=pollination&recordid=74154068&loginid=BH11163) | | 127 | | | 1.57% | | 0.9006 | 0.6757 | | |
| GO:0051707 | response to other organism | [43](http://sas.ebioservice.com/bioinfoplug_molnetgotree.showgene.do?acc=GO:0051707&name=response+to+other+organism&recordid=74154068&loginid=BH11163) | | 539 | | | 7.98% | | 0.0 | 0.0 | | |
| GO:0043900 | regulation of multi-organism process | [1](http://sas.ebioservice.com/bioinfoplug_molnetgotree.showgene.do?acc=GO:0043900&name=regulation+of+multi-organism+process&recordid=74154068&loginid=BH11163) | | 7 | | | 14.29% | | 0.2152 | 0.3114 | | |
| GO:0065007 | biological regulation | [191](http://sas.ebioservice.com/bioinfoplug_molnetgotree.showgene.do?acc=GO:0065007&name=biological+regulation&recordid=74154068&loginid=BH11163) | | 3747 | | | 5.1% | | 0.0 | 0.0 | | |
| GO:0050789 | regulation of biological process | [178](http://sas.ebioservice.com/bioinfoplug_molnetgotree.showgene.do?acc=GO:0050789&name=regulation+of+biological+process&recordid=74154068&loginid=BH11163) | | 3304 | | | 5.39% | | 0.0 | 0.0 | | |
| GO:0065008 | regulation of biological quality | [28](http://sas.ebioservice.com/bioinfoplug_molnetgotree.showgene.do?acc=GO:0065008&name=regulation+of+biological+quality&recordid=74154068&loginid=BH11163) | | 610 | | | 4.59% | | 0.0285 | 0.0739 | | |
| GO:0065009 | regulation of molecular function | [6](http://sas.ebioservice.com/bioinfoplug_molnetgotree.showgene.do?acc=GO:0065009&name=regulation+of+molecular+function&recordid=74154068&loginid=BH11163) | | 143 | | | 4.2% | | 0.2861 | 0.3803 | | |
| GO:0048518 | positive regulation of biological process | [13](http://sas.ebioservice.com/bioinfoplug_molnetgotree.showgene.do?acc=GO:0048518&name=positive+regulation+of+biological+process&recordid=74154068&loginid=BH11163) | | 208 | | | 6.25% | | 0.0159 | 0.0491 | | |
| GO:0002684 | positive regulation of immune system process | [4](http://sas.ebioservice.com/bioinfoplug_molnetgotree.showgene.do?acc=GO:0002684&name=positive+regulation+of+immune+system+process&recordid=74154068&loginid=BH11163) | | 29 | | | 13.79% | | 0.0162 | 0.0491 | | |
| GO:0009893 | positive regulation of metabolic process | [2](http://sas.ebioservice.com/bioinfoplug_molnetgotree.showgene.do?acc=GO:0009893&name=positive+regulation+of+metabolic+process&recordid=74154068&loginid=BH11163) | | 80 | | | 2.5% | | 0.7065 | 0.6757 | | |
| GO:0048522 | positive regulation of cellular process | [7](http://sas.ebioservice.com/bioinfoplug_molnetgotree.showgene.do?acc=GO:0048522&name=positive+regulation+of+cellular+process&recordid=74154068&loginid=BH11163) | | 132 | | | 5.3% | | 0.1229 | 0.2047 | | |
| GO:0048584 | positive regulation of response to stimulus | [8](http://sas.ebioservice.com/bioinfoplug_molnetgotree.showgene.do?acc=GO:0048584&name=positive+regulation+of+response+to+stimulus&recordid=74154068&loginid=BH11163) | | 57 | | | 14.04% | | 7.0E-4 | 0.0035 | | |
| GO:0051094 | positive regulation of developmental process | [5](http://sas.ebioservice.com/bioinfoplug_molnetgotree.showgene.do?acc=GO:0051094&name=positive+regulation+of+developmental+process&recordid=74154068&loginid=BH11163) | | 50 | | | 10.0% | | 0.0238 | 0.0634 | | |
| GO:0048519 | negative regulation of biological process | [15](http://sas.ebioservice.com/bioinfoplug_molnetgotree.showgene.do?acc=GO:0048519&name=negative+regulation+of+biological+process&recordid=74154068&loginid=BH11163) | | 346 | | | 4.34% | | 0.1252 | 0.2047 | | |
| GO:0009892 | negative regulation of metabolic process | [4](http://sas.ebioservice.com/bioinfoplug_molnetgotree.showgene.do?acc=GO:0009892&name=negative+regulation+of+metabolic+process&recordid=74154068&loginid=BH11163) | | 190 | | | 2.11% | | 0.8338 | 0.6757 | | |
| GO:0045926 | negative regulation of growth | [1](http://sas.ebioservice.com/bioinfoplug_molnetgotree.showgene.do?acc=GO:0045926&name=negative+regulation+of+growth&recordid=74154068&loginid=BH11163) | | 9 | | | 11.11% | | 0.2613 | 0.3622 | | |
| GO:0048523 | negative regulation of cellular process | [9](http://sas.ebioservice.com/bioinfoplug_molnetgotree.showgene.do?acc=GO:0048523&name=negative+regulation+of+cellular+process&recordid=74154068&loginid=BH11163) | | 181 | | | 4.97% | | 0.1166 | 0.2035 | | |
| GO:0048585 | negative regulation of response to stimulus | [8](http://sas.ebioservice.com/bioinfoplug_molnetgotree.showgene.do?acc=GO:0048585&name=negative+regulation+of+response+to+stimulus&recordid=74154068&loginid=BH11163) | | 64 | | | 12.5% | | 0.0014 | 0.0063 | | |
| GO:0051093 | negative regulation of developmental process | [2](http://sas.ebioservice.com/bioinfoplug_molnetgotree.showgene.do?acc=GO:0051093&name=negative+regulation+of+developmental+process&recordid=74154068&loginid=BH11163) | | 92 | | | 2.17% | | 0.7747 | 0.6757 | | |
| GO:0050789 | regulation of biological process | [178](http://sas.ebioservice.com/bioinfoplug_molnetgotree.showgene.do?acc=GO:0050789&name=regulation+of+biological+process&recordid=74154068&loginid=BH11163) | | 3304 | | | 5.39% | | 0.0 | 0.0 | | |
| GO:0002682 | regulation of immune system process | [6](http://sas.ebioservice.com/bioinfoplug_molnetgotree.showgene.do?acc=GO:0002682&name=regulation+of+immune+system+process&recordid=74154068&loginid=BH11163) | | 37 | | | 16.22% | | 0.0016 | 0.0073 | | |
| GO:0019222 | regulation of metabolic process | [105](http://sas.ebioservice.com/bioinfoplug_molnetgotree.showgene.do?acc=GO:0019222&name=regulation+of+metabolic+process&recordid=74154068&loginid=BH11163) | | 1928 | | | 5.45% | | 0.0 | 0.0 | | |
| GO:0040008 | regulation of growth | [1](http://sas.ebioservice.com/bioinfoplug_molnetgotree.showgene.do?acc=GO:0040008&name=regulation+of+growth&recordid=74154068&loginid=BH11163) | | 48 | | | 2.08% | | 0.7735 | 0.6757 | | |
| GO:0042752 | regulation of circadian rhythm | [2](http://sas.ebioservice.com/bioinfoplug_molnetgotree.showgene.do?acc=GO:0042752&name=regulation+of+circadian+rhythm&recordid=74154068&loginid=BH11163) | | 17 | | | 11.76% | | 0.1089 | 0.1997 | | |
| GO:0043900 | regulation of multi-organism process | [1](http://sas.ebioservice.com/bioinfoplug_molnetgotree.showgene.do?acc=GO:0043900&name=regulation+of+multi-organism+process&recordid=74154068&loginid=BH11163) | | 7 | | | 14.29% | | 0.2152 | 0.3114 | | |
| GO:0048518 | positive regulation of biological process | [13](http://sas.ebioservice.com/bioinfoplug_molnetgotree.showgene.do?acc=GO:0048518&name=positive+regulation+of+biological+process&recordid=74154068&loginid=BH11163) | | 208 | | | 6.25% | | 0.0159 | 0.0491 | | |
| GO:0048519 | negative regulation of biological process | [15](http://sas.ebioservice.com/bioinfoplug_molnetgotree.showgene.do?acc=GO:0048519&name=negative+regulation+of+biological+process&recordid=74154068&loginid=BH11163) | | 346 | | | 4.34% | | 0.1252 | 0.2047 | | |
| GO:0048583 | regulation of response to stimulus | [19](http://sas.ebioservice.com/bioinfoplug_molnetgotree.showgene.do?acc=GO:0048583&name=regulation+of+response+to+stimulus&recordid=74154068&loginid=BH11163) | | 150 | | | 12.67% | | 0.0 | 0.0 | | |
| GO:0050793 | regulation of developmental process | [13](http://sas.ebioservice.com/bioinfoplug_molnetgotree.showgene.do?acc=GO:0050793&name=regulation+of+developmental+process&recordid=74154068&loginid=BH11163) | | 271 | | | 4.8% | | 0.0852 | 0.1691 | | |
| GO:0050794 | regulation of cellular process | [168](http://sas.ebioservice.com/bioinfoplug_molnetgotree.showgene.do?acc=GO:0050794&name=regulation+of+cellular+process&recordid=74154068&loginid=BH11163) | | 3034 | | | 5.54% | | 0.0 | 0.0 | | |
| GO:0051239 | regulation of multicellular organismal process | [7](http://sas.ebioservice.com/bioinfoplug_molnetgotree.showgene.do?acc=GO:0051239&name=regulation+of+multicellular+organismal+process&recordid=74154068&loginid=BH11163) | | 202 | | | 3.47% | | 0.4321 | 0.4941 | | |
