# Supplementary material for: The bZIP protein from Tamarix hispida, ThbZIP1, is ACGT elements binding factor that enhances abiotic stress signaling in transgenic Arabidopsis
Source: BMC Plant Biol. 2013 Oct 4;13:151. doi: 10.1186/1471-2229-13-151 (PMC3852707; doi:10.1186/1471-2229-13-151)
Supplement: Additional file 7: Table S4 — The significantly upregulated genes in transgenic plants overexpression of ThbZIP1. [file 1471-2229-13-151-S7.doc]

Additional file 7: Table S4 The significantly upregulated genes in transgenic plants overexpression of ThbZIP1

| **Probe Id** | | **Locus tag** | **Fold**  **change** | **bZIP Recognition**  **Sequence** | **Description** |
| --- | --- | --- | --- | --- | --- |
| **Control** | | | | | |
| A_84_P19231 | AT2G26150 | | 25.0335 | -114 (CACGTG)  -399 (CACGTG) | ATHSFA2 |
| A_84_P14320 | AT1G74310 | | 8.5474 | -142 (CACGTG) | protein coding |
| A_84_P11916 | AT4G12870 | | 5.0984 | -107 (CACGTG)  -229 (TACGTA) | GILT family protein |
| A_84_P17757 | AT5G18670 | | 4.921 | -80 (CACGTG) | beta-amylase |
| A_84_P162443 | AT5G64510 | | 3.7383 | -172 (GACGTC) | hypothetical protein |
| A_84_P22292 | AT4G04020 | | 3.6355 | -170 (CACGTG) | structural molecule |
| A_84_P12513 | AT2G22240 | | 3.6151 | -85 (CACGTG)  -268 (CACGTG)  -776 (CACGTG) | inositol-3-phosphate synthase isozyme 2 |
| A_84_P118252 | AT2G36630 | | 3.6117 | -112 (CACGTG) | hypothetical protein |
| A_84_P18342 | AT3G16050 | | 3.4779 | -64 (CACGTG)  -240 (GACGTC)  -261 (CACGTG)  -323 (TACGTA)  -590 (TACGTA) | protein coding |
| A_84_P844786 | AT2G16890 | | 3.1108 | -435 (TACGTA)  -537 (TACGTA)  -887 (TACGTA) | UDP-glucosyl transferase family protein |
| A_84_P283680 | AT3G07090 | | 3.0806 | -73 (GACGTC) | protein coding |
| A_84_P12228 | AT5G67280 | | 3.0315 | -164 (GACGTC) | RLK (RECEPTOR-LIKE KINASE) |
| A_84_P10477 | AT1G13080 | | 3.0026 | -225 (CACGTG) | protein coding |
| A_84_P279650 | AT1G19490 | | 2.928 | -149 (CACGTG)  -252 (CACGTG)  -374 (CACGTG)  -408 (GACGTC) | protein coding |
| A_84_P767112 | AT5G09590 | | 2.8374 | -24 (GACGTC)  -78 (CACGTG) | mtHSC70-2 |
| A_84_P16958 | AT5G15450 | | 2.8241 | -177 (CACGTG) | APG6/CLPB-P |
| A_84_P766447 | AT5G02500 | | 2.7038 | -251 (CACGTG) | HSC70-1 |
| A_84_P126821 | AT2G46790 | | 2.6092 | -214 (CACGTG)  -286 (CACGTG) | APRR9 transcription regulator |
| A_84_P19603 | AT1G62740 | | 2.5341 | -182 (GACGTC) | protein coding |
| A_84_P588557 | AT4G36830 | | 2.4922 | -511 (TACGTA)  -652 (TACGTA)  -722 (CACGTG)  -747 (TACGTA) | SUR4 membrane family protein |
| A_84_P12309 | AT1G67360 | | 2.4278 | -268 (CACGTG) | protein coding |
| A_84_P22433 | AT5G01260 | | 2.3834 | -57 (CACGTG)  -154 (CACGTG) | glycoside hydrolase starch-binding domain-containing protein |
| A_84_P805060 | AT5G61020 | | 2.1984 | -268 (TACGTA) | ECT3 |
| A_84_P21609 | AT1G56170 | | 2.0827 | -74 (CACGTG)  -189 (CACGTG)  -215 (CACGTG) | protein coding |
| A_84_P581411 | AT4G32295 | | 2.0356 | -267 (CACGTG)  -511 (GACGTC) | hypothetical protein |
| **Salt stress** | | | | | |
| A_84_P187174 | AT3G17520 | | 81.7964 | -231 (CACGTG) | protein coding |
| A_84_P20292 | AT3G02380 | | 20.3485 | -77 (CACGTG) | protein coding |
| A_84_P16823 | AT1G01060 | | 19.8187 | -111 (CACGTG) | protein coding |
| A_84_P23101 | AT3G21890 | | 14.4316 | -91 (CACGTG) | protein coding |
| A_84_P14787 | AT4G25580 | | 10.5901 | -51 (CACGTG)  -414 (CACGTG)  -501 (CACGTG) | stress-responsive protein-related |
| A_84_P21735 | AT1G12370 | | 10.4881 | -91 (CACGTG) | protein coding |
| A_84_P19231 | AT2G26150 | | 9.891 | -114 (CACGTG)  -399 (CACGTG) | ATHSFA2 |
| A_84_P19904 | AT1G10370 | | 9.4026 | -106 (CACGTG) | protein coding |
| A_84_P12766 | AT3G51240 | | 9.299 | -321 (CACGTG)  -733 (GACGTC) | protein coding |
| A_84_P19702 | AT5G44110 | | 8.9815 | -659 (TACGTA) | POP1 |
| A_84_P14520 | AT3G01500 | | 8.611 | -356 (CACGTG)  -367 (TACGTA)  -860 (TACGTA) | protein coding |
| A_84_P11287 | AT5G15600 | | 7.5571 | -52 (CACGTG)  -269 (CACGTG) | SP1L4 (SPIRAL1-LIKE4) |
| A_84_P17757 | AT5G18670 | | 7.4279 | -80 (CACGTG) | BMY3 (BETA-AMYLASE 9) |
| A_84_P23539 | AT5G58770 | | 7.1298 | -474 (CACGTG) | dehydrodolichyl diphosphate synthase, putative |
| A_84_P809539 | AT2G34620 | | 6.6422 | -64 (CACGTG)  -152 (CACGTG) | mTERF-related |
| A_84_P16494 | AT3G21870 | | 6.5482 | -148 (CACGTG) | protein coding |
| A_84_P785139 | AT1G06360 | | 6.5266 | -59 (TACGTA) | protein coding |
| A_84_P581339 | AT4G12005 | | 5.3542 | -278 (CACGTG) | hypothetical protein |
| A_84_P199724 | AT5G43630 | | 5.2143 | -74 (CACGTG)  -372 (TACGTA) | zinc knuckle family protein |
| A_84_P21647 | AT5G58760 | | 5.1782 | -209 (CACGTG) | DDB2 (DAMAGED DNA-BINDING 2) |
| A_84_P536338 | AT5G56210 | | 2.495 | -77 (CACGTG) | WIP2 (WPP-DOMAIN INTERACTING PROTEIN 2) |
| A_84_P15827 | AT5G05860 | | 3.2476 | -756 (CACGTG) | UGT76C2 (UDP-glucosyl transferase 76C2) |
| A_84_P234663 | AT5G55950 | | 2.185 | -221 (CACGTG)  -508 (GACGTC)  -674 (TACGTA) | transporter-related |
| A_84_P16762 | AT5G02280 | | 2.2427 | -87 (GACGTC)  -125 (CACGTG) | synbindin, putative |
| A_84_P146548 | AT2G35840 | | 2.248 | -387 (CACGTG) | sucrose-phosphatase 1 (SPP1) |
| A_84_P806129 | AT5G38410 | | 2.0307 | -107 (CACGTG) | ribulose bisphosphate carboxylase small chain 3B |
| A_84_P805443 | AT5G38420 | | 2.0966 | -159 (CACGTG) | ribulose bisphosphate carboxylase small chain 2B |
| A_84_P19531 | AT4G28660 | | 4.5885 | -199 (CACGTG) | PSB28 |
| A_84_P18690 | AT5G13400 | | 2.4947 | -102 (CACGTG) | proton-dependent oligopeptide transport (POT) family protein |
| A_84_P13021 | AT5G14640 | | 2.0833 | -37 (GACGTC)  -72 (CACGTG) | protein kinase family protein |
| A_84_P20472 | AT4G27520 | | 2.5467 | -386 (TACGTA)  -614 (CACGTG)  -638 (CACGTG) | plastocyanin-like domain-containing protein |
| A_84_P161503 | AT5G17300 | | 3.0107 | -201 (CACGTG) | myb family transcription factor |
| A_84_P197514 | AT5G55580 | | 2.032 | -352 (GACGTC) | mTERF family protein |
| A_84_P12011 | AT4G39800 | | 3.821 | -146 (CACGTG)  -162 (CACGTG)  -223 (CACGTG)  -467 (CACGTG)  -601 (CACGTG) | MI-1-P SYNTHASE |
| A_84_P126821 | AT2G46790 | | 3.2007 | -214 (CACGTG)  -286 (CACGTG) | APRR9 (PSEUDO-RESPONSE REGULATOR 9) |
