# Supplementary material for: The bZIP protein from Tamarix hispida, ThbZIP1, is ACGT elements binding factor that enhances abiotic stress signaling in transgenic Arabidopsis
Source: BMC Plant Biol. 2013 Oct 4;13:151. doi: 10.1186/1471-2229-13-151 (PMC3852707; doi:10.1186/1471-2229-13-151)
Supplement: Additional file 8 — Primers used in the study. Table S5. Primer sequences used in construction of the overexpression vectors of ThbZIP1 and ThbZIP1 fused with GFP. Table S6. The primer sequences used in the yeast one-hybrid analyses. Table S7. The primers used for construction of the reporter vectors. Table S8. The primer sequences used in real-time RT-PCR. [file 1471-2229-13-151-S8.doc]

Additional file 8:

Table S5: Primer sequences used in construction of the overexpression vectors of ThbZIP1 and ThbZIP1 fused with GFP .

| **Primer names** | **Primers sequences (**5’-3’**)** |
| --- | --- |
| pROKII-*ThbZIP1*F | CGCGGATCCATGTATCAACCCGTGAGTTCTG |
| pROKII-*ThbZIP1*R | CGGGGTACCGAACTGAAACATATCAGCGGTA |
| *GFP-ThbZIP1*F | CTCTAGAGGATCCCCATGTATCAACCCGTGAGTTCTG |
| *GFP-ThbZIP1*R | GAACTGAAACATATCAGCGGTAT |
| *GFP*R | TCGAGCTCGGTACCCTCACTTGTACAGCTCATCCATGCC |

Table S6: The primer sequences used in the yeast one-hybrid analyses.

| **Primer names** | **Primers sequences (**5’-3’**)** |
| --- | --- |
| pHIS2-CF | AATTCCACGTGCACGTGCACGTGGAGCT |
| pHIS2-CR | CCACGTGCACGTGCCGTGG |
| pHIS2-GF | aattcGACGTGGACGTGGACGTGgagct |
| pHIS2-GR | CCACGTCCACGTCCACGTCG |
| pHIS2-AF | AATTCTACGTATACGTATACGTAGAGCT |
| pHIS2-AR | CTACGTATACGTATACGTAG |
| pHIS2-CM1F | AATTCCAAGTGCAAGTGCAAGTGGAGCT |
| pHIS2-CM1R | CCACTTGCACTTGCACTTGG |
| pHIS2-CM2F | AATTCCAAAAGCAAAAGCAAAAGGAGCT |
| pHIS2-CM2R | CCTTTTGCTTTTGCTTTTGG |
| pHIS2-CM3F | AATTCCACATGCACATGCACATGGAGCT |
| pHIS2-CM3R | CCATGTGCATGTGCATGTGG |
| pHIS2-CM4F | AATTCCACGAGCACGAGCACGAGGAGCT |
| pHIS2-CM4R | CCTCGTGCTCGTGCTCGTGG |
| pHIS2-EF | AATTCGCAAATGAGCAAATGAGCAAATGAGAGCT |
| pHIS2-ER | CTCATTTGCTCATTTGCTCATTTGCG |
| pHIS2-EM1F | AATTCGACAATGAGACAATGAGACAATGAGAGCT |
| pHIS2-EM1R | CTCATTGTCTCATTGTCTCATTGTCG |
| pHIS2-EM2F | AATTCGCACGTGAGCACGTGAGCACGTGAGAGCT |
| pHIS2-EM2R | CTCACGTGCTCACGTGCTCACGTGCG |
| pHIS2-EM3F | AATTCGACCGCAAGACCGCAAGACCGCAAGAGCT |
| pHIS2-EM3R | CTTGCGGTCTTGCGGTCTTGCGGTCG |
| pHIS2-E-boxp+F | CCGGAATTCATCATATGCTTTTATTGCACGC |
| pHIS2-E-boxp+R | CGAGCTCAACAACTGAAAAGGAATGCGAG |
| pHIS2-E-boxp-F | CCGGAATTCACCTTTGCCCTTTTACCCTATC |
| pHIS2-E-boxp-R | CGAGCTCATTTAAAGAAACCCGAAACCCT |
| *ThMYC4-*Rec2F | AAGCAGTGGTATCAACGCAGAGTGGCCATTATGGCCC ATGAACAGTTCGCCAGCGGATT |
| *ThMYC4*-Rec2R | TCTAGAGGCCGAGGCGGCCGACATGAATCCGCTGGCG  AACTGTTCAT |
| *ThMYC6-*Rec2F | AAGCAGTGGTATCAACGCAGAGTGGCCATTATGGCCC ATGACAAGTACTGCGGTGGGTA |
| *ThMYC6-*Rec2R | TCTAGAGGCCGAGGCGGCCGACATGATAGAACCGCTG  GTTGAGCTTC |
| *ThbZIP1-*Rec2*F* | AAGCAGTGGTATCAACGCAGAGTGGCCATTATGGCCC ATGTATCAACCCGTGAGTTCTG |
| *ThbZIP1-*Rec2*R* | TCTAGAGGCCGAGGCGGCCGACATGGAACTGAAACAT  ATCAGCGGTA |

Table S7: The primers used for construction of the reporter vectors.

| **Primer names** | **Primers sequences (**5’-3’**)** |
| --- | --- |
| pCAM-CF | AGCTTCACGTCCACGTCCACGTCACCCTTCCTCTATATAAGGAAGTTCATTTCATTTGGAGAGAACACGGC |
| pCAM-CR | CATGGCCGTGTTCTCTCCAAATGAAATGAACTTCCTTATATAGAGGAAGGGTGACGTGGACGTGGACGTGA |
| pCAM-GF | AGCTTCACGTGCACGTGCACGTGACCCTTCCTCTATATAAGGAAGTTCATTTCATTTGGAGAGAACACGGC |
| pCAM-GR | CATGGCCGTGTTCTCTCCAAATGAAATGAACTTCCTTATATAGAGGAAGGGTCACGTGCACGTGCACGTGA |
| pCAM-AF | AGCTTTACGTATACGTATACGTAACCCTTCCTCTATATAAGGAAGTTCATTTCATTTGGAGAGAACACGGC |
| pCAM-AR | CATGGCCGTGTTCTCTCCAAATGAAATGAACTTCCTTATATAGAGGAAGGGTTACGTATACGTATACGTAA |
| pCAM-CM2F | AGCTTCAAAAGCAAAAGCAAAAGACCCTTCCTCTATATAAGGAAGTTCATTTCATTTGGAGAGAACACGGC |
| pCAM-CM2R | CATGGCCGTGTTCTCTCCAAATGAAATGAACTTCCTTATATAGAGGAAGGGTCTTTTGCTTTTGCTTTTGA |
| pCAM-E-boxF | AGCTTGCAAATGAGCAAATGAGCAAATGAACCCTTCCTCTATATAAGGAAGTTCATTTCATTTGGAGAGAACACGGC |
| pCAM-E-boxR | CATGGCCGTGTTCTCTCCAAATGAAATGAACTTCCTTATATAGAGGAAGGGTTCATTTGCTCATTTGCTCATTTGCA |
| pCAM-mE-boxF | AGCTTGACCGCAAGACCGCAAGACCGCAAACCCTTCCTCTATATAAGGAAGTTCATTTCATTTGGAGAGAACACGGC |
| pCAM-mE-boxR | CATGGCCGTGTTCTCTCCAAATGAAATGAACTTCCTTATATAGAGGAAGGGTTTGCGGTCTTGCGGTCTTGCGGTCA |
| pCAM-E-boxp+F | AGCTTATCATATGCTTTTATTGCACGCACCCTTCCTCTATATAAGGAAGTTCATTTCATTTGGAGAGAACACGGC |
| pCAM-E-boxp+R | CATGGCCGTGTTCTCTCCAAATGAAATGAACTTCCTTATATAGAGGAAGGGTCAACAACTGAAAAGGAATGCGAGA |
| pCAM-E-boxp-F | AGCTTACCTTTGCCCTTTTACCCTATCACCCTTCCTCTATATAAGGAAGTTCATTTCATTTGGAGAGAACACGGC |
| pCAM-E-boxp-R | CATGGCCGTGTTCTCTCCAAATGAAATGAACTTCCTTATATAGAGGAAGGGTCATTTAAAGAAACCCGAAACCCTA |

Table S8: The primer sequences used in real time RT-PCR.

| **Gene** | **GenBank Accession number** | **Forward primers (5'-3')** | **Reverse primers (5'-3')** |
| --- | --- | --- | --- |
| *ThbZIP1* | Fj752700 | TGTTCGCTACGCAAACTTGGAC | TAATTCCTCTACGTCAGCATTC |
